# Expression of the cereblon binding protein argonaute 2 plays an important role for multiple myeloma cell growth and survival

**DOI:** 10.1186/s12885-016-2331-0

**Published:** 2016-05-03

**Authors:** Qinqin Xu, Yue-xian Hou, Paul Langlais, Patrick Erickson, James Zhu, Chang-Xin Shi, Moulun Luo, Yuanxiao Zhu, Ye Xu, Lawrence J. Mandarino, Keith Stewart, Xiu-bao Chang

**Affiliations:** Department of Biochemistry & Molecular Biology, Mayo Clinic College of Medicine, Mayo Clinic Arizona, 13400 E. Shea Boulevard, Scottsdale, AZ 85259 USA; Zhejiang Provincial Key Laboratory of Nephrology, Hangzhou Traditional Chinese Medical Hospital, 453 Tiyuchang Rd, Hangzhou, 310007 China; Division of Hematology-Oncology, Mayo Clinic Arizona, Scottsdale, AZ USA; Center for Metabolic and Vascular Biology, Arizona State University, Tempe, AZ USA; Key Laboratory of Carcinogenesis and Translational Research, Breast Center, Beijing Cancer Hospital & Institute, Peking University Cancer Hospital, Beijing, 100142 P. R. China; Department of Medicine, Mayo Clinic Arizona, Scottsdale, AZ USA

**Keywords:** Multiple myeloma (MM), Immunomodulatory drug (IMiD), Lenalidomide, Cereblon (CRBN), Argonaute 2 (AGO2), MicroRNA (miRNA)

## Abstract

**Background:**

Immunomodulatory drugs (IMiDs), such as lenalidomide, are therapeutically active compounds that bind and modulate the E3 ubiquitin ligase substrate recruiter cereblon, thereby affect steady-state levels of cereblon and cereblon binding partners, such as ikaros and aiolos, and induce many cellular responses, including cytotoxicity to multiple myeloma (MM) cells. Nevertheless, it takes many days for MM cells to die after IMiD induced depletion of ikaros and aiolos and thus we searched for other cereblon binding partners that participate in IMiD cytotoxicity.

**Methods:**

Cereblon binding partners were identified from a MM cell line expressing histidine-tagged cereblon by pulling down cereblon and its binding partners and verified by co-immunoprecipitation. IMiD effects were determined by western blot analysis, cell viability assay, microRNA array and apoptosis analysis.

**Results:**

We identified argonaute 2 (AGO2) as a cereblon binding partner and found that the steady-state levels of AGO2 were regulated by cereblon. Upon treatment of IMiD-sensitive MM cells with lenalidomide, the steady-state levels of cereblon were significantly increased, whereas levels of AGO2 were significantly decreased. It has been reported that AGO2 plays a pivotal role in microRNA maturation and function. Interestingly, upon treatment of MM cells with lenalidomide, the steady-state levels of microRNAs were significantly altered. In addition, silencing of AGO2 in MM cells, regardless of sensitivity to IMiDs, significantly decreased the levels of AGO2 and microRNAs and massively induced cell death.

**Conclusion:**

These results support the notion that the cereblon binding partner AGO2 plays an important role in regulating MM cell growth and survival and AGO2 could be considered as a novel drug target for overcoming IMiD resistance in MM cells.

**Electronic supplementary material:**

The online version of this article (doi:10.1186/s12885-016-2331-0) contains supplementary material, which is available to authorized users.

## Background

Immunomodulatory drugs (IMiDs), such as lenalidomide, are therapeutically active compounds widely used in the treatment of multiple myeloma (MM) [1]. Treatment with IMiDs results in significant effects on: immunomodulatory activities; anti-angiogenic activities; anti-inflammatory activities; anti-proliferation; pro-apoptotic effects; cell-cycle arrest; and inhibition of cell migration and metastasis [2]. Although significant remissions in patients with MM have been induced with IMiDs, the molecular mechanism of IMiDs’ action has only recently unraveled.

Using immobilized thalidomide, Ito et al. identified cereblon (CRBN) and DNA damage-binding protein 1 (DDB1) as binding proteins and further demonstrated that CRBN was the primary target of thalidomide-induced teratogenicity [3]. We subsequently found that CRBN expression was required for the anti-MM activity of IMiDs [4].

CRBN has been found to be an E3 ubiquitin ligase substrate recruiter [5–7], but the full functional role of CRBN in this complex is still not well known. In fact CRBN also binds to BK_Ca_ [8, 9], ClC-2 [10], AMPK [11], PSMB4 [12], ikaros (IKZF1) and aiolos (IKZF3) [13–15] and MEIS2 [16], thus it is possible that CRBN might function as a substrate-recruiter to bind each of these proteins for ubiquitination by the E3 ubiquitin ligase machinery and other binding partners with clinically relevant function may also exist.

Indeed, in this report, we have identified argonaute 2 (AGO2), also termed eukaryotic translation initiation factor 2 subunit C2 (EIF2C2), as a CRBN-downstream binding factor. AGO2 plays a pivotal role in microRNA (miRNA) maturation, stability and function [17–19]. We show that the treatment of IMiD-sensitive MM cells with lenalidomide significantly increased CRBN, subsequently decreasing both AGO2 protein and its target miRNAs and inducing apoptosis. Furthermore, directly reducing cellular AGO2 levels produced cellular cytotoxicity regardless of whether they are IMiD-sensitive or -resistant MM cells. Therefore, the expression of CRBN-downstream binding protein AGO2, by regulating miRNA levels, plays an important role for MM cell growth and survival.

## Results

### Lenalidomide-induced cell-death is a slow process

**Fig. 1:**
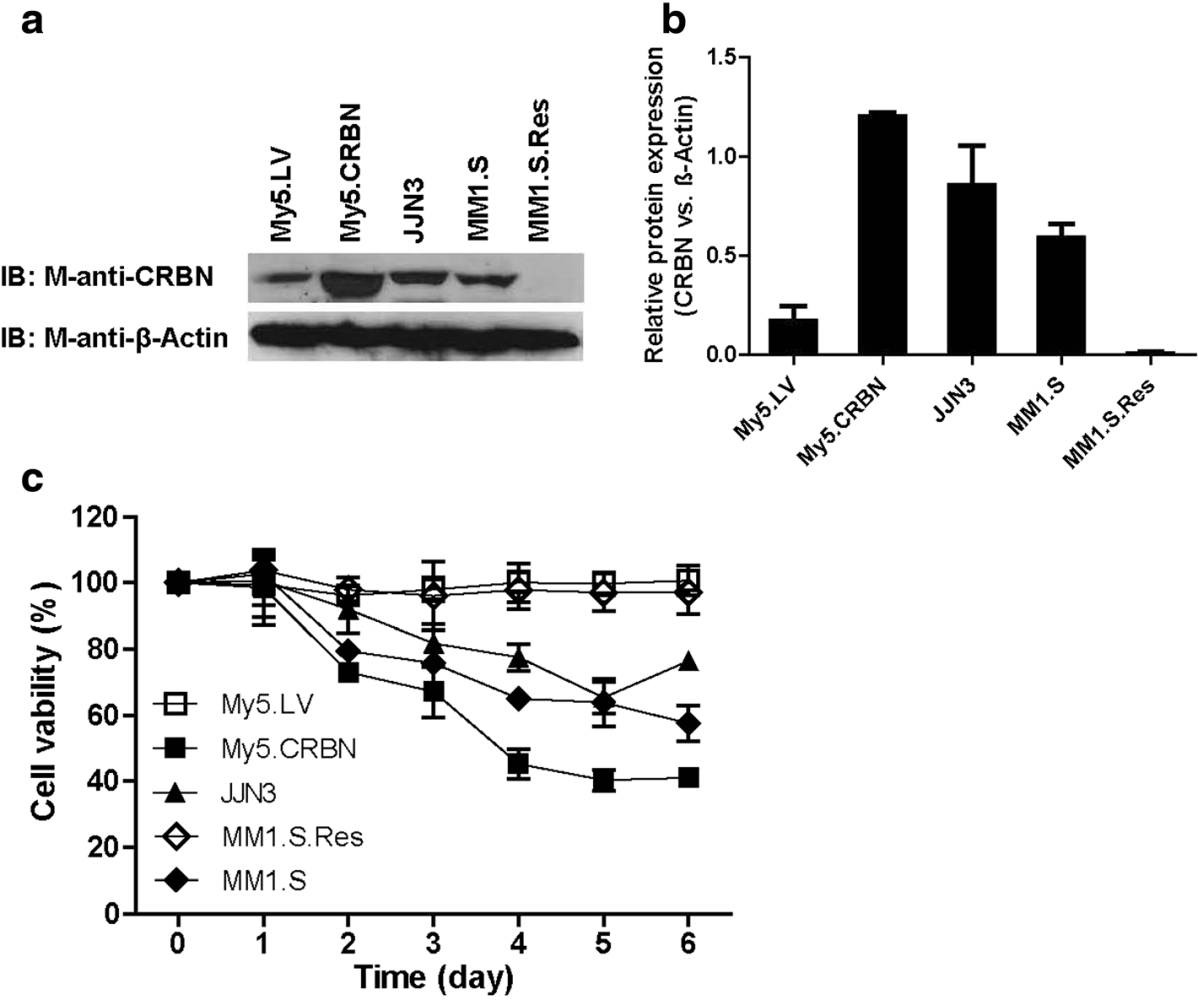
Effects of lenalidomide on the MM cell lines with variant levels of CRBN. **a** Expression of CRBN in MM cell lines. 100 μg of total proteins from whole cell lysates were loaded in each lane and probed with our recently developed mouse-anti-CRBN monoclonal antibody 2F11G5 (M-anti-CRBN) and mouse-anti-β-actin antibody (M-anti-β-Actin). **b** Comparison of CRBN expression in MM cell lines. The intensity of the β-actin band was considered as 1.0 and the relative intensity of CRBN band was compared with its corresponding β-actin band. **c**. The effects of lenalidomide on the MM cell lines. MM cells were plated in a volume of 200 μl at 10,000 cells per well in 96-well plate containing either DMSO (DMSO-treated control viabilities were considered as 100 %) or 10 μM lenalidomide. Cell survival was followed by MTT assay

We have found that CRBN expression is required for the anti-MM activity of lenalidomide [4]. IKZF1 and IKZF3 were found to be CRBN-downstream binding proteins [13–15, 20]. We have, however, noticed that although IKZF1 and IKZF3 were degraded within hours of the treatment with lenalidomide [13–15, 20], it can take many days for the IMiD-sensitive MM cells to die. In order to better understand the response of MM cells to IMiD, lentiviral particle harboring human CRBN cDNA infected My5 cells (My5.CRBN.His) and lentivirus vector (as a control) infected My5 cells (My5.LV) were treated with various concentrations of lenalidomide for several days and the survival of the cells was monitored by 3–(4,5-dimethylthiazol-2-yl)–2,5-diphenyltetrazolium bromide dye (MTT) assay. The results in Additional file 1: Figure S1 indicated that My5.LV cells, which express lower levels of CRBN, were resistant to lenalidomide, whereas My5. CRBN. His cells, which express higher levels of CRBN, were sensitive to lenalidomide, indicating that high level of CRBN is required for the anti-MM activity of lenalidomide. Despite the sensitivity observed, high concentrations of lenalidomide are also required for the IMiD-induced cell death (Additional file 1: Figure S1). Even with high concentration of lenalidomide, the CRBN-low MM cells, such as My5.LV and MM1.S. Res cells (Fig. 1a and b), cannot be efficiently killed (Fig. 1c). This observation is consistent with the conclusion made in [21]. Of note, the treatment of the MM cells expressing higher levels of CRBN, such as My5. CRBN, JJN3 and MM1.S (Fig. 1a and b), with 10 μM lenalidomide for one day did not have significant effect on them (Fig. 1c), suggesting that although IMiD-induced degradation of IKZF1 and IKZF3 occurs within hours, the effects of the degradation of these transcription factors on the proteins associated with cell growth and death may take days. These results also suggested that there might be other un-identified CRBN downstream binding factors that contribute to the delayed IMiD-induced cell death.

### Identification of AGO2 as a potential CRBN-downstream binding protein

In order to identify potential CRBN downstream binding factors, the two MM cell lines mentioned in Fig. 1, i.e., My5.LV and My5. CRBN. His, were used to do the pull-down experiments with nickel-charged agarose beads. As shown in Fig. 1, the infection of My5 cells with lentivirus vector did not alter the sensitivity to lenalidomide, whereas expression of the His-tagged CRBN in My5 cells increased the sensitivity to IMiD, suggesting that His-tagged CRBN is functional. Since we have functional His-tagged CRBN in human MM cells, it is possible for us to pull down the His-tagged CRBN and its binding proteins with nickel-charged agarose beads. In addition, lentivirus vector infected My5 (My5.LV) cells do not express His-tagged CRBN, providing a good negative control for our pull-down experiment.

In order to preserve their natural association between CRBN and its downstream binding proteins, My5. CRBN. His cells and their control My5.LV cells were broken by nitrogen cavitation without any detergent. 115 proteins, including CRBN itself, cullin-4B (CUL-4B) and AGO2, were detected in the pull-down samples from My5.CRBN.His cell lysates, but not from the control My5.LV cell lysates (Table 1 and Additional file 2: Table S2). In addition, 59 proteins with higher assigned spectra (higher than 5 fold), including DDB1 and CUL-4A, were detected in My5.CRBN.His cell lysates versus the negative control My5.LV cell lysates (Table 1 and Additional file 2: Table S2). Since CUL-4A, CUL-4B and DDB1 are members of the E3-CRBN ubiquitin ligase complex, pulling down each of these components with His-tagged CRBN suggested that the other proteins, such as AGO2 (Table 1), pulled-down from My5.CRBN.His cells, but not from negative control cells, might also be CRBN binding factors.

**Table 1:** Proteins pulled-down with Ni++ − charged beads

Proteins	My5/LV cell lysates	My5/CRBN cell lysates
CRBN	0^a^	28^a^
CUL-4B	0	6
AGO2	0	6
DDB1	6	81
CUL-4A	2	19

^a^The number in each column represents the number of assigned spectra for that protein

### Validation of AGO2 as a CRBN-downstream binding protein

**Fig. 2:**
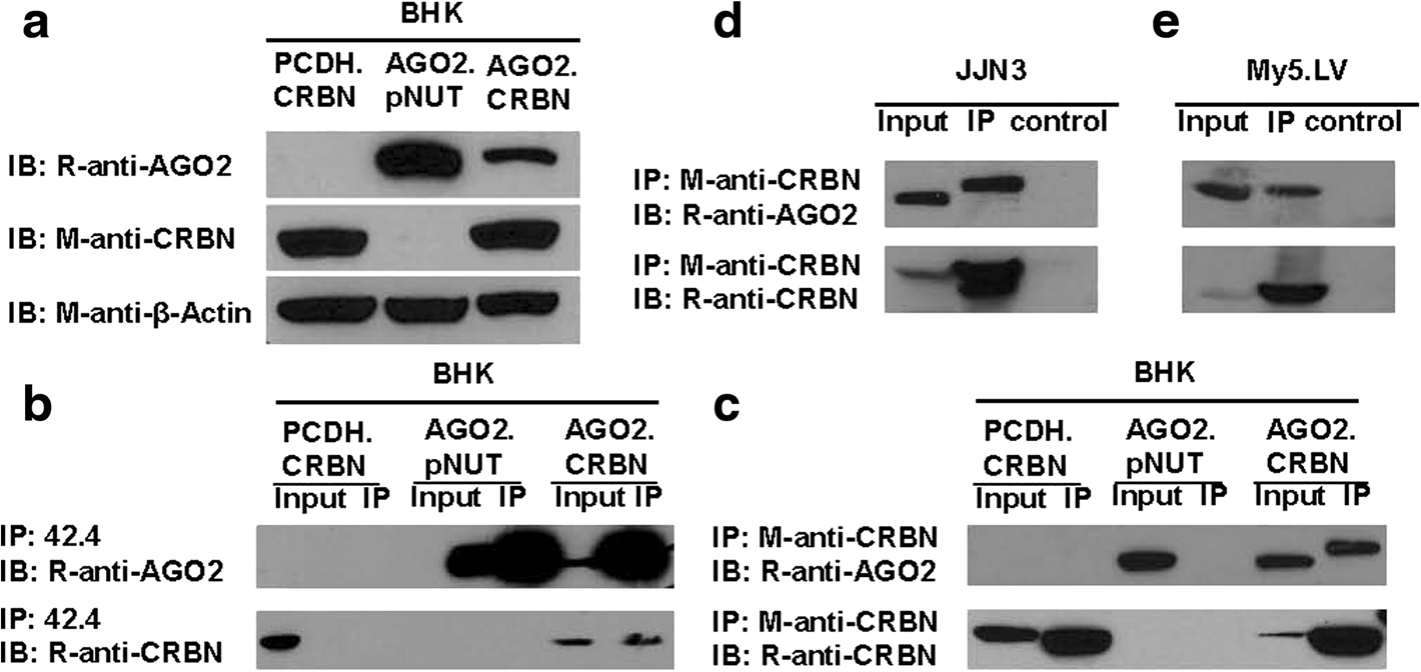
Validation of AGO2 as a CRBN-downstream binding protein. **a** Expression of His-tagged CRBN and MRP1 antibody 42.4 epitope-tagged AGO2 in BHK cells. Total proteins from whole cell lysates were separated on an acrylamide gel and probed with mouse-anti MRP1 antibody 42.4 (42.4), M-anti-CRBN and M-anti-Actin antibodies. **b** Co-IP of CRBN with 42.4-tagged AGO2. Samples were IPed with 42.4 and probed with either rabbit-anti-CRBN (R-anti-CRBN) or rabbit-anti-AGO2 (R-anti-AGO2). **c** Co-IP of 42.4-tagged AGO2 with CRBN. The three cell lysates mentioned above were IPed with our mouse-anti-CRBN antibody 2F11G5 and probed with either rabbit-anti-CRBN or rabbit-anti-AGO2. **d** & **e** Co-IP of wild-type AGO2 with endogenous CRBN in JJN3 cell (d) and in My5.LV cell (e). Samples were IPed with our mouse-anti-CRBN antibody 2F11G5 and probed with either rabbit-anti-AGO2 or rabbit-anti-CRBN. Input: whole cell lysates; IP: whole cell lysates immunoprecipitated (IPed) with M-anti-CRBN; Control: whole cell lysates IPed with protein G beads without adding primary antibody; IB: samples probed with the antibodies indicated in the figure

In order to validate AGO2 is a CRBN binding protein, AGO2 and CRBN were expressed alone or in combination in baby hamster kidney (BHK) cells. The results in Additional file 1: Figure S2 indicated that all the methotrexate (MTX) resistant BHK cells express high levels of GFP, suggesting that the MTX resistant cells harbored both pCDH.GFP.AGO2.42.4 and pNUT.CRBN.His plasmid DNAs. The results in Fig. 2a indicate that: 1) pCDH.CRBN cells express CRBN, but not AGO2; 2) AGO2.pNUT cells express AGO2, but not CRBN; 3) AGO2.CRBN cells express both CRBN and AGO2. Interestingly, the level of AGO2 in AGO2.pNUT cells is significantly higher than in CRBN expressing AGO2.CRBN cells (Fig. 2a), suggesting that the steady-state levels of AGO2 might be regulated by CRBN.

To further confirm whether AGO2 is a CRBN-downstream binding protein, cell lysates were prepared from the aforementioned three BHK cell lines and used to do co-immunoprecipitation (Co-IP) with our mouse monoclonal antibody 42.4 [22] which recognizes the epitope tag in AGO2.42.4. The results in Fig. 2b indicated that 42.4 did not pull-down His-tagged CRBN from either pCDH.CRBN or AGO2.pNUT cell lysates (Fig. 2b), but clearly pulled-down the His-tagged CRBN from AGO2.CRBN cell lysates (Fig. 2b). Subsequently, these cell lysates were used to do Co-IP with our mouse monoclonal antibody 2F11G5 which recognizes human CRBN. The results in Fig. 2c clearly indicated that this antibody pulled down CRBN and AGO2 from AGO2.CRBN cell lysates, suggesting that AGO2 is a CRBN downstream binding protein.

In order to confirm whether the un-tagged proteins could also be co-IPed with mouse monoclonal antibody against CRBN, the cell lysates from MM cell expressing higher level of CRBN (JJN3 in Fig. 1a and b) or lower level of CRBN (My5.LV in Fig. 1a and b) were used to do Co-IP with 2F11G5. The results in Fig. 2d and e clearly indicated that 2F11G5 antibody pulled-down endogenous CRBN and AGO2, further confirming that AGO2 is a CRBN-downstream binding protein.

### Lenalidomide treatment of MM cells affects the steady-state levels of CRBN and AGO2

**Fig. 3:**
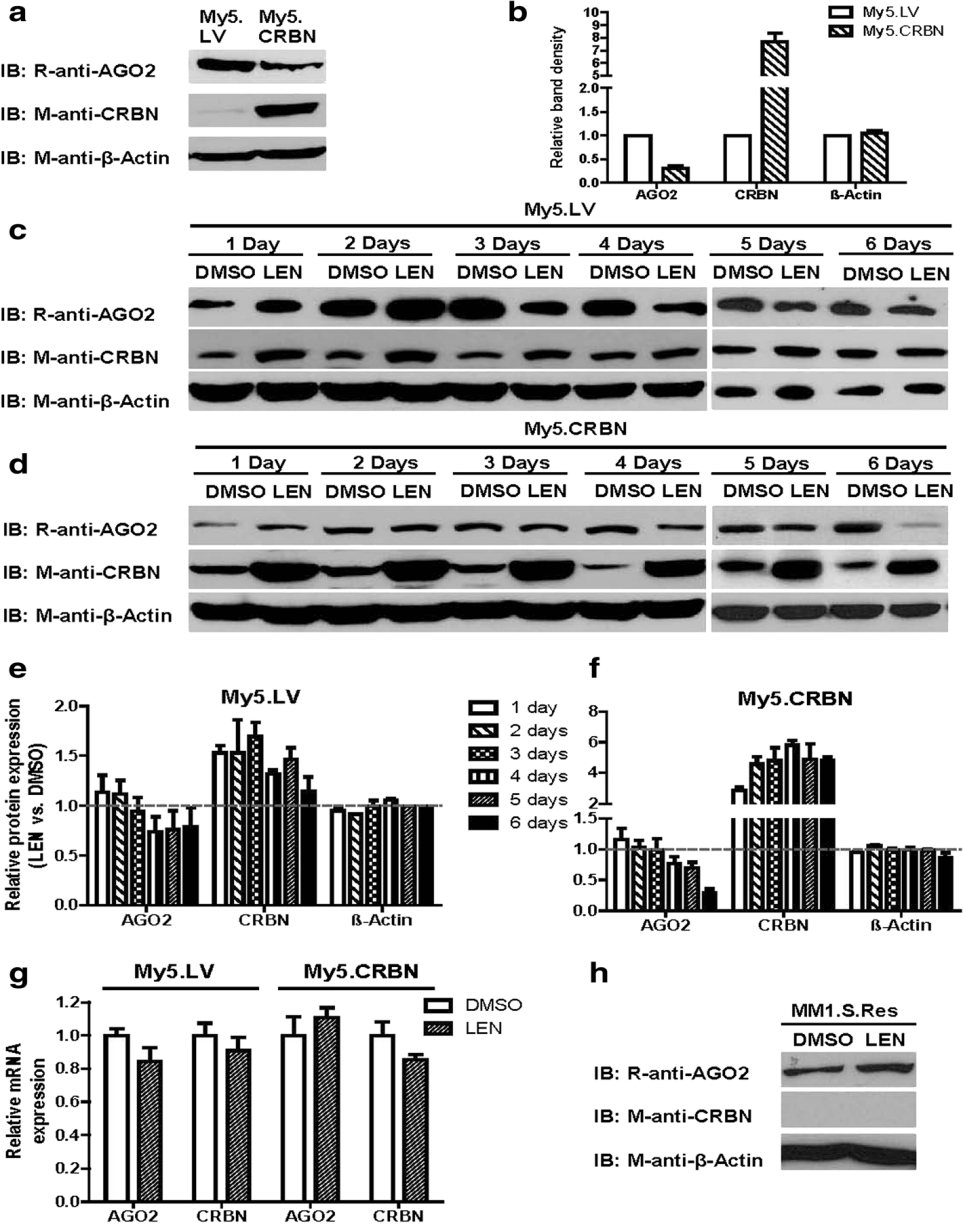
Lenalidomide treatment of MM cells affected the steady-state levels of CRBN and AGO2. **a** CRBN and AGO2 protein expression in My5.LV cell or in My5.CRBN cell. 100 μg of total proteins from whole cell lysates were loaded in each lane and analyzed by western blot. **b** The steady-state levels of AGO2, CRBN and β-actin were compared between My5.LV and My5.CRBN cells. **c** & **d** Relative levels of AGO2, CRBN and β-actin. Cells were treated with either DMSO (control) or 10 μM lenalidomide (LEN) for the time indicated in the figure. 100 μg of total proteins from My5.LV cells (**c**) or My5.CRBN cells (**d**) were loaded in each lane and analyzed by western blot. **e** & **f** Comparison of the protein expression between the samples treated with either DMSO or lenalidomide. The steady-state levels of AGO2, CRBN and β-actin in My5.LV cells (**e**) or in My5.CRBN cells (**f**) were compared between the treatments with either DMSO (considered as 1.0) or lenalidomide. **g** Quantitative analysis of AGO2 mRNA or CRBN mRNA. Total RNA was isolated from My5.LV and My5.CRBN cells treated with either DMSO or 10 μM lenalidomide for six days and used to do quantitative polymerase chain reaction (qPCR). **h** Relative levels of AGO2, CRBN and β-actin in MM1.S.Res cells treated with either DMSO or 10 μM lenalidomide. MM1.S.Res cells were treated with either DMSO or 10 μM lenalidomide for six days. 100 μg of total proteins from these treated cells were loaded in each lane and analyzed by western blot

We and others have found that the treatment of MM cells with IMiDs affected the steady-state levels of CRBN and its downstream binding factors, such as IKZF1 and IKZF3 [13–15, 20]. Based on these results, we speculated that the treatment of MM cells with IMiDs should also affect the steady-state levels of AGO2. In fact, the steady-state levels of AGO2 in BHK cells expressing both CRBN and AGO2 are significantly less than in BHK cells expressing AGO2 along (Fig. 2a), suggesting that CRBN may down-regulate AGO2. We then checked the relative levels of AGO2 in lentiviral vector-treated My5 cell or in CRBN cDNA-treated My5 cell (Fig. 3a) and showed that the steady-state level of CRBN in My5.CRBN cell is approximately 8 fold higher than in My5.LV (Fig. 3b), whereas AGO2 in CRBN-high My5.CRBN cell is approximately 30 % of CRBN-low My5.LV (Fig. 3b), suggesting that CRBN may down-regulate its downstream binding protein AGO2.

In order to test the effects of IMiDs on the steady-state levels of AGO2, CRBN-low My5.LV and CRBN-high My5.CRBN cells were treated with either dimethyl sulfoxide (DMSO) or lenalidomide. It is clear, from the results in Fig. 3c and e, that the steady-state levels of CRBN, upon treatment of CRBN-low My5.LV cells with 10 μM lenalidomide, were slightly increased. However, the steady-state levels of AGO2, upon treatment of CRBN-low My5.LV cells with 10 μM lenalidomide, were not significantly altered within 2 or 3 days incubation (Fig. 3e and Additional file 1: Figure S3) and slightly decreased after 4 days (Fig. 3c and e). In contrast, although CRBN in My5.CRBN cell is approximately 8 fold higher than in My5.LV cell (Fig. 3b), the steady-state levels of CRBN in lenalidomide treated CRBN-high My5.CRBN cells is approximately five fold higher than the DMSO treated cells (Fig. 3d and f). Of note, although AGO2 in CRBN-high My5.CRBN cells is approximately 30 % of CRBN-low My5.LV cells (Fig. 3b), the steady-state levels of AGO2 in lenalidomide treated CRBN-high My5.CRBN for 6 days is approximately 20 % of the DMSO treated cells (Fig. 3f). In addition, the treatment of CRBN-low My5.LV or CRBN-high My5.CRBN cells with lenalidomide for 6 days did not significantly alter the levels of AGO2 mRNA or CRBN mRNA (Fig. 3g), suggesting that lenalidomide post-translationally, in a CRBN-dependent manner, modulates AGO2. Interestingly, the treatment of MM1.S.Res cells, which express undetectable amount of CRBN (Fig. 1a and b), with lenalidomide for 6 days did not significantly affect the steady-state levels of AGO2 (Fig. 3h), suggesting that CRBN is required for lenalidomide-mediated degradation of AGO2.

### Degradation of AGO2 is directly associated with effective CRBN

**Fig. 4:**
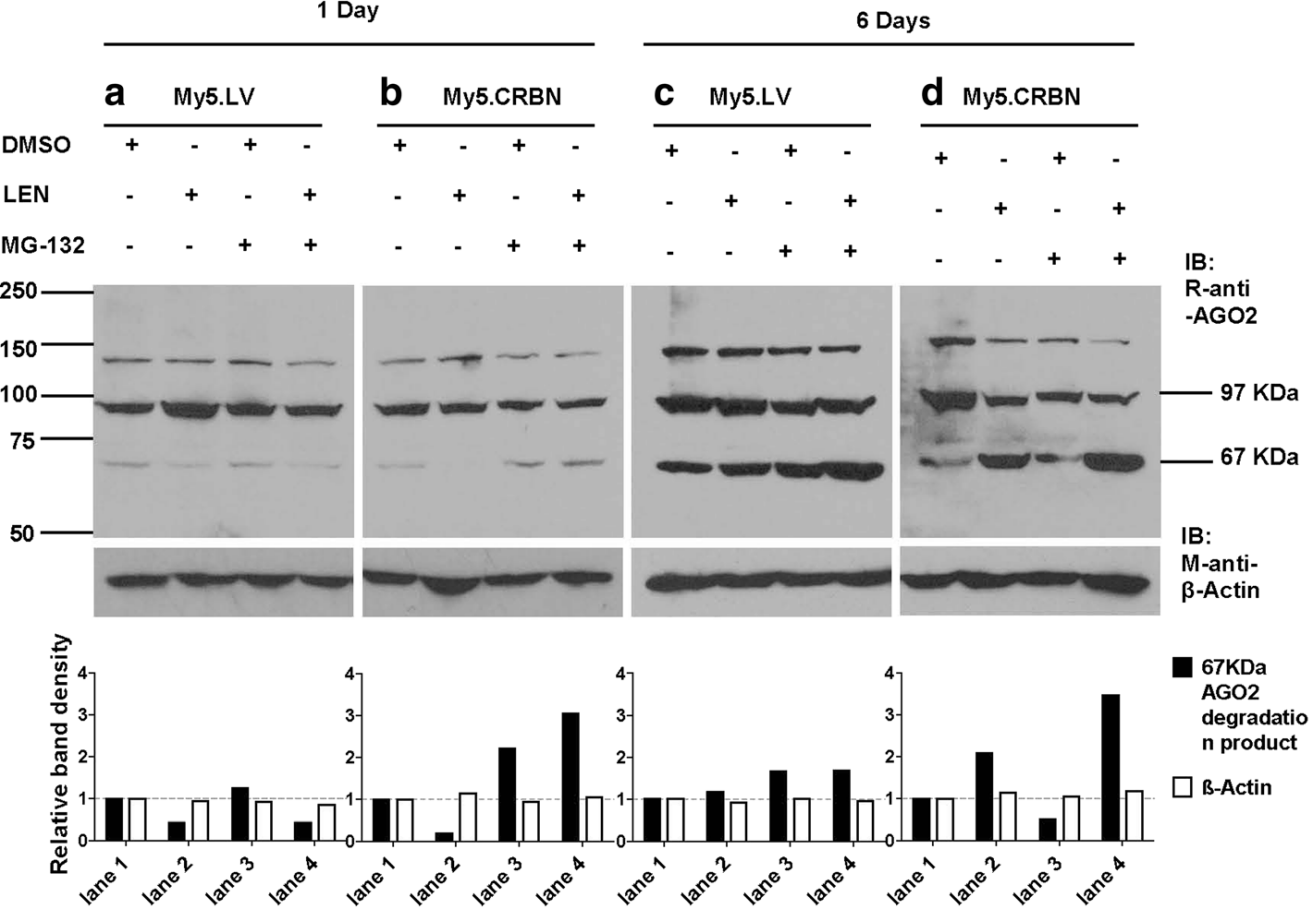
Lenalidomide treatment of MM cells affected degradation of AGO2. **a** & **b** The effects of lenalidomide treatment of My5.LV cells (**a**) or My5.CRBN cells (**b**) for 1 day. MM cells were treated with either DMSO (−) or 10 μM lenalidomide (+) for 18 hours and then 10 μM proteasome inhibitor MG132 (+) were added to the media for additional 6 hours. **c** & **d** The effects of lenalidomide treatment of My5.LV cells (**c**) or My5.CRBN cells (d) for 6 days. MM cells were treated with either DMSO (−) or 10 μM lenalidomide (+) for 138 hours and then 10 μM proteasome inhibitor MG132 (+) were added to the media for additional 6 hours. 100 μg of total proteins from whole cell lysates were loaded in each lane and analyzed by western blot. The intensity of β-actin band or the 67 kDa AGO2 degradation product in samples neither treated with lenalidomide nor MG132 was considered as 1 and the relative amount of β-actin or the 67 kDa AGO2 degradation product in other samples was calculated accordingly

The results in Fig. 3 suggested that the binding of lenalidomide to CRBN may prevent E3-CRBN-proteasome mediated degradation of AGO2. In order to prove this hypothesis, the CRBN-low My5.LV cells and CRBN-high My5.CRBN cells were treated with lenalidomide and proteasome inhibitor MG132 and the degradation products of AGO2 were monitored with AGO2 antibody in western blots. It is clear that, upon treatment of CRBN-low My5.LV cells or CRBN-high My5.CRBN cells for 24 hours, the accumulation of AGO2 degradation product (67 kDa) was significantly decreased (lane 2 in Fig. 4a and b), suggesting that binding of lenalidomide to CRBN not only prevents E3-CRBN-mediated ubiquitination of AGO2, but also proteasome-mediated degradation. The treatment of CRBN-low My5.LV cells with proteasome inhibitor MG132 did not significantly increased the accumulation of the 67 kDa AGO2 degradation product (lane 3 in Fig. 4a), suggesting that E3-CRBN-proteasome-mediated degradation of AGO2 in CRBN-low My5.LV cells is not very efficient. This conclusion is supported by the result derived from My5.LV cells treated with lenalidomide and MG132 (lane 4 in Fig. 4a). In contrast, the treatment of CRBN-high My5.CRBN cells with proteasome inhibitor MG132 did significantly increase, regardless of whether lenalidomide was added or not, the accumulation of the 67 kDa AGO2 degradation product (lane 3 and 4 in Fig. 4b), suggesting that lenalidomide-free CRBN in CRBN-high My5.CRBN cells can recruit AGO2 for E3-CRBN-proteasome-mediated degradation.

The results in Fig. 3, upon treatment of MM cells with lenalidomide for 6 days, suggested that AGO2 degradation might be directly associated with the amount of lenalidomide-free CRBN in MM cells. In order to prove this hypothesis, the degradation products of AGO2, upon treatment of the CRBN-low My5.LV cells or CRBN-high My5.CRBN cells with lenalidomide for 6 days, were monitored with AGO2 antibody in western blots. It is clear that, upon treatment of MM cells with lenalidomide for 6 days, the accumulation of the 67 kDa AGO2 degradation product in CRBN-low My5.LV cells was slightly increased (lane 2 in Fig. 4c), whereas the accumulation of this degradation product in CRBN-high My5.CRBN cells was significantly increased (lane 2 in Fig. 4d), suggesting that the treatment with lenalidomide for 6 days accumulated much more lenalidomide-free CRBN in CRBN-high My5.CRBN cells than in CRBN-low My5.LV cells. Interestingly, although most of the AGO2 protein in CRBN-high My5.CRBN cells were degraded upon treatment with lenalidomide for 6 days (Fig. 3d and f), the treatment with proteasome inhibitor MG132 significantly increased the accumulation of the 67 kDa AGO2 degradation product (lane 4 in Fig. 4d), suggesting that the treatment with lenalidomide for 6 days accumulated much more lenalidomide-free CRBN in CRBN-high My5.CRBN cells than in CRBN-low My5.LV cells. These results support our hypothesis that the E3-CRBN-proteasome-mediated degradation of AGO2 is directly associated with lenalidomide-free CRBN or effective CRBN.

### The effects of AGO2-shRNA treatment on MM cells

We have noticed that it took days to decrease the steady-state levels of AGO2 (Fig. 3) and wondered whether this is related to the slow process of lenalidomide-induced MM cell death (Fig. 1c). To test this hypothesis, AGO2-shRNAs and AGO2 itself were used to treat the AGO2-high My5.LV cells and AGO2-low My5.CRBN cells.

**Fig. 5:**
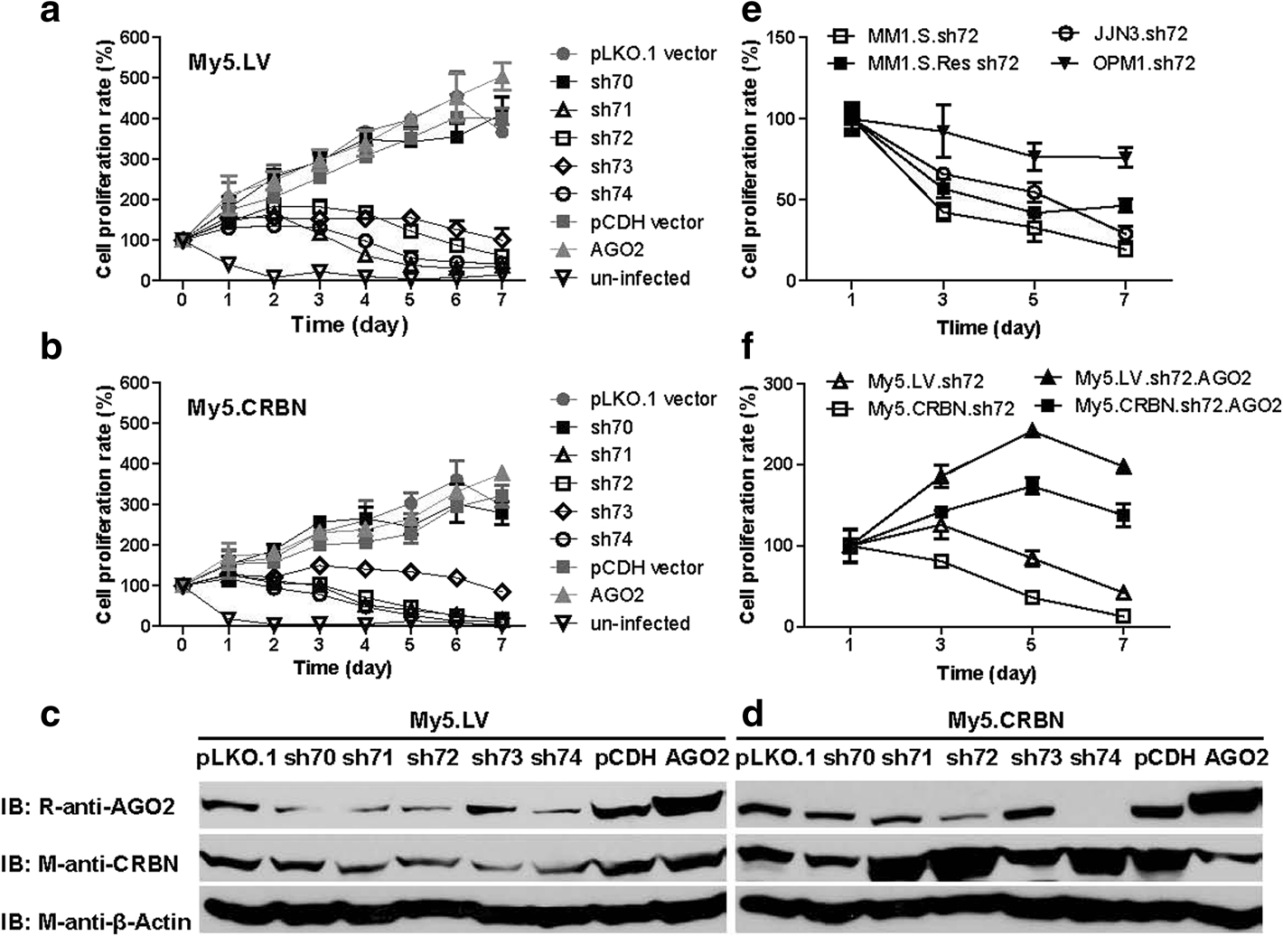
Silencing of AGO2 induced cytotoxicity in IMiD-resistant and IMiD-sensitive MM cells. **a** & **b** The effects of silencing AGO2 with AGO2-shRNA on MM cell growth and survival. IMiD-resistant My5.LV cells (**a**) and IMiD-sensitive My5.CRBN cells (**b**) were infected with lentiviral particles harboring either lentiviral vector, AGO2-shRNA or AGO2 cDNA. The media were replaced 18 hours post-infection and then 1 μM puromycin (final concentration) was added 24 hours later (day0). Cell survival was followed by MTT assay. **c** & **d** The effects of silencing AGO2 with AGO2-shRNA on the steady-state levels of AGO2, CRBN and β-actin. IMiD-resistant My5.LV cells (**c**) and IMiD-sensitive My5.CRBN cells (**d**) were lysed three days post infection with viral particles indicated in (**a)** and (**b**) and 100 μg of total proteins from these treated cells were loaded in each lane and analyzed by western blot. **e** The effects of silencing AGO2 with AGO2-shRNA on other MM cell lines. **f** Expression of recombinant AGO2 can reverse the AGO2.shRNA induced cytotoxicity. The infection of My5.LV and My5.CRBN cells with viral particles harboring AGO2-shRNA-72 was performed exactly the same as in (**a**) and (**b**) The rescue experiments were performed by: 1) collecting the MM cells treated with AGO2-shRNA-72 for six days; 2) infecting these cells with viral particles harboring AGO2 cDNA; 3) replacing the media 18 hours post-infection; 4) performing MTT assay 24 hours later (day1)

Interestingly, the treatment of My5.LV or My5.CRBN cells with pLKO.1 vector or pCDH.puro vector did not have a significant effect on cell growth and survival, whereas the treatment with AGO2-shRNA, especially the treatment with sh72 and sh74, decreased the levels of AGO2 (Fig. 5c and d), and induced cell death (Fig. 5a and b), regardless of their sensitivity to IMiDs (Fig. 1c). The treatment of other MM cell lines, such as JJN3, OPM1, MM1.S and MM1.S.Res, with AGO2-sh72 also induced cell death (Fig. 5e). Although the treatment of My5.LV and My5.CRBN cells with AGO2 cDNA increased the steady-state levels of AGO2 (Fig. 5c and d), this treatment did not have a significant effect on cell growth and survival (Fig. 5a and b), but slightly increased IC50 value of lenalidomide for IMiD-sensitive My5.CRBN cells (Additional file 1: Figure S7). However, the treatment of AGO2-sh72 treated My5.LV or My5.CRBN cells with AGO2 cDNA did increase their growth and survival (Fig. 5f). Of note, the growth rate of the MM cells expressing higher levels of CRBN is lower than the cells with lower levels of CRBN (Additional file 1: Figure S4A, S4B and S4C). In addition, the cytotoxicities induced by the treatment with AGO2-shRNA in AGO2-low My5.CRBN cells occurred much earlier than in AGO2-high My5.LV cells (Additional file 1: Figure S4D), suggesting that it might take longer time to decrease the AGO2 level in AGO2-high My5.LV cells to a critical point to inhibit cell growth or to induce cell death.

### Reducing the expression of AGO2 affected steady-state levels of microRNAs

**Fig. 6:**
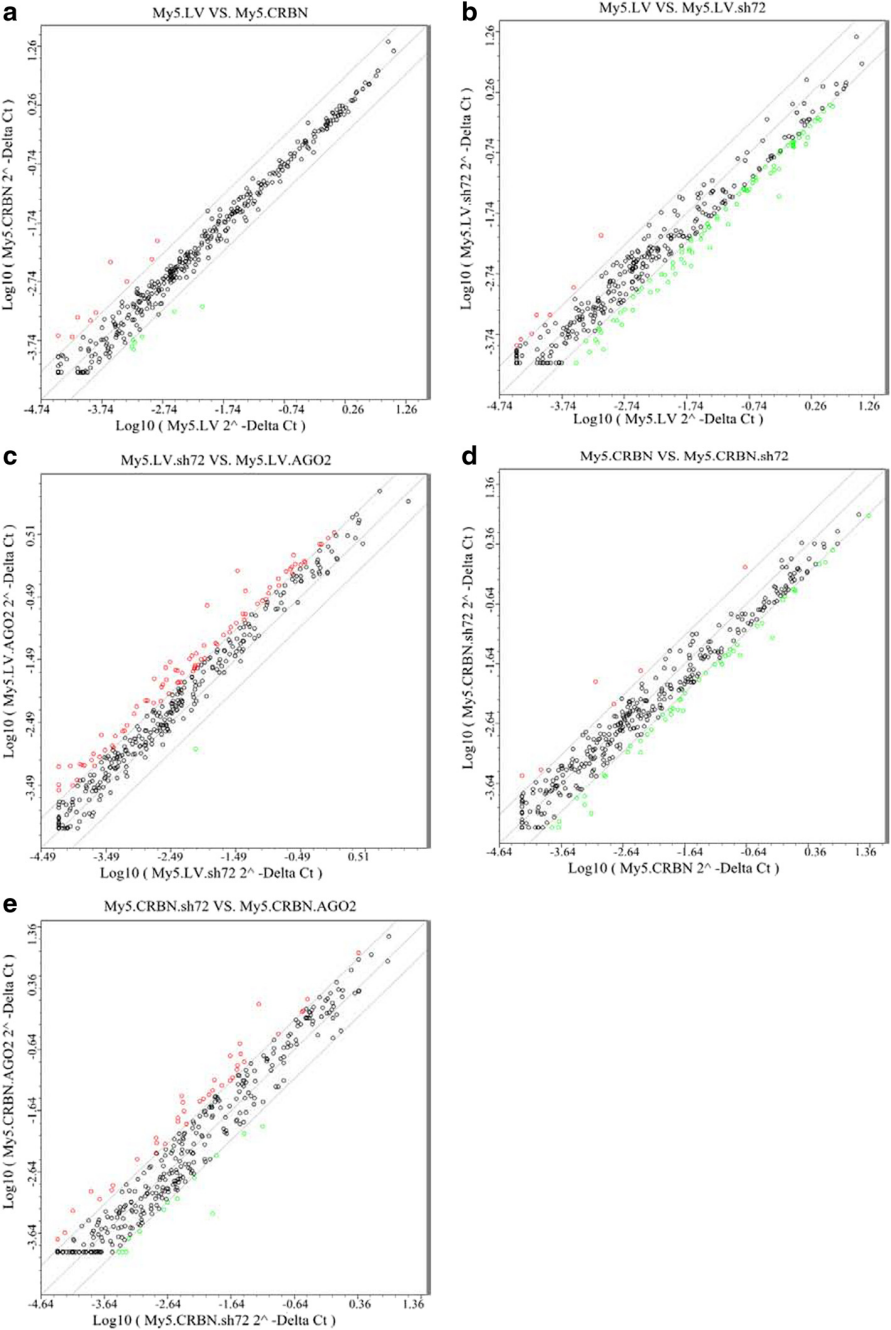
Silencing of AGO2 with its shRNA altered the steady-state levels of miRNAs. Total RNAs were isolated from MM cells treated with either AGO2-shRNA72 (sh72) or AGO2 cDNA (AGO2) for three days and the steady-state levels of miRNAs were analyzed with microRNA array kit. **a** Comparison of the steady-state levels of miRNAs between My5.LV and My5.CRBN cells; **b** between My5.LV and sh72 treated My5.LV cells (My5.LV.sh72); **c** between sh72 treated My5.LV (My5.LV.sh72) and AGO2 treated My5.LV cells (My5.LV.AGO2); **d** between My5.CRBN and sh72 treated My5.CRBN cells (My5.CRBN.sh72); **e** between AGO2-sh72 treated My5.CRBN (My5.CRBN.sh72) and AGO2 treated My5.CRBN cells (My5.CRBN.AGO2)

AGO2 is considered as a master regulator of miRNA maturation and function [17–19]. In order to test the effects of altering the levels of AGO2 on miRNAs, total RNAs were isolated and used to determine the steady-state levels of 372 miRNAs. The results in Fig. 6a indicated that, without any treatment, majority of the miRNAs in My5.LV cell were similar to that in My5.CRBN cell. However, the steady-state levels of 88 miRNAs in AGO2-sh72 treated My5.LV cells were at least 3 fold lower than in the un-treated My5.CRBN cells (Additional file 1: Figure S5F), suggesting that silencing of AGO2 decreased the stability of miRNAs. This conclusion is further supported by the comparison between the un-treated and AGO2-sh72-treated My5.LV cells (Fig. 6b and Additional file 1: Figure S5C). In contrast, increasing the level of AGO2, by treatment of My5.LV cells with AGO2 cDNA, significantly enhanced the steady-state levels of many miRNAs (Additional file 1: Figure S5B). Interestingly, if the comparison is done between AGO2-sh72 treated My5.LV cells and AGO2 cDNA treated My5.LV cells, majority of the miRNAs were increased (Fig. 5c and Additional file 1: Figure S5D), suggesting that the stabilities of miRNAs in MM cells are associated with the intracellular levels of AGO2.

For cells expressing higher level of CRBN, such as My5.CRBN, CRBN may play an important role in regulating the intracellular levels of AGO2 (Figs. 2a and 3a). Decreasing the level of AGO2 by treatment with AGO2-sh72 resulted in decreasing many miRNAs for at least 3 fold (Fig. 6d and Additional file 1: Figure S5E). In contrast, increasing the level of AGO2, by the treatment of My5.CRBN cells with AGO2 cDNA, significantly enhanced the steady-state levels of miRNAs (Fig. 6e and Additional file 1: Figure S5H). In summary, higher levels of AGO2 resulted in enhanced levels of many miRNAs whereas reduced levels of this protein lead to decreased levels of many miRNAs, as shown in Additional file 2: Table S3 and Additional file 1: Figure S5.

### Treatment of MM cells with lenalidomide significantly affects steady-state levels of miRNAs

**Fig. 7:**
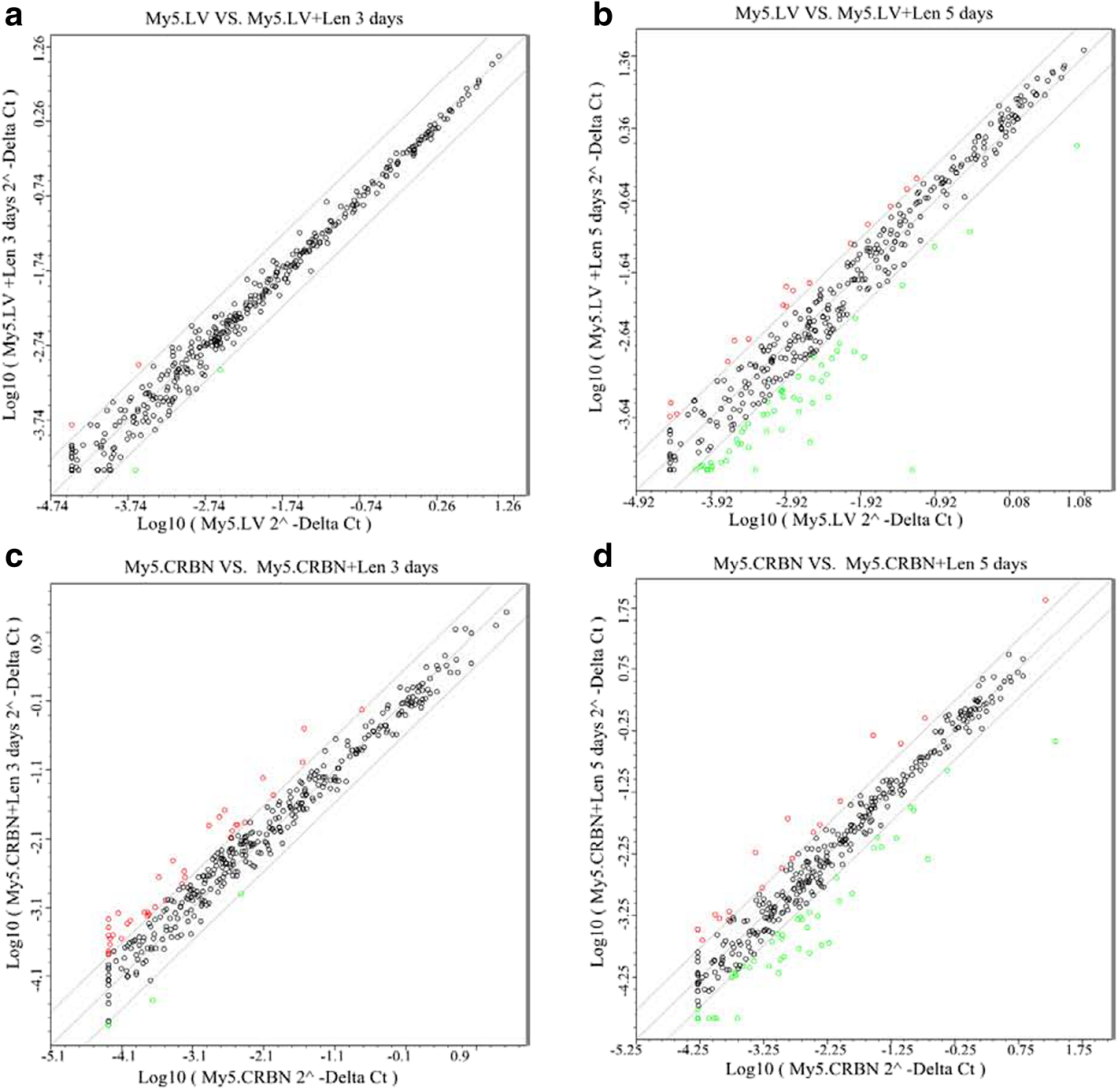
Treatment of MM cells with lenalidomide altered steady-state levels of miRNAs. Total RNAs were isolated at day 3 or day 5 post 10 μM lenalidomide treatment and the steady-state levels of miRNAs were analyzed with microRNA array kit. **a** Comparison of the steady-state levels of miRNAs between My5.LV and My5.LV treated with 10 μM lenalidomide for 3 days; **b** between My5.LV and My5.LV treated with 10 μM lenalidomide for 5 days; **c** between My5.CRBN and My5.CRBN treated with 10 μM lenalidomide for 3 days; **d** between My5.CRBN and My5.CRBN treated with 10 μM lenalidomide for 5 days

Since the degradation of AGO2 was associated with the time of lenalidomide treatment (Fig. 3e and f), the effects of IMiD on the steady-state levels of miRNAs should also be associated with incubation time. For example, upon treatment of IMiD-resistant My5.LV cells with 10 μM lenalidomide for 3 days, the levels of AGO2 were not significantly affected, whereas this protein dropped to ~ 80 % after 5 days (Fig. 3e). Interestingly, the levels of miRNAs were not significantly affected after 3 days treatment with lenalidomide (Fig. 7a), whereas many miRNAs were either up- or down-regulated at least 3 fold after 5 days treatment (Fig. 7b and Additional file 1: Figure S6A), suggesting that miRNAs’ stability is related to the intracellular levels of AGO2. In the case of IMiD-sensitive My5.CRBN cells, many miRNAs were up-regulated at least 3 fold after 3 days treatment with lenalidomide (Fig. 7c and Additional file 1: Figure S6Q), whereas treatment for 5 days resulted in down-regulating many miRNAs (Fig. 7d and Additional file 1: Figure S6J). These results are consistent with our notion that higher levels of AGO2 mainly up-regulate miRNAs whereas lower levels of this protein primarily down-regulate miRNAs (Fig. 6). Further comparison of the samples treated with lenalidomide for 5 days with other samples (Additional file 2: Table S4 and Additional file 1: Figure S6B-S6I and S6K-S6Q) strongly supported the above conclusion.

### Treatment of MM cells, regardless of their IMiD-sensitivities, with AGO2-shRNA induced apoptosis

**Fig. 8:**
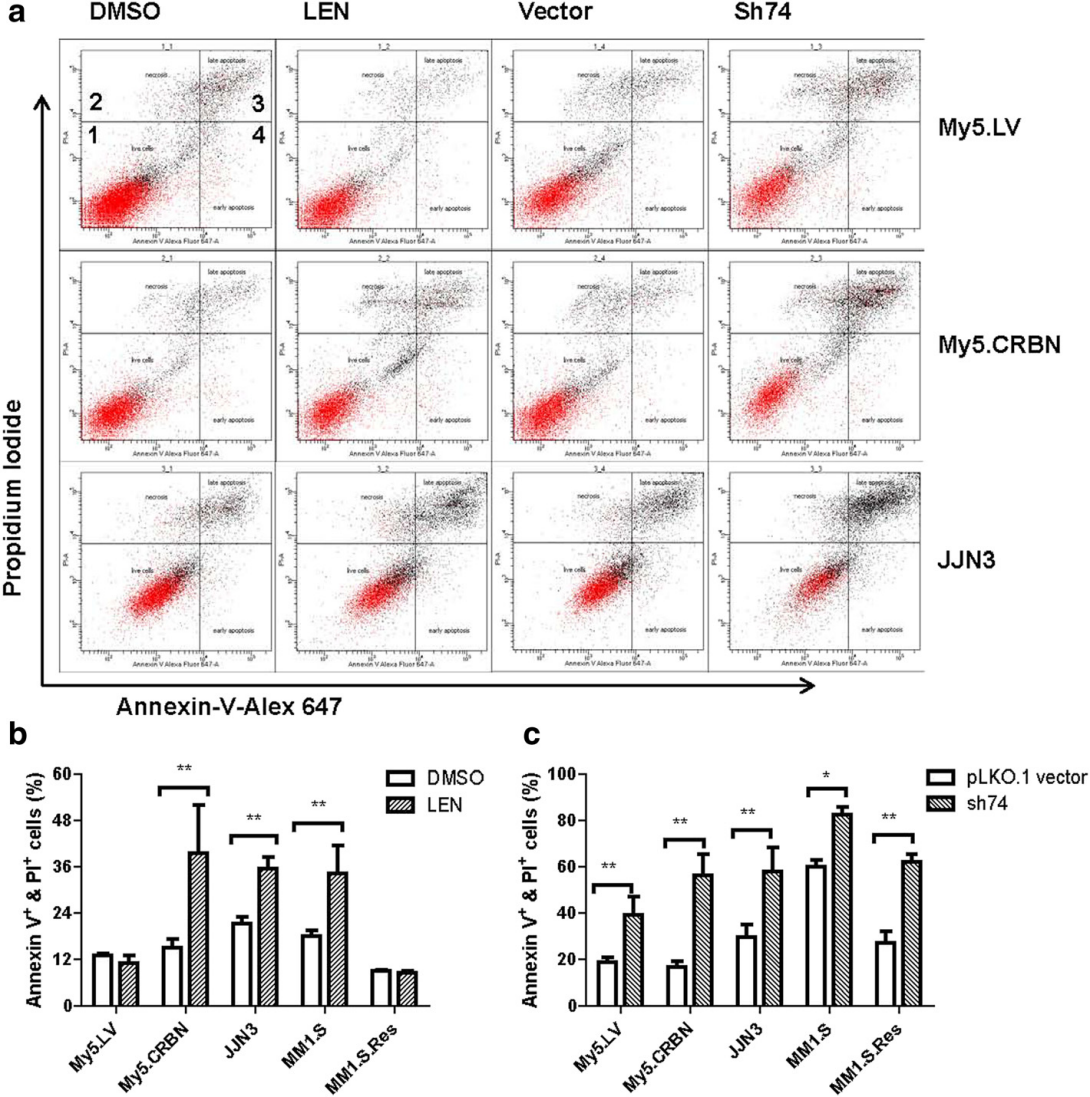
Treatment of MM cells, regardless of their IMiD-sensitivities, with AGO2-shRNA induced apoptosis. **a** Profiles for apoptosis analysis with Alexa Fluor 647 Annexin V. MM cells were treated with either DMSO (as a control) or 10 μM lenalidomide (LEN) for six days; or with either lentiviral vector (as a control) or AGO2-shRNA74 (sh74) for four days, respectively. The cells were stained with annexin V-Alexa Flour 647 and 0.5 mg/mL propidium iodide (PI) and then analyzed by flow cytometry. Region 1 indicates the live cells; 2, necrotic cells; 3, late apoptotic cells; 4, early apoptotic cells. **b** Effects of lenalidomide on MM cells. Annexin V^+^ indicates annexin V positive cells. PI^+^ indicates propidium iodide positive cells. The bar graph indicates the mean ± standard deviation. *P value < 0.05; **P value < 0.01. **c** Effects of AGO2-shRNA74 on MM cells

Based on our results presented above, we hypothesized that: 1) the interaction between IMiDs and CRBN will inhibit degradation of CRBN, leading to enhanced steady-state levels of CRBN (Fig. 3); 2) the enhanced steady-state levels of CRBN, once the bound IMiDs are dissociated, will recruit AGO2 for E3 ubiquitin ligase-proteasome mediated degradation; 3) the enhanced degradation of AGO2 will decrease the steady-state levels of AGO2 to a critical point that leads to decrease the stability of miRNAs; 4) some of the decreased miRNAs may be responsible for the expression of the proteins associated with cell growth, survival and apoptosis; 5) treatment of MM cells, regardless of their sensitivities to IMiDs, with AGO2-shRNA should induce apoptosis. To test this hypothesis, the five MM cell lines used in Fig. 1 were treated with either 10 μM lenalidomide or AGO2-shRNA-74 and analyzed with an Alexa Fluor 647 Annexin V kit to determine the percentage of live cells, apoptotic cells and necrotic cells (Fig. 8a).

It is clear that the treatment of CRBN-low MM cells, such as My5.LV or MM1.S.Res (Fig. 1a and b), with 10 μM lenalidomide did not induce apoptosis (Fig. 8b), whereas the treatment of CRBN-high MM cells, such as My5.CRBN, JJN3 or MM1.S (Fig. 1a and b), with 10 μM lenalidomide significantly induced apoptosis (Fig. 8b). In contrast, the treatment of MM cells, regardless of their steady-state levels of CRBN, with AGO2-shRNA-74 significantly induced apoptosis (Fig. 8c), suggesting that AGO2 could be considered as a novel drug target to overcome IMiD resistance.

## Discussion and Conclusions

We have identified AGO2 as a CRBN-downstream binding protein. This conclusion is based on: 1) AGO2 was pulled down with His-tagged CRBN (Table 1 and Additional file 2: Table S2); 2) CRBN was co-IPed with 42.4-tagged AGO2 (Fig. 2b); 3) 42.4-tagged AGO2 was co-IPed with CRBN (Fig. 2c); 4) endogenous AGO2 was co-IPed with wild-type CRBN (Fig. 2d and e); 5) the steady-state levels of AGO2 in CRBN-high MM cells are significantly lower than the corresponding CRBN-low MM cells (Figs. 2a and 3a); and 6) treatment of MM cells with lenalidomide affects the steady-state levels of AGO2 (Fig. 3c, d, e and f) and miRNAs (Fig. 7b and d).

AGO2 is considered as a master regulator of miRNA maturation and function [17–19, 23–25] and miRNAs regulate up to 90 % of human genes via a silencing process mediated by miRNA-induced silencing complexes (miRISCs) [23]. Dysregulation of miRNAs is associated with cancer initiation and progression [26, 27]. It has been found that: 1) miR-125b induced myeloid leukemia by enhancing myeloid progenitor output from stem cells as well as inducing immortality, self-renewal and tumorigenesis in myeloid progenitors [28]; 2) high-risk myeloma is associated with global elevation of miRNAs and over-expression of AGO2 [29]; and 3) over-expression of AGO2 resulted in increased miRNA accumulation [17, 30]. However, the mechanism of AGO2 regulation is largely un-known. Now we have found that AGO2 is a CRBN-downstream binding factor that is tightly regulated by the effective CRBN (Fig. 4) at the post-translational level. In addition, we have found that the steady-state levels of AGO2 in CRBN-high MM cells are significantly lower than the corresponding CRBN-low MM cells. Therefore, dysregulation of CRBN in cancer cells is responsible for malfunctions of AGO2 and miRNAs.

It has been reported that IMiDs decreased the expression of vascular endothelial growth factor and basic fibroblast growth factor [31], thereby inhibiting new blood vessel formation and decreasing the tumor growth. Indeed, microvessel growth in the IMiDs treated samples was significantly less than in the corresponding controls [32–37]. However, the molecular mechanism of IMiD-induced anti-angiogenic effects is not well documented. Recent finding indicated that over-expression of AGO2 increased angiogenesis, via regulation of miRNA levels, whereas silencing of AGO2 inhibited angiogenesis [38]. We have found, in this report, that AGO2 is a CRBN-downstream binding protein and the treatment of MM cells with lenalidomide for 5 days resulted in decreased both AGO2 (Fig. 3) and miRNAs (Fig. 7), indicating that IMiD-induced anti-angiogenic effects may go through CRBN-AGO2-miRNA pathway.

We conclude that AGO2 plays an important role in regulating MM cell growth and survival. This conclusion is based on our finding that silencing AGO2 expression halted MM cell growth (Fig. 5a and b). In other words, MM cell growth requires higher levels of AGO2. Based on this conclusion, we predicted that the growth rate of My5.LV cells, which have significant higher levels of AGO2 than My5.CRBN cells (Fig. 3a and b), should be higher than that of My5.CRBN cells. Indeed, the growth rates of pLKO.1- and pCDH-treated My5.LV cells were significantly higher than the corresponding treated My5.CRBN cells (Additional file 1: Figure S4A and S4B). In addition, the growth rate of AGO2-cDNA-treated My5.LV cells was significantly higher than the corresponding treated My5.CRBN cells (Additional file 1: Figure S4C), implying that the steady-state levels of AGO2 might be controlled by CRBN. Furthermore, the cell survival of the AGO2-high My5.LV cells, upon treatment with AGO2-sh72, is significantly higher than the AGO2-low My5.CRBN cells treated with the same shRNA (Additional file 1: Figure S4D), suggesting that it may take longer time to decrease the endogenous AGO2 to a critical point to inhibit cell growth in AGO2-high My5.LV cells than in AGO2-low My5.CRBN cells.

One puzzle we had in the past is that the time of IMiD-induced IKZF1 or IKZF3 degradation (within hours) and IMiD-induced cytotoxicity (days) is not temporally consistent. IKZFs are zinc finger transcription factors that play important roles in lymphocyte differentiation [39, 40]. Treatment with IMiDs or IKZF-shRNA not only induced fast-degradation of IKZFs [13–15, 20], but also modulate, within relatively short time, the expression of their downstream factors, such as interferon regulatory factor 4 (IRF4) [13, 15] or interleukin-2 (IL-2) [20]. However, the time required for IKZF-shRNA-induced cytotoxicity was significantly longer than the time of IMiD-induced cell death [13, 14], implying that there might be other CRBN binding factors that participate in IMiD-induced cytotoxicity. We have found that AGO2 is a CRBN binding factor that is modulated by lenalidomide. AGO2 is a critical component of miRISCs that modulate a wide variety of protein syntheses at translational level. Interestingly, the time of lenalidomide-induced degradation of AGO2 (Fig. 3f) is consistent with lenalidomide-induced alteration of miRNAs (Fig. 7c and d). In addition, the time of lenalidomide-induced alteration of miRNAs (Fig. 7c and d) is also more or less consistent with the time of lenalidomide induced cell death (Fig. 1c). Therefore, although detailed mechanism of IMiD-induced cell death is not clear yet, part of the IMiD-induced cell death may go through CRBN-AGO2-miRNAs pathway.

The conclusion made in previous paragraph elicited a question of whether AGO2 is the sole factor responsible for the early onset of IMiD-induced cytotoxicity. We have found that: 1) the steady-state levels of AGO2 in IMiD-resistant My5.LV cells, upon treatment with lenalidomide, were not significantly affected (Fig. 3e and g); 2) the treatment of IMiD-resistant My5.LV cells with 10 μM lenalidomide for 3 days did not significantly alter the steady-state levels of miRNAs (Fig. 7a). Therefore, these results are consistent with IMiD-resistance of My5.LV cells (Fig. 1c). However, we have also found that, upon treatment of My5.LV cells with 10 μM lenalidomide for 5 days, the steady-state levels of some miRNAs were significantly down-regulated (Fig. 7b). This fact is more or less consistent with lenalidomide-induced moderate down-regulation of AGO2 (Fig. 3e) and mild cytotoxicity (Additional file 1: Figure S1). One possible explanation for this fact is that lenalidomide treatment for 5 days did not decrease the levels of critical miRNAs to a threshold to massively trigger apoptosis in those MM cells expressing low levels of CRBN. However, in considering the following observations: 1) the miRNAs altered by the treatment with AGO2-sh72 are not exactly the same as the treatment with lenalidomide (Fig. 6b versus Fig. 7b or Fig. 6d versus Fig. 7d or Additional file 1: Figure S5 versus S6); 2) the proteins altered by the treatment with AGO2-sh72 are not exactly the same as the treatment with lenalidomide [For example, IKZF1 or IKZF3 in My5.CRBN cells was significantly increased upon treatment with AGO2-sh72, whereas these proteins were significantly decreased upon treatment with lenalidomide (data not shown)]; 3) slightly different response of My5.LV and My5.CRBN cells to AGO2-shRNAs was observed (Additional file 1: Figure S4D); we cannot rule out the possibility that some other un-identified CRBN-downstream binding factors may also participate in IMiD-induced cytotoxicity. In addition, the effects occurred within the first two or three days of lenalidomide treatment may not be directly associated with AGO2 (Figs. 1c and 5). Furthermore, although the sensitivity of MM cells to lenalidomide is associated with: 1) the intracellular levels of CRBN (Fig. 1); 2) the differential regulation of miRNAs, upon treatment with lenalidomide, in MM cells with variant levels of CRBN (Additional file 1: Figure S6R and S6S), the detailed molecular mechanism of lenalidomide induced cell death is not clear yet.

It is clear that IMiD binding to CRBN enhanced accumulation of CRBN, especially in IMiD-sensitive cells (Fig. 3f), and eventually resulted in decreased levels of AGO2 (Fig. 3). The decreased levels of AGO2 (Fig. 3 and 5) resulted in down-regulation of miRNAs (Fig. 6 and 7) that further affected protein syntheses. It is not clear, however, which protein synthesis is affected by the decreased levels of miRNAs. It has been reported that silencing of AGO2 with its shRNA enhanced protein expression of cyclin-dependent kinase (CDK) inhibitors p21Waf1/Cip1 and p27Kip1 [29]. Since these proteins are CDK inhibitors, enhanced expression of these proteins will inhibit CDKs and result in enhanced cell cycle arrest [29]. Enhanced expression of p21Waf1/Cip1 might be regulated by decreased level of miR-106 [29]. Indeed, the steady-state levels of miR-106a-3p and miR-106b-3p were significantly reduced upon treatment of the MM cells with lenalidomide for 5 days (Additional file 1: Figure S6C, S6D, S6G, S6K, S6L, and S6O). In fact, many miRNAs were either up-regulated or down-regulated at least 4 fold upon treatment of the MM cells with either AGO2-shRNA or lenalidomide (Additional file 2: Tables S3 and S4). Although we still don’t know how many proteins were up- or down-regulated upon these treatments, the treatment of IMiD-resistant or IMiD-sensitive MM cells (Fig. 1c) with AGO2-shRNA strongly inhibited cell growth and induced cell death (Figs. 5 and 8). Therefore, AGO2 could be considered as a novel therapeutic target for overcoming IMiD-resistance in MM cells.

## Methods

### Cell culture

Human MM cell lines were maintained in RPMI-1640 medium (Thermo Scientific) supplemented with 10 % heat-inactivated fetal bovine serum (Thermo Scientific) and 1 % penicillin/streptomycin. BHK cells were cultured in DMEM/F12 medium (Thermo Scientific) supplemented with 5 % fetal bovine serum and 1 % penicillin/streptomycin.

### Pull-down His-tagged CRBN and its associated proteins

10 histidine residues were introduced into the C-terminus of human CRBN cDNA by polymerase chain reaction (PCR) and then cloned into pCDH-CMV-MCS-EF1-copGFP (green fluorescent protein) expression vector (System Bioscience, Mountain view, CA), named as pCDH.GFP.CRBN.His. Lentiviral particles harboring the cloning vector and pCDH.GFP.CRBN.His were prepared, according to the method described [41], and used to infect human myeloma OCI-My5 (My5) cells to generate control cell line My5.LV and CRBN expressing cell line My5.CRBN.His. The infected MM cells were sorted for GFP expression at day 14 after infection.

My5.LV and My5.CRBN.His cells were grown up in RPMI-1640 medium at 37 °C, treated with 2 mM sodium butyrate for overnight before harvesting, harvested by centrifugation and stored at - 80 °C. The cell pellets were re-suspended in 1 × binding buffer (20 mM Tris/HCl, pH7.9; 500 mM NaCl; and 10 % glycerol) with protease inhibitors and then equilibrated on ice for 30 minutes at 800 p.s.i. in a Parr nitrogen cavitation bomb [22]. After releasing the pressure, the cell lysates were centrifuged at 33,000 × g for 30 minutes at 4 °C to collect supernatant. The pellet was re-suspended in 1 × binding buffer, sonicated for 20 pulses on ice and centrifuged at 33,000 × g for 30 minutes at 4 °C to collect supernatant. The two supernatants were combined and the protein concentration of the supernatants was determined. Equal amount of proteins from My5.LV supernatant or My5.CRBN.His supernatant were mixed, in two separated tubes, with similar amount of nickel-charged NTA (Ni-NTA) agarose beads, adjusted to have 5 mM imidazole, and gently rotated on a rocker overnight at 4 °C. The loaded Ni-NTA agarose beads were packed into a column (in two separated columns) and washed with 1 × binding buffer containing 10 mM imidazole (1^st^ wash), 20 mM imidazole (2^nd^ wash) and 40 mM imidazole (3^rd^ wash) [42]. The bound proteins were eluted with 1 ×binding buffer containing 100 mM EDTA.

### Tandem mass spectrometry (MS/MS) analysis

For the proteomics experiments, the proteins pulled-down with nickel-charged NTA agarose beads were resolved by a 10 % sodium dodecyl sulfate polyacrylamide gel electrophoresis (SDS-PAGE). The gel bands (10 gel bands per lane) were excised and destained, digested with trypsin, and desalted using ZipTips (Millipore, Billerica, MA) as previously described [43]. HPLC-ESI-MS/MS analysis was performed on a Thermo Scientific Orbitrap Elite Velos Pro fitted with an EASY-Spray Ion Source (Thermo Scientfic, Waltham, MA). On-line HPLC was performed using a Thermo Scientific Dionex Ultimate 3000 Series Nano/Cap System NCS-3500RS nanoLC with an Acclaim PepMap100 trap column (ThermoScientific, 75 μm ID ×2 cm, 3 μm C18, 100 Å) and an Acclaim PepMap RSLC analytical column (ThermoScientific, 75 μm ID ×15 cm, 2 μm C18, 100 Å); loading was performed at flow rate of 6 nl/min, and elution was performed at a gradient of 2 to 35 % Buffer B for 65 minutes, followed by a step to 90 % Buffer B and a hold for 5 minutes, followed by a return to 4 % Buffer B (Buffer A: 0.1 % formic acid in water, Buffer B: 80 % acetonitrile in 0.1 % formic acid and water); analytical flow rate, 300 nl/min. A “top-10” data-dependent MS/MS analysis was performed (acquisition of a full scan spectrum followed by collision-induced dissociation mass spectra of the 10 most abundant ions in the survey scan). The fragment mass spectra were then searched against the human SwissProt_2013_02 database (539,165 entries) using Mascot (Matrix Science, London, UK; version 2.4). The search variables used were: 1) 10 ppm mass tolerance for precursor ion masses and 0.5 Da for product ion masses; 2) digestion with trypsin; 3) a maximum of two missed tryptic cleavages; 4) variable modifications of oxidation of methionine and phosphorylation of serine, threonine, and tyrosine. Cross-correlation of Mascot search results with X! Tandem was accomplished with Scaffold (version Scaffold_4.2.1; Proteome Software, Portland, OR, USA). Probability of peptide assignments and protein identifications were made through the use of Scaffold. Only peptides with ≥ 95 % probability were considered.

### Validation of AGO2 as a CRBN downstream binding protein

In order to validate AGO2 as a CRBN binding protein, the epitope of 42.4 antibody, a multidrug resistance-associated protein 1 (MRP1) antibody [22], was introduced into the C-terminus of human AGO2 cDNA (from DNASU, Tempe, AZ) by PCR and then cloned into the pCDH-CMV-MCS-EF1-copGFP expression vector that provides GFP expression, named as pCDH.GFP.AGO2.42.4. In the meantime, the 10 His-tagged human CRBN cDNA was shifted from pCDH.GFP.CRBN.His to pNUT expression vector [44, 45], named as pNUT.CRBN.His.

Three cell lines had been established by introducing, via calcium phosphate transfection method [42], the following pairs of DNAs: 1) pCDH.GFP.AGO2.42.4 + pNUT (named as AGO2/pNUT); 2) pCDH-CMV-MCS-EF1-copGFP + pNUT.CRBN.His (named as pCDH/CRBN); 3) pCDH.GFP.AGO2.42.4 + pNUT.CRBN.His (named as AGO2/CRBN), into BHK cells [22]. Permanent cell lines were established by selection with 100 μM MTX.

For co-immunoprecipitation (Co-IP) of CRBN with AGO2, the aforementioned three BHK cell lines were lysed with NP40 lysis buffer (0.1 % NP40; 150 mM NaCl; 10 mM NaMoO_4_• 2H_2_0; and 50 mM Tris–HCl, pH7.6), supplemented with 1 ×protease inhibitor cocktail containing: Aprotonin, 2 μg/mL; Benzamide, 121 μg/mL; E64, 3.5 μg/mL; Leupeptin, 1 μg/mL; and Pefabloc, 50 μg/mL. Primary antibody, after pre-cleaning with protein G beads (Invitrogen), was added to the cell lysates and gently rotated overnight at 4 °C. Protein G beads were added to the antibody treated cell lysates and washed with NP40 cell lysis buffer three times. The bound proteins were eluted with 45 μL of 2 ×SDS-PAGE loading buffer at 90 °C for 10 minutes.

To Co-IP of AGO2 with CRBN, the cell lysates prepared from My5.LV and JJN3 MM cell lines were IPed with our recently made anti-human CRBN monoclonal antibody 2F11G5.

### Immunoblotting

Western blot was performed according to the routine protocol. Briefly, equal amounts of proteins were subjected to SDS-PAGE, followed by transferring the proteins to nitrocellulose membranes, probed with primary antibody overnight at 4 °C, washed with phosphate buffered saline containing 0.1 % Tween-20 and then incubated with appropriate horseradish peroxidase-conjugated secondary antibody. Chemiluminescent film detection was performed according to the manufacturer’s recommendation. Anti-human CRBN mouse monoclonal antibodies, 2B11G10 and 2F11G5, were made by ourselves (through GenScript). Rabbit-anti-CRBN antibody was purchased from Sigma, whereas rabbit-anti-AGO2 antibody or other antibodies were from Cell Signaling Technology.

### MTT assay

Cell viability was measured by employing 3–(4,5-dimethylthiazol-2-yl)–2,5-diphenyltetrazolium bromide (MTT) dye (Sigma-Aldrich) performed according to the method described [42, 46].

### Modulating the expression of human AGO2 in MM cells

The 42.4-tagged AGO2 cDNA was shifted from pCDH.GFP.AGO2.42.4 to pCDH-CMV-MCS-EF1-Puro, named as pCDH.puro.AGO2.42.4, and used to make lentiviral particles, according to the routine method. AGO2-shRNA constructs were purchased from Sigma-Aldrich (The sequences of the shRNAs are shown in Additional file 2: Table S1) and used to make lentiviral particles. In the meantime, pLKO.1, the vector which harbored AGO2-shRNAs, and pCDH-CMV-MCS-EF1-Puro, the vector which harbored 42.4-tagged AGO2 cDNA, were also used to make lentiviral particles. These viral particles were used to infect My5.LV or My5.CRBN.His cells and selected with 1 μM puromycin, performed according to the routine method.

### MicroRNA array (miRNA array) and quantitative polymerase chain reaction (qPCR)

For miRNA array, total RNA was isolated from My5 cells with miRNeasy Mini kit (QIAGEN). Reverse transcription was performed with miScript II RT kit (QIAGEN), according to the protocol provided by QIAGEN. Quantitative analysis of miRNA was performed with miScript SYBR Green PCR Kit (QIAGEN) and miScript miRNA PCR Arrays (QIAGEN), performed with our Applied Biosystems 7900HT Fast Real-Time PCR System, according to the protocol provided by the manufacturer. Data analyses were performed with QIAGEN online software. The miRNA array data presented in this manuscript have been deposited in the NCBI GEO database (accession no. GSE61693).

For qPCR, total RNA was used to do qPCR with either CRBN primers (forward: 5’-CAGTCTGCCGACATCACATAC; reverse: 5’-GCACCATACTGACTTCTTGAGGG) or AGO2 primers (forward: 5’-TCCACCTAGACCCGACTTTGG; reverse: 5’-GTGTTCCACGATTTCCCTGTT).

### Apoptosis analysis

The Alexa Fluor 647 Annexin V kit (BioLegend) was used for detecting early apoptotic cells, late stage apoptotic cells, necrotic cells and live cells, performed according to the manufacture’s instructions.

### Statistical analysis

Mean values and two-tailed P values were calculated based on the unpaired t test from GraphPad Software Quick Calcs. By conventional criteria, if P value is less than 0.05, the difference between two samples is considered to be statistically significant.

### Availability of data and materials

The miRNA array datasets supporting the conclusions of this article are available in the NCBI GEO database (Accession no. GSE61693 and hyperlink to datasets in http://www.ncbi.nlm.nih.gov/geo/query/acc.cgi?acc=GSE61693).

The datasets supporting the conclusions of this article are included within the article and its additional files.

## Additional files

Additional file 1: Figure S1. Lenalidomide induced cell death is a slow process. Cell survival was monitored by MTT assay. **Figure S2.** Co-transfection of pCDH.GFP.AGO2 with pNUT.CRBN.His yielded methotrexate (MTX)-resistant cells that express high levels of GFP. Pictures were taken from MTX-selected BHK cells that were transfected with the mixed DNAs containing pCDH.GFP.AGO2 and pNUT.CRBN.His**. Figure S3.** AGO2 expression is associated with cell growth. Cell lysates were harvested from My5.LV without any treatment (−), treated with dimethyl sulfoxide (DMSO) or 10 μM lenalidomide (LEN) and analyzed by western blot (probed with AGO2-specific antibody). **Figure S4.** The growth rate of the MM cells with higher levels of CRBN is lower than the cells with low levels of CRBN. Cell survival, after infection of My5.LV or My5.CRBN.His cells with viral particles containing pCDH vector (A), AGO2 cDNA (B), pLKO.1 vector (C) or AGO2-shRNA-72 (D), was followed by MTT assay**. Figure S5.** Silencing of AGO2 with its shRNA or over-expression of AGO2 altered the steady-state levels of miRNAs. Total RNAs were isolated after AGO-shRNA72 (sh72) treatment for 3 days or over-expression of AGO2 and the steady-state levels of miRNAs were analyzed with microRNA array kit. Scatter plots (a), heat-maps (b) and up- or down-regulated miRNAs (c) were established by comparison of the steady-state levels of miRNAs between two samples listed in each plot. **Figure S6.** Treatment of MM cells with lenalidomide altered steady-state levels of miRNAs. Total RNAs were isolated after lenalidomide treatment for 3 or 5 days and the steady-state levels of miRNAs were analyzed with microRNA array kit. Scatter plots (a), heat-maps (b) and up- or down-regulated miRNAs (c) were established by comparison of the steady-state levels of miRNAs between two samples listed in each plot. DOCX 5669 kb) Additional file 2: Table S1. Sequence of human AGO2 small hairpin RNA (shRNA). **Table S2.** Proteins detected by tandem mass spectrometry analyses. **Table S3.** The miRNA up- or down-regulated by AGO2 shRNA. **Table S4.** The miRNA up- or down-regulated by lenalidomide treatment. (DOCX 159 kb)

## Abbreviations

AGO2: argonaute 2
BHK: baby hamster kidney cell
CDK: cyclin-dependent kinase
Co-IP: co-immunoprecipitation
CRBN: cereblon
CUL-4A: culin-4A
CUL-4B: culin-4B
DDB1: DNA damage-binding protein 1
DMSO: dimethyl sulfoxide
EIF2C2: eukaryotic translation initiation factor 2 subunit C2
GFP: green fluorescent protein
IKZF1: ikaros
IKZF3: aiolos
IL-2: interleukin-2
IMiDs: Immunomodulatory drugs
IRF4: interferon regulatory factor 4
miRISCs: microRNA-induced silencing complexes
miRNA: microRNA
MM: multiple myeloma
MRP1: multidrug resistance-associated protein 1
MS/MS: Tandem mass spectrometry
MTT: 3-(4,5-dimethylthiazol-2-yl)-2,5-diphenyltetrazolium bromide dye
MTX: methotrexate
My5.CRBN.His: lentiviral particles harboring human CRBN cDNA infected My5 cells
My5.LV: lentivirus vector infected My5 cells
Ni-NTA: Nickel-charged NTA agarose beads
pCDH: pCDH-CMV-MCS-EF1
PCR: polymerase chain reaction
qPCR: quantitative polymerase chain reaction
SDS-PAGE: sodium dodecyl sulfate polyacrylamide gel electrophoresis
shRNA: small hairpin RNA
