# Supplementary material for: Expression of the cereblon binding protein argonaute 2 plays an important role for multiple myeloma cell growth and survival
Source: BMC Cancer. 2016 May 3;16:297. doi: 10.1186/s12885-016-2331-0 (PMC4855823; doi:10.1186/s12885-016-2331-0)
Supplement: Additional file 1: Figure S1. — Lenalidomide induced cell death is a slow process. Cell survival was monitored by MTT assay. Figure S2. Co-transfection of pCDH.GFP.AGO2 with pNUT.CRBN.His yielded methotrexate (MTX)-resistant cells that express high levels of GFP. Pictures were taken from MTX-selected BHK cells that were transfected with the mixed DNAs containing pCDH.GFP.AGO2 and pNUT.CRBN.His. Figure S3. AGO2 expression is associated with cell growth. Cell lysates were harvested from My5.LV without any treatment (−), treated with dimethyl sulfoxide (DMSO) or 10 μM lenalidomide (LEN) and analyzed by western blot (probed with AGO2-specific antibody). Figure S4. The growth rate of the MM cells with higher levels of CRBN is lower than the cells with low levels of CRBN. Cell survival, after infection of My5.LV or My5.CRBN.His cells with viral particles containing pCDH vector (A), AGO2 cDNA (B), pLKO.1 vector (C) or AGO2-shRNA-72 (D), was followed by MTT assay. Figure S5. Silencing of AGO2 with its shRNA or over-expression of AGO2 altered the steady-state levels of miRNAs. Total RNAs were isolated after AGO-shRNA72 (sh72) treatment for 3 days or over-expression of AGO2 and the steady-state levels of miRNAs were analyzed with microRNA array kit. Scatter plots (a), heat-maps (b) and up- or down-regulated miRNAs (c) were established by comparison of the steady-state levels of miRNAs between two samples listed in each plot. Figure S6. Treatment of MM cells with lenalidomide altered steady-state levels of miRNAs. Total RNAs were isolated after lenalidomide treatment for 3 or 5 days and the steady-state levels of miRNAs were analyzed with microRNA array kit. Scatter plots (a), heat-maps (b) and up- or down-regulated miRNAs (c) were established by comparison of the steady-state levels of miRNAs between two samples listed in each plot. DOCX 5669 kb) [file 12885_2016_2331_MOESM1_ESM.docx]

Figure S1. Lenalidomide induced cell death is a slow process. Cellsurvival was monitored byMTT assay.


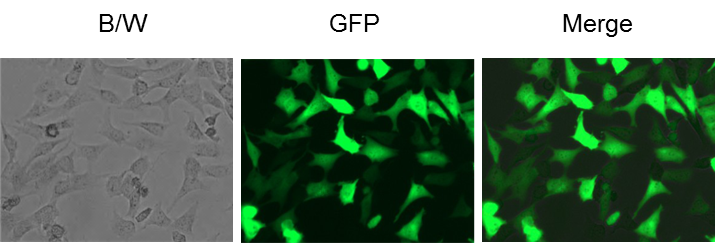


Figure S2. Co-transfection of pCDH.AGO2 with pNUT.CRBN.Hisyielded MTX-resistant cells that express high levels of GFP. Pictures were taken from MTX-selected BHK cells that were transfected with the mixed DNAs containing pCDH.AGO2 and pNUT.CRBN.His.


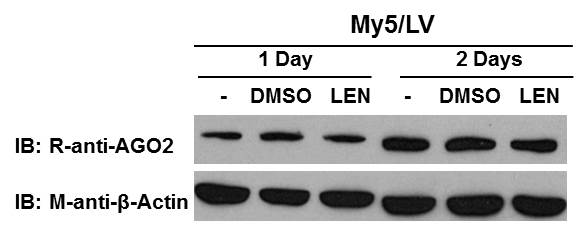


Figure S3. AGO2 expression is associated with cell growth. Cell lysates were harvested from My5.LV without any treatment (-), treated with DMSO (DMSO) or 10 µM lenalidomide (LEN).

**B**

**A**

**D**

**C**

**Figure S4. The growth rate of the MM cells with higher levels of CRBN is lower than the cells with low levels of CRBN.** Cell survival, after infection of My5.LV or My5.CRBN.His cells with viral particles containing pCDH vector (A),AGO2 cDNA (B), pLKO.1 vector (C) or AGO2-shRNA-72 (D), was followed by MTT assay.

**Figure S5. Silencing of AGO2 with its shRNA altered the steady-state levels of miRNAs.** Total RNAs were isolated after AGO-shRNA72 (sh72) treatment for 3 days and the steady-state levels of miRNAs were analyzed with microRNA array kit. Scatter plots (+/- 3 fold cut-off) and heat-maps were established by comparison of the steady-state levels of miRNAs between two samples listed in each plot.

**Figure S5A. Scatter plot (a) and heat-map (b) of My5.LV vs My5.CRBN & the miRNAs up- or down-regulated in this pair [c]**


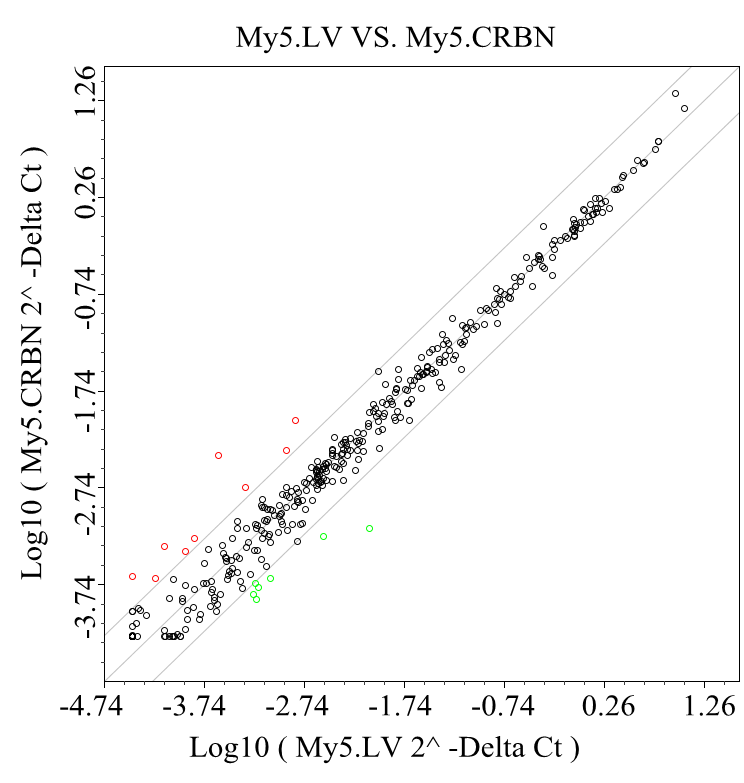

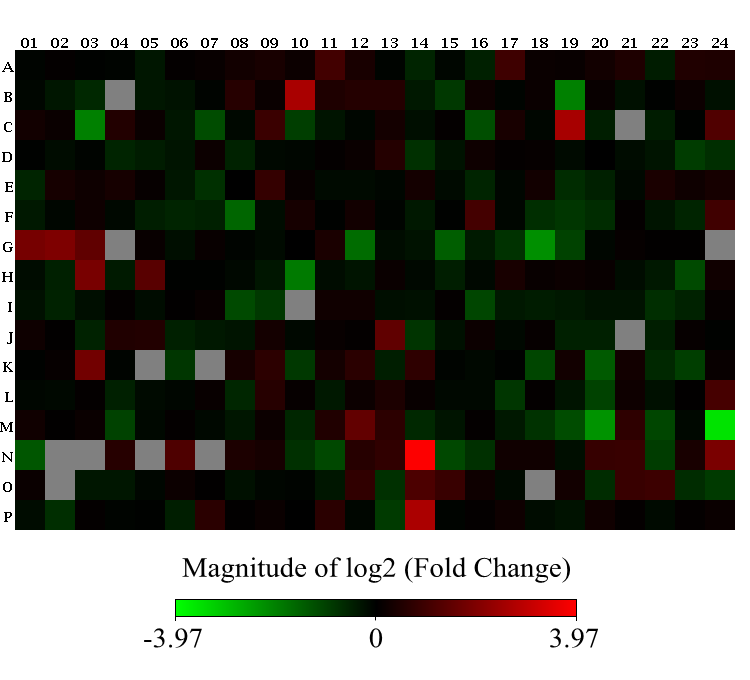


| Arrays included in Test Group: | | My5.CRBN |
| --- | --- | --- |
| Arrays included in Control Group: | | My5.LV |
| Genes Over-Expressed | | |
| Position | Mature ID | Fold Regulation |
| B10 | hsa-miR-125a-5p | 6.2607 |
| C19 | hsa-miR-143-3p | 6.0765 |
| G01 | hsa-miR-205-3p | 3.5621 |
| G02 | hsa-miR-206 | 3.8703 |
| H03 | hsa-miR-223-3p | 3.6185 |
| K03 | hsa-miR-34a-3p | 3.4324 |
| N14 | hsa-miR-551b-3p | 15.6734 |
| N24 | hsa-miR-600 | 3.7027 |
| P14 | cel-miR-39-3p | 6.4227 |
| Genes Under-Expressed | | |
| Position | Mature ID | Fold Regulation |
| B19 | hsa-miR-129-1-3p | -3.9908 |
| C03 | hsa-miR-1324 | -3.9733 |
| G12 | hsa-miR-211-5p | -3.2056 |
| G18 | hsa-miR-218-5p | -4.7115 |
| H10 | hsa-miR-23b-5p | -3.7036 |
| M20 | hsa-miR-508-5p | -4.9444 |
| M24 | hsa-miR-513a-5p | -11.652 |

**Figure S5B. Scatter plot (a) and heat-map (b) of My5.LV vs My5.LV.AGO2& the miRNAs up- or down-regulated in this pair [c]**

(c)

(b)

(a)

**Figure S5C. Scatter plot (a) and heat-map (b) of My5.LV vs My5.LV.sh72 & the miRNAs up- ordown-regulated in this pair [c]**

(c)

(b)

(a)


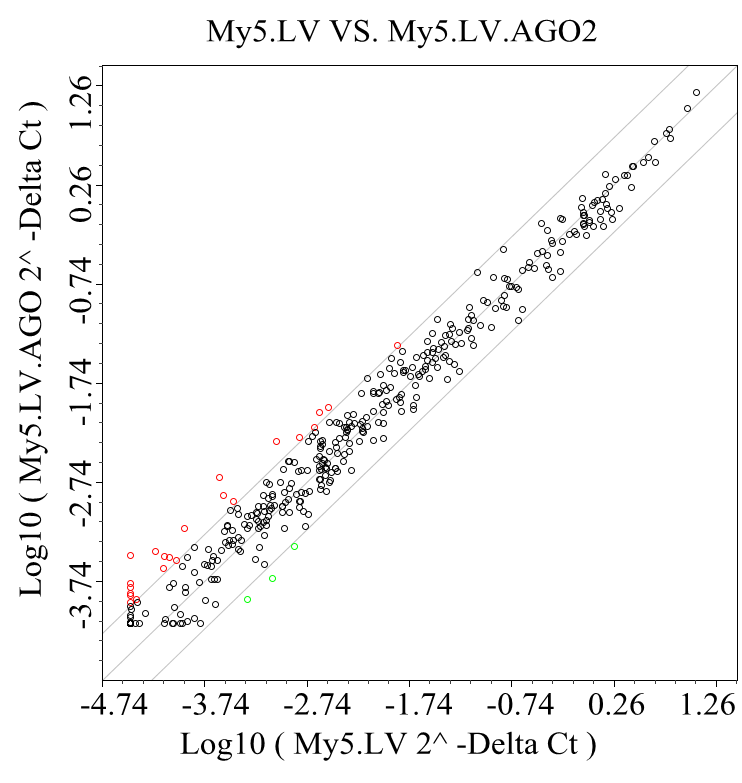

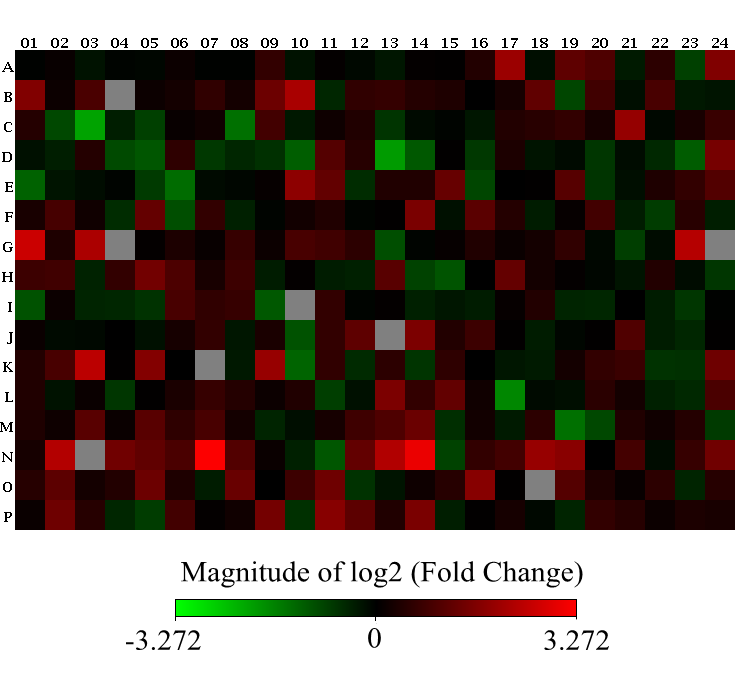


| Arrays included in Test Group: | | My5.LV.AGO2 |
| --- | --- | --- |
| Arrays included in Control Group: | | My5.LV |
| Genes Over-Expressed | | |
| Position | Mature ID | Fold Regulation |
| A17 | hsa-miR-106a-3p | 3.9942 |
| A24 | hsa-miR-10b-3p | 3.1007 |
| B01 | hsa-miR-1180-3p | 3.1521 |
| B10 | hsa-miR-125a-5p | 4.4954 |
| C21 | hsa-miR-144-3p | 3.7899 |
| E10 | hsa-miR-187-3p | 3.5153 |
| G01 | hsa-miR-205-3p | 6.113 |
| G03 | hsa-miR-208a-3p | 4.6061 |
| G23 | hsa-miR-221-3p | 5.0678 |
| J14 | hsa-miR-338-3p | 3.0268 |
| K03 | hsa-miR-34a-3p | 5.3466 |
| K05 | hsa-miR-34b-5p | 3.2544 |
| K09 | hsa-miR-363-3p | 3.8905 |
| L13 | hsa-miR-424-3p | 3.0002 |
| N02 | hsa-miR-517b-3p | 4.9876 |
| N07 | hsa-miR-522-3p | 9.658 |
| N13 | hsa-miR-549a | 4.8667 |
| N14 | hsa-miR-551b-3p | 8.1141 |
| N18 | hsa-miR-575 | 3.8495 |
| N19 | hsa-miR-580-3p | 3.4299 |
| O16 | hsa-miR-765 | 3.3744 |
| P11 | hsa-miR-99a-3p | 3.3345 |
| Genes Under-Expressed | | |
| Position | Mature ID | Fold Regulation |
| C03 | hsa-miR-1324 | -4.3338 |
| D13 | hsa-miR-155-5p | -4.0026 |
| L17 | hsa-miR-429 | -3.3652 |

(c)

(b)

(a)


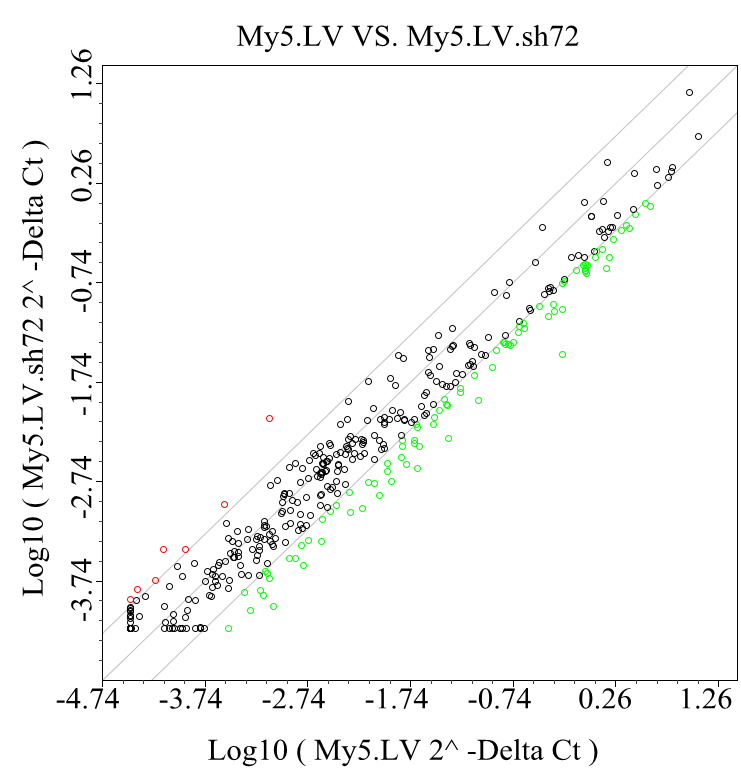

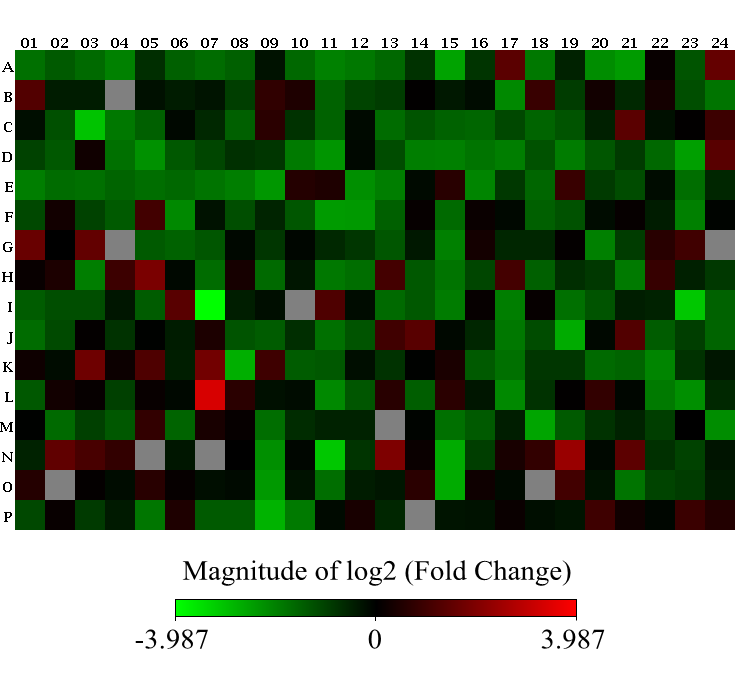


| Arrays included in Test Group: | | | | My.LV.sh72 | |
| --- | --- | --- | --- | --- | --- |
| Arrays included in Control Group: | | | | My.LV | |
| Genes Over-Expressed | | | | | |
| Position | Mature ID | | | Fold Regulation | |
| G01 | hsa-miR-205-3p | | | 3.0845 | |
| H05 | hsa-miR-224-5p | | | 3.7744 | |
| K03 | hsa-miR-34a-3p | | | 3.2603 | |
| K07 | hsa-miR-34c-5p | | | 3.4564 | |
| L07 | hsa-miR-410-3p | | | 10.3617 | |
| N13 | hsa-miR-549a | | | 3.8853 | |
| N19 | hsa-miR-580-3p | | | 5.276 | |
| Genes Under-Expressed | | | | | |
| Position | Mature ID | | | Fold Regulation | |
| A01 | hsa-let-7a-5p | | | -3.3906 | |
| A03 | hsa-let-7c-5p | | | -3.164 | |
| A04 | hsa-let-7d-5p | | | -4.1279 | |
| A07 | hsa-let-7f-5p | | | -3.2217 | |
| A10 | hsa-let-7i-5p | | | -3.0905 | |
| A11 | hsa-miR-1-3p | | | -4.0832 | |
| A12 | hsa-miR-100-5p | | | -3.6901 | |
| A13 | hsa-miR-101-3p | | | -3.0841 | |
| A15 | hsa-miR-103a-3p | | | -5.8615 | |
| A18 | hsa-miR-106b-5p | | | -3.6531 | |
| A20 | hsa-miR-107 | | | -4.631 | |
| A21 | hsa-miR-10a-5p | | | -5.4597 | |
| B17 | hsa-miR-128-3p | | | -4.449 | |
| B24 | hsa-miR-130b-3p | | | -3.5355 | |
| C03 | hsa-miR-1324 | | | -8.375 | |
| C04 | hsa-miR-133a-3p | | | -3.6572 | |
| C13 | hsa-miR-139-5p | | | -3.1973 | |
| C16 | hsa-miR-141-3p | | | -3.0035 | |
| D04 | hsa-miR-148a-3p | | -3.3407 | | |
| D05 | hsa-miR-148b-3p | | -4.8948 | | |
| D10 | hsa-miR-152-3p | | -3.7115 | | |
| D11 | hsa-miR-153-3p | | -5.0276 | | |
| D14 | hsa-miR-155-3p | | -3.9519 | | |
| D15 | hsa-miR-15a-5p | | -4.0306 | | |
| D16 | hsa-miR-15b-5p | | -3.521 | | |
| D17 | hsa-miR-15b-3p | | -3.903 | | |
| D19 | hsa-miR-17-5p | | -3.8654 | | |
| D22 | hsa-miR-181b-5p | | -3.0655 | | |
| D23 | hsa-miR-181c-5p | | -5.6353 | | |
| E01 | hsa-miR-181d-5p | | -3.8853 | | |
| E02 | hsa-miR-182-5p | | -3.2223 | | |
| E03 | hsa-miR-182-3p | | -3.3803 | | |
| E05 | hsa-miR-183-3p | | -3.2957 | | |
| E06 | hsa-miR-184 | | -3.0858 | | |
| E07 | hsa-miR-185-5p | | -3.5268 | | |
| E08 | hsa-miR-186-5p | | -3.9576 | | |
| E09 | hsa-miR-186-3p | | -5.2367 | | |
| E12 | hsa-miR-18a-5p | | -4.769 | | |
| E13 | hsa-miR-18a-3p | | -3.9023 | | |
| E16 | hsa-miR-190a-5p | | -4.2406 | | |
| E23 | hsa-miR-193a-3p | | -3.2807 | | |
| F06 | hsa-miR-196a-5p | | -4.4451 | | |
| F11 | hsa-miR-199a-5p | | -5.4729 | | |
| F12 | hsa-miR-199b-5p | | -5.3216 | | |
| F15 | hsa-miR-19b-3p | | -3.1235 | | |
| F23 | hsa-miR-204-5p | | -3.9762 | | |
| G15 | hsa-miR-215-5p | | -3.9703 | | |
| G20 | hsa-miR-219a-5p | | -4.0325 | | |
| H03 | hsa-miR-223-3p | | -3.9517 | | |
| H07 | hsa-miR-23a-3p | | -3.2577 | | |
| H09 | hsa-miR-23b-3p | | -3.1525 | | |
| H11 | hsa-miR-24-3p | | -3.6516 | | |
| H12 | hsa-miR-25-3p | | -3.3057 | | |
| H15 | hsa-miR-26b-5p | | -3.5155 | | |
| H21 | hsa-miR-28-5p | | -3.7724 | | |
| I07 | hsa-miR-301a-3p | | -15.8543 | | |
| I13 | hsa-miR-30a-5p | | -3.1541 | | |
| I15 | hsa-miR-30c-5p | | -3.8383 | | |
| I17 | hsa-miR-30d-5p | | -3.883 | | |
| I19 | hsa-miR-30e-5p | | -3.3829 | | |
| I23 | hsa-miR-32-5p | | -8.7391 | | |
| J01 | hsa-miR-320a | | -3.2339 | | |
| J11 | hsa-miR-335-5p | | -3.3363 | | |
| J17 | hsa-miR-33a-5p | | -3.642 | | |
| J19 | hsa-miR-33b-5p | | -6.4714 | | |
| K08 | hsa-miR-361-5p | | -6.7559 | | |
| K17 | hsa-miR-374c-5p | | -3.3476 | | |
| K20 | hsa-miR-376b-3p | | -3.1809 | | |
| K22 | hsa-miR-377-3p | | -4.2991 | | |
| L11 | hsa-miR-423-5p | | -4.4987 | | |
| L17 | hsa-miR-429 | | -4.4763 | | |
| L22 | hsa-miR-451a | -3.7479 | | |  |
| L23 | hsa-miR-454-3p | -4.7829 | | |  |
| M02 | hsa-miR-484 | -3.1421 | | |  |
| M09 | hsa-miR-491-5p | -3.3165 | | |  |
| M15 | hsa-miR-499a-5p | -3.3334 | | |  |
| M18 | hsa-miR-505-3p | -6.067 | | |  |
| M24 | hsa-miR-513a-5p | -4.6889 | | |  |
| N09 | hsa-miR-532-5p | -4.7216 | | |  |
| N11 | hsa-miR-542-3p | -8.7085 | | |  |
| N15 | hsa-miR-567 | -6.4256 | | |  |
| O09 | hsa-miR-652-3p | -5.3618 | | |  |
| O11 | hsa-miR-7-5p | -3.2705 | | |  |
| O15 | hsa-miR-744-5p | -6.494 | | |  |
| O21 | hsa-miR-9-5p | -3.5184 | | |  |
| P05 | hsa-miR-93-5p | -3.56 | | |  |
| P09 | hsa-miR-98-5p | -7.0753 | | |  |
| P10 | hsa-miR-99a-5p | -3.7216 | | |  |

**Figure S5D. Scatter plot (a) and heat-map (b) of My5.LV.AGO2 vs My5.LV.sh72 & the miRNAs up- or down-regulated in this pair [c]**

(c)

(b)

(a)


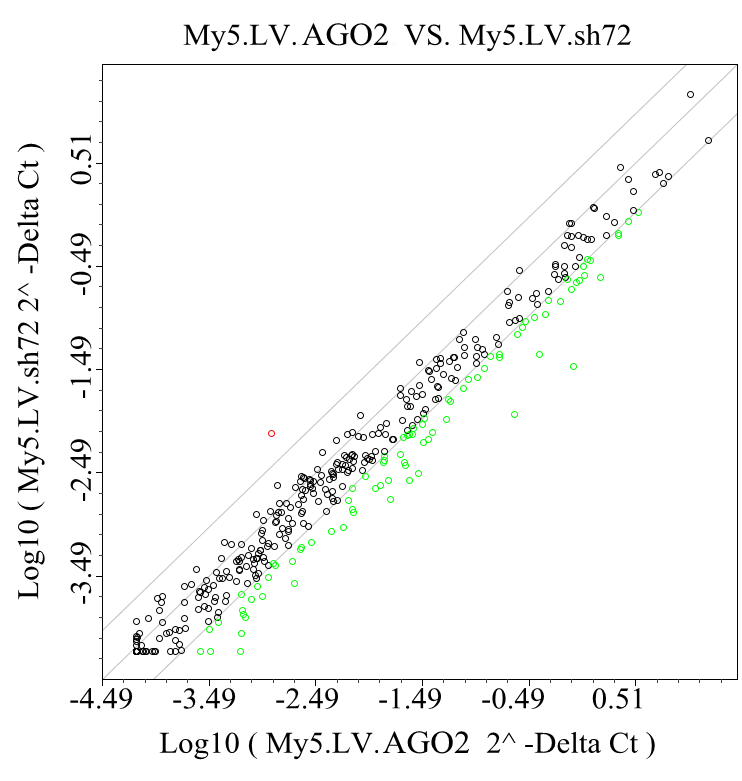

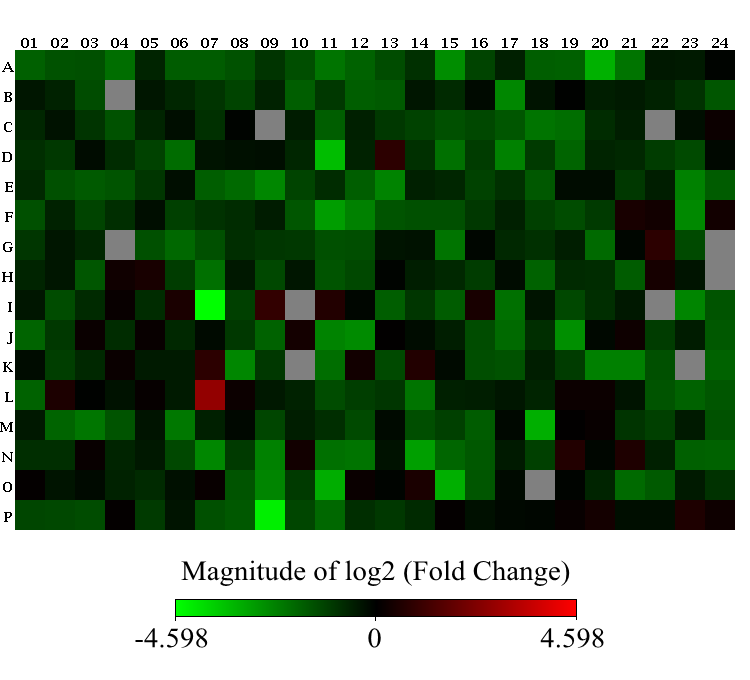


| Arrays included in Test Group: | | | | | | My5.LV. sh72 | | |
| --- | --- | --- | --- | --- | --- | --- | --- | --- |
| Arrays included in Control Group: | | | | | | My5.LV. AGO2 | | |
| Genes Over-Expressed | | | | | | | | |
| Position | | | Mature ID | | | Fold Regulation | | |
| L07 | | | hsa-miR-410-3p | | | 6.3572 | | |
| Genes Under-Expressed | | | | | | | | |
| Position | | | Mature ID | | | Fold Regulation | | |
| A01 | | | hsa-let-7a-5p | | | -3.3325 | | |
| A04 | | | hsa-let-7d-5p | | | -3.9797 | | |
| A06 | | | hsa-let-7e-5p | | | -3.1292 | | |
| A07 | | | hsa-let-7f-5p | | | -3.1903 | | |
| A11 | | | hsa-miR-1-3p | | | -4.2192 | | |
| A12 | | | hsa-miR-100-5p | | | -3.4114 | | |
| A15 | | | hsa-miR-103a-3p | | | -5.9698 | | |
| A18 | | | hsa-miR-106b-5p | | | -3.2013 | | |
| A19 | | | hsa-miR-106b-3p | | | -3.3507 | | |
| A20 | | | hsa-miR-107 | | | -9.3352 | | |
| A21 | | | hsa-miR-10a-5p | | | -4.2987 | | |
| B10 | | | hsa-miR-125a-5p | | | -3.26 | | |
| B12 | | | hsa-miR-126-3p | | | -3.2114 | | |
| B13 | | | hsa-miR-126-5p | | | -3.057 | | |
| B17 | | | hsa-miR-128-3p | | | -5.4151 | | |
| C11 | | | hsa-miR-138-5p | | | -3.2745 | | |
| C18 | | | hsa-miR-142-5p | | | -4.2113 | | |
| C19 | | | hsa-miR-143-3p | | | -3.9359 | | |
| D06 | | | hsa-miR-149-5p | | | -3.8624 | | |
| D11 | | | hsa-miR-153-3p | | | -10.6818 | | |
| D15 | | | hsa-miR-15a-5p | | | -4.0708 | | |
| D17 | | | hsa-miR-15b-3p | | | -5.0309 | | |
| D19 | | | | hsa-miR-17-5p | | -3.5152 | | |
| E03 | | | | | | hsa-miR-182-3p | -3.0609 | |
| E07 | | | | | | hsa-miR-185-5p | -3.2027 | |
| E08 | | | | | | hsa-miR-186-5p | -3.7396 | |
| E09 | | | | | | hsa-miR-186-3p | -5.524 | |
| E12 | | | | | | hsa-miR-18a-5p | -3.2172 | |
| E13 | | | | | | hsa-miR-18a-3p | -5.1598 | |
| E18 | | | | | | hsa-miR-191-5p | -3.0196 | |
| E23 | | | | | | hsa-miR-193a-3p | -5.0945 | |
| E24 | | | | | | hsa-miR-193a-5p | -3.1511 | |
| F11 | | | | | | hsa-miR-199a-5p | -7.2282 | |
| F12 | | | | | | hsa-miR-199b-5p | -5.102 | |
| F23 | | | | | | hsa-miR-204-5p | -5.6323 | |
| G06 | | | | | | hsa-miR-20a-3p | -3.6709 | |
| G15 | | | | | | hsa-miR-215-5p | -4.2226 | |
| G20 | | | | | | hsa-miR-219a-5p | -3.7566 | |
| H07 | | | | | | hsa-miR-23a-3p | -4.0539 | |
| H18 | | | | | | hsa-miR-27b-3p | -3.3746 | |
| H21 | | | | | | hsa-miR-28-5p | -3.1693 | |
| I07 | | | | | | hsa-miR-301a-3p | -24.2217 | |
| I13 | | | | | | hsa-miR-30a-5p | -3.2768 | |
| I15 | | | | | | hsa-miR-30c-5p | -3.1327 | |
| I17 | | | | | | hsa-miR-30d-5p | -4.0974 | |
| I23 | | | | | | hsa-miR-32-5p | -5.3659 | |
| J01 | | | | | | hsa-miR-320a | -3.5269 | |
| J09 | | | | | | hsa-miR-330-3p | -3.4288 | |
| J11 | | | | | | hsa-miR-335-5p | -5.2501 | |
| J12 | | | | | | hsa-miR-335-3p | -5.8106 | |
| J17 | | | | | | hsa-miR-33a-5p | -3.7216 | |
| J19 | | | | | | hsa-miR-33b-5p | -6.1028 | |
| J24 | | | | | | hsa-miR-345-5p | -3.0277 | |
| K08 | | | | | | hsa-miR-361-5p | -5.4824 | |
| K11 | | | | | | hsa-miR-365b-3p | -3.9094 | |
| K20 | | | | | | hsa-miR-376b-3p | -4.9765 | |
| K21 | | | | | | hsa-miR-376c-3p | -4.9364 | |
| K24 | | | | | | hsa-miR-378a-3p | -3.3984 | |
| L01 | | | | | | hsa-miR-378a-5p | -3.4306 | |
| L14 | | | | | | hsa-miR-425-5p | -4.2922 | |
| L23 | | | | | | hsa-miR-454-3p | -3.3653 | |
| M02 | | | | | | hsa-miR-484 | -3.5337 | |
| M03 | | | | | | hsa-miR-485-5p | -4.4025 | |
| M06 | | | | | | hsa-miR-488-3p | -4.4457 | |
| M16 | | | | | | hsa-miR-500a-5p | -3.1401 | |
| M18 | | | | | | hsa-miR-505-3p | -8.9557 | |
| N07 | | | | | | hsa-miR-522-3p | -5.4891 | |
| N09 | | | | | | hsa-miR-532-5p | -5.1331 | |
| N11 | hsa-miR-542-3p | | | | -4.0773 |  |  |  |
| N12 | hsa-miR-542-5p | | | | -4.2776 |  |  |  |
| N14 | hsa-miR-551b-3p | | | | -7.3061 |  |  |  |
| N15 | hsa-miR-567 | | | | -3.5904 |  |  |  |
| N16 | hsa-miR-570-3p | | | | -3.0393 |  |  |  |
| N23 | hsa-miR-589-3p | | | | -3.3454 |  |  |  |
| N24 | hsa-miR-600 | | | | -3.4221 |  |  |  |
| O09 | hsa-miR-652-3p | | | | -5.3475 |  |  |  |
| O11 | hsa-miR-7-5p | | | | -8.7757 |  |  |  |
| O15 | hsa-miR-744-5p | | | | -9.046 |  |  |  |
| O21 | hsa-miR-9-5p | | | | -3.7595 |  |  |  |
| O22 | hsa-miR-9-3p | | | | -3.0983 |  |  |  |
| P08 | hsa-miR-96-5p | | | | -3.0128 |  |  |  |
| P09 | hsa-miR-98-5p | | | | -19.9047 |  |  |  |
| P11 | hsa-miR-99a-3p | | | | -3.6868 |  |  |  |

|  |
| --- |

Figure S5E. Scatter plot (a) and heat-map (b) of My5.CRBN vs My5.CRBN.sh72 & the miRNAs up- or down-regulated in this pair [c]

(c)

(b)

(a)


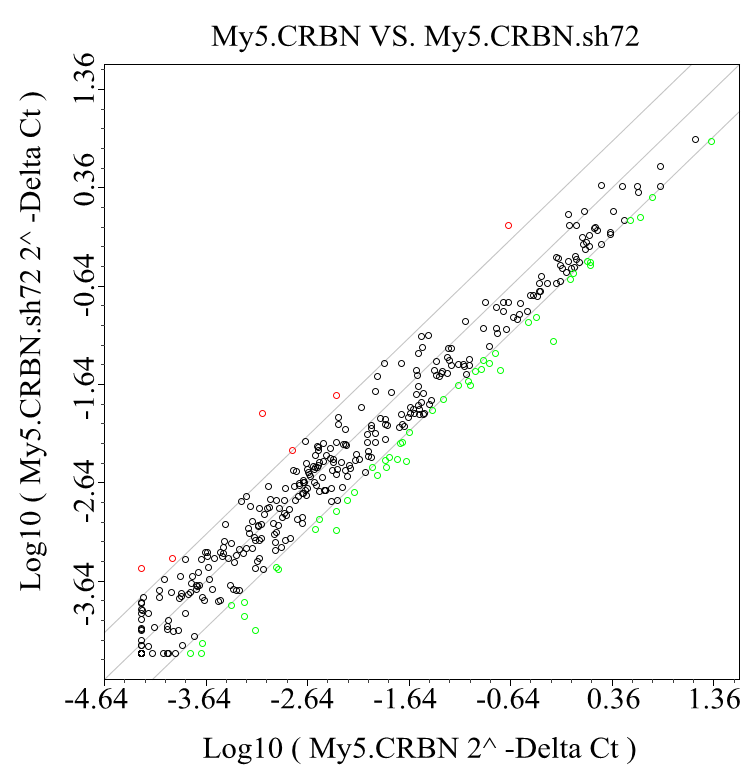

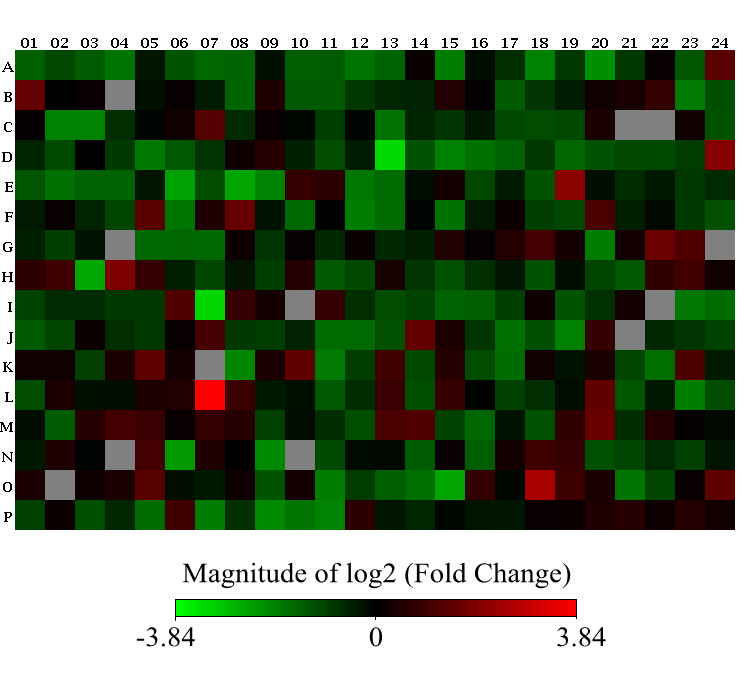


| Arrays included in Test Group: | | My5.CRBN.sh72 |
| --- | --- | --- |
| Arrays included in Control Group: | | My5.CRBN |
| Genes Over-Expressed | | |
| Position | Mature ID | Fold Regulation |
| D24 | hsa-miR-181c-3p | 4.0098 |
| E19 | hsa-miR-191-3p | 4.2872 |
| G22 | hsa-miR-22-5p | 3.0318 |
| H04 | hsa-miR-223-5p | 3.6648 |
| L07 | hsa-miR-410-3p | 14.3165 |
| O18 | hsa-miR-802 | 5.7302 |
| Genes Under-Expressed | | |
| Position | Mature ID | Fold Regulation |
| A04 | hsa-let-7d-5p | -3.3694 |
| A12 | hsa-miR-100-5p | -3.3357 |
| A15 | hsa-miR-103a-3p | -3.7582 |
| A18 | hsa-miR-106b-5p | -3.8642 |
| A20 | hsa-miR-107 | -4.4718 |
| B23 | hsa-miR-130a-3p | -3.5749 |
| C02 | hsa-miR-132-5p | -3.8893 |
| C03 | hsa-miR-1324 | -3.937 |
| C13 | hsa-miR-139-5p | -3.2719 |
| D05 | hsa-miR-148b-3p | -3.5025 |
| D13 | hsa-miR-155-5p | -9.6464 |
| D15 | hsa-miR-15a-5p | -3.7942 |
| D16 | hsa-miR-15b-5p | -3.2481 |
| E02 | hsa-miR-182-5p | -3.1225 |
| E06 | hsa-miR-184 | -5.4112 |
| E08 | hsa-miR-186-5p | -5.7071 |
| E09 | hsa-miR-186-3p | -3.9598 |
| E12 | hsa-miR-18a-5p | -3.523 |
| E13 | hsa-miR-18a-3p | -3.1063 |
| F06 | hsa-miR-196a-5p | -3.4613 |
| F12 | hsa-miR-199b-5p | -3.7429 |
| F13 | hsa-miR-19a-3p | -3.1184 |
| F15 | hsa-miR-19b-3p | -3.2105 |
| G20 | hsa-miR-219a-5p | -3.6498 |
| H03 | hsa-miR-223-3p | -5.8251 |
| I07 | hsa-miR-301a-3p | -9.5336 |
| I23 | hsa-miR-32-5p | -3.5257 |
| I24 | hsa-miR-32-3p | -3.1039 |
| J11 | hsa-miR-335-5p | -3.1181 |
| J12 | hsa-miR-335-3p | -3.0056 |
| J17 | hsa-miR-33a-5p | -3.1608 |
| J19 | hsa-miR-33b-5p | -3.7958 |
| K08 | hsa-miR-361-5p | -4.1589 |
| K11 | hsa-miR-365b-3p | -3.5589 |
| K17 | hsa-miR-374c-5p | -3.0946 |
| K22 | hsa-miR-377-3p | -3.146 |
| L23 | hsa-miR-454-3p | -3.7446 |
| N06 | hsa-miR-520g-3p | -4.9587 |
| N09 | hsa-miR-532-5p | -4.1859 |
| O11 | hsa-miR-7-5p | -3.6143 |
| O14 | hsa-miR-720 | -3.171 |
| O15 | hsa-miR-744-5p | -5.7103 |
| O21 | hsa-miR-9-5p | -3.3341 |
| P05 | hsa-miR-93-5p | -3.1146 |
| P07 | hsa-miR-95-3p | -3.6379 |
| P09 | hsa-miR-98-5p | -4.1841 |
| P10 | hsa-miR-99a-5p | -3.4272 |
| P11 | hsa-miR-99a-3p | -3.9321 |

Figure S5F. Scatter plot (a) and heat-map (b) of My5.CRBN vs My5.LV.sh72 & the miRNAs up- or down-regulated in this pair [c]

(c)

(b)

(a)


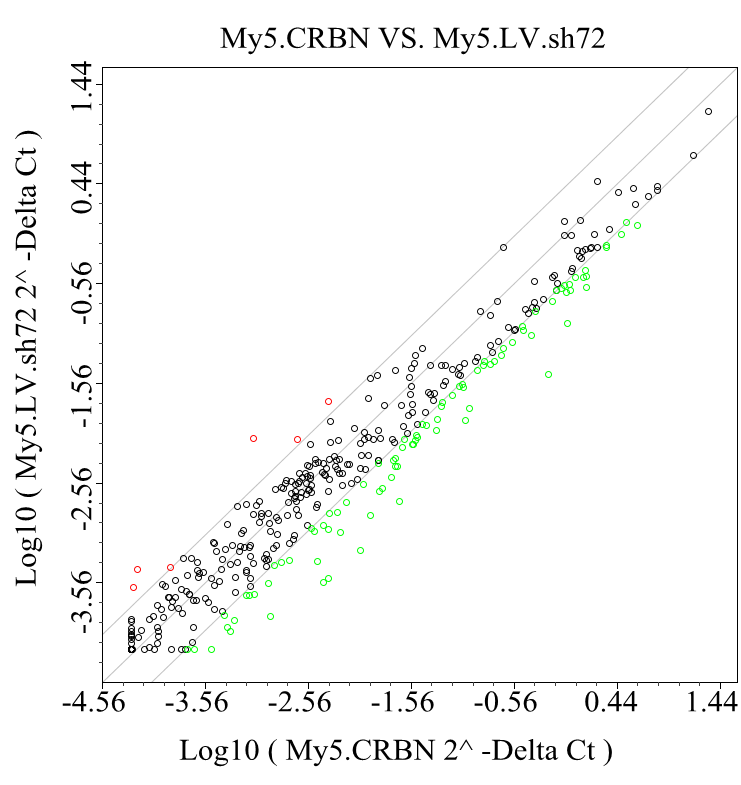

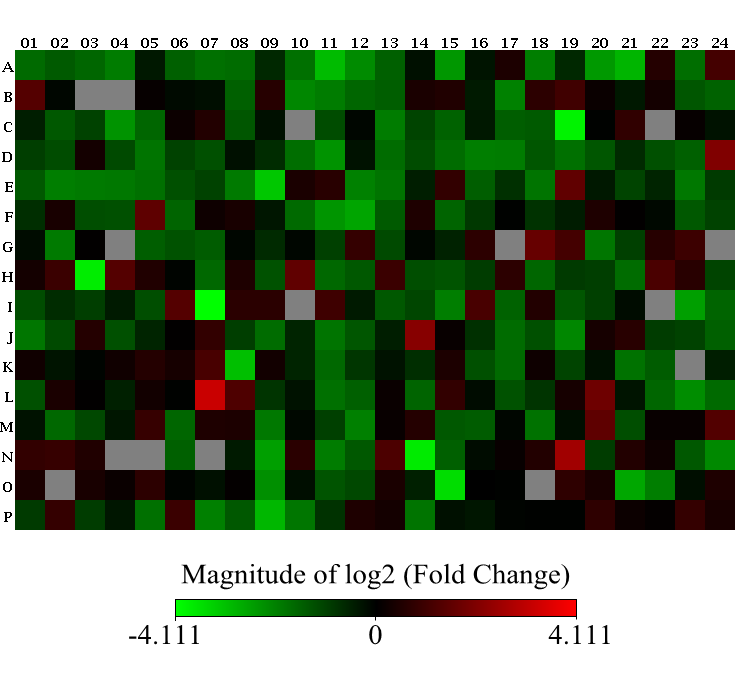


| Arrays included in Test Group: | | My5.LV.sh72 |  |  |
| --- | --- | --- | --- | --- |
| Arrays included in Control Group: | | My5.CRBN |  |  |
| Genes Over-Expressed | | |  |  |
| Position | Mature ID | Fold Regulation |  |  |
| D24 | hsa-miR-181c-3p | 4.1913 |  |  |
| G18 | hsa-miR-218-5p | 3.0952 |  |  |
| J14 | hsa-miR-338-3p | 4.5403 |  |  |
| L07 | hsa-miR-410-3p | 9.5995 |  |  |
| L20 | hsa-miR-433-3p | 3.4532 |  |  |
| N19 | hsa-miR-580-3p | 6.1305 |  |  |
| Genes Under-Expressed | | |  |  |
| Position | Mature ID | Fold Regulation |  |  |
| A01 | hsa-let-7a-5p | -3.245 |  |  |
| A03 | hsa-let-7c-5p | -3.122 |  |  |
| A04 | hsa-let-7d-5p | -3.9579 |  |  |
| A07 | hsa-let-7f-5p | -3.4771 |  |  |
| A08 | hsa-let-7g-5p | -3.366 |  |  |
| A10 | hsa-let-7i-5p | -3.5052 |  |  |
| A11 | hsa-miR-1-3p | -8.2463 |  |  |
| A12 | hsa-miR-100-5p | -4.7698 |  |  |
| A15 | hsa-miR-103a-3p | -5.509 |  |  |
| A18 | hsa-miR-106b-5p | -4.0821 |  |  |
| A20 | hsa-miR-107 | -5.5945 |  |  |
| A21 | hsa-miR-10a-5p | -7.5964 |  |  |
| A23 | hsa-miR-10b-5p | -3.4452 |  |  |
| B10 | hsa-miR-125a-5p | -4.5402 |  |  |
| B11 | hsa-miR-125b-5p | -3.9753 |  |  |
| B12 | hsa-miR-126-3p | -3.11 |  |  |
| B17 | hsa-miR-128-3p | -4.2613 |  |  |
| C04 | hsa-miR-133a-3p | -5.2225 |  |  |
| C05 | hsa-miR-133b | -3.1375 | |  |
| C13 | hsa-miR-139-5p | -3.955 | |  |
| C19 | hsa-miR-143-3p | -15.2565 | |  |
| D05 | hsa-miR-148b-3p | -3.6249 | |  |
| D10 | hsa-miR-152-3p | -3.4517 | |  |
| D11 | hsa-miR-153-3p | -5.277 | |  |
| D13 | hsa-miR-155-5p | -3.3624 | |  |
| D15 | hsa-miR-15a-5p | -3.3279 | |  |
| D16 | hsa-miR-15b-5p | -4.0592 | |  |
| D17 | hsa-miR-15b-3p | -4.0388 | |  |
| D19 | hsa-miR-17-5p | -3.4663 | |  |
| E02 | hsa-miR-182-5p | -4.1279 | |  |
| E03 | hsa-miR-182-3p | -3.8947 | |  |
| E04 | hsa-miR-183-5p | -3.7885 | |  |
| E05 | hsa-miR-183-3p | -3.5015 | |  |
| E08 | hsa-miR-186-5p | -3.9226 | |  |
| E09 | hsa-miR-186-3p | -9.2511 | |  |
| E12 | hsa-miR-18a-5p | -4.2724 | |  |
| E13 | hsa-miR-18a-3p | -3.671 | |  |
| E18 | hsa-miR-191-5p | -3.6256 | |  |
| E23 | hsa-miR-193a-3p | -3.7988 | |  |
| F06 | hsa-miR-196a-5p | -3.03 | |  |
| F10 | hsa-miR-199b-3p | -3.2529 | |  |
| F11 | hsa-miR-199a-5p | -5.4029 | |  |
| F12 | hsa-miR-199b-5p | -6.4469 | |  |
| F15 | hsa-miR-19b-3p | -3.0259 | |  |
| G02 | hsa-miR-206 | -3.8862 | |  |
| G20 | hsa-miR-219a-5p | -3.7418 | |  |
| H03 | hsa-miR-223-3p | -14.2994 | |  |
| H07 | hsa-miR-23a-3p | -3.177 | |  |
| H11 | hsa-miR-24-3p | -3.211 | |  |
| H18 | hsa-miR-27b-3p | -3.1225 | |  |
| H21 | hsa-miR-28-5p | -3.3466 | |  |
| I07 | hsa-miR-301a-3p | -17.2806 | |  |
| I15 | hsa-miR-30c-5p | -4.0448 | |  |
| I17 | hsa-miR-30d-5p | -3.0136 | |  |
| I23 | hsa-miR-32-5p | -6.0079 | |  |
| I24 | hsa-miR-32-3p | -3.0453 | |  |
| J01 | hsa-miR-320a | -3.7743 | |  |
| J09 | hsa-miR-330-3p | -3.3409 | |  |
| J11 | hsa-miR-335-5p | -3.6501 | |  |
| J17 | hsa-miR-33a-5p | -3.3185 | |  |
| J19 | hsa-miR-33b-5p | -4.5678 | |  |
| K08 | hsa-miR-361-5p | -8.4846 | |  |
| K11 | hsa-miR-365b-3p | -3.2063 | |  |
| K17 | hsa-miR-374c-5p | -3.2792 | |  |
| K21 | hsa-miR-376c-3p | -3.5707 | | |
| L11 | hsa-miR-423-5p | -3.4852 | | |
| L14 | hsa-miR-425-5p | -3.0331 | | |
| L22 | hsa-miR-451a | -3.1323 | | |
| L23 | hsa-miR-454-3p | -4.9241 | | |
| L24 | hsa-miR-455-5p | -3.2641 | | |
| M02 | hsa-miR-484 | -3.2199 | | |
| M06 | hsa-miR-488-3p | -3.0937 | | |
| M09 | hsa-miR-491-5p | -3.7814 | | |
| M12 | hsa-miR-497-5p | -4.1767 | | |
| M18 | hsa-miR-505-3p | -3.5552 | | |
| N09 | hsa-miR-532-5p | -5.9775 | | |
| N11 | hsa-miR-542-3p | -4.0193 | | |
| N14 | hsa-miR-551b-3p | -14.1126 | | |
| N24 | hsa-miR-600 | -4.6449 | | |
| O09 | hsa-miR-652-3p | -4.9858 | | |
| O15 | hsa-miR-744-5p | -11.8635 | | |
| O21 | hsa-miR-9-5p | -6.4811 | | |
| O22 | hsa-miR-9-3p | -4.0536 | | |
| P05 | hsa-miR-93-5p | -3.4895 | | |
| P07 | hsa-miR-95-3p | -4.1513 | | |
| P09 | hsa-miR-98-5p | -7.806 | | |
| P10 | hsa-miR-99a-5p | -3.7419 | | |
| P14 | cel-miR-39-3p | -3.6503 | | |

Figure S5G. Scatter plot (a) and heat-map (b) of My5.LV vs My5.CRBN.sh72 & the miRNAs up- or down-regulated in this pair [c]

(b)

(a)


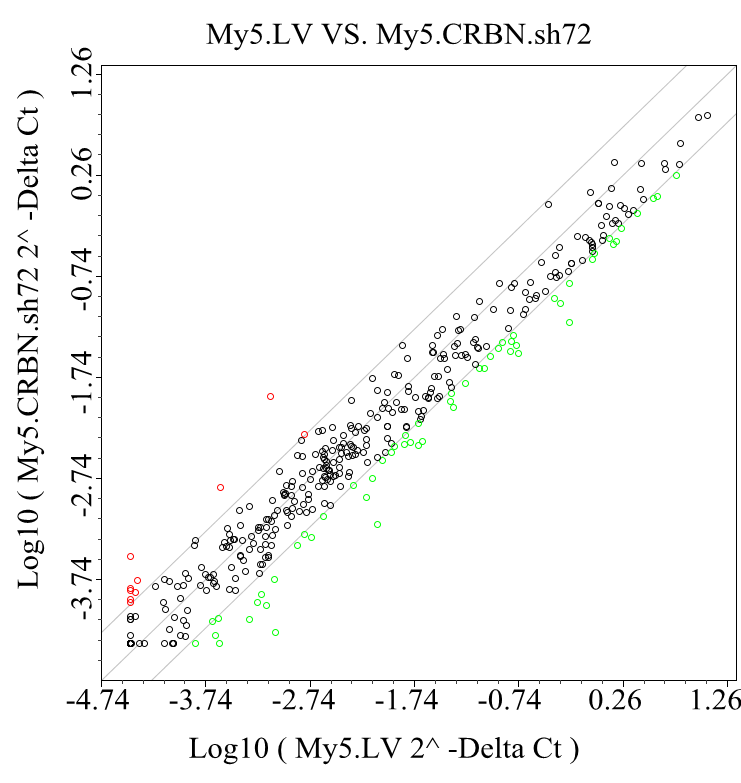

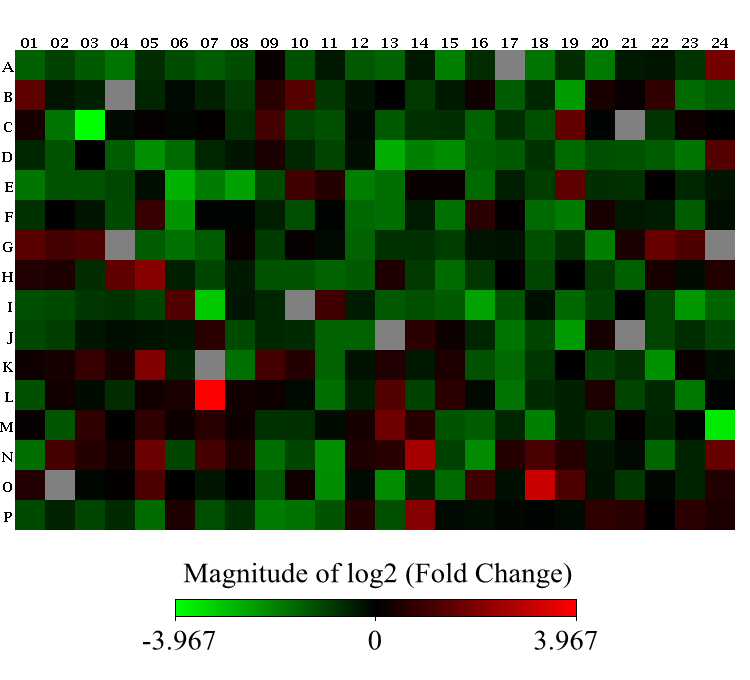


| Arrays included in Test Group: | | My5.CRBN.sh72 |
| --- | --- | --- |
| Arrays included in Control Group: | | My5.LV |
| Genes Over-Expressed | |  |
| Position | Mature ID | Fold Regulation |
| A24 | hsa-miR-10b-3p | 3.5287 |
| G22 | hsa-miR-22-5p | 3.0651 |
| H05 | hsa-miR-224-5p | 4.455 |
| K05 | hsa-miR-34b-5p | 4.073 |
| L07 | hsa-miR-410-3p | 15.4531 |
| M13 | hsa-miR-498 | 3.3562 |
| N05 | hsa-miR-520f-3p | 3.1127 |
| N14 | hsa-miR-551b-3p | 5.9441 |
| O18 | hsa-miR-802 | 8.889 |
| P14 | cel-miR-39-3p | 4.2843 |
| Genes Under-Expressed | |  |
| Position | Mature ID | Fold Regulation |
| A04 | hsa-let-7d-5p | -3.5141 |
| A15 | hsa-miR-103a-3p | -3.9986 |
| A18 | hsa-miR-106b-5p | -3.4581 |
| A20 | hsa-miR-107 | -3.7017 |
| B19 | hsa-miR-129-1-3p | -5.3264 |
| B23 | hsa-miR-130a-3p | -3.1562 |
| C02 | hsa-miR-132-5p | -3.472 |
| C03 | hsa-miR-1324 | -15.643 |
| D05 | hsa-miR-148b-3p | -4.7295 |
| D06 | hsa-miR-149-5p | -3.1478 |
| D13 | hsa-miR-155-5p | -6.5983 |
| D14 | hsa-miR-155-3p | -3.9534 |
| D15 | hsa-miR-15a-5p | -4.5953 |
| D19 | hsa-miR-17-5p | -3.225 |
| D23 | hsa-miR-181c-5p | -3.5642 |
| E01 | hsa-miR-181d-5p | -3.5643 |
| E06 | hsa-miR-184 | -6.8629 |
| E07 | hsa-miR-185-5p | -3.7703 |
| E08 | hsa-miR-186-5p | -5.7581 |
| E12 | hsa-miR-18a-5p | -3.9324 |
| E13 | hsa-miR-18a-3p | -3.302 |
| E16 | hsa-miR-190a-5p | -3.1217 |
| F06 | hsa-miR-196a-5p | -5.078 |
| F12 | hsa-miR-199b-5p | -3.0896 |
| F13 | hsa-miR-19a-3p | -3.2484 |
| F15 | hsa-miR-19b-3p | -3.3141 |
| F18 | hsa-miR-200b-3p | -3.1262 |
| F19 | hsa-miR-200c-3p | -3.8237 |
| G06 | hsa-miR-20a-3p | -3.3456 |
| G20 | hsa-miR-219a-5p | -3.9334 |
| H15 | hsa-miR-26b-5p | -3.2102 |
| I07 | hsa-miR-301a-3p | -8.7467 |
| I16 | hsa-miR-30c-1-3p | -5.8015 |
| I19 | hsa-miR-30e-5p | -3.0867 |
| I23 | hsa-miR-32-5p | -5.1285 |
| J17 | hsa-miR-33a-5p | -3.469 |
| J19 | hsa-miR-33b-5p | -5.3777 |
| K08 | hsa-miR-361-5p | -3.3115 |
| K17 | hsa-miR-374c-5p | -3.159 |
| K22 | hsa-miR-377-3p | -4.8289 |
| L11 | hsa-miR-423-5p | -3.2462 |
| L17 | hsa-miR-429 | -3.5023 |
| L23 | hsa-miR-454-3p | -3.6373 |
| M18 | hsa-miR-505-3p | -4.0169 |
| M24 | hsa-miR-513a-5p | -12.6964 |
| N01 | hsa-miR-514a-3p | -3.2608 |
| N09 | hsa-miR-532-5p | -3.3064 |
| N11 | hsa-miR-542-3p | -4.7077 |
| N16 | hsa-miR-570-3p | -4.5348 |
| N22 | hsa-miR-588 | -3.0161 |
| O11 | hsa-miR-7-5p | -4.6035 |
| O13 | hsa-miR-708-3p | -4.4842 |
| O15 | hsa-miR-744-5p | -3.1258 |
| P05 | hsa-miR-93-5p | -3.1775 |
| P09 | hsa-miR-98-5p | -3.7925 |
| P10 | hsa-miR-99a-5p | -3.4086 |

(c)

Figure S5H. Scatter plot (a) and heat-map (b) of My5.CRBN.sh72 vs My5.CRBN.AGO2& the miRNAs up- or down-regulated in this pair [c].

(b)

(a)


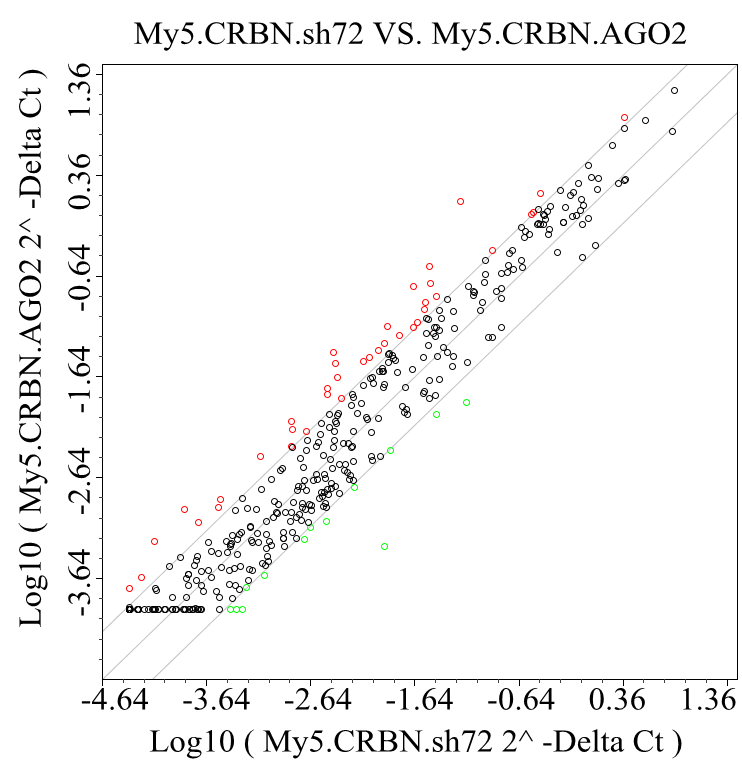

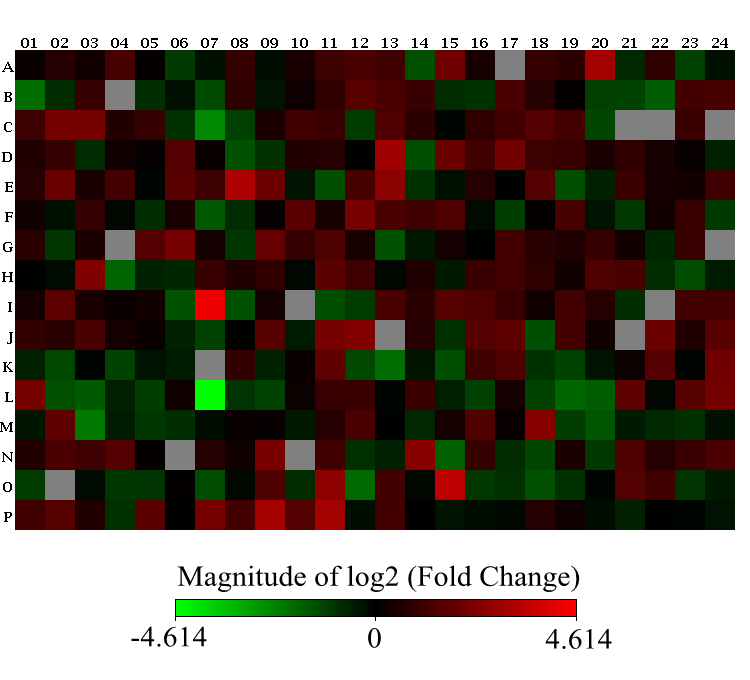


| Arrays included in Test Group: | | My5.CRBN. AGO2 |
| --- | --- | --- |
| Arrays included in Control Group: | | My5.CRBN.sh72 |
| Genes Over-Expressed | | |
| Position | Mature ID | Fold Regulation |
| A15 | hsa-miR-103a-3p | 4.084 |
| A20 | hsa-miR-107 | 7.9237 |
| C02 | hsa-miR-132-5p | 4.4111 |
| C03 | hsa-miR-1324 | 4.3668 |
| D13 | hsa-miR-155-5p | 7.4688 |
| D15 | hsa-miR-15a-5p | 3.8215 |
| D17 | hsa-miR-15b-3p | 4.155 |
| E02 | hsa-miR-182-5p | 3.6654 |
| E08 | hsa-miR-186-5p | 8.937 |
| E09 | hsa-miR-186-3p | 3.8681 |
| E13 | hsa-miR-18a-3p | 5.7512 |
| F10 | hsa-miR-199b-3p | 3.1312 |
| F12 | hsa-miR-199b-5p | 4.5122 |
| G06 | hsa-miR-20a-3p | 4.3836 |
| G09 | hsa-miR-21-5p | 3.6063 |
| H03 | hsa-miR-223-3p | 4.9459 |
| H11 | hsa-miR-24-3p | 3.1121 |
| I02 | hsa-miR-29a-5p | 3.1453 |
| I07 | hsa-miR-301a-3p | 20.1988 |
| J11 | hsa-miR-335-5p | 4.3793 |
| J12 | hsa-miR-335-3p | 5.2725 |
| J17 | hsa-miR-33a-5p | 3.2031 |
| J22 | hsa-miR-342-3p | 3.785 |
| K11 | hsa-miR-365b-3p | 3.2365 |
| K24 | hsa-miR-378a-3p | 4.0902 |
| L01 | hsa-miR-378a-5p | 4.2762 |
| L21 | hsa-miR-449a | 3.1633 |
| L24 | hsa-miR-455-5p | 4.2893 |
| M02 | hsa-miR-484 | 3.2404 |
| M18 | hsa-miR-505-3p | 5.4885 |
| N09 | hsa-miR-532-5p | 4.6067 |
| N14 | hsa-miR-551b-3p | 5.6154 |
| O11 | hsa-miR-7-5p | 5.9514 |
| O15 | hsa-miR-744-5p | 10.7507 |
| P05 | hsa-miR-93-5p | 3.0658 |
| P07 | hsa-miR-95-3p | 4.4071 |
| P09 | hsa-miR-98-5p | 8.0804 |
| P11 | hsa-miR-99a-3p | 7.8863 |
| Genes Under-Expressed | | |
| Position | Mature ID | Fold Regulation |
| B01 | hsa-miR-1180-3p | -3.8304 |
| B22 | hsa-miR-129-5p | -3.1391 |
| C07 | hsa-miR-135a-5p | -5.6229 |
| F07 | hsa-miR-196a-3p | -3.0158 |
| H04 | hsa-miR-223-5p | -3.4896 |
| K13 | hsa-miR-372-3p | -3.9544 |
| L03 | hsa-miR-381-3p | -3.0939 |
| L07 | hsa-miR-410-3p | -24.4948 |
| L19 | hsa-miR-432-5p | -3.5935 |
| L20 | hsa-miR-433-3p | -3.2885 |
| M03 | hsa-miR-485-5p | -4.5028 |
| N15 | hsa-miR-567 | -3.3675 |
| O12 | hsa-miR-708-5p | -3.7848 |

(c)

Figure S5I. Scatter plot (a) and heat-map (b) of My5.LV.AGO2 vs My5.CRBN.sh72 & the miRNAs up- or down-regulated in this pair [c]

(b)

(a)


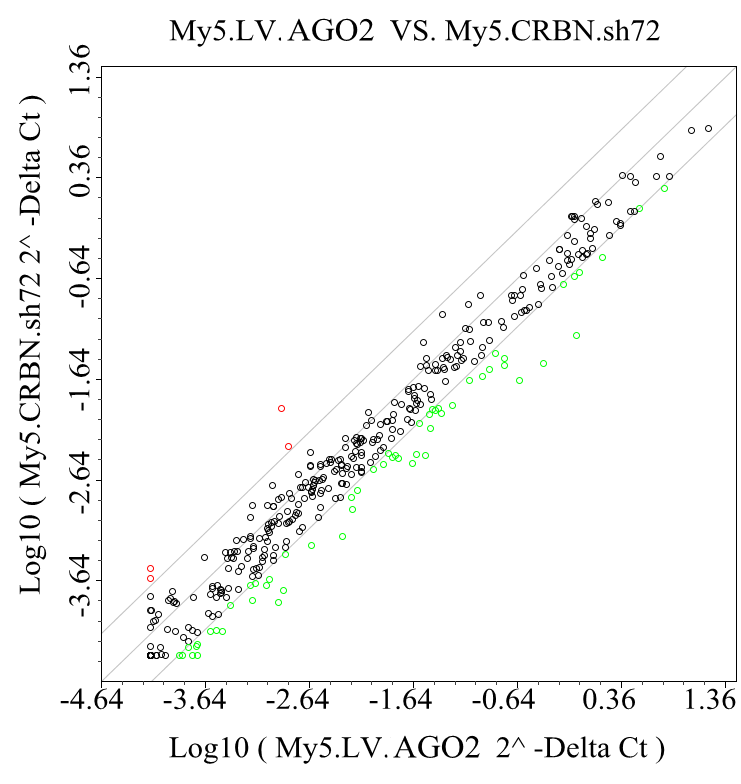

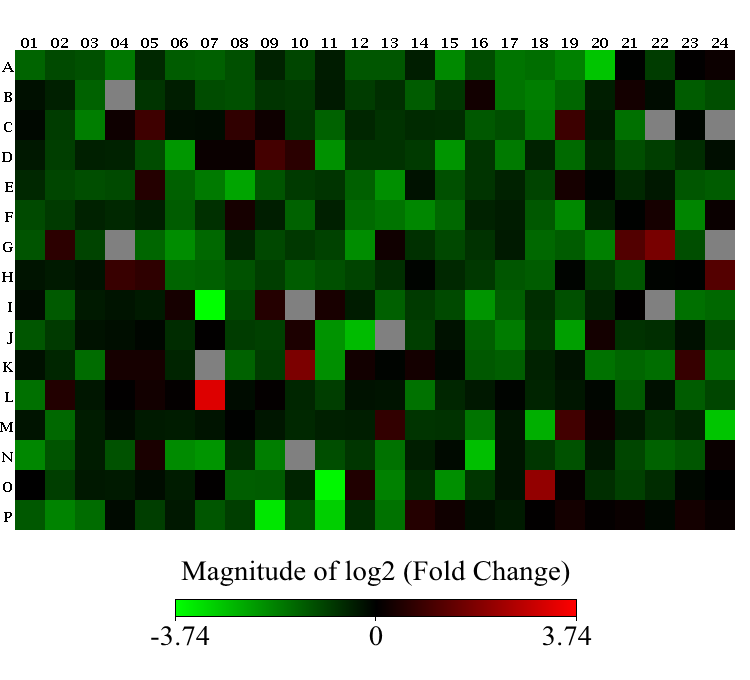


| Arrays included in Test Group: | | My5.CRBN.sh72 |
| --- | --- | --- |
| Arrays included in Control Group: | | My5.LV.AGO2 |
| Genes Over-Expressed | | |
| Position | Mature ID | Fold Regulation |
| G22 | hsa-miR-22-5p | 3.4193 |
| K10 | hsa-miR-363-5p | 3.5289 |
| L07 | hsa-miR-410-3p | 9.481 |
| O18 | hsa-miR-802 | 4.4918 |
| Genes Under-Expressed | | |
| Position | Mature ID | Fold Regulation |
| A04 | hsa-let-7d-5p | -3.3879 |
| A15 | hsa-miR-103a-3p | -4.0725 |
| A17 | hsa-miR-106a-3p | -3.2778 |
| A18 | hsa-miR-106b-5p | -3.0304 |
| A19 | hsa-miR-106b-3p | -3.7325 |
| A20 | hsa-miR-107 | -7.4619 |
| B17 | hsa-miR-128-3p | -3.2722 |
| B18 | hsa-miR-1284 | -3.587 |
| C03 | hsa-miR-1324 | -3.6095 |
| C18 | hsa-miR-142-5p | -3.3669 |
| C21 | hsa-miR-144-3p | -3.1102 |
| D06 | hsa-miR-149-5p | -4.7026 |
| D11 | hsa-miR-153-3p | -4.4327 |
| D15 | hsa-miR-15a-5p | -4.6412 |
| D17 | hsa-miR-15b-3p | -3.43 |
| E07 | hsa-miR-185-5p | -3.4238 |
| E08 | hsa-miR-186-5p | -5.4409 |
| E13 | hsa-miR-18a-3p | -4.3661 |
| F13 | hsa-miR-19a-3p | -3.2814 |
| F14 | hsa-miR-19a-5p | -3.9969 |
| F19 | hsa-miR-200c-3p | -4.0454 |
| F23 | hsa-miR-204-5p | -3.8734 |
| G06 | hsa-miR-20a-3p | -4.2611 |
| G12 | hsa-miR-211-5p | -4.2776 |
| G20 | hsa-miR-219a-5p | -3.6643 |
| I07 | hsa-miR-301a-3p | -13.3629 |
| I16 | hsa-miR-30c-1-3p | -4.5496 |
| I23 | hsa-miR-32-5p | -3.149 |
| J11 | hsa-miR-335-5p | -4.4848 |
| J12 | hsa-miR-335-3p | -6.7332 |
| J17 | hsa-miR-33a-5p | -3.5448 |
| J19 | hsa-miR-33b-5p | -5.0713 |
| K03 | hsa-miR-34a-3p | -3.0172 |
| K11 | hsa-miR-365b-3p | -4.3393 |
| K20 | hsa-miR-376b-3p | -3.1885 |
| K22 | hsa-miR-377-3p | -3.0823 |
| K24 | hsa-miR-378a-3p | -3.1675 |
| L01 | hsa-miR-378a-5p | -3.109 |
| L14 | hsa-miR-425-5p | -3.1711 |
| M16 | hsa-miR-500a-5p | -3.2383 |
| M18 | hsa-miR-505-3p | -5.9295 |
| M24 | hsa-miR-513a-5p | -7.5402 |
| N01 | hsa-miR-514a-3p | -3.9915 |
| N06 | hsa-miR-520g-3p | -4.1358 |
| N07 | hsa-miR-522-3p | -4.5943 |
| N09 | hsa-miR-532-5p | -3.5946 |
| N13 | hsa-miR-549a | -3.1924 |
| N16 | hsa-miR-570-3p | -7.0791 |
| O11 | hsa-miR-7-5p | -12.3525 |
| O13 | hsa-miR-708-3p | -3.7514 |
| O15 | hsa-miR-744-5p | -4.3542 |
| P02 | hsa-miR-92a-1-5p | -3.8208 |
| P03 | hsa-miR-92b-3p | -3.0051 |
| P09 | hsa-miR-98-5p | -10.6693 |
| P11 | hsa-miR-99a-3p | -8.3069 |
| P13 | cel-miR-39-3p | -3.1653 |

(c)

**Figure S6. Treatment of MM cells with lenalidomide altered steady-state levels of miRNAs.** Total RNAs were isolatedafter lenalidomide treatment for 3 or 5 days and the steady-state levels of miRNAs were analyzed with microRNA array kit. Scatter plots (a, +/- 3 fold cut-off) and heat-maps (b) were established by comparison of the steady-state levels of miRNAs between two samples listed in each plot. Up- or down-regulated miRNAs were listed in (c).

**Figure S6A. Scatter plot (a) and heat-map (b) of My5.LV vs My5.LV+ Len 5 days& the miRNAs up- or down-regulated in this pair [c]**


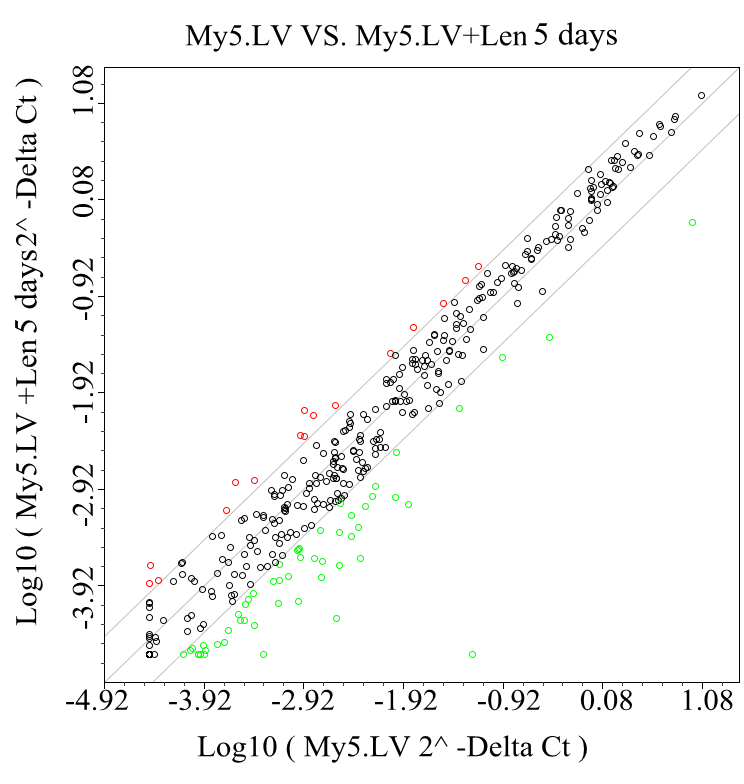

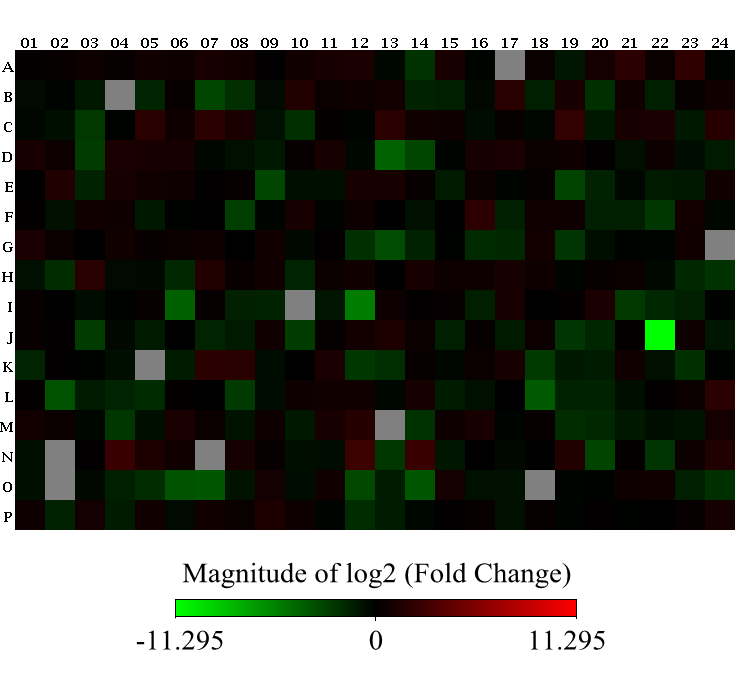


| Arrays included in Test Group: | | My5.LV + Len 5 days | |
| --- | --- | --- | --- |
| Arrays included in Control Group: | | My5.LV | |
| Genes Over-Expressed | | | |
| Position | Mature ID | Fold Regulation | |
| A21 | hsa-miR-10a-5p | 3.6139 | |
| A23 | hsa-miR-10b-5p | 4.0002 | |
| B17 | hsa-miR-128-3p | 3.3529 | |
| C05 | hsa-miR-133b | 3.5035 | |
| C07 | hsa-miR-135a-5p | 3.5296 | |
| C13 | hsa-miR-139-5p | 3.7326 | |
| C19 | hsa-miR-143-3p | 4.7626 | |
| C24 | hsa-miR-145-3p | 3.2509 | |
| F16 | hsa-miR-200a-3p | 3.8673 | |
| H03 | hsa-miR-223-3p | 3.4721 | |
| K07 | hsa-miR-34c-5p | 3.6868 | |
| K08 | hsa-miR-361-5p | 3.4589 | |
| L24 | hsa-miR-455-5p | 3.6318 | |
| N04 | hsa-miR-519d-3p | 5.574 | |
| N12 | hsa-miR-542-5p | 6.4922 | |
| N14 | hsa-miR-551b-3p | 5.5935 | |
| Genes Over-Expressed | | | |
| Position | Mature ID | Fold Regulation | |
| A14 | hsa-miR-101-5p | -4.6481 | |
| B07 | hsa-miR-124-3p | -8.633 | |
| B08 | hsa-miR-124-5p | -4.111 | |
| B20 | hsa-miR-1290 | -4.3062 | |
| C03 | hsa-miR-1324 | -5.5262 | |
| C10 | hsa-miR-137 | -4.48 | |
| D03 | hsa-miR-147a | -6.1754 | |
| D13 | hsa-miR-155-5p | -20.3765 |  |
| D14 | hsa-miR-155-3p | -8.6598 |  |
| E09 | hsa-miR-186-3p | -8.4428 |  |
| E19 | hsa-miR-191-3p | -7.8476 |  |
| F08 | hsa-miR-196b-5p | -6.6059 |  |
| F22 | hsa-miR-203a-3p | -5.2067 |  |
| G12 | hsa-miR-211-5p | -4.4226 |  |
| G13 | hsa-miR-212-3p | -10.1217 |  |
| G16 | hsa-miR-216a-5p | -3.6625 |  |
| G17 | hsa-miR-217 | -3.3708 |  |
| G19 | hsa-miR-218-1-3p | -4.9507 |  |
| H02 | hsa-miR-222-5p | -3.8229 |  |
| H06 | hsa-miR-224-3p | -3.387 |  |
| H23 | hsa-miR-298 | -3.4936 |  |
| H24 | hsa-miR-299-5p | -4.6928 |  |
| I06 | hsa-miR-300 | -19.377 |  |
| I12 | hsa-miR-302c-3p | -45.685 |  |
| I21 | hsa-miR-31-5p | -5.6101 |  |
| I22 | hsa-miR-31-3p | -3.3695 |  |
| J03 | hsa-miR-323b-5p | -6.4814 |  |
| J10 | hsa-miR-331-5p | -6.1596 |  |
| J19 | hsa-miR-33b-5p | -4.8656 |  |
| J20 | hsa-miR-340-5p | -3.1905 |  |
| J22 | hsa-miR-342-3p | -2511.7909 |  |
| K12 | hsa-miR-370-3p | -5.3633 |  |
| K13 | hsa-miR-372-3p | -4.0671 |  |
| K18 | hsa-miR-375 | -5.7846 |  |
| K23 | hsa-miR-377-5p | -4.4738 |  |
| L02 | hsa-miR-379-5p | -12.7666 |  |
| L05 | hsa-miR-383-5p | -3.6797 |  |
| L08 | hsa-miR-411-5p | -5.8608 |  |
| L18 | hsa-miR-431-5p | -16.2953 |  |
| M04 | hsa-miR-486-3p | -5.4048 |  |
| M14 | hsa-miR-499a-3p | -4.7695 |  |
| M19 | hsa-miR-506-3p | -3.9042 |  |
| M20 | hsa-miR-508-5p | -3.3229 |  |
| N13 | hsa-miR-549a | -5.2141 |  |
| N20 | hsa-miR-581 | -8.2059 |  |
| N22 | hsa-miR-588 | -5.0568 |  |
| O05 | hsa-miR-626 | -3.7859 |  |
| O06 | hsa-miR-639 | -12.2935 |  |
| O07 | hsa-miR-643 | -13.9736 |  |
| O12 | hsa-miR-708-5p | -9.3127 |  |
| O14 | hsa-miR-720 | -13.7211 |  |
| O24 | hsa-miR-924 | -4.3368 |  |
| P12 | hsa-miR-99b-5p | -3.744 |  |

(b)

(a)

(c)

**Figure S6B. Scatter plot (a) and heat-map (b) of My5.CRBN vs My5.LV+ Len 5 days & the miRNAs up- or down-regulated in this pair [c]**

(b)

(a)


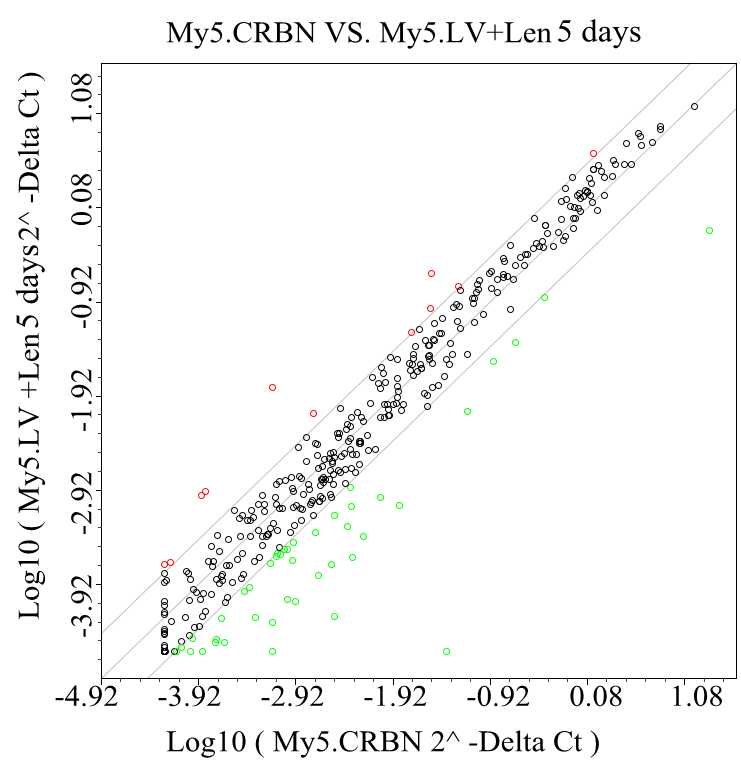

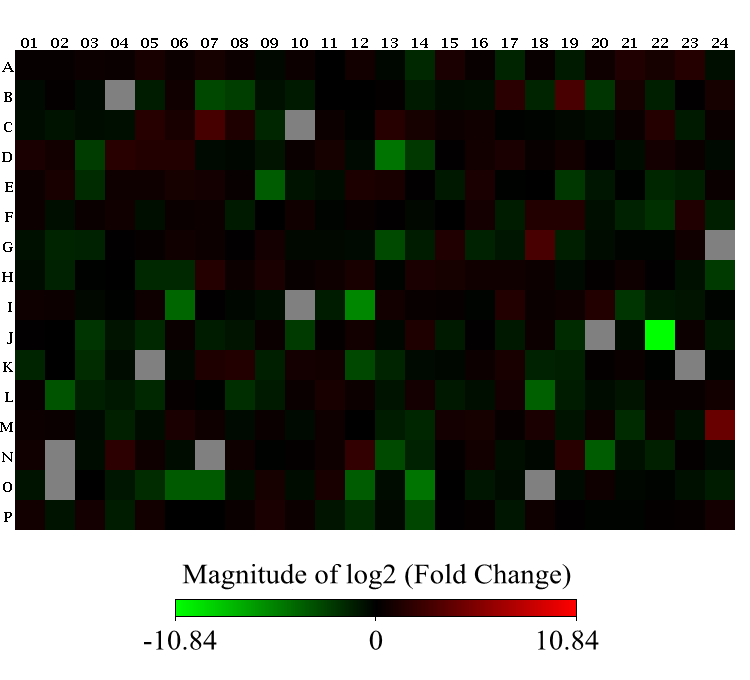


| Arrays included in Test Group: | | My5.LV + Len 5 days | |
| --- | --- | --- | --- |
| Arrays included in Control Group: | | My5.CRBN | |
| Genes Over-Expressed | | | |
| Position | Mature ID | Fold Regulation | |
| B17 | hsa-miR-128-3p | 3.5005 | |
| B19 | hsa-miR-129-1-3p | 8.2334 | |
| C05 | hsa-miR-133b | 3.1268 | |
| C07 | hsa-miR-135a-5p | 8.0426 | |
| C13 | hsa-miR-139-5p | 3.0174 | |
| D04 | hsa-miR-148a-3p | 3.3026 | |
| G18 | hsa-miR-218-5p | 8.1746 | |
| M24 | hsa-miR-513a-5p | 21.5384 | |
| N04 | hsa-miR-519d-3p | 3.6606 | |
| N12 | hsa-miR-542-5p | 4.285 | |
| N19 | hsa-miR-580-3p | 3.3382 | |
| Genes Under-Expressed | | | |
| Position | Mature ID | Fold Regulation | |
| A14 | hsa-miR-101-5p | -3.1737 | |
| B07 | hsa-miR-124-3p | -8.2414 | |
| B08 | hsa-miR-124-5p | -6.1474 | |
| B20 | hsa-miR-1290 | -4.7175 | |
| C09 | hsa-miR-136-5p | -3.0604 | |
| D03 | hsa-miR-147a | -5.9499 | |
| D13 | hsa-miR-155-5p | -29.7893 | |
| D14 | hsa-miR-155-3p | -5.1248 | |
| E03 | hsa-miR-182-3p | -3.3619 | |
| E09 | hsa-miR-186-3p | -14.915 | |
| E19 | hsa-miR-191-3p | -4.8859 | |
| E22 | hsa-miR-192-3p | -3.0054 | |
| F22 | hsa-miR-203a-3p | -4.1998 | |
| G13 | hsa-miR-212-3p | -8.9494 | |
| H05 | hsa-miR-224-5p | -3.2716 | |
| H06 | hsa-miR-224-3p | -3.2891 |  |
| H24 | hsa-miR-299-5p | -5.5792 |  |
| I06 | hsa-miR-300 | -19.7558 |  |
| I12 | hsa-miR-302c-3p | -53.9945 |  |
| I21 | hsa-miR-31-5p | -4.6445 |  |
| J03 | hsa-miR-323b-5p | -4.5199 |  |
| J05 | hsa-miR-324-5p | -3.2455 |  |
| J10 | hsa-miR-331-5p | -5.6197 |  |
| J19 | hsa-miR-33b-5p | -3.4344 |  |
| J22 | hsa-miR-342-3p | -1832.7242 |  |
| K03 | hsa-miR-34a-3p | -3.585 |  |
| K12 | hsa-miR-370-3p | -8.4401 |  |
| L02 | hsa-miR-379-5p | -11.8021 |  |
| L05 | hsa-miR-383-5p | -3.3026 |  |
| L08 | hsa-miR-411-5p | -3.903 |  |
| L18 | hsa-miR-431-5p | -16.9392 |  |
| M14 | hsa-miR-499a-3p | -3.0835 |  |
| M21 | hsa-miR-509-3p | -3.4177 |  |
| N13 | hsa-miR-549a | -8.6693 |  |
| N20 | hsa-miR-581 | -14.8003 |  |
| O05 | hsa-miR-626 | -3.5832 |  |
| O06 | hsa-miR-639 | -13.8653 |  |
| O07 | hsa-miR-643 | -14.3217 |  |
| O12 | hsa-miR-708-5p | -15.4659 |  |
| O14 | hsa-miR-720 | -31.1195 |  |
| P12 | hsa-miR-99b-5p | -3.4756 |  |
| P14 | cel-miR-39-3p | -7.64 |  |

(c)

**Figure S6C. Scatter plot (a) and heat-map (b) of My5.LV.AGO2 vs My5.LV+ Len 5 days& the miRNAs up- or down-regulated in this pair [c]**

(b)

(a)


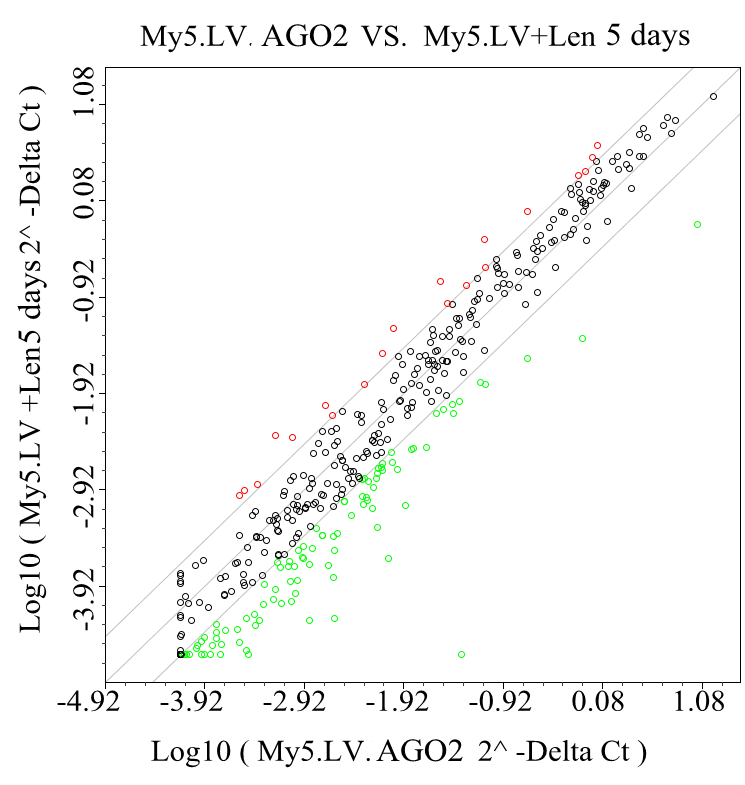

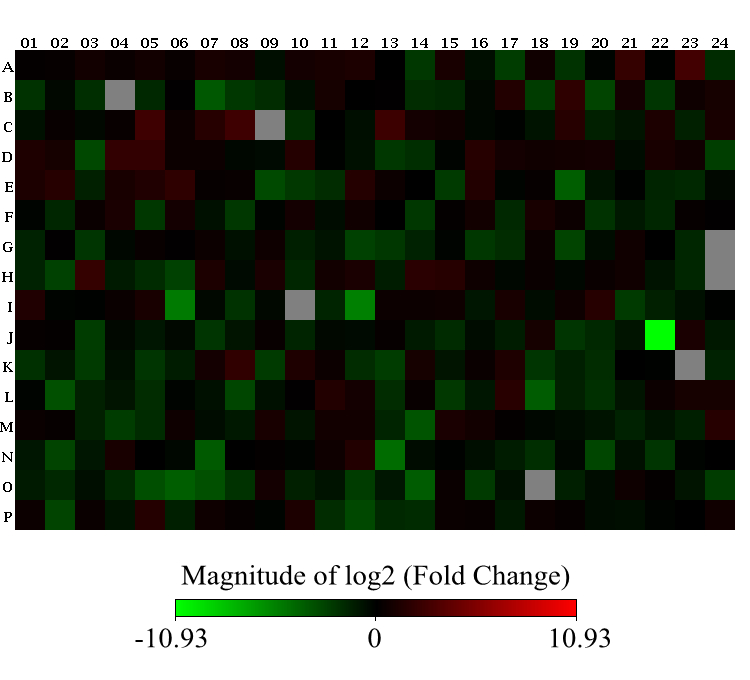


| Arrays included in Test Group: | | My5.LV + Len 5days |
| --- | --- | --- |
| Arrays included in Control Group: | | My5.LV.AGO2 |
| Genes Over-Expressed | | |
| Position | Mature ID | Fold Regulation |
| A21 | hsa-miR-10a-5p | 4.5899 |
| A23 | hsa-miR-10b-5p | 7.1098 |
| B19 | hsa-miR-129-1-3p | 3.8659 |
| C05 | hsa-miR-133b | 6.1852 |
| C07 | hsa-miR-135a-5p | 3.0388 |
| C08 | hsa-miR-135b-5p | 6.1537 |
| C13 | hsa-miR-139-5p | 5.9654 |
| C19 | hsa-miR-143-3p | 3.0381 |
| D04 | hsa-miR-148a-3p | 4.3423 |
| D05 | hsa-miR-148b-3p | 4.3952 |
| D16 | hsa-miR-15b-5p | 3.1585 |
| E02 | hsa-miR-182-5p | 3.1576 |
| E06 | hsa-miR-184 | 3.9056 |
| H03 | hsa-miR-223-3p | 4.6718 |
| H14 | hsa-miR-26a-5p | 3.5447 |
| H15 | hsa-miR-26b-5p | 3.0051 |
| I20 | hsa-miR-30e-3p | 3.1118 |
| K08 | hsa-miR-361-5p | 4.2624 |
| L17 | hsa-miR-429 | 3.3469 |
| M24 | hsa-miR-513a-5p | 3.1125 |
| Genes Under-Expressed | | |
| Position | Mature ID | Fold Regulation |
| A14 | hsa-miR-101-5p | -4.8392 |
| A17 | hsa-miR-106a-3p | -5.8638 |
| A19 | hsa-miR-106b-3p | -4.5292 |
| A24 | hsa-miR-10b-3p | -3.4078 |
| B01 | hsa-miR-1180-3p | -4.3894 |
| B03 | hsa-miR-122-5p | -3.9251 |
| B05 | hsa-miR-1224-3p | -3.2863 |
| B07 | hsa-miR-124-3p | -13.3373 |
| B08 | hsa-miR-124-5p | -4.9261 |
| B09 | hsa-miR-125a-3p | -3.6363 |
| B14 | hsa-miR-1265 | -3.784 |
| B15 | hsa-miR-127-3p | -3.3191 |
| B18 | hsa-miR-1284 | -5.9571 |
| B20 | hsa-miR-1290 | -7.6417 |
| B22 | hsa-miR-129-5p | -4.6404 |
| C10 | hsa-miR-137 | -3.6282 |
| D03 | hsa-miR-147a | -8.5492 |
| D13 | hsa-miR-155-5p | -5.0909 |
| D14 | hsa-miR-155-3p | -3.9702 |
| D24 | hsa-miR-181c-3p | -6.1977 |
| E09 | hsa-miR-186-3p | -8.906 |
| E10 | hsa-miR-187-3p | -5.3032 |
| E11 | hsa-miR-187-5p | -3.7864 |
| E15 | hsa-miR-18b-3p | -5.5841 |
| E19 | hsa-miR-191-3p | -16.7562 |
| E23 | hsa-miR-193a-3p | -3.1914 |
| F02 | hsa-miR-193b-5p | -3.0647 |
| F05 | hsa-miR-195-3p | -5.0177 |
| F08 | hsa-miR-196b-5p | -5.0034 |
| F14 | hsa-miR-19a-5p | -4.9639 |
| F17 | hsa-miR-200a-5p | -3.3527 |
| F20 | hsa-miR-200c-5p | -4.4316 |
| F22 | hsa-miR-203a-3p | -3.067 |
| G03 | hsa-miR-208a-3p | -4.5981 |
| G12 | hsa-miR-211-5p | -6.5689 |
| G13 | hsa-miR-212-3p | -5.0884 |
| G16 | hsa-miR-216a-5p | -4.8936 |
| G17 | hsa-miR-217 | -3.6594 |
| G19 | hsa-miR-218-1-3p | -7.5226 |
| G23 | hsa-miR-221-3p | -3.0196 |
| H02 | hsa-miR-222-5p | -6.7452 |
| H05 | hsa-miR-224-5p | -3.4673 |
| H06 | hsa-miR-224-3p | -6.6374 |
| H10 | hsa-miR-23b-5p | -3.088 |
| H23 | hsa-miR-298 | -3.1353 |
| I06 | hsa-miR-300 | -36.6862 |
| I08 | hsa-miR-302a-3p | -4.3707 |
| I12 | hsa-miR-302c-3p | -44.0527 |
| I21 | hsa-miR-31-5p | -5.5661 |
| J03 | hsa-miR-323b-5p | -6.0386 |
| J07 | hsa-miR-326 | -4.5507 |
| J15 | hsa-miR-338-5p | -3.4927 |
| J19 | hsa-miR-33b-5p | -4.5885 |
| J20 | hsa-miR-340-5p | -3.2263 |
| J22 | hsa-miR-342-3p | -1951.3608 |
| K01 | hsa-miR-346 | -4.0486 |
| K03 | hsa-miR-34a-3p | -5.5842 |
| K05 | hsa-miR-34b-5p | -4.7778 |
| K09 | hsa-miR-363-3p | -5.7319 |
| K12 | hsa-miR-370-3p | -3.6686 |
| K13 | hsa-miR-372-3p | -6.0272 |
| K18 | hsa-miR-375 | -4.5858 |
| K20 | hsa-miR-376b-3p | -3.6947 |
| L02 | hsa-miR-379-5p | -10.911 |
| L05 | hsa-miR-383-5p | -3.7147 |
| L08 | hsa-miR-411-5p | -8.0495 |
| L13 | hsa-miR-424-3p | -3.6697 |
| L15 | hsa-miR-425-3p | -5.4351 |
| L18 | hsa-miR-431-5p | -14.9633 |
| L20 | hsa-miR-433-3p | -4.0787 |
| M04 | hsa-miR-486-3p | -5.9127 |
| M05 | hsa-miR-486-5p | -3.3974 |
| M14 | hsa-miR-499a-3p | -12.263 |
| N02 | hsa-miR-517b-3p | -7.3222 |
| N07 | hsa-miR-522-3p | -14.1788 |
| N13 | hsa-miR-549a | -25.3752 |
| N18 | hsa-miR-575 | -3.9534 |
| N20 | hsa-miR-581 | -8.1593 |
| N22 | hsa-miR-588 | -4.5797 |
| O02 | hsa-miR-606 | -3.3446 |
| O04 | hsa-miR-622 | -3.3602 |
| O05 | hsa-miR-626 | -9.8911 |
| O06 | hsa-miR-639 | -15.9805 |
| O07 | hsa-miR-643 | -10.8217 |
| O08 | hsa-miR-649 | -4.4587 |
| O12 | hsa-miR-708-5p | -6.0139 |
| O14 | hsa-miR-720 | -15.4331 |
| O16 | hsa-miR-765 | -5.5277 |
| O24 | hsa-miR-924 | -6.1247 |
| P02 | hsa-miR-92a-1-5p | -7.5454 |
| P11 | hsa-miR-99a-3p | -3.7246 |
| P12 | hsa-miR-99b-5p | -8.5133 |
| P13 | cel-miR-39-3p | -3.2375 |
| P14 | cel-miR-39-3p | -3.5342 |

(c)

**Figure S6D. Scatter plot (a) and heat-map (b) of My5.CRBN.AGO2 vs My5.LV+ Len 5 days& the miRNAs up- or down-regulated in this pair [c]**


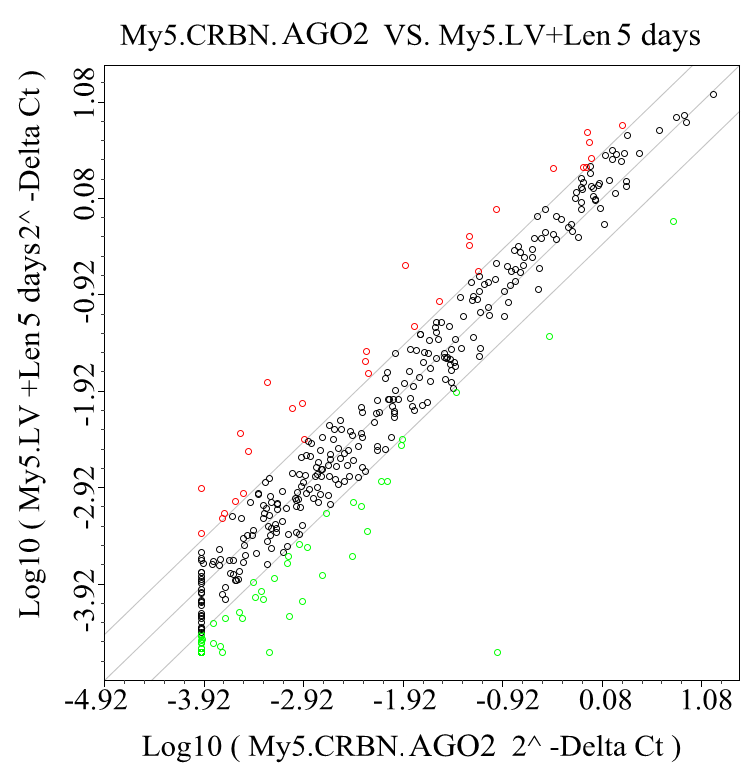

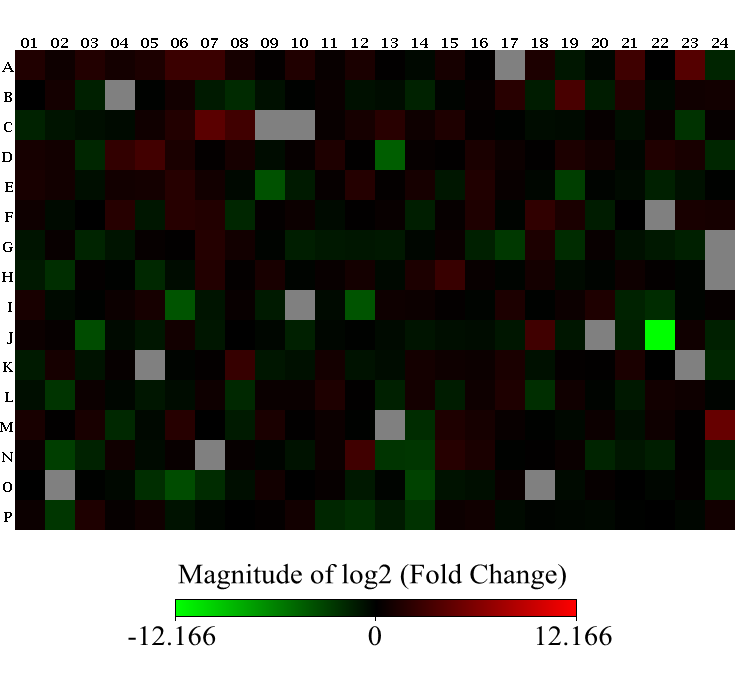


| Arrays included in Test Group: | | My5.LV + Len 5 days |
| --- | --- | --- |
| Arrays included in Control Group: | | My5.CRBN.AGO2 |
| Genes Over-Expressed | | |
| Position | Mature ID | Fold Regulation |
| A03 | hsa-let-7c-5p | 3.0113 |
| A06 | hsa-let-7e-5p | 6.9054 |
| A07 | hsa-let-7f-5p | 6.8084 |
| A21 | hsa-miR-10a-5p | 7.6234 |
| A23 | hsa-miR-10b-5p | 15.8608 |
| B17 | hsa-miR-128-3p | 3.6587 |
| B19 | hsa-miR-129-1-3p | 10.4729 |
| B21 | hsa-miR-129-2-3p | 3.3812 |
| C06 | hsa-miR-134-5p | 3.0636 |
| C07 | hsa-miR-135a-5p | 19.1416 |
| C08 | hsa-miR-135b-5p | 8.5436 |
| C13 | hsa-miR-139-5p | 3.6724 |
| D04 | hsa-miR-148a-3p | 5.1603 |
| D05 | hsa-miR-148b-3p | 8.8964 |
| E06 | hsa-miR-184 | 3.4731 |
| E12 | hsa-miR-18a-5p | 3.2882 |
| F04 | hsa-miR-195-5p | 3.5977 |
| F06 | hsa-miR-196a-5p | 3.4128 |
| F07 | hsa-miR-196a-3p | 3.1249 |
| F18 | hsa-miR-200b-3p | 4.8691 |
| G07 | hsa-miR-20b-5p | 3.241 |
| H07 | hsa-miR-23a-3p | 3.0079 |
| H15 | hsa-miR-26b-5p | 6.2679 |
| J18 | hsa-miR-33a-3p | 8.4166 |
| K08 | hsa-miR-361-5p | 6.0896 |
| M06 | hsa-miR-488-3p | 3.5824 |
| M24 | hsa-miR-513a-5p | 28.5013 |
| N12 | hsa-miR-542-5p | 8.5796 |
| N15 | hsa-miR-567 | 3.4567 |
| Genes Under-Expressed | | |
| Position | Mature ID | Fold Regulation |
| A17 | hsa-miR-106a-3p | -4.7909 |
| A24 | hsa-miR-10b-3p | -3.1865 |
| B04 | hsa-miR-122-3p | -4.7909 |
| B08 | hsa-miR-124-5p | -3.8886 |
| B14 | hsa-miR-1265 | -3.0045 |
| C01 | hsa-miR-132-3p | -3.1808 |
| C09 | hsa-miR-136-5p | -4.7909 |
| C10 | hsa-miR-137 | -4.7909 |
| C23 | hsa-miR-145-5p | -5.2137 |
| D03 | hsa-miR-147a | -3.6087 |
| D13 | hsa-miR-155-5p | -23.0646 |
| D24 | hsa-miR-181c-3p | -3.5226 |
| E09 | hsa-miR-186-3p | -14.5694 |
| E19 | hsa-miR-191-3p | -7.8838 |
| F08 | hsa-miR-196b-5p | -3.6044 |
| F22 | hsa-miR-203a-3p | -4.7909 |
| G03 | hsa-miR-208a-3p | -3.2577 |
| G17 | hsa-miR-217 | -6.4786 |
| G19 | hsa-miR-218-1-3p | -4.417 |
| G24 | hsa-miR-221-5p | -4.7909 |
| H02 | hsa-miR-222-5p | -4.5166 |
| H05 | hsa-miR-224-5p | -3.6619 |
| H24 | hsa-miR-299-5p | -4.7909 |
| I06 | hsa-miR-300 | -16.2592 |
| I10 | hsa-miR-302b-3p | -4.7909 |
| I12 | hsa-miR-302c-3p | -15.5886 |
| I21 | hsa-miR-31-5p | -3.1535 |
| I22 | hsa-miR-31-3p | -4.3499 |
| J03 | hsa-miR-323b-5p | -12.5089 |
| J20 | hsa-miR-340-5p | -4.7909 |
| J22 | hsa-miR-342-3p | -4595.5224 |
| K05 | hsa-miR-34b-5p | -4.7909 |
| K23 | hsa-miR-377-5p | -4.7909 |
| K24 | hsa-miR-378a-3p | -3.6188 |
| L02 | hsa-miR-379-5p | -5.7647 |
| L08 | hsa-miR-411-5p | -3.7839 |
| L18 | hsa-miR-431-5p | -4.518 |
| M04 | hsa-miR-486-3p | -3.7392 |
| M13 | hsa-miR-498 | -4.7909 |
| M14 | hsa-miR-499a-3p | -4.3223 |
| N02 | hsa-miR-517b-3p | -7.9042 |
| N03 | hsa-miR-518b | -3.0812 |
| N07 | hsa-miR-522-3p | -4.7909 |
| N13 | hsa-miR-549a | -5.473 |
| N14 | hsa-miR-551b-3p | -5.9674 |
| N20 | hsa-miR-581 | -3.1946 |
| O02 | hsa-miR-606 | -4.7909 |
| O05 | hsa-miR-626 | -4.5682 |
| O06 | hsa-miR-639 | -12.6715 |
| O07 | hsa-miR-643 | -4.2339 |
| O14 | hsa-miR-720 | -9.0699 |
| O18 | hsa-miR-802 | -4.7909 |
| O24 | hsa-miR-924 | -4.4994 |
| P02 | hsa-miR-92a-1-5p | -5.8705 |
| P11 | hsa-miR-99a-3p | -3.5361 |
| P12 | hsa-miR-99b-5p | -4.582 |
| P14 | cel-miR-39-3p | -5.1547 |

(b)

(a)

(c)

**Figure S6E. Scatter plot (a) and heat-map (b) of My5.LV+ Len 3 days vs My5.LV+ Len 5 days& the miRNAs up- or down-regulated in this pair [c]**

(b)

(a)


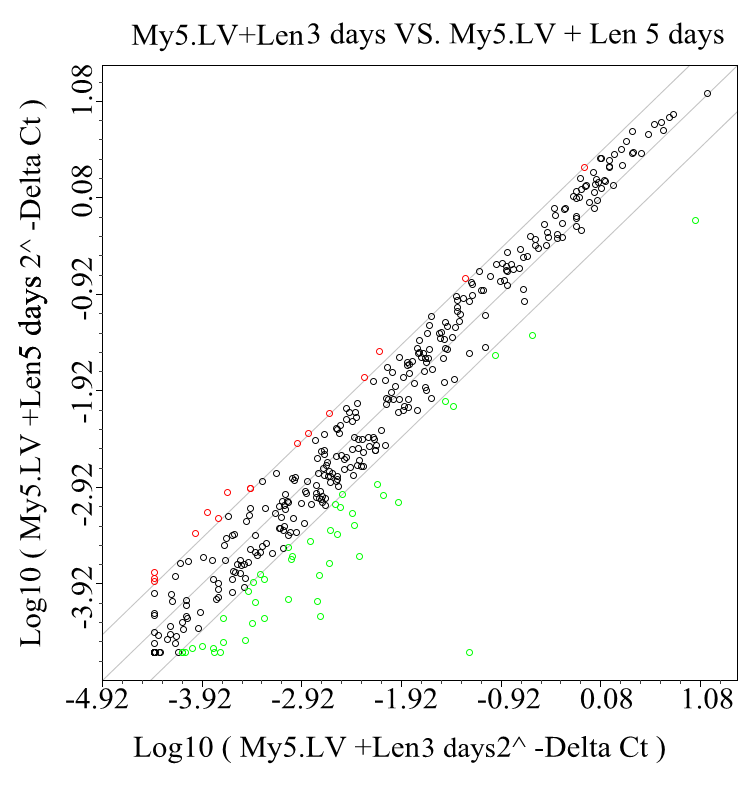

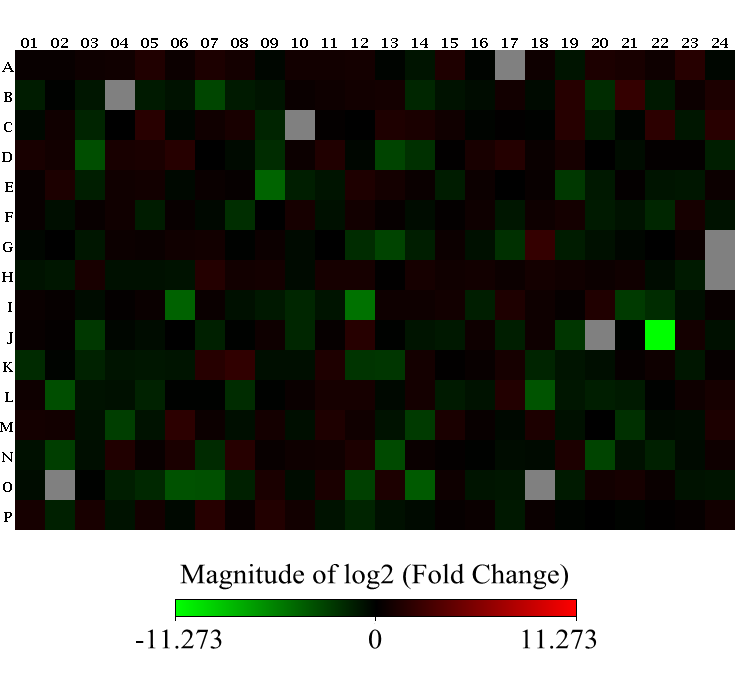


| Arrays included in Test Group: | | My5.LV + Len 5 days |
| --- | --- | --- |
| Arrays included in Control Group: | | My5.LV+ Len 3 days |
| Genes Over-Expressed | | |
| Position | Mature ID | Fold Regulation |
| A23 | hsa-miR-10b-5p | 3.1207 |
| B19 | hsa-miR-129-1-3p | 3.2044 |
| B21 | hsa-miR-129-2-3p | 4.7889 |
| C05 | hsa-miR-133b | 3.3463 |
| C19 | hsa-miR-143-3p | 3.1171 |
| C22 | hsa-miR-144-5p | 3.9405 |
| C24 | hsa-miR-145-3p | 3.4016 |
| D06 | hsa-miR-149-5p | 3.179 |
| G18 | hsa-miR-218-5p | 4.9126 |
| H07 | hsa-miR-23a-3p | 3.0425 |
| J12 | hsa-miR-335-3p | 3.2312 |
| K07 | hsa-miR-34c-5p | 3.1845 |
| K08 | hsa-miR-361-5p | 4.3414 |
| M06 | hsa-miR-488-3p | 3.8785 |
| N08 | hsa-miR-524-5p | 3.2349 |
| P07 | hsa-miR-95-3p | 3.1468 |
| Genes Under-Expressed | | |
| Position | Mature ID | Fold Regulation |
| B07 | hsa-miR-124-3p | -8.3351 |
| B14 | hsa-miR-1265 | -3.1414 |
| B20 | hsa-miR-1290 | -3.7977 |
| C03 | hsa-miR-1324 | -3.0531 |
| D03 | hsa-miR-147a | -10.5973 |
| D09 | hsa-miR-151a-5p | -3.8609 |
| D13 | hsa-miR-155-5p | -7.8694 |
| D14 | hsa-miR-155-3p | -4.3895 |
| E09 | hsa-miR-186-3p | -21.5347 |
| E19 | hsa-miR-191-3p | -5.4811 |
| F08 | hsa-miR-196b-5p | -4.1756 |
| F22 | hsa-miR-203a-3p | -3.2963 |
| G12 | hsa-miR-211-5p | -3.7956 |
| G13 | hsa-miR-212-3p | -7.9943 |
| G17 | hsa-miR-217 | -4.4699 |
| I06 | hsa-miR-300 | -19.659 |
| I10 | hsa-miR-302b-3p | -3.2942 |
| I12 | hsa-miR-302c-3p | -33.7171 |
| I21 | hsa-miR-31-5p | -6.0657 |
| I22 | hsa-miR-31-3p | -3.7425 |
| J03 | hsa-miR-323b-5p | -5.428 |
| J10 | hsa-miR-331-5p | -3.2763 |
| J19 | hsa-miR-33b-5p | -5.3807 |
| J22 | hsa-miR-342-3p | -2474.0927 |
| K01 | hsa-miR-346 | -3.6163 |
| K12 | hsa-miR-370-3p | -4.8347 |
| K13 | hsa-miR-372-3p | -5.3539 |
| K18 | hsa-miR-375 | -3.008 |
| L02 | hsa-miR-379-5p | -10.735 |
| L08 | hsa-miR-411-5p | -3.7579 |
| L18 | hsa-miR-431-5p | -13.503 |
| M04 | hsa-miR-486-3p | -6.5675 |
| M14 | hsa-miR-499a-3p | -5.9644 |
| M21 | hsa-miR-509-3p | -4.4452 |
| N02 | hsa-miR-517b-3p | -6.7778 |
| N07 | hsa-miR-522-3p | -3.5303 |
| N13 | hsa-miR-549a | -9.4865 |
| N20 | hsa-miR-581 | -8.1492 |
| O05 | hsa-miR-626 | -3.4929 |
| O06 | hsa-miR-639 | -12.0501 |
| O07 | hsa-miR-643 | -11.5096 |
| O12 | hsa-miR-708-5p | -7.0834 |
| O14 | hsa-miR-720 | -15.4599 |
| P12 | hsa-miR-99b-5p | -3.0812 |

(c)

**Figure S6F. Scatter plot (a) and heat-map (b) of My5.LV.AGO2+ Len 3 days vs My5.LV + Len 5 days& the miRNAs up- or down-regulated in this pair [c]**

(a)

(c)

(b)


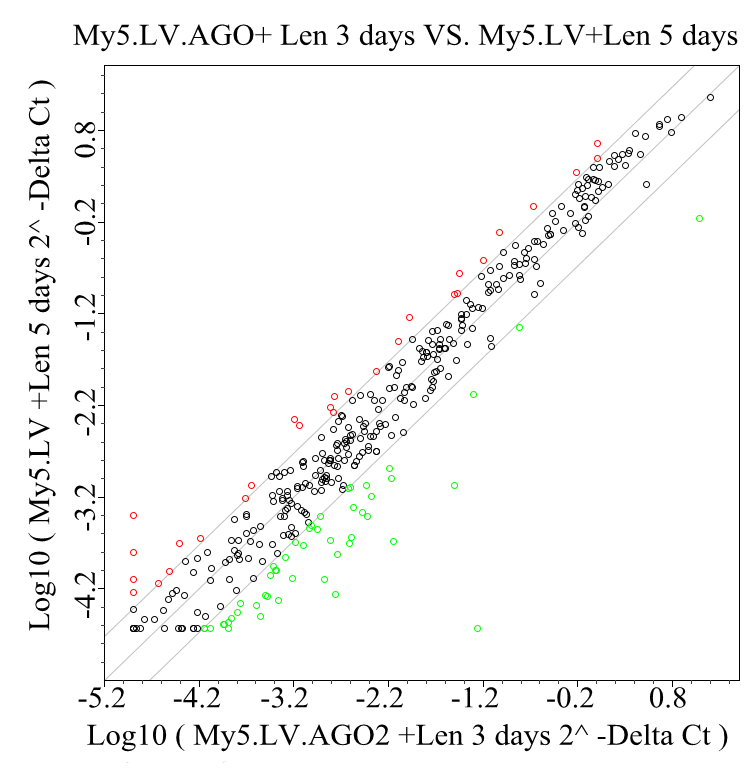

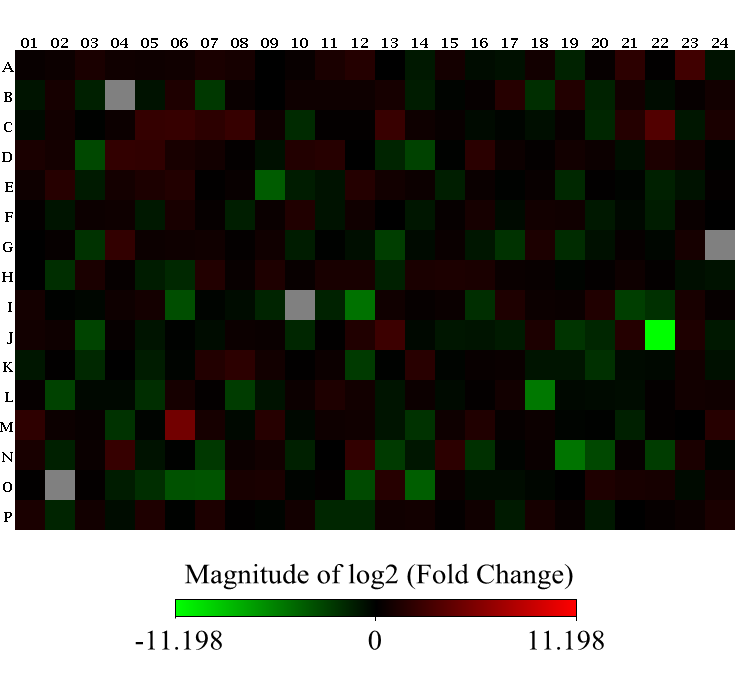


| Arrays included in Test Group: | | My5.LV+ Len 5 days |
| --- | --- | --- |
| Arrays included in Control Group: | | My5.LV.AGO2 + Len 3 days |
| Genes Over-Expressed | |  |
| Position | Mature ID | Fold Regulation |
| A21 | hsa-miR-10a-5p | 3.747 |
| A23 | hsa-miR-10b-5p | 6.9363 |
| B17 | hsa-miR-128-3p | 3.261 |
| C05 | hsa-miR-133b | 4.9755 |
| C06 | hsa-miR-134-5p | 5.1843 |
| C07 | hsa-miR-135a-5p | 3.8614 |
| C08 | hsa-miR-135b-5p | 5.1692 |
| C13 | hsa-miR-139-5p | 5.3683 |
| C21 | hsa-miR-144-3p | 3.0839 |
| C22 | hsa-miR-144-5p | 12.3239 |
| D04 | hsa-miR-148a-3p | 4.4876 |
| D05 | hsa-miR-148b-3p | 4.3475 |
| D11 | hsa-miR-153-3p | 3.1314 |
| D16 | hsa-miR-15b-5p | 3.6168 |
| E02 | hsa-miR-182-5p | 3.1111 |
| E12 | hsa-miR-18a-5p | 3.076 |
| G04 | hsa-miR-208b-3p | 4.4957 |
| J13 | hsa-miR-337-5p | 6.3363 |
| K08 | hsa-miR-361-5p | 3.8762 |
| K14 | hsa-miR-373-3p | 3.4528 |
| M01 | hsa-miR-483-5p | 3.9446 |
| M06 | hsa-miR-488-3p | 31.5816 |
| M09 | hsa-miR-491-5p | 3.1649 |
| M24 | hsa-miR-513a-5p | 3.2073 |
| N04 | hsa-miR-519d-3p | 5.0437 |
| N12 | hsa-miR-542-5p | 4.6744 |
| N15 | hsa-miR-567 | 3.7644 |
| O13 | hsa-miR-708-3p | 3.1132 |
| Genes Under-Expressed | |  |
| Position | Mature ID | Fold Regulation |
| B07 | hsa-miR-124-3p | -5.4779 |
| B18 | hsa-miR-1284 | -3.965 |
| C10 | hsa-miR-137 | -3.5258 |
| C20 | hsa-miR-143-5p | -3.108 |
| D03 | hsa-miR-147a | -9.0119 |
| D13 | hsa-miR-155-5p | -3.0502 |
| D14 | hsa-miR-155-3p | -7.2536 |
| E09 | hsa-miR-186-3p | -16.4975 |
| E19 | hsa-miR-191-3p | -3.4042 |
| G03 | hsa-miR-208a-3p | -4.6981 |
| G13 | hsa-miR-212-3p | -6.7655 |
| G17 | hsa-miR-217 | -4.6771 |
| G19 | hsa-miR-218-1-3p | -3.7099 |
| H02 | hsa-miR-222-5p | -4.0588 |
| H06 | hsa-miR-224-3p | -3.2778 |
| I06 | hsa-miR-300 | -11.0714 |
| I12 | hsa-miR-302c-3p | -31.3718 |
| I16 | hsa-miR-30c-1-3p | -4.0524 |
| I21 | hsa-miR-31-5p | -6.4415 |
| I22 | hsa-miR-31-3p | -4.4294 |
| J03 | hsa-miR-323b-5p | -7.9693 |
| J10 | hsa-miR-331-5p | -3.1329 |
| J19 | hsa-miR-33b-5p | -4.9821 |
| J20 | hsa-miR-340-5p | -3.0986 |
| J22 | hsa-miR-342-3p | -2348.6861 |
| K03 | hsa-miR-34a-3p | -3.4624 |
| K12 | hsa-miR-370-3p | -5.9986 |
| K20 | hsa-miR-376b-3p | -4.265 |
| L02 | hsa-miR-379-5p | -7.4388 |
| L05 | hsa-miR-383-5p | -3.9843 |
| L08 | hsa-miR-411-5p | -6.3439 |
| L18 | hsa-miR-431-5p | -37.624 |
| M04 | hsa-miR-486-3p | -4.6578 |
| M14 | hsa-miR-499a-3p | -4.5248 |
| N07 | hsa-miR-522-3p | -5.5016 |
| N13 | hsa-miR-549a | -5.9662 |
| N16 | hsa-miR-570-3p | -4.3522 |
| N19 | hsa-miR-580-3p | -34.7613 |
| N20 | hsa-miR-581 | -9.1215 |
| N22 | hsa-miR-588 | -6.0249 |
| O05 | hsa-miR-626 | -4.1407 |
| O06 | hsa-miR-639 | -12.2797 |
| O07 | hsa-miR-643 | -12.5763 |
| O12 | hsa-miR-708-5p | -9.7112 |
| O14 | hsa-miR-720 | -17.4331 |
| P02 | hsa-miR-92a-1-5p | -3.0639 |
| P11 | hsa-miR-99a-3p | -3.1336 |
| P12 | hsa-miR-99b-5p | -3.2056 |

**Figure S6G. Scatter plot (a) and heat-map (b) of My5.CRBN+ Len 3 days vs My5.LV+ Len 5 days & the miRNAs up- or down-regulated in this pair [c]**

(b)

(a)


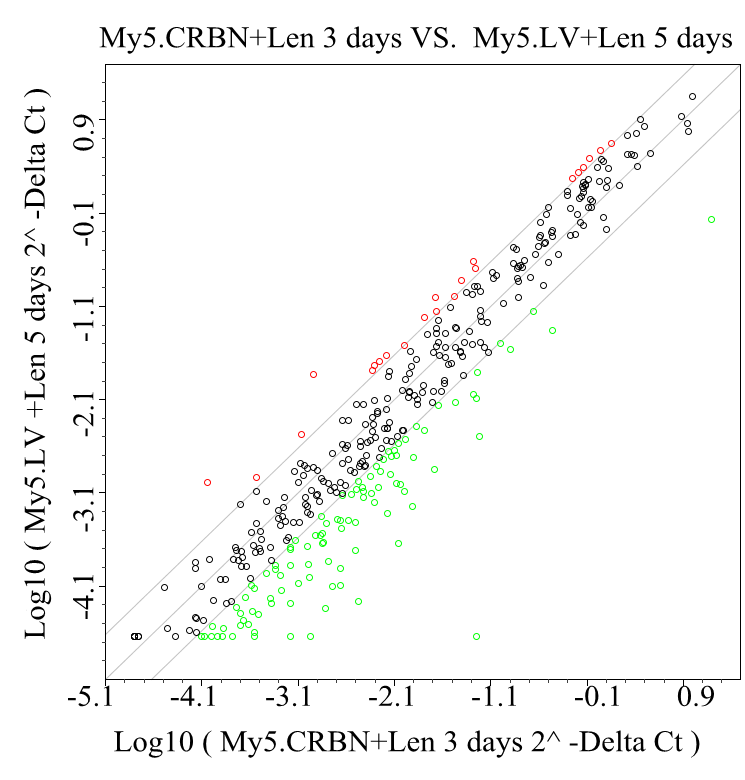

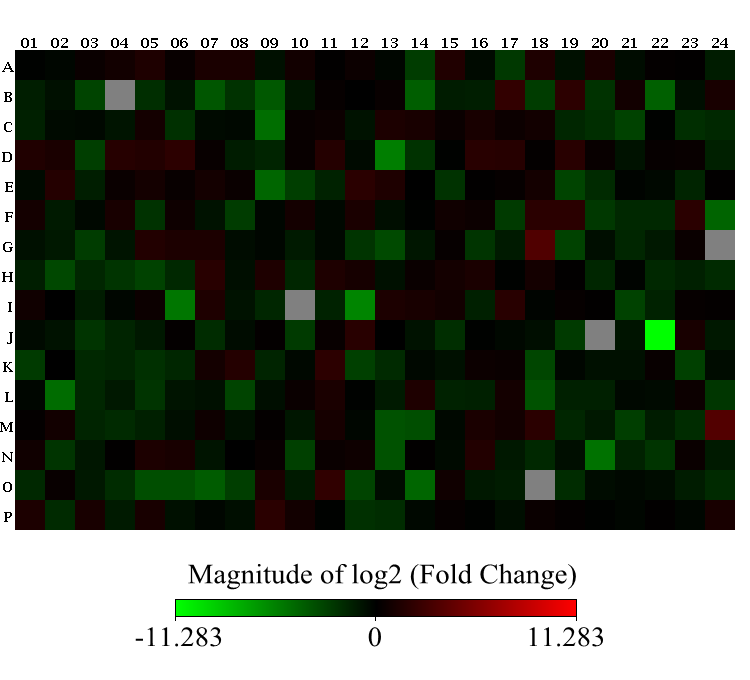


| Arrays included in Test Group: | | My5.LV+ Len 5 days | |
| --- | --- | --- | --- |
| Arrays included in Control Group: | | My5.CRBN+ Len 3 days | |
| Genes Over-Expressed | | | |
| Position | Mature ID | Fold Regulation | |
| B17 | hsa-miR-128-3p | 4.7319 | |
| B19 | hsa-miR-129-1-3p | 3.9288 | |
| D04 | hsa-miR-148a-3p | 3.1443 | |
| D06 | hsa-miR-149-5p | 3.8781 | |
| D16 | hsa-miR-15b-5p | 3.311 | |
| D17 | hsa-miR-15b-3p | 3.2081 | |
| D19 | hsa-miR-17-5p | 3.4004 | |
| E02 | hsa-miR-182-5p | 3.0241 | |
| E12 | hsa-miR-18a-5p | 3.6432 | |
| F18 | hsa-miR-200b-3p | 3.6624 | |
| F19 | hsa-miR-200c-3p | 3.7129 | |
| F23 | hsa-miR-204-5p | 3.7292 | |
| G18 | hsa-miR-218-5p | 11.4409 | |
| H07 | hsa-miR-23a-3p | 3.3477 | |
| I17 | hsa-miR-30d-5p | 3.3511 | |
| J12 | hsa-miR-335-3p | 3.4523 | |
| K08 | hsa-miR-361-5p | 3.0029 | |
| K11 | hsa-miR-365b-3p | 3.7392 | |
| M18 | hsa-miR-505-3p | 3.6989 | |
| M24 | hsa-miR-513a-5p | 12.6082 | |
| O11 | hsa-miR-7-5p | 4.4933 | |
| P09 | hsa-miR-98-5p | 3.6337 | |
| Genes Under-Expressed | | | |
| Position | Mature ID | Fold Regulation | |
| A14 | hsa-miR-101-5p | -6.1965 | |
| A17 | hsa-miR-106a-3p | -5.7217 | |
| B03 | hsa-miR-122-5p | -7.9064 | |
| B05 | hsa-miR-1224-3p | -4.0864 | |
| B07 | hsa-miR-124-3p | -13.975 | |
| B08 | hsa-miR-124-5p | -4.671 | |
| B09 | hsa-miR-125a-3p | -14.4431 |  |
| B14 | hsa-miR-1265 | -17.8922 |  |
| B18 | hsa-miR-1284 | -6.2546 |  |
| B20 | hsa-miR-1290 | -4.6461 |  |
| B22 | hsa-miR-129-5p | -19.2178 |  |
| C06 | hsa-miR-134-5p | -4.4188 |  |
| C09 | hsa-miR-136-5p | -28.9927 |  |
| C19 | hsa-miR-143-3p | -3.2713 |  |
| C20 | hsa-miR-143-5p | -4.1681 |  |
| C21 | hsa-miR-144-3p | -7.6394 |  |
| C23 | hsa-miR-145-5p | -4.1568 |  |
| C24 | hsa-miR-145-3p | -3.4768 |  |
| D03 | hsa-miR-147a | -6.6564 |  |
| D09 | hsa-miR-151a-5p | -3.0972 |  |
| D13 | hsa-miR-155-5p | -46.3938 |  |
| D14 | hsa-miR-155-3p | -4.76 |  |
| E09 | hsa-miR-186-3p | -22.8976 |  |
| E10 | hsa-miR-187-3p | -6.5416 |  |
| E15 | hsa-miR-18b-3p | -4.5283 |  |
| E19 | hsa-miR-191-3p | -7.8272 |  |
| E20 | hsa-miR-1914-3p | -3.6877 |  |
| E23 | hsa-miR-193a-3p | -3.0792 |  |
| F05 | hsa-miR-195-3p | -4.6462 |  |
| F08 | hsa-miR-196b-5p | -6.2795 |  |
| F17 | hsa-miR-200a-5p | -6.0045 |  |
| F20 | hsa-miR-200c-5p | -5.4932 |  |
| F21 | hsa-miR-202-3p | -3.343 |  |
| F22 | hsa-miR-203a-3p | -3.4637 |  |
| F24 | hsa-miR-205-5p | -21.7886 |  |
| G03 | hsa-miR-208a-3p | -6.413 |  |
| G12 | hsa-miR-211-5p | -5.0765 |  |
| G13 | hsa-miR-212-3p | -9.4077 |  |
| G16 | hsa-miR-216a-5p | -4.9217 |  |
| G19 | hsa-miR-218-1-3p | -7.5731 |  |
| G21 | hsa-miR-22-3p | -3.1348 |  |
| H02 | hsa-miR-222-5p | -9.3777 |  |
| H03 | hsa-miR-223-3p | -3.286 |  |
| H04 | hsa-miR-223-5p | -4.9991 |  |
| H05 | hsa-miR-224-5p | -7.7499 |  |
| H06 | hsa-miR-224-3p | -3.3521 |  |
| H10 | hsa-miR-23b-5p | -3.2554 |  |
| H20 | hsa-miR-28-3p | -3.1696 |  |
| H22 | hsa-miR-296-3p | -3.5162 |  |
| H24 | hsa-miR-299-5p | -3.7082 |  |
| I06 | hsa-miR-300 | -38.259 |  |
| I09 | hsa-miR-302a-5p | -3.2437 |  |
| I12 | hsa-miR-302c-3p | -61.6473 |  |
| I21 | hsa-miR-31-5p | -7.7642 |  |
| J03 | hsa-miR-323b-5p | -4.9451 |  |
| J07 | hsa-miR-326 | -3.7709 |  |
| J10 | hsa-miR-331-5p | -5.7877 |  |
| J15 | hsa-miR-338-5p | -4.1758 |  |
| J19 | hsa-miR-33b-5p | -5.768 |  |
| J22 | hsa-miR-342-3p | -2491.2991 |  |
| K01 | hsa-miR-346 | -5.875 |  |
| K03 | hsa-miR-34a-3p | -3.4575 |  |
| K05 | hsa-miR-34b-5p | -4.3462 |  |
| K06 | hsa-miR-34c-3p | -3.2406 |  |
| K12 | hsa-miR-370-3p | -6.9492 |  |
| K13 | hsa-miR-372-3p | -3.6834 |  |
| K18 | hsa-miR-375 | -8.3182 |  |
| K23 | hsa-miR-377-5p | -7.2134 |  |
| L02 | hsa-miR-379-5p | -27.0906 |  |
| L03 | hsa-miR-381-3p | -3.1446 |  |
| L05 | hsa-miR-383-5p | -4.9231 |  |
| L08 | hsa-miR-411-5p | -8.2081 |  |
| L18 | hsa-miR-431-5p | -12.4696 |  |
| L24 | hsa-miR-455-5p | -5.3207 |  |
| M03 | hsa-miR-485-5p | -3.0163 |  |
| M04 | hsa-miR-486-3p | -3.5313 |  |
| M13 | hsa-miR-498 | -12.2411 |  |
| M14 | hsa-miR-499a-3p | -10.9898 |  |
| M19 | hsa-miR-506-3p | -3.1608 |  |
| M21 | hsa-miR-509-3p | -6.8795 |  |
| M23 | hsa-miR-512-5p | -3.8567 |  |
| N02 | hsa-miR-517b-3p | -5.0743 |  |
| N10 | hsa-miR-539-5p | -6.923 |  |
| N13 | hsa-miR-549a | -12.7302 |  |
| N18 | hsa-miR-575 | -3.5161 |  |
| N20 | hsa-miR-581 | -33.3592 |  |
| N22 | hsa-miR-588 | -4.9374 |  |
| O01 | hsa-miR-605-5p | -3.3479 |  |
| O04 | hsa-miR-622 | -3.7749 |  |
| O05 | hsa-miR-626 | -10.716 |  |
| O06 | hsa-miR-639 | -11.1828 |  |
| O07 | hsa-miR-643 | -16.36 |  |
| O08 | hsa-miR-649 | -6.6743 |  |
| O12 | hsa-miR-708-5p | -8.1641 |  |
| O14 | hsa-miR-720 | -23.0236 |  |
| O19 | hsa-miR-885-5p | -3.7444 |  |
| O24 | hsa-miR-924 | -3.7349 |  |
| P02 | hsa-miR-92a-1-5p | -3.7222 |  |
| P12 | hsa-miR-99b-5p | -4.3616 |  |
| P13 | cel-miR-39-3p | -3.8715 |  |

(c)

**Figure S6H. Scatter plot (a) and heat-map (b) of My5.CRBN.AGO2 +Len 3 days vs My5.LV+ Len 5 days& the miRNAs up- or down-regulated in this pair [c]**

(b)

(c)

(a)


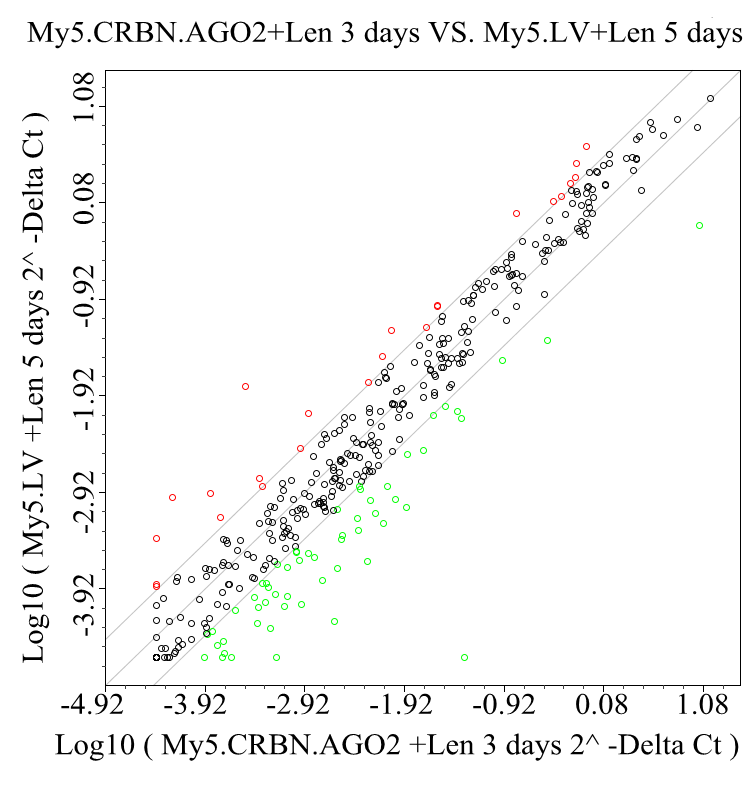

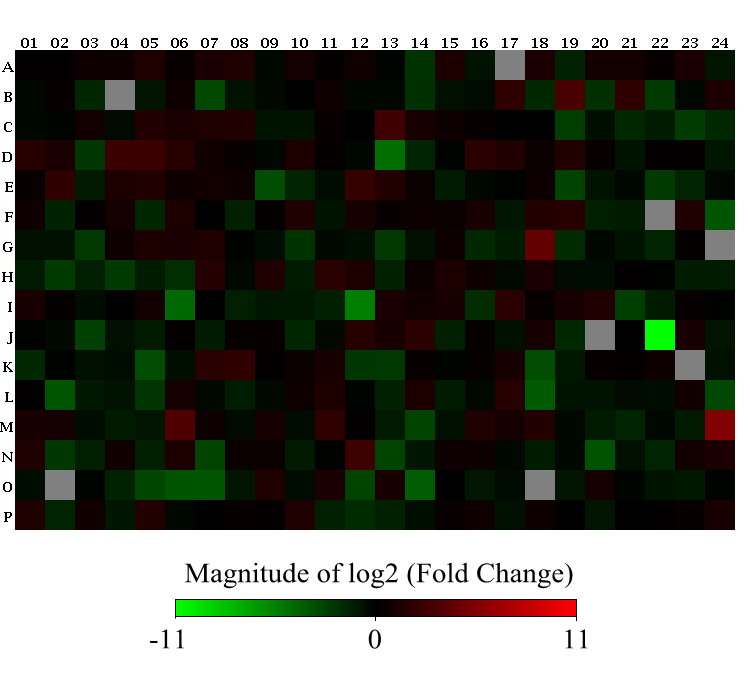


| Arrays included in Test Group: | | My5.LV+ Len 5 days |
| --- | --- | --- |
| Arrays included in Control Group: | | My5.CRBN.AGO2+ Len 3 days |
| Genes Over-Expressed | | |
| Position | Mature ID | Fold Regulation |
| B17 | hsa-miR-128-3p | 3.9481 |
| B19 | hsa-miR-129-1-3p | 8.6472 |
| B21 | hsa-miR-129-2-3p | 3.8539 |
| C13 | hsa-miR-139-5p | 6.3774 |
| D01 | hsa-miR-146a-5p | 3.0733 |
| D04 | hsa-miR-148a-3p | 5.7403 |
| D05 | hsa-miR-148b-3p | 5.8237 |
| D06 | hsa-miR-149-5p | 3.1434 |
| D16 | hsa-miR-15b-5p | 3.5117 |
| E02 | hsa-miR-182-5p | 4.0288 |
| E12 | hsa-miR-18a-5p | 4.8663 |
| F19 | hsa-miR-200c-3p | 3.0849 |
| G18 | hsa-miR-218-5p | 18.5112 |
| H11 | hsa-miR-24-3p | 3.2817 |
| I17 | hsa-miR-30d-5p | 3.4834 |
| J12 | hsa-miR-335-3p | 3.1736 |
| J14 | hsa-miR-338-3p | 3.4265 |
| K07 | hsa-miR-34c-5p | 3.2817 |
| K08 | hsa-miR-361-5p | 4.3059 |
| L17 | hsa-miR-429 | 3.0511 |
| M06 | hsa-miR-488-3p | 10.406 |
| M11 | hsa-miR-495-3p | 3.9086 |
| M24 | hsa-miR-513a-5p | 48.5347 |
| N12 | hsa-miR-542-5p | 6.1147 |
| Genes Under-Expressed | | |
| Position | Mature ID | Fold Regulation |
| A14 | hsa-miR-101-5p | -4.428 |
| B03 | hsa-miR-122-5p | -3.1598 |
| B07 | hsa-miR-124-3p | -8.3953 |
| B14 | hsa-miR-1265 | -4.1236 |
| B18 | hsa-miR-1284 | -3.2731 |
| B20 | hsa-miR-1290 | -4.1752 |
| B22 | hsa-miR-129-5p | -5.6819 |
| C19 | hsa-miR-143-3p | -6.2989 |
| C21 | hsa-miR-144-3p | -3.3592 |
| C23 | hsa-miR-145-5p | -5.8481 |
| C24 | hsa-miR-145-3p | -3.3724 |
| D03 | hsa-miR-147a | -5.1593 |
| D13 | hsa-miR-155-5p | -26.7007 |
| D14 | hsa-miR-155-3p | -3.0019 |
| E09 | hsa-miR-186-3p | -9.467 |
| E19 | hsa-miR-191-3p | -7.208 |
| E22 | hsa-miR-192-3p | -5.8337 |
| F05 | hsa-miR-195-3p | -3.1462 |
| F24 | hsa-miR-205-5p | -12.969 |
| G03 | hsa-miR-208a-3p | -5.3385 |
| G10 | hsa-miR-21-3p | -4.3373 |
| G13 | hsa-miR-212-3p | -5.4494 |
| G16 | hsa-miR-216a-5p | -3.3756 |
| G19 | hsa-miR-218-1-3p | -3.5598 |
| G22 | hsa-miR-22-5p | -3.0088 |
| H02 | hsa-miR-222-5p | -5.5841 |
| H04 | hsa-miR-223-5p | -5.7236 |
| H06 | hsa-miR-224-3p | -3.9691 |
| I06 | hsa-miR-300 | -22.2482 |
| I12 | hsa-miR-302c-3p | -43.5587 |
| I16 | hsa-miR-30c-1-3p | -3.7981 |
| I21 | hsa-miR-31-5p | -6.2265 |
| J03 | hsa-miR-323b-5p | -6.8183 |
| J10 | hsa-miR-331-5p | -3.1916 |
| J19 | hsa-miR-33b-5p | -3.3477 |
| J22 | hsa-miR-342-3p | -2048.4775 |
| K01 | hsa-miR-346 | -3.3929 |
| K05 | hsa-miR-34b-5p | -9.4193 |
| K12 | hsa-miR-370-3p | -4.9764 |
| K13 | hsa-miR-372-3p | -5.2615 |
| K18 | hsa-miR-375 | -9.6899 |
| L02 | hsa-miR-379-5p | -13.3488 |
| L05 | hsa-miR-383-5p | -4.6193 |
| L18 | hsa-miR-431-5p | -14.9593 |
| L24 | hsa-miR-455-5p | -8.4683 |
| M14 | hsa-miR-499a-3p | -7.1889 |
| N02 | hsa-miR-517b-3p | -5.0704 |
| N07 | hsa-miR-522-3p | -7.615 |
| N13 | hsa-miR-549a | -7.6005 |
| N20 | hsa-miR-581 | -11.6355 |
| O05 | hsa-miR-626 | -8.1466 |
| O06 | hsa-miR-639 | -12.2513 |
| O07 | hsa-miR-643 | -13.1985 |
| O12 | hsa-miR-708-5p | -7.2665 |
| O14 | hsa-miR-720 | -15.9428 |
| P12 | hsa-miR-99b-5p | -3.4546 |

**Figure S6I. Scatter plot and heat-map (b) of My5.LV vs My5.CRBN+ Len 5 days& the miRNAs up- or down-regulated in thispair [c]**

(c)

(b)

(a)


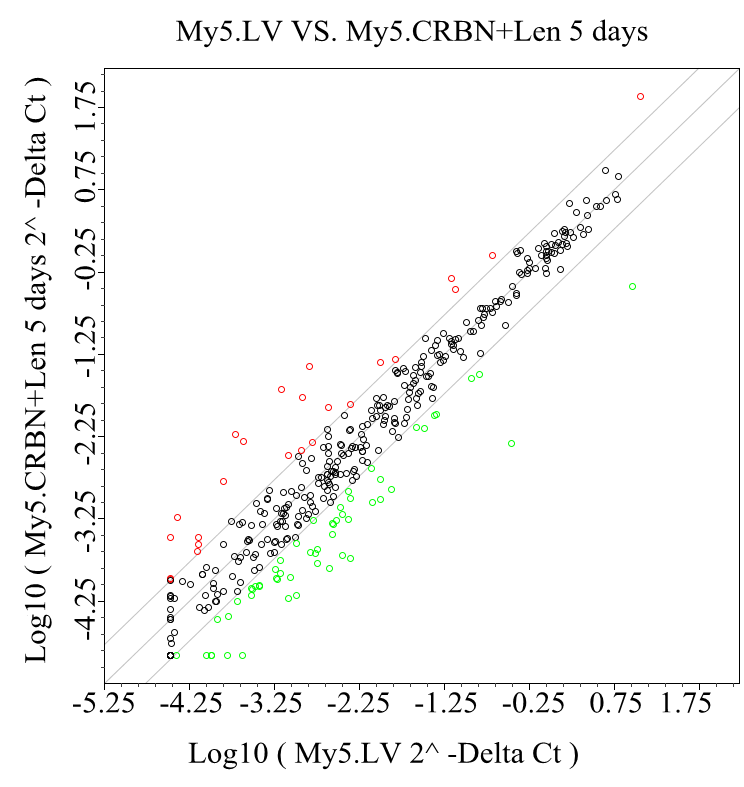

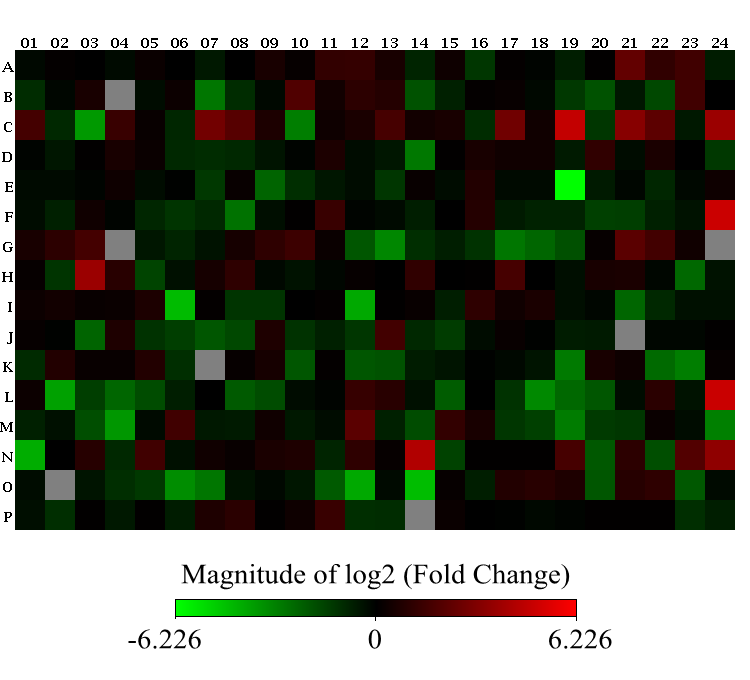


| Arrays included in Test Group: | | My5.CRBN+ Len 5 days | |
| --- | --- | --- | --- |
| Arrays included in Control Group: | | My5.LV | |
| Genes Over-Expressed | |  | |
| Position | Mature ID | Fold Regulation | |
| A21 | hsa-miR-10a-5p | 5.2925 | |
| B10 | hsa-miR-125a-5p | 3.8134 | |
| C01 | hsa-miR-132-3p | 3.1534 | |
| C07 | hsa-miR-135a-5p | 6.9359 | |
| C08 | hsa-miR-135b-5p | 4.2643 | |
| C13 | hsa-miR-139-5p | 3.2896 | |
| C17 | hsa-miR-142-3p | 6.6703 | |
| C19 | hsa-miR-143-3p | 27.2455 | |
| C21 | hsa-miR-144-3p | 9.8576 | |
| C22 | hsa-miR-144-5p | 4.7086 | |
| C24 | hsa-miR-145-3p | 14.205 | |
| F24 | hsa-miR-205-5p | 31.2744 | |
| G03 | hsa-miR-208a-3p | 3.1395 | |
| G21 | hsa-miR-22-3p | 4.6249 | |
| G22 | hsa-miR-22-5p | 3.0516 | |
| H03 | hsa-miR-223-3p | 14.0238 | |
| H17 | hsa-miR-27a-5p | 3.289 | |
| J13 | hsa-miR-337-5p | 3.0674 | |
| L24 | hsa-miR-455-5p | 30.8971 | |
| M12 | hsa-miR-497-5p | 4.5781 | |
| N14 | hsa-miR-551b-3p | 20.1149 | |
| N19 | hsa-miR-580-3p | 3.2275 | |
| N23 | hsa-miR-589-3p | 3.9698 | |
| N24 | hsa-miR-600 | 11.3281 | |
| Genes Under-Expressed | |  | |
| Position | Mature ID | Fold Regulation | |
| B07 | hsa-miR-124-3p | -7.4167 | |
| B14 | hsa-miR-1265 | -4.0472 | |
| B20 | hsa-miR-1290 | -4.0476 | |
| B22 | hsa-miR-129-5p | -3.4314 | |
| C03 | hsa-miR-1324 | -13.4306 |  |
| C10 | hsa-miR-137 | -8.3742 |  |
| D14 | hsa-miR-155-3p | -7.62 |  |
| E09 | hsa-miR-186-3p | -5.3537 |  |
| E19 | hsa-miR-191-3p | -74.8541 |  |
| F08 | hsa-miR-196b-5p | -6.8368 |  |
| G12 | hsa-miR-211-5p | -4.2675 |  |
| G13 | hsa-miR-212-3p | -10.1093 |  |
| G17 | hsa-miR-217 | -7.3295 |  |
| G18 | hsa-miR-218-5p | -5.6702 |  |
| G19 | hsa-miR-218-1-3p | -3.8143 |  |
| H05 | hsa-miR-224-5p | -3.1805 |  |
| H23 | hsa-miR-298 | -5.9019 |  |
| I06 | hsa-miR-300 | -24.0324 |  |
| I12 | hsa-miR-302c-3p | -17.7479 |  |
| I21 | hsa-miR-31-5p | -5.5873 |  |
| J03 | hsa-miR-323b-5p | -5.4658 |  |
| J07 | hsa-miR-326 | -4.3125 |  |
| J08 | hsa-miR-328-3p | -3.4385 |  |
| K10 | hsa-miR-363-5p | -4.3493 |  |
| K12 | hsa-miR-370-3p | -4.34 |  |
| K13 | hsa-miR-372-3p | -4.0117 |  |
| K19 | hsa-miR-376a-3p | -7.9071 |  |
| K22 | hsa-miR-377-3p | -5.9913 |  |
| K23 | hsa-miR-377-5p | -8.3626 |  |
| L02 | hsa-miR-379-5p | -15.6126 |  |
| L04 | hsa-miR-382-5p | -5.5484 |  |
| L05 | hsa-miR-383-5p | -3.5996 |  |
| L08 | hsa-miR-411-5p | -4.556 |  |
| L09 | hsa-miR-421 | -3.6659 |  |
| L15 | hsa-miR-425-3p | -4.7322 |  |
| L18 | hsa-miR-431-5p | -10.3312 |  |
| L19 | hsa-miR-432-5p | -5.8255 |  |
| L20 | hsa-miR-433-3p | -4.3536 |  |
| M03 | hsa-miR-485-5p | -3.7552 |  |
| M04 | hsa-miR-486-3p | -12.9446 |  |
| M14 | hsa-miR-499a-3p | -3.6302 |  |
| M19 | hsa-miR-506-3p | -8.0942 |  |
| M24 | hsa-miR-513a-5p | -8.8679 |  |
| N01 | hsa-miR-514a-3p | -19.2714 |  |
| N15 | hsa-miR-567 | -3.0627 |  |
| N20 | hsa-miR-581 | -4.4404 |  |
| N22 | hsa-miR-588 | -3.7072 |  |
| O06 | hsa-miR-639 | -11.0681 |  |
| O07 | hsa-miR-643 | -7.3097 |  |
| O11 | hsa-miR-7-5p | -4.5453 |  |
| O12 | hsa-miR-708-5p | -17.8749 |  |
| O14 | hsa-miR-720 | -24.7839 |  |
| O20 | hsa-miR-888-5p | -4.3212 |  |
| O23 | hsa-miR-920 | -4.3766 |  |

**Figure S6J. Scatter plot (a) and heat-map (b) of My5.CRBN vs My5.CRBN+Len 5 days& the miRNAs up- or down-regulated in this pair [c]**

(b)

(a)


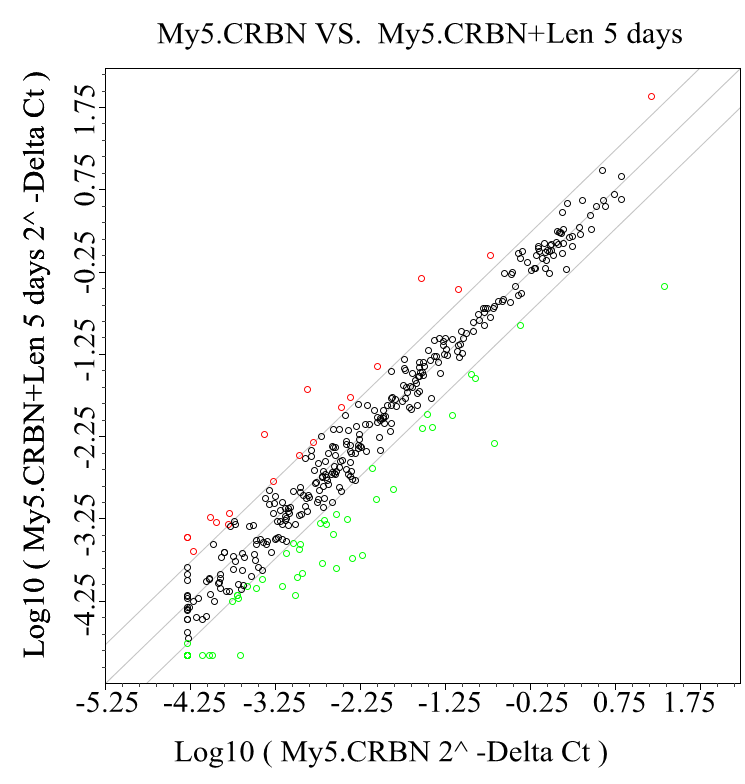

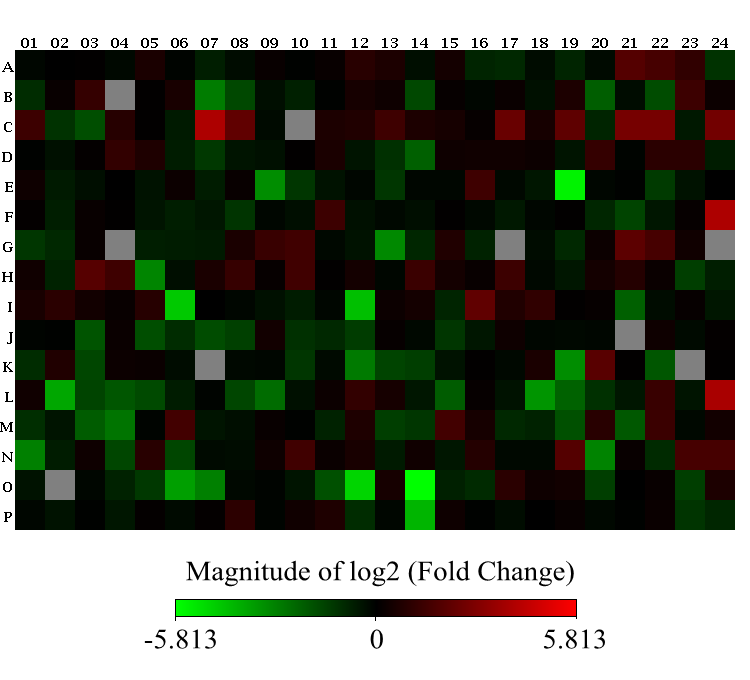


| Arrays included in Test Group: | | My5.CRBN + Len 5 days | |
| --- | --- | --- | --- |
| Arrays included in Control Group: | | My5.CRBN | |
| Genes Over-Expressed | | | |
| Position | Mature ID | Fold Regulation | |
| A21 | hsa-miR-10a-5p | 3.8038 | |
| A22 | hsa-miR-10a-3p | 3.0162 | |
| C07 | hsa-miR-135a-5p | 15.8045 | |
| C08 | hsa-miR-135b-5p | 4.6029 | |
| C17 | hsa-miR-142-3p | 5.1141 | |
| C19 | hsa-miR-143-3p | 4.4837 | |
| C21 | hsa-miR-144-3p | 6.3545 | |
| C22 | hsa-miR-144-5p | 6.421 | |
| C24 | hsa-miR-145-3p | 6.0414 | |
| F24 | hsa-miR-205-5p | 15.6464 | |
| G21 | hsa-miR-22-3p | 4.2951 | |
| G22 | hsa-miR-22-5p | 3.0185 | |
| H03 | hsa-miR-223-3p | 3.8756 | |
| I16 | hsa-miR-30c-1-3p | 4.5788 | |
| K20 | hsa-miR-376b-3p | 3.9766 | |
| L24 | hsa-miR-455-5p | 14.5197 | |
| N19 | hsa-miR-580-3p | 3.7502 | |
| N23 | hsa-miR-589-3p | 3.0398 | |
| N24 | hsa-miR-600 | 3.0594 | |
| Genes Under-Expressed | | | |
| Position | Mature ID | Fold Regulation | |
| B04 | hsa-miR-122-3p | -4.257 | |
| B07 | hsa-miR-124-3p | -7.0803 | |
| B08 | hsa-miR-124-5p | -3.1615 | |
| B14 | hsa-miR-1265 | -3.1128 | |
| B20 | hsa-miR-1290 | -4.4342 | |
| B22 | hsa-miR-129-5p | -3.3713 | |
| C03 | hsa-miR-1324 | -3.3802 | |
| C10 | hsa-miR-137 | -4.257 |  |
| D14 | hsa-miR-155-3p | -4.5095 |  |
| E09 | hsa-miR-186-3p | -9.4577 |  |
| E19 | hsa-miR-191-3p | -46.6039 |  |
| G04 | hsa-miR-208b-3p | -4.257 |  |
| G13 | hsa-miR-212-3p | -8.9384 |  |
| G17 | hsa-miR-217 | -4.257 |  |
| G24 | hsa-miR-221-5p | -4.257 |  |
| H05 | hsa-miR-224-5p | -8.3098 |  |
| I06 | hsa-miR-300 | -24.5021 |  |
| I12 | hsa-miR-302c-3p | -20.976 |  |
| I21 | hsa-miR-31-5p | -4.6256 |  |
| J03 | hsa-miR-323b-5p | -3.8117 |  |
| J05 | hsa-miR-324-5p | -3.4477 |  |
| J07 | hsa-miR-326 | -3.3268 |  |
| J21 | hsa-miR-340-3p | -4.257 |  |
| K07 | hsa-miR-34c-5p | -4.257 |  |
| K12 | hsa-miR-370-3p | -6.8298 |  |
| K19 | hsa-miR-376a-3p | -9.515 |  |
| K22 | hsa-miR-377-3p | -3.9032 |  |
| K23 | hsa-miR-377-5p | -4.257 |  |
| L02 | hsa-miR-379-5p | -14.4331 |  |
| L04 | hsa-miR-382-5p | -3.9326 |  |
| L05 | hsa-miR-383-5p | -3.2308 |  |
| L08 | hsa-miR-411-5p | -3.0341 |  |
| L09 | hsa-miR-421 | -5.4829 |  |
| L15 | hsa-miR-425-3p | -4.2979 |  |
| L18 | hsa-miR-431-5p | -10.7394 |  |
| L19 | hsa-miR-432-5p | -4.6806 |  |
| M03 | hsa-miR-485-5p | -4.2123 |  |
| M04 | hsa-miR-486-3p | -6.3314 |  |
| M19 | hsa-miR-506-3p | -3.5442 |  |
| M21 | hsa-miR-509-3p | -4.0652 |  |
| N01 | hsa-miR-514a-3p | -7.619 |  |
| N04 | hsa-miR-519d-3p | -3.0129 |  |
| N06 | hsa-miR-520g-3p | -3.0658 |  |
| N20 | hsa-miR-581 | -8.0087 |  |
| O02 | hsa-miR-606 | -4.257 |  |
| O06 | hsa-miR-639 | -12.4833 |  |
| O07 | hsa-miR-643 | -7.4918 |  |
| O11 | hsa-miR-7-5p | -3.5686 |  |
| O12 | hsa-miR-708-5p | -29.6854 |  |
| O14 | hsa-miR-720 | -56.2102 |  |
| P14 | cel-miR-39-3p | -17.6255 |  |

(c)

**Figure S6K. Scatter plot (a) and heat-map (b) of My5.LV.AGO2 vs My5.CRBN+ Len 5 days& the miRNAs up- or down-regulated in this pair [c]**

(b)

(a)


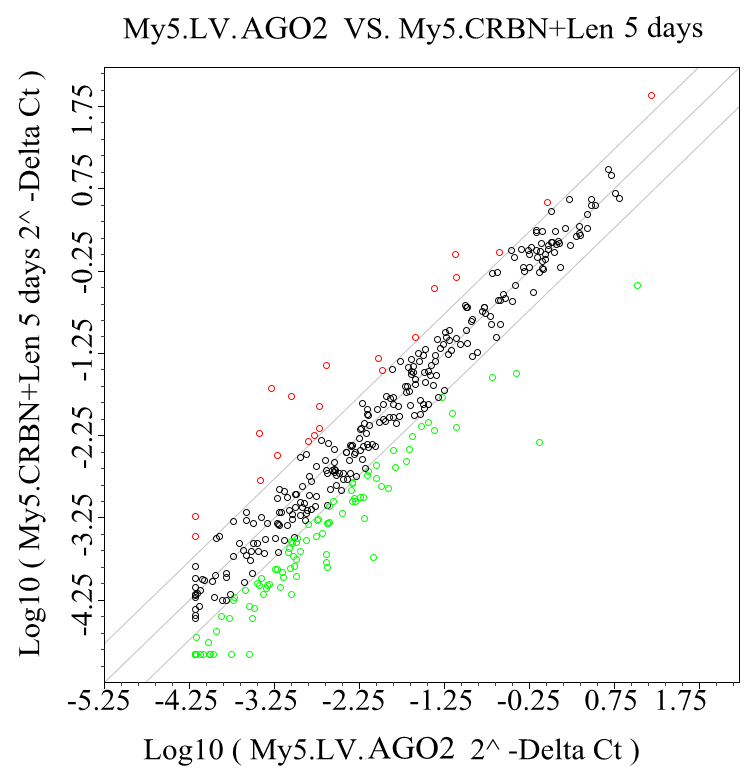

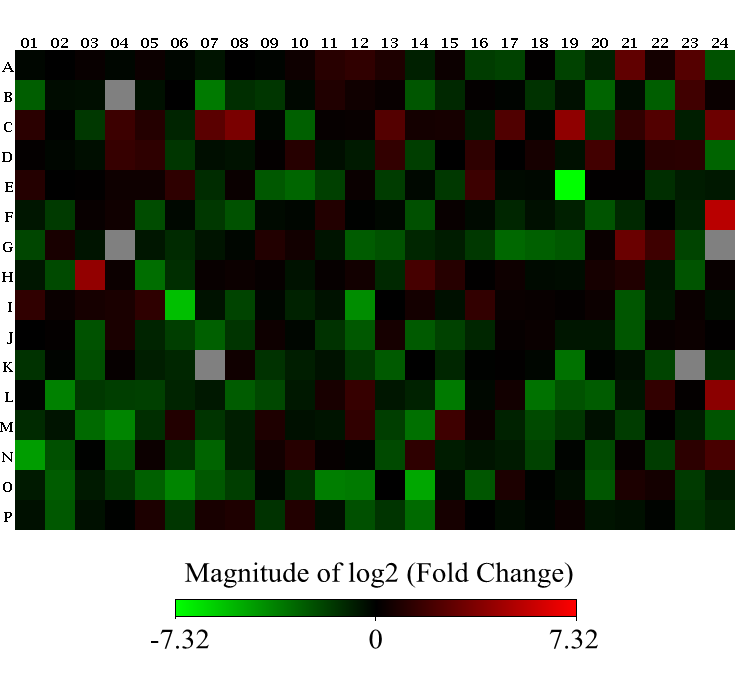


| Arrays included in Test Group: | | My5.CRBN+ Len 5 days | | |
| --- | --- | --- | --- | --- |
| Arrays included in Control Group: | | My5.LV.AGO2 | | |
| Genes Over-Expressed | | | | |
| Position | Mature ID | Fold Regulation | | |
| A21 | hsa-miR-10a-5p | 6.7219 | | |
| A23 | hsa-miR-10b-5p | 5.2683 | | |
| B23 | hsa-miR-130a-3p | 3.629 | | |
| C04 | hsa-miR-133a-3p | 3.3469 | | |
| C07 | hsa-miR-135a-5p | 5.9716 | | |
| C08 | hsa-miR-135b-5p | 11.4807 | | |
| C13 | hsa-miR-139-5p | 5.2575 | | |
| C17 | hsa-miR-142-3p | 4.9565 | | |
| C19 | hsa-miR-143-3p | 17.3801 | | |
| C22 | hsa-miR-144-5p | 5.0333 | | |
| C24 | hsa-miR-145-3p | 8.6957 | | |
| D20 | hsa-miR-181a-5p | 3.6967 | | |
| E16 | hsa-miR-190a-5p | 3.3207 | | |
| F24 | hsa-miR-205-5p | 40.628 | | |
| G21 | hsa-miR-22-3p | 8.0912 | | |
| G22 | hsa-miR-22-5p | 3.4043 | | |
| H03 | hsa-miR-223-3p | 18.8696 | | |
| H14 | hsa-miR-26a-5p | 4.0769 | | |
| L24 | hsa-miR-455-5p | 15.9865 | | |
| M15 | hsa-miR-499a-5p | 3.4348 | | |
| N24 | hsa-miR-600 | 4.1528 | | |
| Genes Under-Expressed | | | | |
| Position | Mature ID | Fold Regulation | | |
| A16 | hsa-miR-105-5p | -3.3204 | | |
| A17 | hsa-miR-106a-3p | -3.77 | | |
| A19 | hsa-miR-106b-3p | -3.7914 | | |
| A24 | hsa-miR-10b-3p | -5.0203 | | |
| B01 | hsa-miR-1180-3p | -6.6181 | | |
| B04 | hsa-miR-122-3p | -5.4307 | | |
| B07 | hsa-miR-124-3p | -11.4581 | |  |
| B14 | hsa-miR-1265 | -5.5335 | |  |
| B20 | hsa-miR-1290 | -7.1829 | |  |
| B22 | hsa-miR-129-5p | -6.4534 | |  |
| C03 | hsa-miR-1324 | -3.0991 | |  |
| C10 | hsa-miR-137 | -6.7821 | |  |
| D14 | hsa-miR-155-3p | -3.4935 | |  |
| D24 | hsa-miR-181c-3p | -7.3155 | |  |
| E09 | hsa-miR-186-3p | -5.6474 | |  |
| E10 | hsa-miR-187-3p | -7.596 | |  |
| E11 | hsa-miR-187-5p | -3.5267 | |  |
| E13 | hsa-miR-18a-3p | -3.2876 | |  |
| E15 | hsa-miR-18b-3p | -3.062 | |  |
| E19 | hsa-miR-191-3p | -159.8293 | |  |
| F02 | hsa-miR-193b-5p | -3.1499 | |  |
| F05 | hsa-miR-195-3p | -4.5911 | |  |
| F07 | hsa-miR-196a-3p | -3.0942 | |  |
| F08 | hsa-miR-196b-5p | -5.1783 | |  |
| F14 | hsa-miR-19a-5p | -4.8408 | |  |
| F20 | hsa-miR-200c-5p | -5.266 | |  |
| G01 | hsa-miR-205-3p | -4.0318 | |  |
| G04 | hsa-miR-208b-3p | -5.4307 | |  |
| G12 | hsa-miR-211-5p | -6.3385 | |  |
| G13 | hsa-miR-212-3p | -5.0822 | |  |
| G16 | hsa-miR-216a-5p | -3.0745 | |  |
| G17 | hsa-miR-217 | -7.9572 | |  |
| G18 | hsa-miR-218-5p | -6.8339 | |  |
| G19 | hsa-miR-218-1-3p | -5.7959 | |  |
| G23 | hsa-miR-221-3p | -3.8036 | |  |
| G24 | hsa-miR-221-5p | -5.4307 | |  |
| H02 | hsa-miR-222-5p | -4.3252 | |  |
| H05 | hsa-miR-224-5p | -8.807 | |  |
| H23 | hsa-miR-298 | -5.2966 | |  |
| I06 | hsa-miR-300 | -45.5001 | |  |
| I08 | hsa-miR-302a-3p | -3.8782 | |  |
| I12 | hsa-miR-302c-3p | -17.1138 | |  |
| I21 | hsa-miR-31-5p | -5.5434 | |  |
| J03 | hsa-miR-323b-5p | -5.0924 | |  |
| J06 | hsa-miR-325 | -3.4276 | |  |
| J07 | hsa-miR-326 | -6.6881 | |  |
| J12 | hsa-miR-335-3p | -5.771 | |  |
| J14 | hsa-miR-338-3p | -6.024 | |  |
| J15 | hsa-miR-338-5p | -3.7643 | |  |
| J21 | hsa-miR-340-3p | -5.5788 | |  |
| K03 | hsa-miR-34a-3p | -4.6502 | |  |
| K07 | hsa-miR-34c-5p | -5.4307 | |  |
| K13 | hsa-miR-372-3p | -5.9451 | |  |
| K19 | hsa-miR-376a-3p | -9.4979 | |  |
| K22 | hsa-miR-377-3p | -3.8243 | |  |
| K23 | hsa-miR-377-5p | -5.4307 | |  |
| L02 | hsa-miR-379-5p | -13.3433 | |  |
| L03 | hsa-miR-381-3p | -3.0882 | |  |
| L04 | hsa-miR-382-5p | -3.4343 | |  |
| L05 | hsa-miR-383-5p | -3.6339 | |  |
| L08 | hsa-miR-411-5p | -6.2575 |  |  |
| L09 | hsa-miR-421 | -4.1084 |  |  |
| L15 | hsa-miR-425-3p | -11.2152 |  |  |
| L18 | hsa-miR-431-5p | -9.4867 |  |  |
| L19 | hsa-miR-432-5p | -5.145 |  |  |
| L20 | hsa-miR-433-3p | -6.305 |  |  |
| M03 | hsa-miR-485-5p | -8.2199 |  |  |
| M04 | hsa-miR-486-3p | -14.1609 |  |  |
| M13 | hsa-miR-498 | -3.4102 |  |  |
| M14 | hsa-miR-499a-3p | -9.3338 |  |  |
| M18 | hsa-miR-505-3p | -4.3449 |  |  |
| M21 | hsa-miR-509-3p | -3.2885 |  |  |
| M24 | hsa-miR-513a-5p | -5.2666 |  |  |
| N01 | hsa-miR-514a-3p | -23.59 |  |  |
| N02 | hsa-miR-517b-3p | -4.997 |  |  |
| N04 | hsa-miR-519d-3p | -5.398 |  |  |
| N07 | hsa-miR-522-3p | -7.2748 |  |  |
| N13 | hsa-miR-549a | -4.3927 |  |  |
| N18 | hsa-miR-575 | -3.6677 |  |  |
| N20 | hsa-miR-581 | -4.4152 |  |  |
| N22 | hsa-miR-588 | -3.3575 |  |  |
| O02 | hsa-miR-606 | -6.2519 |  |  |
| O05 | hsa-miR-626 | -6.7802 |  |  |
| O06 | hsa-miR-639 | -14.3876 |  |  |
| O07 | hsa-miR-643 | -5.6609 |  |  |
| O08 | hsa-miR-649 | -3.3859 |  |  |
| O11 | hsa-miR-7-5p | -12.1963 |  |  |
| O12 | hsa-miR-708-5p | -11.5431 |  |  |
| O14 | hsa-miR-720 | -27.8763 |  |  |
| O16 | hsa-miR-765 | -5.6176 |  |  |
| O20 | hsa-miR-888-5p | -5.6298 |  |  |
| O23 | hsa-miR-920 | -3.1811 |  |  |
| P02 | hsa-miR-92a-1-5p | -5.7173 |  |  |
| P12 | hsa-miR-99b-5p | -4.8771 |  |  |
| P14 | cel-miR-39-3p | -8.1535 |  |  |

(c)

**Figure S6L. Scatter plot (a) and heat-map (b) of My5.CRBN.AGO2 vs. My5.CRBN+ Len 5 days& the miRNAs up- or down-regulated in this pair [c]**

(b)


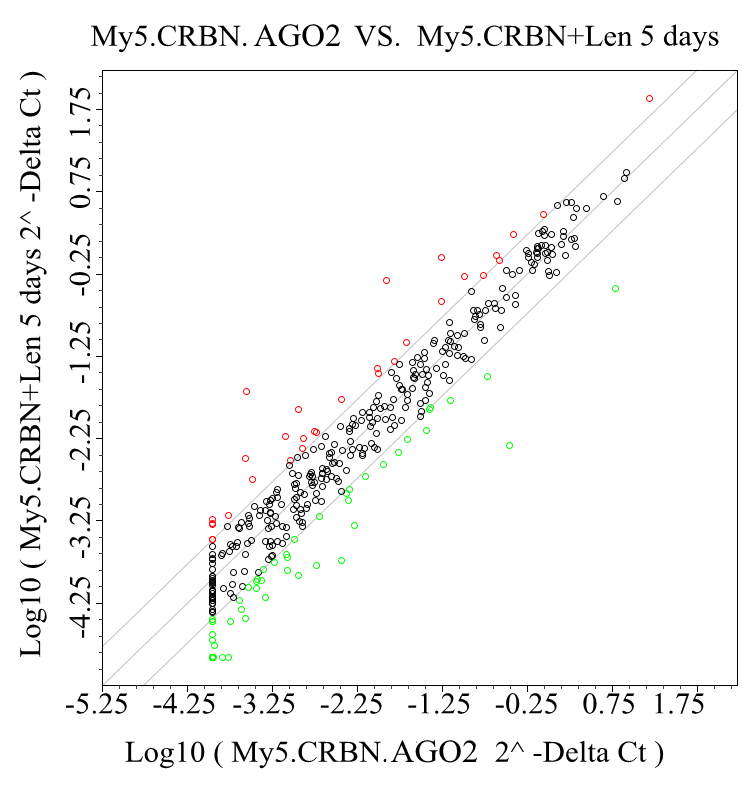

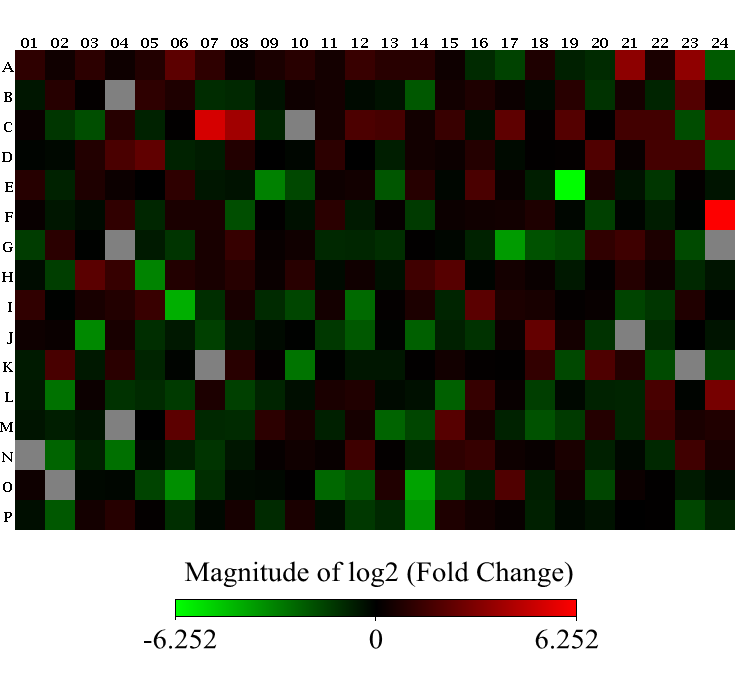


| Arrays included in Test Group: | | My5.CRBN+len5days |
| --- | --- | --- |
| Arrays included in Control Group: | | My5.CRBN.AGO2 |
| Genes Over-Expressed | | |
| Position | Mature ID | Fold Regulation |
| A06 | hsa-let-7e-5p | 4.7029 |
| A21 | hsa-miR-10a-5p | 11.1645 |
| A23 | hsa-miR-10b-5p | 11.7528 |
| B23 | hsa-miR-130a-3p | 3.9949 |
| C07 | hsa-miR-135a-5p | 37.6149 |
| C08 | hsa-miR-135b-5p | 15.9392 |
| C12 | hsa-miR-139-3p | 3.6432 |
| C13 | hsa-miR-139-5p | 3.2366 |
| C17 | hsa-miR-142-3p | 4.916 |
| C19 | hsa-miR-143-3p | 4.0997 |
| C21 | hsa-miR-144-3p | 3.0207 |
| C22 | hsa-miR-144-5p | 3.0523 |
| C24 | hsa-miR-145-3p | 5.2732 |
| D04 | hsa-miR-148a-3p | 3.4579 |
| D05 | hsa-miR-148b-3p | 5.1497 |
| D20 | hsa-miR-181a-5p | 3.8336 |
| D22 | hsa-miR-181b-5p | 3.142 |
| D23 | hsa-miR-181c-5p | 3.2163 |
| E16 | hsa-miR-190a-5p | 3.5517 |
| F24 | hsa-miR-205-5p | 76.2054 |
| H03 | hsa-miR-223-3p | 4.5645 |
| H15 | hsa-miR-26b-5p | 4.3854 |
| I16 | hsa-miR-30c-1-3p | 4.6831 |
| J18 | hsa-miR-33a-3p | 5.4049 |
| K02 | hsa-miR-34a-5p | 3.3858 |
| K20 | hsa-miR-376b-3p | 3.8065 |
| L22 | hsa-miR-451a | 3.3569 |
| L24 | hsa-miR-455-5p | 7.5368 |
| M06 | hsa-miR-488-3p | 4.7235 |
| M15 | hsa-miR-499a-5p | 4.2936 |
| O17 | hsa-miR-770-5p | 3.9133 |
| Genes Under-Expressed | | |
| Position | Mature ID | Fold Regulation |
| A17 | hsa-miR-106a-3p | -3.0802 |
| A24 | hsa-miR-10b-3p | -4.6943 |
| B04 | hsa-miR-122-3p | -8.9554 |
| B14 | hsa-miR-1265 | -4.3935 |
| C03 | hsa-miR-1324 | -3.7492 |
| C10 | hsa-miR-137 | -8.9554 |
| C23 | hsa-miR-145-5p | -3.5996 |
| D24 | hsa-miR-181c-3p | -4.1579 |
| E09 | hsa-miR-186-3p | -9.2386 |
| E10 | hsa-miR-187-3p | -3.3841 |
| E13 | hsa-miR-18a-3p | -4.3306 |
| E19 | hsa-miR-191-3p | -75.2001 |
| F08 | hsa-miR-196b-5p | -3.7304 |
| F20 | hsa-miR-200c-5p | -3 |
| G04 | hsa-miR-208b-3p | -8.9554 |
| G17 | hsa-miR-217 | -14.0872 |
| G18 | hsa-miR-218-5p | -3.9821 |
| G19 | hsa-miR-218-1-3p | -3.4032 |
| G23 | hsa-miR-221-3p | -3.5289 |
| G24 | hsa-miR-221-5p | -8.9554 |
| H05 | hsa-miR-224-5p | -9.3014 |
| I06 | hsa-miR-300 | -20.1655 |
| I10 | hsa-miR-302b-3p | -3.244 |
| I12 | hsa-miR-302c-3p | -6.0559 |
| I21 | hsa-miR-31-5p | -3.1407 |
| J03 | hsa-miR-323b-5p | -10.5488 |
| J12 | hsa-miR-335-3p | -4.5191 |
| J14 | hsa-miR-338-3p | -5.1349 |
| J21 | hsa-miR-340-3p | -8.9554 |
| K07 | hsa-miR-34c-5p | -8.9554 |
| K10 | hsa-miR-363-5p | -7.256 |
| K19 | hsa-miR-376a-3p | -3.4382 |
| K22 | hsa-miR-377-3p | -3.5594 |
| K23 | hsa-miR-377-5p | -8.9554 |
| K24 | hsa-miR-378a-3p | -3.0577 |
| L02 | hsa-miR-379-5p | -7.0498 |
| L15 | hsa-miR-425-3p | -5.1658 |
| M04 | hsa-miR-486-3p | -8.9554 |
| M13 | hsa-miR-498 | -5.5333 |
| M14 | hsa-miR-499a-3p | -3.2898 |
| M18 | hsa-miR-505-3p | -4.0217 |
| N01 | hsa-miR-514a-3p | -8.9554 |
| N02 | hsa-miR-517b-3p | -5.3942 |
| N04 | hsa-miR-519d-3p | -6.654 |
| O02 | hsa-miR-606 | -8.9554 |
| O05 | hsa-miR-626 | -3.1314 |
| O06 | hsa-miR-639 | -11.4085 |
| O11 | hsa-miR-7-5p | -5.8761 |
| O12 | hsa-miR-708-5p | -4.1954 |
| O14 | hsa-miR-720 | -16.3828 |
| O15 | hsa-miR-744-5p | -3.1575 |
| O20 | hsa-miR-888-5p | -3.3387 |
| P02 | hsa-miR-92a-1-5p | -4.4482 |
| P14 | cel-miR-39-3p | -11.8919 |

(c)

(a)

**Figure S6M. Scatter plot (a) and heat-map (b) of My5.LV+Len 3 days vs My5.CRBN+ Len 5 days& the miRNAs up- or down-regulated in this pair [c].**


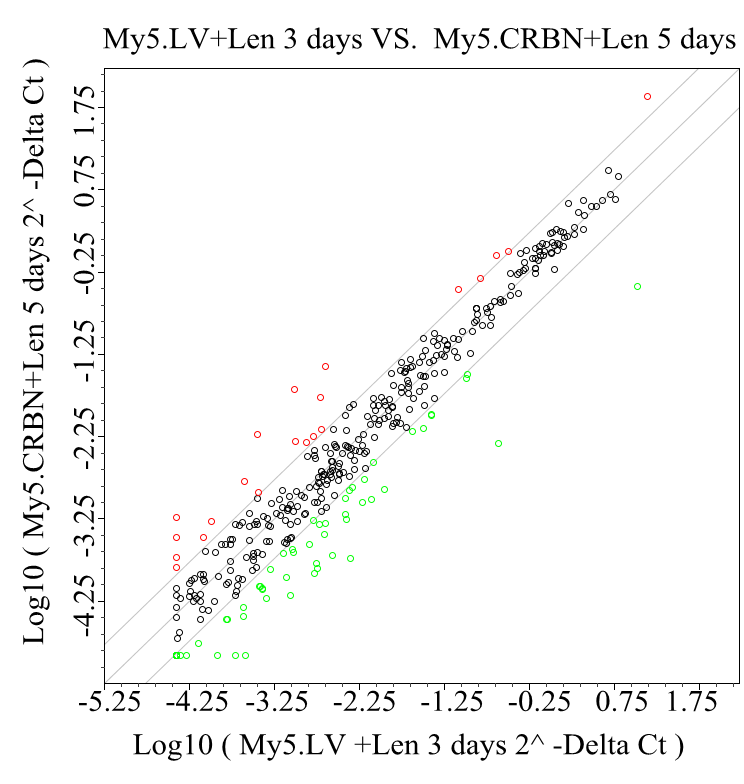

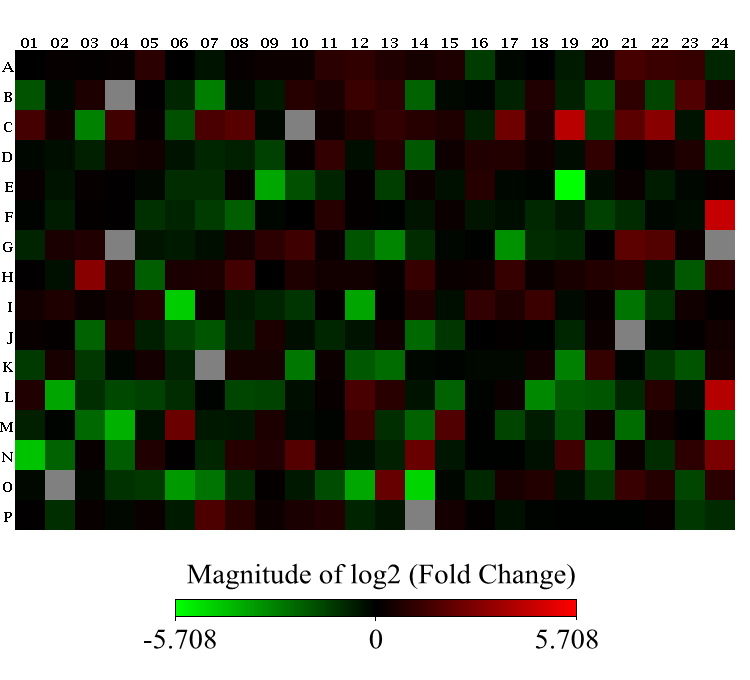


| Arrays included in Test Group: | | My5.CRBN+ Len 5 days | |
| --- | --- | --- | --- |
| Arrays included in Control Group: | | My5.LV+ Len 3 days | |
| Genes Over-Expressed | | | |
| Position | Mature ID | Fold Regulation | |
| B23 | hsa-miR-130a-3p | 3.4523 | |
| C07 | hsa-miR-135a-5p | 3.1471 | |
| C08 | hsa-miR-135b-5p | 3.8112 | |
| C17 | hsa-miR-142-3p | 5.5635 | |
| C19 | hsa-miR-143-3p | 17.8323 | |
| C21 | hsa-miR-144-3p | 4.034 | |
| C22 | hsa-miR-144-5p | 8.6035 | |
| C24 | hsa-miR-145-3p | 14.8636 | |
| F24 | hsa-miR-205-5p | 21.8698 | |
| G21 | hsa-miR-22-3p | 4.1986 | |
| G22 | hsa-miR-22-5p | 3.5594 | |
| H03 | hsa-miR-223-3p | 8.4894 | |
| L12 | hsa-miR-424-5p | 3.082 | |
| L24 | hsa-miR-455-5p | 16.8 | |
| M06 | hsa-miR-488-3p | 5.1139 | |
| M15 | hsa-miR-499a-5p | 3.4843 | |
| N10 | hsa-miR-539-5p | 3.6968 | |
| N14 | hsa-miR-551b-3p | 4.9971 | |
| N24 | hsa-miR-600 | 6.5633 | |
| O13 | hsa-miR-708-3p | 4.8409 | |
| P07 | hsa-miR-95-3p | 3.2741 | |
| Genes Under-Expressed | | | |
| Position | Mature ID | Fold Regulation | |
| B01 | hsa-miR-1180-3p | -3.6473 | |
| B04 | hsa-miR-122-3p | -3.1771 | |
| B07 | hsa-miR-124-3p | -7.1608 | |
| B14 | hsa-miR-1265 | -4.5937 | |
| B20 | hsa-miR-1290 | -3.5697 |  |
| C03 | hsa-miR-1324 | -7.4201 |  |
| C06 | hsa-miR-134-5p | -3.4359 |  |
| C10 | hsa-miR-137 | -3.1771 |  |
| D14 | hsa-miR-155-3p | -3.8625 |  |
| D24 | hsa-miR-181c-3p | -3.0106 |  |
| E09 | hsa-miR-186-3p | -13.6553 |  |
| E10 | hsa-miR-187-3p | -3.4933 |  |
| E19 | hsa-miR-191-3p | -52.2815 |  |
| F08 | hsa-miR-196b-5p | -4.3215 |  |
| G04 | hsa-miR-208b-3p | -3.1771 |  |
| G12 | hsa-miR-211-5p | -3.6625 |  |
| G13 | hsa-miR-212-3p | -7.9845 |  |
| G17 | hsa-miR-217 | -9.7195 |  |
| G24 | hsa-miR-221-5p | -3.1771 |  |
| H05 | hsa-miR-224-5p | -4.2652 |  |
| H23 | hsa-miR-298 | -3.8633 |  |
| I06 | hsa-miR-300 | -24.3821 |  |
| I12 | hsa-miR-302c-3p | -13.0986 |  |
| I21 | hsa-miR-31-5p | -6.041 |  |
| J03 | hsa-miR-323b-5p | -4.5775 |  |
| J07 | hsa-miR-326 | -3.8186 |  |
| J14 | hsa-miR-338-3p | -5.0337 |  |
| J21 | hsa-miR-340-3p | -3.1771 |  |
| K07 | hsa-miR-34c-5p | -3.1771 |  |
| K10 | hsa-miR-363-5p | -6.5153 |  |
| K12 | hsa-miR-370-3p | -3.9123 |  |
| K13 | hsa-miR-372-3p | -5.281 |  |
| K19 | hsa-miR-376a-3p | -7.1786 |  |
| K23 | hsa-miR-377-5p | -3.6359 |  |
| L02 | hsa-miR-379-5p | -13.1281 |  |
| L04 | hsa-miR-382-5p | -3.0343 |  |
| L15 | hsa-miR-425-3p | -4.5314 |  |
| L18 | hsa-miR-431-5p | -8.5608 |  |
| L19 | hsa-miR-432-5p | -4.0223 |  |
| L20 | hsa-miR-433-3p | -3.8406 |  |
| M03 | hsa-miR-485-5p | -5.0517 |  |
| M04 | hsa-miR-486-3p | -15.7292 |  |
| M14 | hsa-miR-499a-3p | -4.5397 |  |
| M19 | hsa-miR-506-3p | -3.3624 |  |
| M21 | hsa-miR-509-3p | -5.2874 |  |
| M24 | hsa-miR-513a-5p | -6.8796 |  |
| N01 | hsa-miR-514a-3p | -20.6686 |  |
| N02 | hsa-miR-517b-3p | -4.6255 |  |
| N04 | hsa-miR-519d-3p | -4.1289 |  |
| N20 | hsa-miR-581 | -4.4097 |  |
| O02 | hsa-miR-606 | -3.1771 |  |
| O06 | hsa-miR-639 | -10.8489 |  |
| O07 | hsa-miR-643 | -6.0207 |  |
| O11 | hsa-miR-7-5p | -3.2804 |  |
| O12 | hsa-miR-708-5p | -13.596 |  |
| O14 | hsa-miR-720 | -27.9247 |  |
| P14 | cel-miR-39-3p | -3.1771 |  |

(c)

(b)

(a)

**Figure S6N. Scatter plot (a) and heat-map (b) of My5.LV.AGO2+ Len 3 days vs My5.CRBN +Len 5 days& the miRNAs up- or down-regulated in this pair [c]**

(b)

(a)


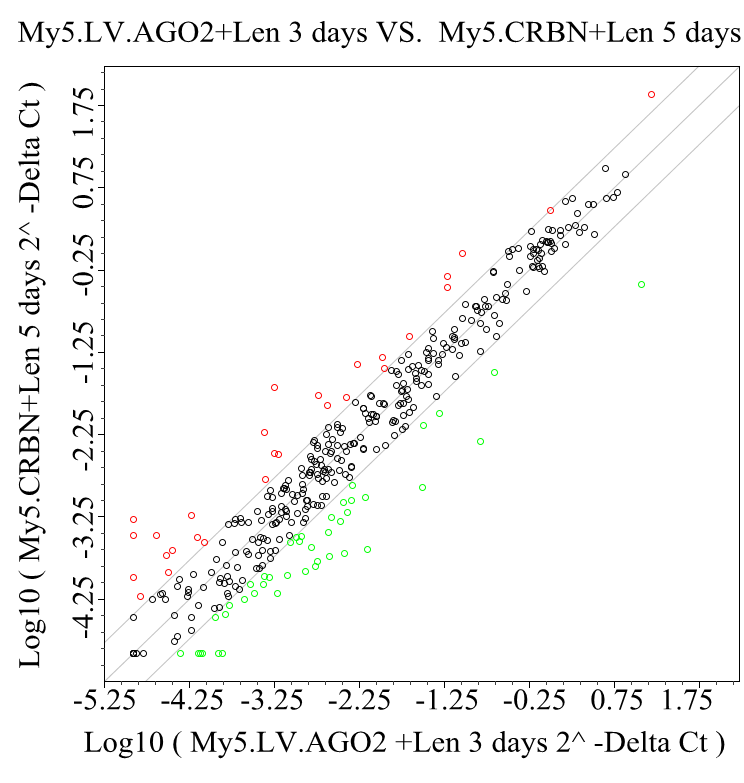

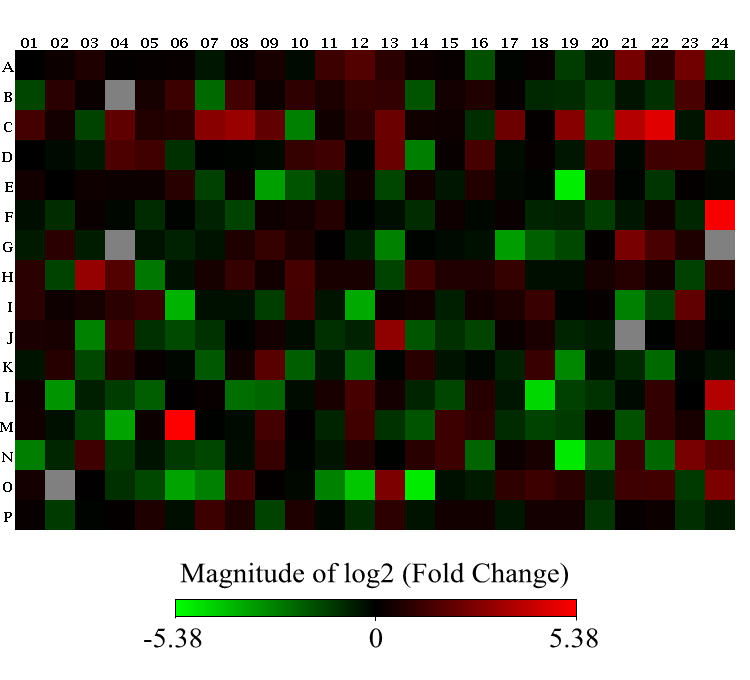


| Arrays included in Test Group: | | My5.CRBN+ Len 5 days | | |
| --- | --- | --- | --- | --- |
| Arrays included in Control Group: | | My5.LV.AGO2 + Len 3 days | | |
| Genes Over-Expressed | | | | |
| Position | Mature ID | Fold Regulation | | |
| A12 | hsa-miR-100-5p | 3.273 | | |
| A21 | hsa-miR-10a-5p | 5.4875 | | |
| A23 | hsa-miR-10b-5p | 5.1398 | | |
| C04 | hsa-miR-133a-3p | 3.935 | | |
| C07 | hsa-miR-135a-5p | 7.588 | | |
| C08 | hsa-miR-135b-5p | 9.6439 | | |
| C09 | hsa-miR-136-5p | 4.0259 | | |
| C13 | hsa-miR-139-5p | 4.7313 | | |
| C17 | hsa-miR-142-3p | 4.9018 | | |
| C19 | hsa-miR-143-3p | 7.3807 | | |
| C21 | hsa-miR-144-3p | 14.4798 | | |
| C22 | hsa-miR-144-5p | 26.9079 | | |
| C24 | hsa-miR-145-3p | 9.8243 | | |
| D04 | hsa-miR-148a-3p | 3.0071 | | |
| D13 | hsa-miR-155-5p | 4.5323 | | |
| F24 | hsa-miR-205-5p | 37.9136 | | |
| G21 | hsa-miR-22-3p | 5.7525 | | |
| H03 | hsa-miR-223-3p | 9.0518 | | |
| H04 | hsa-miR-223-5p | 3.2866 | | |
| I23 | hsa-miR-32-5p | 4.0357 | | |
| J13 | hsa-miR-337-5p | 8.2863 | | |
| K09 | hsa-miR-363-3p | 3.6411 | | |
| L24 | hsa-miR-455-5p | 13.9776 | | |
| M06 | hsa-miR-488-3p | 41.6412 | | |
| N23 | hsa-miR-589-3p | 5.8079 | | |
| N24 | hsa-miR-600 | 3.6376 | | |
| O13 | hsa-miR-708-3p | 6.2152 | | |
| O24 | hsa-miR-924 | | 6.2025 |  |
| Genes Under-Expressed | | | |  |
| Position | Mature ID | | Fold Regulation |  |
| A16 | hsa-miR-105-5p | | -3.2491 |  |
| B07 | hsa-miR-124-3p | | -4.7061 |  |
| B14 | hsa-miR-1265 | | -3.4572 |  |
| C10 | hsa-miR-137 | | -6.5907 |  |
| C20 | hsa-miR-143-5p | | -3.5789 |  |
| D14 | hsa-miR-155-3p | | -6.3827 |  |
| E09 | hsa-miR-186-3p | | -10.4612 |  |
| E10 | hsa-miR-187-3p | | -3.4367 |  |
| E19 | hsa-miR-191-3p | | -32.4712 |  |
| G13 | hsa-miR-212-3p | | -6.7573 |  |
| G17 | hsa-miR-217 | | -10.1701 |  |
| G18 | hsa-miR-218-5p | | -4.0941 |  |
| H05 | hsa-miR-224-5p | | -5.8389 |  |
| I06 | hsa-miR-300 | | -13.7313 |  |
| I12 | hsa-miR-302c-3p | | -12.1875 |  |
| I21 | hsa-miR-31-5p | | -6.4153 |  |
| J03 | hsa-miR-323b-5p | | -6.7205 |  |
| J14 | hsa-miR-338-3p | | -3.4817 |  |
| K07 | hsa-miR-34c-5p | | -3.5908 |  |
| K10 | hsa-miR-363-5p | | -3.9851 |  |
| K12 | hsa-miR-370-3p | | -4.8541 |  |
| K19 | hsa-miR-376a-3p | | -7.2969 |  |
| K22 | hsa-miR-377-3p | | -4.7654 |  |
| L02 | hsa-miR-379-5p | | -9.0971 |  |
| L05 | hsa-miR-383-5p | | -3.8976 |  |
| L08 | hsa-miR-411-5p | | -4.9316 |  |
| L09 | hsa-miR-421 | | -4.4199 |  |
| L18 | hsa-miR-431-5p | | -23.8535 |  |
| M04 | hsa-miR-486-3p | | -11.1555 |  |
| M14 | hsa-miR-499a-3p | | -3.444 |  |
| M21 | hsa-miR-509-3p | | -3.1969 |  |
| M24 | hsa-miR-513a-5p | | -5.1109 |  |
| N01 | hsa-miR-514a-3p | | -6.2333 |  |
| N16 | hsa-miR-570-3p | | -4.2609 |  |
| N19 | hsa-miR-580-3p | | -30.942 |  |
| N20 | hsa-miR-581 | | -4.9358 |  |
| N22 | hsa-miR-588 | | -4.4169 |  |
| O06 | hsa-miR-639 | | -11.0557 |  |
| O07 | hsa-miR-643 | | -6.5787 |  |
| O11 | hsa-miR-7-5p | | -6.672 |  |
| O12 | hsa-miR-708-5p | | -18.6398 |  |
| O14 | hsa-miR-720 | | -31.489 |  |

(c)

**Figure S6O. Scatter plot (a) and heat-map (b) of My5.CRBN +Len 3 days vs My5.CRBN + Len 5 days& the miRNAs up- or down-regulated in this pair [c].**

(b)

(a)


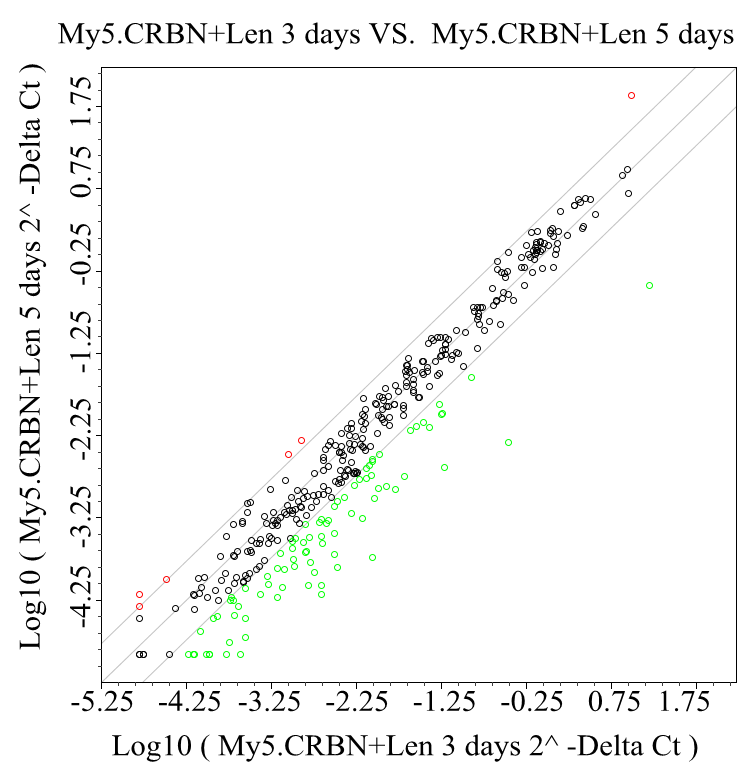

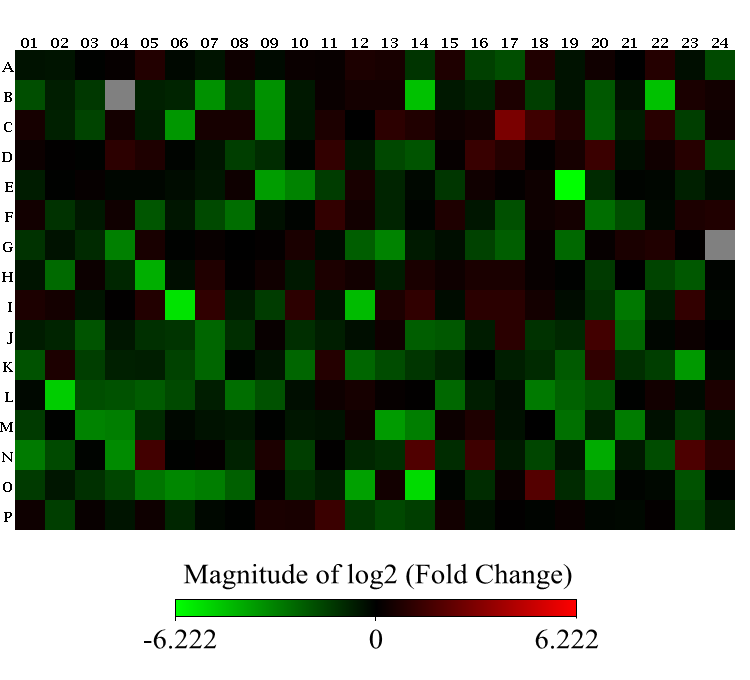


| Arrays included in Test Group: | | | | | | My5.CRBN+ Len 5 days | | | | |
| --- | --- | --- | --- | --- | --- | --- | --- | --- | --- | --- |
| Arrays included in Control Group: | | | | | | My5.CRBN+ Len 3 days | | | | |
| Genes Over-Expressed | | | | | | | | | | |
| Position | | | Mature ID | | | Fold Regulation | | | | |
| C17 | | | hsa-miR-142-3p | | | 7.8171 | | | | |
| J20 | | | hsa-miR-340-5p | | | 3.0031 | | | | |
| N05 | | | hsa-miR-520f-3p | | | 3.0332 | | | | |
| N14 | | | hsa-miR-551b-3p | | | 3.9164 | | | | |
| N23 | | | hsa-miR-589-3p | | | 3.6209 | | | | |
| O18 | | | hsa-miR-802 | | | 4.1198 | | | | |
| Genes Under-Expressed | | | | | | | | | | |
| Position | | | Mature ID | | | Fold Regulation | | | | |
| A17 | | | hsa-miR-106a-3p | | | -3.6786 | | | | |
| A24 | | | hsa-miR-10b-3p | | | -3.5461 | | | | |
| B01 | | | hsa-miR-1180-3p | | | -3.7647 | | | | |
| B07 | | | hsa-miR-124-3p | | | -12.006 | | | | |
| B09 | | | hsa-miR-125a-3p | | | -11.6521 | | | | |
| B14 | | | hsa-miR-1265 | | | -26.1642 | | | | |
| B20 | | | hsa-miR-1290 | | | -4.3671 | | | | |
| B22 | | | hsa-miR-129-5p | | | -26.7263 | | | | |
| C03 | | | hsa-miR-1324 | | | -3.1202 | | | | |
| C06 | | | hsa-miR-134-5p | | | -12.9913 | | | | |
| C09 | | | hsa-miR-136-5p | | | -11.0935 | | | | |
| C20 | | | hsa-miR-143-5p | | | -4.7996 | | | | |
| D13 | | | hsa-miR-155-5p | | | -3.3559 | | | | |
| D14 | | | hsa-miR-155-3p | | | -4.1885 | | | | |
| D24 | | | hsa-miR-181c-3p | | | -3.1383 | | | | |
| E09 | | | hsa-miR-186-3p | | | -14.5196 | | | | |
| E10 | | | hsa-miR-187-3p | | | -9.3699 | | | | |
| E19 | | | hsa-miR-191-3p | | | -74.6602 | | | | |
| F05 | | | hsa-miR-195-3p | | | -4.2512 | | | | |
| F07 | | | hsa-miR-196a-3p | | | -3.4891 | |  |  |  |
| F08 | | | hsa-miR-196b-5p | | | -6.499 | |  |  |  |
| F17 | | | hsa-miR-200a-5p | | | -3.8266 | |  |  |  |
| F20 | | | hsa-miR-200c-5p | | | -6.5274 | |  |  |  |
| F21 | | | hsa-miR-202-3p | | | -3.6919 | |  |  |  |
| G04 | | | hsa-miR-208b-3p | | | -8.5937 | |  |  |  |
| G12 | | | hsa-miR-211-5p | | | -4.8984 | |  |  |  |
| G13 | | | hsa-miR-212-3p | | | -9.3962 | |  |  |  |
| G16 | | | hsa-miR-216a-5p | | | -3.0922 | |  |  |  |
| G17 | | | hsa-miR-217 | | | -4.8265 | |  |  |  |
| G19 | | | hsa-miR-218-1-3p | | | -5.8348 | |  |  |  |
| H02 | | | hsa-miR-222-5p | | | -6.0132 | |  |  |  |
| H05 | | | hsa-miR-224-5p | | | -19.685 | |  |  |  |
| H22 | | | hsa-miR-296-3p | | | -3.1648 | |  |  |  |
| H23 | | | hsa-miR-298 | | | -4.3844 | |  |  |  |
| I06 | | | hsa-miR-300 | | | -47.4507 | |  |  |  |
| I12 | | | hsa-miR-302c-3p | | | -23.949 | |  |  |  |
| I21 | | | hsa-miR-31-5p | | | -7.7326 | |  |  |  |
| J03 | | | hsa-miR-323b-5p | | | -4.1702 | |  |  |  |
| J07 | | | hsa-miR-326 | | | -5.542 | |  |  |  |
| J14 | | | hsa-miR-338-3p | | | -4.8448 | |  |  |  |
| J15 | | | hsa-miR-338-5p | | | -4.5006 | |  |  |  |
| J21 | | | hsa-miR-340-3p | | | -5.5584 | |  |  |  |
| K01 | | | hsa-miR-346 | | | -4.0014 | |  |  |  |
| K06 | | | hsa-miR-34c-3p | | | -3.0479 | |  |  |  |
| K07 | | | hsa-miR-34c-5p | | | -5.6218 | |  |  |  |
| K10 | | | hsa-miR-363-5p | | | -5.6261 | |  |  |  |
| K12 | | | hsa-miR-370-3p | | | -5.6233 | |  |  |  |
| K13 | | | hsa-miR-372-3p | | | -3.6332 | |  |  |  |
| K19 | | | hsa-miR-376a-3p | | | -4.5906 | |  |  |  |
| K23 | | | hsa-miR-377-5p | | | -13.4837 | |  |  |  |
| L02 | | | hsa-miR-379-5p | | | -33.1298 | |  |  |  |
| L03 | | | hsa-miR-381-3p | | | -3.7163 | |  |  |  |
| L04 | | | hsa-miR-382-5p | | | -4.0694 | |  |  |  |
| L05 | | | hsa-miR-383-5p | | | -4.816 | |  |  |  |
| L06 | | | hsa-miR-409-3p | | | -3.4773 | |  |  |  |
| L08 | | | hsa-miR-411-5p | | | -6.3808 | |  |  |  |
| L09 | | | hsa-miR-421 | | | -3.9372 | |  |  |  |
| L15 | | | hsa-miR-425-3p | | | -5.8729 | |  |  |  |
| L18 | | | hsa-miR-431-5p | | | -7.9057 | |  |  |  |
| L19 | | | hsa-miR-432-5p | | | -5.1985 | |  |  |  |
| L20 | | | hsa-miR-433-3p | | | -4.0106 | |  |  |  |
| M03 | | | hsa-miR-485-5p | | | -9.417 | |  |  |  |
| M04 | | | hsa-miR-486-3p | | | -8.4575 | |  |  |  |
| M13 | | | hsa-miR-498 | | | -14.1381 | |  |  |  |
| M14 | | | hsa-miR-499a-3p | | | -8.3647 | |  |  |  |
| M19 | | | hsa-miR-506-3p | | | -6.553 | |  |  |  |
| M21 | | | hsa-miR-509-3p | | | -8.183 | |  |  |  |
| N01 | | | hsa-miR-514a-3p | | | -7.8141 | |  |  |  |
| N02 | | | hsa-miR-517b-3p | | | -3.4629 | |  |  |  |
| N04 | | | hsa-miR-519d-3p | | | -10.5273 | |  |  |  |
| N18 | | | hsa-miR-575 | | | -3.2621 | |  |  |  |
| N20 | | | hsa-miR-581 | | | -18.0513 | |  |  |  |
| N22 | | | hsa-miR-588 | | | -3.6197 | |  |  |  |
| O04 | | | hsa-miR-622 | | | -3.2815 | |  |  |  |
| O05 | | | hsa-miR-626 | | | -7.3457 | |  |  |  |
| O06 | | | hsa-miR-639 | | | -10.0681 | |  |  |  |
| O07 | | | hsa-miR-643 | | | -8.558 | |  |  |  |
| O08 | | | hsa-miR-649 | | | -5.0685 | |  |  |  |
| O12 | | | hsa-miR-708-5p | | | -15.6701 | |  |  |  |
| O14 | | | hsa-miR-720 | | | -41.5868 | |  |  |  |
| O20 | | | hsa-miR-888-5p | | | -5.9821 | |  |  |  |
| O23 | | | hsa-miR-920 | | | -3.9792 | |  |  |  |
| P13 | | | cel-miR-39-3p | | | -3.3282 | |  |  |  |

(c)

**Figure S6P. Scatter plot (a) and heat-map (b) of My5.CRBN.AGO2 + Len 3 days vs My5.CRBN+ Len 5 days& the miRNAs up- or down-regulated in this pair [c]**

(b)

(a)


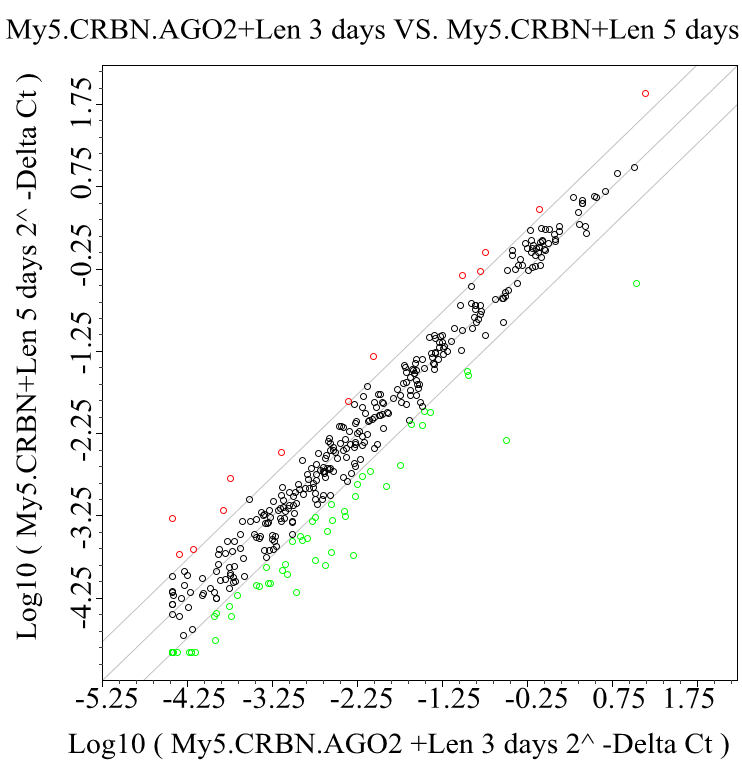

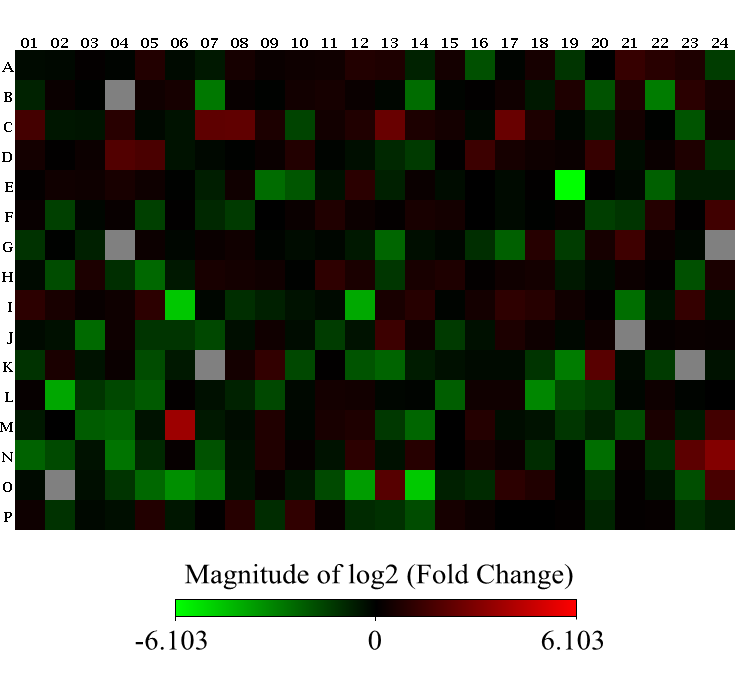


| Arrays included in Test Group: | | My5.CRBN+ Len 5 days | |
| --- | --- | --- | --- |
| Arrays included in Control Group: | | My5.CRBN.AGO2+ Len 3 days | |
| Genes Over-Expressed | | | |
| Position | Mature ID | Fold Regulation | |
| C01 | hsa-miR-132-3p | 3.1315 | |
| C07 | hsa-miR-135a-5p | 4.8126 | |
| C08 | hsa-miR-135b-5p | 4.8892 | |
| C13 | hsa-miR-139-5p | 5.6207 | |
| C17 | hsa-miR-142-3p | 5.5303 | |
| D04 | hsa-miR-148a-3p | 3.8465 | |
| D05 | hsa-miR-148b-3p | 3.3711 | |
| K20 | hsa-miR-376b-3p | 4.3745 | |
| M06 | hsa-miR-488-3p | 13.7206 | |
| N23 | hsa-miR-589-3p | 4.5898 | |
| N24 | hsa-miR-600 | 9.0737 | |
| O13 | hsa-miR-708-3p | 4.1613 | |
| O24 | hsa-miR-924 | 3.2947 | |
| Genes Under-Expressed | | | |
| Position | Mature ID | Fold Regulation | |
| A16 | hsa-miR-105-5p | -3.8186 | |
| B04 | hsa-miR-122-3p | -3.083 | |
| B07 | hsa-miR-124-3p | -7.2124 | |
| B14 | hsa-miR-1265 | -6.03 | |
| B20 | hsa-miR-1290 | -3.9245 | |
| B22 | hsa-miR-129-5p | -7.9018 | |
| C10 | hsa-miR-137 | -3.1375 | |
| C23 | hsa-miR-145-5p | -4.0376 | |
| E09 | hsa-miR-186-3p | -6.0031 | |
| E10 | hsa-miR-187-3p | -4.1688 | |
| E19 | hsa-miR-191-3p | -68.7535 | |
| E22 | hsa-miR-192-3p | -4.8906 | |
| G04 | hsa-miR-208b-3p | -3.083 |  |
| G13 | hsa-miR-212-3p | -5.4427 |  |
| G17 | hsa-miR-217 | -4.8794 |  |
| G24 | hsa-miR-221-5p | -3.083 |  |
| H02 | hsa-miR-222-5p | -3.5806 |  |
| H05 | hsa-miR-224-5p | -5.7023 |  |
| H23 | hsa-miR-298 | -3.7961 |  |
| I06 | hsa-miR-300 | -27.5933 |  |
| I12 | hsa-miR-302c-3p | -16.9219 |  |
| I21 | hsa-miR-31-5p | -6.2012 |  |
| J03 | hsa-miR-323b-5p | -5.7499 |  |
| J07 | hsa-miR-326 | -3.3105 |  |
| J21 | hsa-miR-340-3p | -3.083 |  |
| K05 | hsa-miR-34b-5p | -3.5829 |  |
| K07 | hsa-miR-34c-5p | -3.083 |  |
| K10 | hsa-miR-363-5p | -3.2895 |  |
| K12 | hsa-miR-370-3p | -4.0269 |  |
| K13 | hsa-miR-372-3p | -5.1899 |  |
| K19 | hsa-miR-376a-3p | -7.6994 |  |
| K23 | hsa-miR-377-5p | -3.083 |  |
| L02 | hsa-miR-379-5p | -16.3246 |  |
| L04 | hsa-miR-382-5p | -3.331 |  |
| L05 | hsa-miR-383-5p | -4.5189 |  |
| L09 | hsa-miR-421 | -3.3149 |  |
| L15 | hsa-miR-425-3p | -4.7377 |  |
| L18 | hsa-miR-431-5p | -9.4842 |  |
| L19 | hsa-miR-432-5p | -3.4453 |  |
| M03 | hsa-miR-485-5p | -4.5506 |  |
| M04 | hsa-miR-486-3p | -5.1397 |  |
| M14 | hsa-miR-499a-3p | -5.4717 |  |
| M21 | hsa-miR-509-3p | -3.4837 |  |
| N01 | hsa-miR-514a-3p | -5.0766 |  |
| N02 | hsa-miR-517b-3p | -3.4603 |  |
| N04 | hsa-miR-519d-3p | -6.8365 |  |
| N07 | hsa-miR-522-3p | -3.9071 |  |
| N20 | hsa-miR-581 | -6.2962 |  |
| O02 | hsa-miR-606 | -3.083 |  |
| O05 | hsa-miR-626 | -5.5844 |  |
| O06 | hsa-miR-639 | -11.0301 |  |
| O07 | hsa-miR-643 | -6.9042 |  |
| O11 | hsa-miR-7-5p | -3.428 |  |
| O12 | hsa-miR-708-5p | -13.9474 |  |
| O14 | hsa-miR-720 | -28.7971 |  |
| O23 | hsa-miR-920 | -3.5894 |  |
| P14 | cel-miR-39-3p | -3.5069 |  |

(c)

**Figure S6Q. Scatter plot (a) and heat-map (b) of My5.CRBN vs My5.CRBN + Len 3 days& the miRNAs up- or down-regulated in this pair [c].**

(b)

(a)


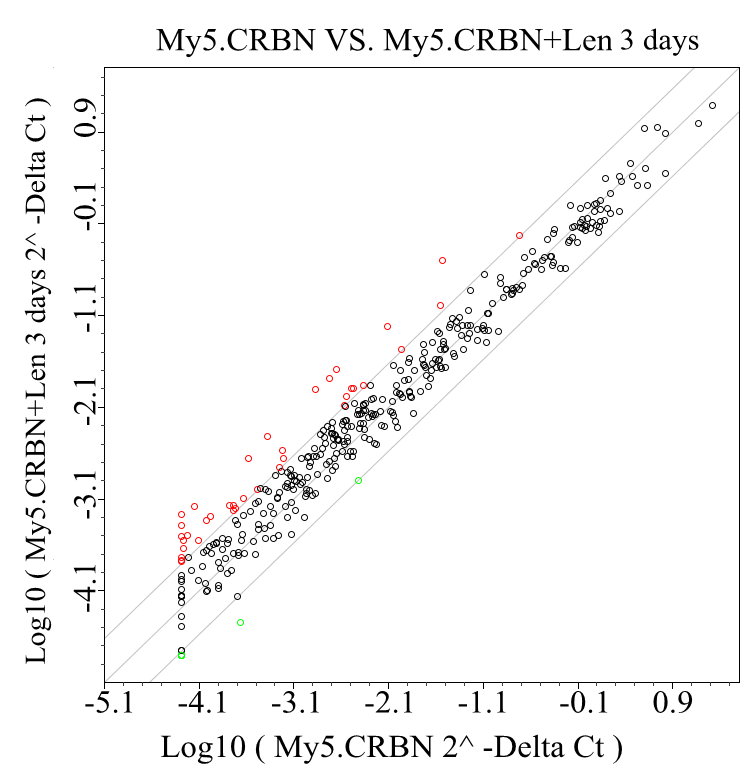

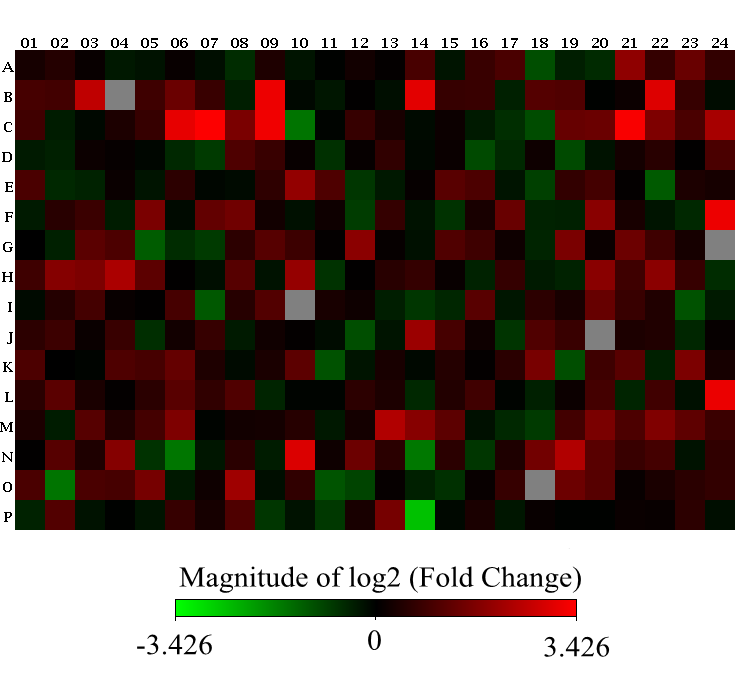


| Arrays included in Test Group: | | My5.CRBN+ Len 3 days | |
| --- | --- | --- | --- |
| Arrays included in Control Group: | | My5.CRBN | |
| Genes Over-Expressed | | | |
| Position | Mature ID | Fold Regulation | |
| A21 | hsa-miR-10a-5p | 3.806 | |
| B03 | hsa-miR-122-5p | 5.9576 | |
| B09 | hsa-miR-125a-3p | 9.2212 | |
| B14 | hsa-miR-1265 | 8.4053 | |
| B22 | hsa-miR-129-5p | 7.9275 | |
| C06 | hsa-miR-134-5p | 8.7294 | |
| C07 | hsa-miR-135a-5p | 10.751 | |
| C08 | hsa-miR-135b-5p | 3.1261 | |
| C09 | hsa-miR-136-5p | 9.4734 | |
| C21 | hsa-miR-144-3p | 10.3389 | |
| C22 | hsa-miR-144-5p | 3.2131 | |
| C24 | hsa-miR-145-3p | 4.8071 | |
| E10 | hsa-miR-187-3p | 3.9392 | |
| F05 | hsa-miR-195-3p | 3.1379 | |
| F20 | hsa-miR-200c-5p | 3.6067 | |
| F24 | hsa-miR-205-5p | 9.1183 | |
| G12 | hsa-miR-211-5p | 3.6796 | |
| G19 | hsa-miR-218-1-3p | 3.1411 | |
| H02 | hsa-miR-222-5p | 3.4937 | |
| H03 | hsa-miR-223-3p | 3.153 | |
| H04 | hsa-miR-223-5p | 4.9434 | |
| H10 | hsa-miR-23b-5p | 4.0199 | |
| H20 | hsa-miR-28-3p | 3.6308 | |
| H22 | hsa-miR-296-3p | 3.6544 | |
| J14 | hsa-miR-338-3p | 4.2376 | |
| K18 | hsa-miR-375 | 3.0509 | |
| K23 | hsa-miR-377-5p | 3.1674 | |
| L24 | hsa-miR-455-5p | 9.0809 |  |
| M06 | hsa-miR-488-3p | 3.2252 |  |
| M13 | hsa-miR-498 | 5.2252 |  |
| M14 | hsa-miR-499a-3p | 3.5641 |  |
| M20 | hsa-miR-508-5p | 3.0936 |  |
| M22 | hsa-miR-511-5p | 3.305 |  |
| N04 | hsa-miR-519d-3p | 3.4941 |  |
| N10 | hsa-miR-539-5p | 7.7917 |  |
| N19 | hsa-miR-580-3p | 5.2748 |  |
| O08 | hsa-miR-649 | 4.4453 |  |
| Genes Under-Expressed | | |  |
| Position | Mature ID | Fold Regulation |  |
| B04 | hsa-miR-122-3p | -3.2975 |  |
| G24 | hsa-miR-221-5p | -3.2975 |  |
| I10 | hsa-miR-302b-3p | -3.2975 |  |
| J20 | hsa-miR-340-5p | -3.2975 |  |
| N14 | hsa-miR-551b-3p | -3.0516 |  |
| O18 | hsa-miR-802 | -3.2975 |  |
| P14 | cel-miR-39-3p | -6.1109 |  |

**Figure S6R. Scatter plot (a) and heat-map (b) of My5.LV + Len 3 days vs My5.CRBN + Len 3 days & the miRNAs up- or down-regulated in this pair [c].**

(b)

(a)


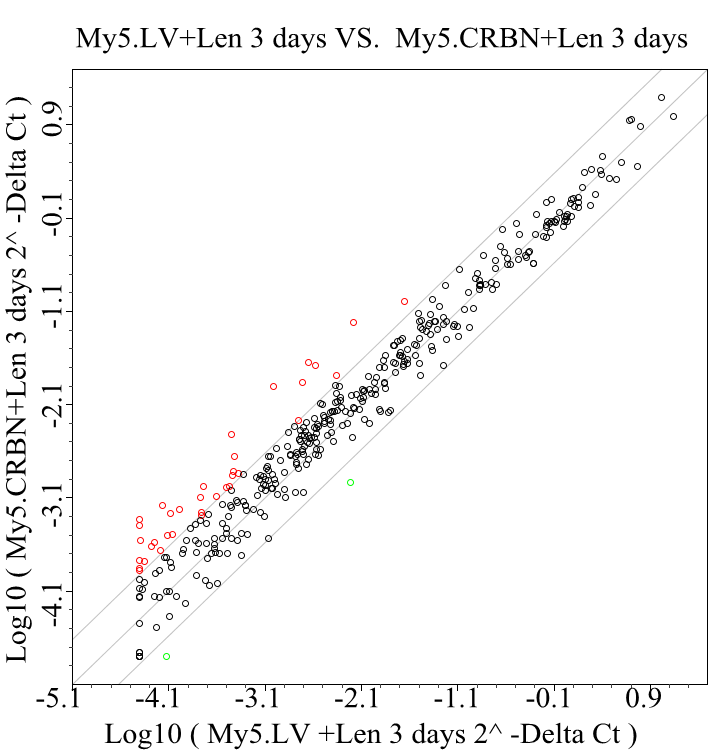

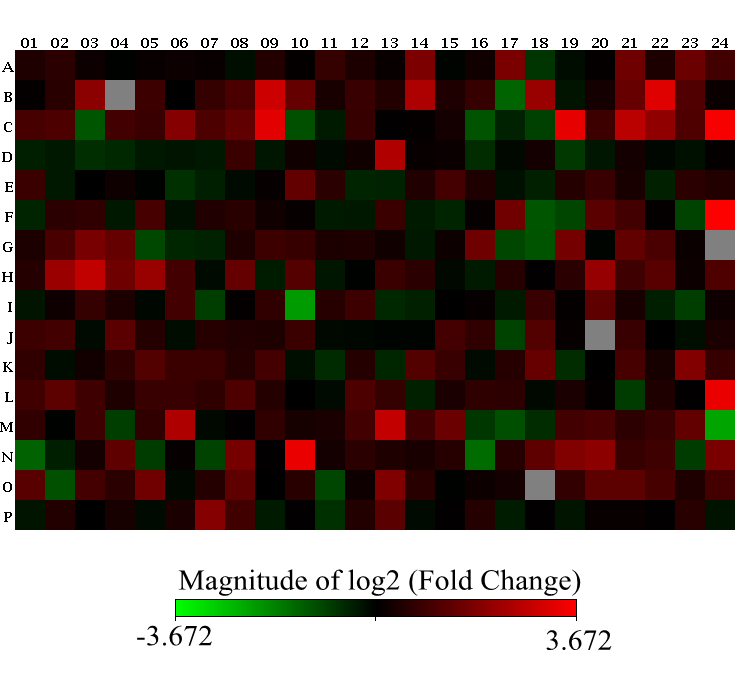


| Arrays included in Test Group: | | | | | | My5.CRBN+ Len 3 days | | |
| --- | --- | --- | --- | --- | --- | --- | --- | --- |
| Arrays included in Control Group: | | | | | | My5.LV+ Len 3 days | | |
| Genes Over-Expressed | | | | | | | | |
| Position | | | Mature ID | | | Fold Regulation | | |
| A14 | | | hsa-miR-101-5p | | | 3.4476 | | |
| A17 | | | hsa-miR-106a-3p | | | 3.3664 | | |
| B03 | | | hsa-miR-122-5p | | | 4.0641 | | |
| B09 | | | hsa-miR-125a-3p | | | 7.7684 | | |
| B14 | | | hsa-miR-1265 | | | 5.6956 | | |
| B18 | | | hsa-miR-1284 | | | 4.6839 | | |
| B22 | | | hsa-miR-129-5p | | | 9.2759 | | |
| C06 | | | hsa-miR-134-5p | | | 3.781 | | |
| C09 | | | hsa-miR-136-5p | | | 9.6842 | | |
| C19 | | | hsa-miR-143-3p | | | 10.1971 | | |
| C21 | | | hsa-miR-144-3p | | | 6.5634 | | |
| C22 | | | hsa-miR-144-5p | | | 4.3053 | | |
| C24 | | | hsa-miR-145-3p | | | 11.8268 | | |
| D13 | | | hsa-miR-155-5p | | | 5.8955 | | |
| F17 | | | hsa-miR-200a-5p | | | 3.0651 | | |
| F24 | | | hsa-miR-205-5p | | | 12.7451 | | |
| G03 | | | hsa-miR-208a-3p | | | 3.3315 | | |
| G19 | | | hsa-miR-218-1-3p | | | 3.2097 | | |
| H02 | | | hsa-miR-222-5p | | | 4.7249 | | |
| H03 | | | hsa-miR-223-3p | | | 6.9066 | | |
| H04 | | | hsa-miR-223-5p | | | 3.0072 | | |
| H05 | | | hsa-miR-224-5p | | | 4.6152 | | |
| H20 | | | hsa-miR-28-3p | | | 4.5151 | | |
| K23 | | | hsa-miR-377-5p | | | 3.7085 | | |
| L24 | | | hsa-miR-455-5p | | | 10.507 | | |
| M06 | | | hsa-miR-488-3p | | | 5.8244 | | |
| M13 | | | hsa-miR-498 | | | 7.0122 | | |
| N08 | | | hsa-miR-524-5p | | | 3.223 | | |
| N10 | | | hsa-miR-539-5p | | | 10.4401 | | |
| N19 | | | hsa-miR-580-3p | | | 3.6807 | | |
| N20 | | | hsa-miR-581 | | | 4.0936 | | |
| N24 | | | hsa-miR-600 | | | 3.3604 | | |
| O05 | | | hsa-miR-626 | | | 3.068 | | |
| O13 | | | hsa-miR-708-3p | | | 3.5876 | | |
| P07 | | | hsa-miR-95-3p | | | 3.8003 | | |
| Genes Under-Expressed | | | | | | | | |
| Position | | | Mature ID | | | Fold Regulation | | |
| I10 | | | Has-miR-302b-3p | | | -4.7698 | | |
| M24 | | | Has-miR-513a-5p | | | -5.2915 | | |

(c)

**Figure S6S. Scatter plot (a) and heat-map (b) of My5.LV + Len 5days vs My5.CRBN + Len 5 days & the miRNAs up- or down-regulated in this pair [c].**


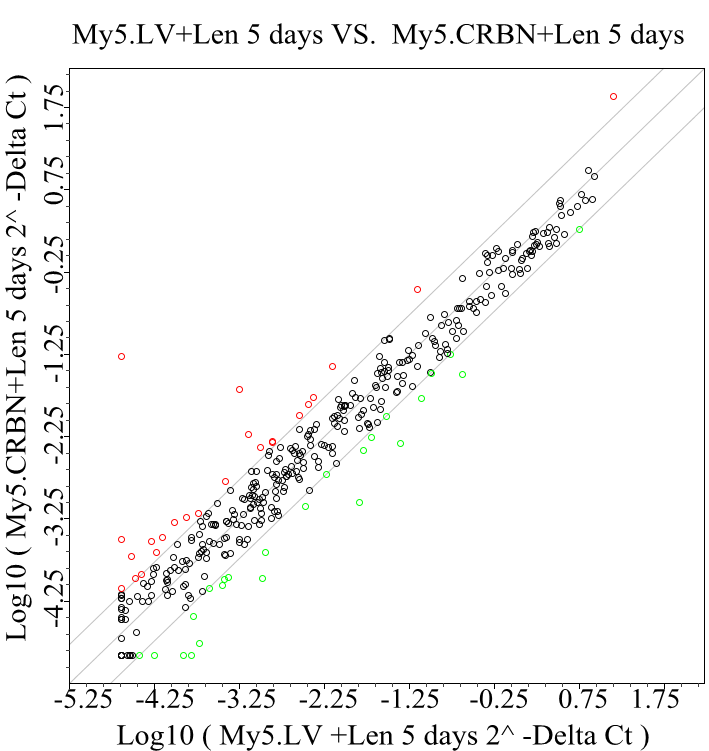

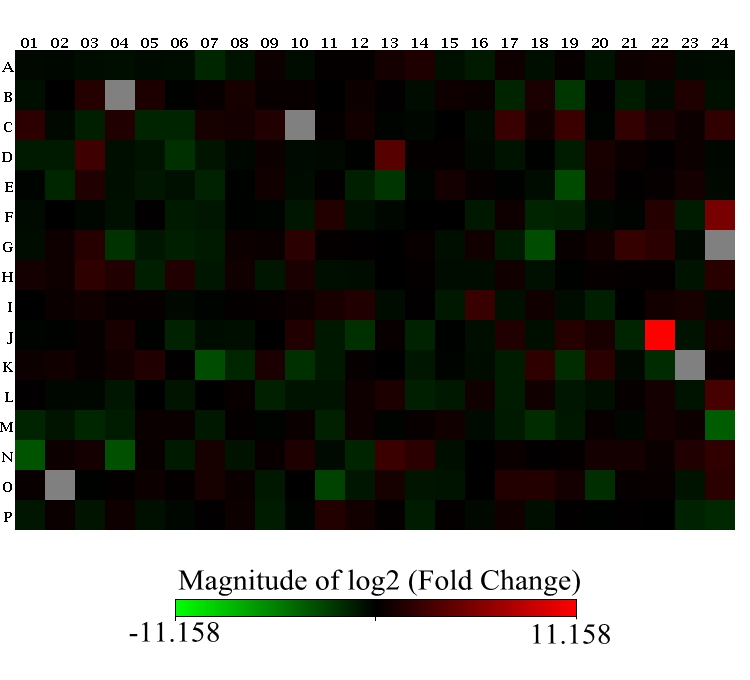


| Arrays included in Test Group: | | My5.CRBN+ Len 5 days |
| --- | --- | --- |
| Arrays included in Control Group: | | My5.LV+ Len 5 days |
| Genes Over-Expressed | | |
| Position | Mature ID | Fold Regulation |

| B03 | hsa-miR-122-5p | 3.0265 |
| --- | --- | --- |
| C01 | hsa-miR-132-3p | 3.7628 |
| C17 | hsa-miR-142-3p | 5.3823 |
| C19 | hsa-miR-143-3p | 5.7207 |
| C21 | hsa-miR-144-3p | 4.6953 |
| C24 | hsa-miR-145-3p | 4.3696 |
| D03 | hsa-miR-147a | 6.3712 |
| D13 | hsa-miR-155-5p | 13.8244 |
| F24 | hsa-miR-205-5p | 37.3879 |
| G03 | hsa-miR-208a-3p | 3.1341 |
| G10 | hsa-miR-21-3p | 3.4993 |
| G21 | hsa-miR-22-3p | 4.922 |
| G22 | hsa-miR-22-5p | 3.4955 |
| H03 | hsa-miR-223-3p | 4.039 |
| H24 | hsa-miR-299-5p | 3.4601 |
| I16 | hsa-miR-30c-1-3p | 5.305 |
| J22 | hsa-miR-342-3p | 2285.4906 |
| K18 | hsa-miR-375 | 4.1088 |
| K20 | hsa-miR-376b-3p | 3.5273 |
| L24 | hsa-miR-455-5p | 8.5074 |
| N13 | hsa-miR-549a | 5.7766 |
| N14 | hsa-miR-551b-3p | 3.5961 |
| N24 | hsa-miR-600 | 4.2232 |
| O24 | hsa-miR-924 | 3.6651 |

| Genes Under-Expressed | | |
| --- | --- | --- |
| Position | Mature ID | Fold Regulation |

| A07 | hsa-let-7f-5p | -3.1095 |
| --- | --- | --- |
| B19 | hsa-miR-129-1-3p | -5.2579 |
| C05 | hsa-miR-133b | -3.0356 |
| D06 | hsa-miR-149-5p | -4.1953 |
| E02 | hsa-miR-182-5p | -3.1366 |
| E13 | hsa-miR-18a-3p | -4.7838 |
| E19 | hsa-miR-191-3p | -9.5385 |
| G04 | hsa-miR-208b-3p | -4.5669 |
| G18 | hsa-miR-218-5p | -9.8381 |
| J12 | hsa-miR-335-3p | -4.3245 |
| J21 | hsa-miR-340-3p | -3.0212 |
| K07 | hsa-miR-34c-5p | -10.1175 |
| K08 | hsa-miR-361-5p | -3.0839 |
| K10 | hsa-miR-363-5p | -4.3181 |
| K19 | hsa-miR-376a-3p | -3.8574 |
| K22 | hsa-miR-377-3p | -3.6771 |
| M01 | hsa-miR-483-5p | -3.0111 |
| M03 | hsa-miR-485-5p | -3.122 |
| M18 | hsa-miR-505-3p | -3.7152 |
| M24 | hsa-miR-513a-5p | -16.3921 |
| N01 | hsa-miR-514a-3p | -12.5538 |
| N04 | hsa-miR-519d-3p | -11.029 |
| O11 | hsa-miR-7-5p | -7.3589 |
| O20 | hsa-miR-888-5p | -4.0183 |

(c)

(a)

(b)
