# Supplementary material for: Expression of the cereblon binding protein argonaute 2 plays an important role for multiple myeloma cell growth and survival
Source: BMC Cancer. 2016 May 3;16:297. doi: 10.1186/s12885-016-2331-0 (PMC4855823; doi:10.1186/s12885-016-2331-0)
Supplement: Additional file 2: Table S1. — Sequence of human AGO2 small hairpin RNA (shRNA). Table S2. Proteins detected by tandem mass spectrometry analyses. Table S3. The miRNA up- or down-regulated by AGO2 shRNA. Table S4. The miRNA up- or down-regulated by lenalidomide treatment. (DOCX 159 kb) [file 12885_2016_2331_MOESM2_ESM.docx]

| Gene Symbol | Species | Clone Name | Short name | Target Sequence |
| --- | --- | --- | --- | --- |
| AGO2 | Human | NM_012154.2-1163s1c1 | sh 70 | CGGCAAGAAGAGATTAGCAAA |
| AGO2 | Human | NM_012154.2-1219s1c1 | sh 71 | \| CGTCCGTGAATTTGGAATCAT \| \| --- \| |
| AGO2 | Human | NM_012154.2-150s1c1 | sh 72 | \| CAATCAAATTACAGGCCAATT \| \| --- \| |
| AGO2 | Human | NM_012154.2-2776s1c1 | sh 73 | CCAGATTTCAAACTTGGATTT |
| AGO2 | Human | NM_012154.2-719s1c1   \|  \| \| --- \| | sh 74 | GCACAGCCAGTAATCGAGTTT |

**Table S1. Sequence of human AGO2 small hairpin RNA (shRNA)**

| #  **Table S2. Proteins detected by MS/MS analysis** | Accession Number | Molecular Weight | My5/LV | My5/CRBN |
| --- | --- | --- | --- | --- |
| 1 | K2C1_HUMAN | 66 kDa | 487 | 436 |
| 2 | K1C10_HUMAN | 59 kDa | 365 | 313 |
| 3 | K22E_HUMAN | 65 kDa | 349 | 258 |
| 4 | K1C9_HUMAN | 62 kDa | 229 | 245 |
| 5 | NONO_HUMAN | 54 kDa | 136 | 298 |
| 6 | TBB5_HUMAN | 50 kDa | 172 | 246 |
| 7 | SFPQ_HUMAN | 76 kDa | 125 | 259 |
| 8 | TBA1B_HUMAN | 50 kDa | 117 | 177 |
| 9 | LDHA_HUMAN | 37 kDa | 106 | 133 |
| 10 | DHX15_HUMAN | 91 kDa | 56 | 167 |
| 11 | LDHB_HUMAN | 37 kDa | 107 | 106 |
| 12 | K1C16_HUMAN | 51 kDa | 86 | 95 |
| 13 | K1C14_HUMAN | 52 kDa | 81 | 87 |
| 14 | TCPB_HUMAN | 57 kDa | 56 | 111 |
| 15 | P5CR3_HUMAN | 29 kDa | 96 | 79 |
| 16 | P20D2_HUMAN | 48 kDa | 55 | 88 |
| 17 | K2C6A_HUMAN | 60 kDa | 63 | 87 |
| 18 | GFPT1_HUMAN | 79 kDa | 76 | 78 |
| 19 | HSP7C_HUMAN | 71 kDa | 65 | 88 |
| 20 | SF3B1_HUMAN | 146 kDa | 46 | 96 |
| 21 | HNRPL_HUMAN | 64 kDa | 43 | 96 |
| 22 | PYR1_HUMAN | 243 kDa | 69 | 70 |
| 23 | TCPZ_HUMAN | 58 kDa | 69 | 75 |
| 24 | K2C5_HUMAN | 62 kDa | 82 | 57 |
| 25 | CHD2_HUMAN | 211 kDa | 45 | 80 |
| 26 | MYH9_HUMAN | 227 kDa | 110 | 25 |
| 27 | SF3B2_HUMAN | 98 kDa | 38 | 81 |
| 28 | TCPH_HUMAN | 59 kDa | 62 | 67 |
| 29 | TCPD_HUMAN | 58 kDa | 55 | 69 |
| 30 | SYNC_HUMAN | 63 kDa | 80 | 40 |
| 31 | UBP7_HUMAN | 128 kDa | 58 | 62 |
| 32 | CD11B_HUMAN | 93 kDa | 34 | 66 |
| 33 | PP1RA_HUMAN | 99 kDa | 44 | 83 |
| 34 | TCPQ_HUMAN | 60 kDa | 62 | 57 |
| 35 | TPR_HUMAN | 267 kDa | 56 | 70 |
| 36 | HS90A_HUMAN | 85 kDa | 57 | 62 |
| 37 | DOCK8_HUMAN | 239 kDa | 40 | 64 |
| 38 | TCPA_HUMAN | 60 kDa | 49 | 76 |
| 39 | TCPG_HUMAN | 61 kDa | 57 | 66 |
| 40 | DPP9_HUMAN | 98 kDa | 52 | 61 |
| 41 | TYY1_HUMAN | 45 kDa | 60 | 56 |
| 42 | TCPE_HUMAN | 60 kDa | 53 | 53 |
| 43 | DDX42_HUMAN | 103 kDa | 27 | 78 |
| 44 | H4_HUMAN | 11 kDa | 51 | 65 |
| 45 | PRP4B_HUMAN | 117 kDa | 17 | 88 |
| 46 | ACTG_HUMAN (+1) | 42 kDa | 55 | 49 |
| 47 | SF3B3_HUMAN | 136 kDa | 43 | 68 |
| 48 | TOP1_HUMAN | 91 kDa | 33 | 67 |
| 49 | EF1A1_HUMAN (+1) | 50 kDa | 45 | 57 |
| 50 | CBL_HUMAN | 100 kDa | 18 | 65 |
| 51 | KI67_HUMAN | 359 kDa | 56 | 34 |
| 52 | CCNT1_HUMAN | 81 kDa | 36 | 52 |
| 53 | PLSL_HUMAN | 70 kDa | 61 | 32 |
| 54 | P85A_HUMAN | 84 kDa | 44 | 39 |
| 55 | TIF1B_HUMAN | 89 kDa | 38 | 49 |
| 56 | IRF4_HUMAN | 52 kDa | 23 | 51 |
| 57 | MECP2_HUMAN | 52 kDa | 31 | 55 |
| 58 | PNPH_HUMAN | 32 kDa | 62 | 26 |
| 59 | TAF2_HUMAN | 137 kDa | 43 | 40 |
| 60 | DDB1_HUMAN | 127 kDa | 6 | 81 |
| 61 | DESP_HUMAN | 332 kDa | 27 | 39 |
| 62 | HNRPK_HUMAN | 51 kDa | 41 | 39 |
| 63 | RS15_HUMAN | 17 kDa | 22 | 41 |
| 64 | RUVB2_HUMAN | 51 kDa | 44 | 38 |
| 65 | SAFB2_HUMAN | 107 kDa | 28 | 44 |
| 66 | RS8_HUMAN | 24 kDa | 30 | 41 |
| 67 | RL18A_HUMAN | 21 kDa | 6 | 56 |
| 68 | RBM33_HUMAN | 130 kDa | 21 | 47 |
| 69 | RL3_HUMAN | 46 kDa | 15 | 48 |
| 70 | CHD8_HUMAN | 291 kDa | 34 | 31 |
| 71 | H2B1C_HUMAN (+8) | 14 kDa | 27 | 27 |
| 72 | GCN1L_HUMAN | 293 kDa | 26 | 40 |
| 73 | SF3A1_HUMAN | 89 kDa | 24 | 35 |
| 74 | LC7L2_HUMAN | 47 kDa | 21 | 52 |
| 75 | IF4A1_HUMAN | 46 kDa | 29 | 37 |
| 76 | SIN3A_HUMAN | 145 kDa | 20 | 43 |
| 77 | RUVB1_HUMAN | 50 kDa | 35 | 39 |
| 78 | U520_HUMAN | 245 kDa | 33 | 31 |
| 79 | ABCF2_HUMAN | 71 kDa | 15 | 46 |
| 80 | CH60_HUMAN | 61 kDa | 36 | 24 |
| 81 | GRP78_HUMAN | 72 kDa | 40 | 25 |
| 82 | UBIQ_HUMAN | 9 kDa | 31 | 24 |
| 83 | FAS_HUMAN | 273 kDa | 25 | 37 |
| 84 | PRP8_HUMAN | 274 kDa | 23 | 38 |
| 85 | TRIO_HUMAN | 347 kDa | 25 | 33 |
| 86 | MATR3_HUMAN | 95 kDa | 22 | 28 |
| 87 | CHD1_HUMAN | 197 kDa | 21 | 40 |
| 88 | RS3_HUMAN | 27 kDa | 24 | 35 |
| 89 | USP9X_HUMAN | 290 kDa | 15 | 37 |
| 90 | ENOA_HUMAN | 47 kDa | 39 | 17 |
| 91 | H14_HUMAN | 22 kDa | 43 | 21 |
| 92 | UBP34_HUMAN | 404 kDa | 40 | 18 |
| 93 | YTDC1_HUMAN | 85 kDa | 11 | 35 |
| 94 | NFKB1_HUMAN | 105 kDa | 27 | 25 |
| 95 | RL13_HUMAN | 24 kDa | 21 | 37 |
| 96 | MTAP_HUMAN | 31 kDa | 30 | 32 |
| 97 | PARP1_HUMAN | 113 kDa | 41 | 13 |
| 98 | WDR82_HUMAN | 35 kDa | 18 | 33 |
| 99 | HNRPF_HUMAN | 46 kDa | 22 | 29 |
| 100 | ACTL8_HUMAN | 41 kDa | 22 | 34 |
| 101 | TLN1_HUMAN | 270 kDa | 45 | 14 |
| 102 | HS90B_HUMAN | 83 kDa | 24 | 28 |
| 103 | ARK72_HUMAN | 40 kDa | 23 | 34 |
| 104 | PR40A_HUMAN | 109 kDa | 17 | 30 |
| 105 | RL10_HUMAN | 25 kDa | 14 | 28 |
| 106 | U5S1_HUMAN | 109 kDa | 26 | 21 |
| 107 | RBM25_HUMAN | 100 kDa | 15 | 27 |
| 108 | HNRPM_HUMAN | 78 kDa | 33 | 21 |
| 109 | RS4X_HUMAN | 30 kDa | 21 | 29 |
| 110 | S30BP_HUMAN | 34 kDa | 17 | 27 |
| 111 | TOP2B_HUMAN | 183 kDa | 19 | 29 |
| 112 | EFTU_HUMAN | 50 kDa | 14 | 33 |
| 113 | ACTN4_HUMAN | 105 kDa | 27 | 22 |
| 114 | GBF1_HUMAN | 206 kDa | 18 | 30 |
| 115 | SYMPK_HUMAN | 141 kDa | 14 | 35 |
| 116 | TBB2C_HUMAN | 50 kDa | 24 | 32 |
| 117 | NFRKB_HUMAN | 139 kDa | 25 | 18 |
| 118 | PP1A_HUMAN | 38 kDa | 18 | 28 |
| 119 | RS3A_HUMAN | 30 kDa | 16 | 28 |
| 120 | PLEC1_HUMAN | 532 kDa | 14 | 31 |
| 121 | PSPC1_HUMAN | 59 kDa | 9 | 36 |
| 122 | TOX4_HUMAN | 66 kDa | 20 | 30 |
| 123 | CPSF1_HUMAN | 161 kDa | 16 | 29 |
| 124 | SR140_HUMAN | 118 kDa | 15 | 28 |
| 125 | VIR_HUMAN | 202 kDa | 8 | 38 |
| 126 | CCNT2_HUMAN | 81 kDa | 12 | 31 |
| 127 | SMRC2_HUMAN | 133 kDa | 22 | 24 |
| 128 | CHERP_HUMAN | 104 kDa | 7 | 19 |
| 129 | PRDX1_HUMAN | 22 kDa | 16 | 23 |
| 130 | TR150_HUMAN | 109 kDa | 16 | 26 |
| 131 | GTF2I_HUMAN | 112 kDa | 39 | 6 |
| 132 | H31T_HUMAN (+3) | 16 kDa | 22 | 17 |
| 133 | DSG1_HUMAN | 114 kDa | 19 | 20 |
| 134 | HSPB1_HUMAN | 23 kDa | 20 | 22 |
| 135 | PCBP1_HUMAN | 37 kDa | 22 | 24 |
| 136 | K2C71_HUMAN | 57 kDa | 25 | 18 |
| 137 | K1C17_HUMAN | 48 kDa | 17 | 23 |
| 138 | MIO_HUMAN | 99 kDa | 18 | 27 |
| 139 | SK2L2_HUMAN | 118 kDa | 12 | 30 |
| 140 | RL27A_HUMAN | 17 kDa | 10 | 30 |
| 141 | ELP1_HUMAN | 150 kDa | 16 | 25 |
| 142 | LMNA_HUMAN | 74 kDa | 15 | 29 |
| 143 | LRCH1_HUMAN | 81 kDa | 24 | 22 |
| 144 | ATX2L_HUMAN | 113 kDa | 14 | 25 |
| 145 | CNDP2_HUMAN | 53 kDa | 32 | 10 |
| 146 | EF1G_HUMAN | 50 kDa | 19 | 22 |
| 147 | RB15B_HUMAN | 97 kDa | 12 | 26 |
| 148 | SPTA2_HUMAN | 285 kDa | 23 | 13 |
| 149 | TRA2B_HUMAN | 34 kDa | 11 | 28 |
| 150 | CA128_HUMAN | 24 kDa | 11 | 29 |
| 151 | MEPCE_HUMAN | 74 kDa | 9 | 32 |
| 152 | RL4_HUMAN | 48 kDa | 14 | 22 |
| 153 | RS2_HUMAN | 31 kDa | 6 | 25 |
| 154 | GEPH_HUMAN | 80 kDa | 13 | 21 |
| 155 | HNRH2_HUMAN | 49 kDa | 14 | 26 |
| 156 | K2C1B_HUMAN | 62 kDa | 25 | 14 |
| 157 | OSB11_HUMAN | 84 kDa | 23 | 16 |
| 158 | RL21_HUMAN | 19 kDa | 10 | 24 |
| 159 | SAFB1_HUMAN | 103 kDa | 17 | 21 |
| 160 | SMC2_HUMAN | 136 kDa | 26 | 15 |
| 161 | FBRL_HUMAN | 34 kDa | 17 | 19 |
| 162 | SF01_HUMAN | 68 kDa | 11 | 21 |
| 163 | SMCA5_HUMAN | 122 kDa | 16 | 20 |
| 164 | MYCB2_HUMAN | 510 kDa | 26 | 2 |
| 165 | CCNL1_HUMAN | 60 kDa | 12 | 18 |
| 166 | DDX24_HUMAN | 96 kDa | 8 | 23 |
| 167 | FLNA_HUMAN | 281 kDa | 16 | 17 |
| 168 | G3P_HUMAN | 36 kDa | 15 | 12 |
| 169 | NUFP2_HUMAN | 76 kDa | 22 | 14 |
| 170 | PSME1_HUMAN | 29 kDa | 24 | 14 |
| 171 | RBP2_HUMAN | 358 kDa | 16 | 21 |
| 172 | ENPL_HUMAN | 92 kDa | 25 | 14 |
| 173 | K6PL_HUMAN | 85 kDa | 9 | 28 |
| 174 | RS6_HUMAN | 29 kDa | 9 | 24 |
| 175 | RS9_HUMAN | 23 kDa | 10 | 24 |
| 176 | SMCA2_HUMAN | 181 kDa | 19 | 17 |
| 177 | FL2D_HUMAN | 44 kDa | 9 | 32 |
| 178 | KPYM_HUMAN | 58 kDa | 17 | 20 |
| 179 | NOP56_HUMAN | 66 kDa | 12 | 11 |
| 180 | SPTB2_HUMAN | 275 kDa | 27 | 5 |
| 181 | TAF5_HUMAN | 87 kDa | 14 | 23 |
| 182 | MMTA2_HUMAN | 29 kDa | 6 | 26 |
| 183 | H2AV_HUMAN (+1) | 14 kDa | 14 | 20 |
| 184 | BRD4_HUMAN | 152 kDa | 8 | 22 |
| 185 | CPSF2_HUMAN | 88 kDa | 7 | 21 |
| 186 | HDAC2_HUMAN | 55 kDa | 18 | 18 |
| 187 | IMB1_HUMAN | 97 kDa | 18 | 17 |
| 188 | UCHL5_HUMAN | 38 kDa | 19 | 14 |
| 189 | ALDOA_HUMAN | 39 kDa | 18 | 13 |
| 190 | CYFP2_HUMAN | 148 kDa | 17 | 16 |
| 191 | DNJA1_HUMAN | 45 kDa | 10 | 13 |
| 192 | PLAK_HUMAN | 82 kDa | 15 | 19 |
| 193 | RL17_HUMAN | 21 kDa | 14 | 18 |
| 194 | SMC4_HUMAN | 147 kDa | 20 | 12 |
| 195 | TAF6_HUMAN | 73 kDa | 21 | 12 |
| 196 | TERA_HUMAN | 89 kDa | 23 | 7 |
| 197 | 1433E_HUMAN | 29 kDa | 20 | 14 |
| 198 | ANXA2_HUMAN | 39 kDa | 21 | 12 |
| 199 | CLH1_HUMAN | 192 kDa | 13 | 17 |
| 200 | DOCK4_HUMAN | 225 kDa | 15 | 12 |
| 201 | FIP1_HUMAN | 67 kDa | 16 | 19 |
| 202 | IPO5_HUMAN | 124 kDa | 12 | 19 |
| 203 | PRKDC_HUMAN | 469 kDa | 17 | 13 |
| 204 | SLTM_HUMAN | 117 kDa | 7 | 21 |
| 205 | CRBN_HUMAN | 51 kDa | 0 | 28 |
| 206 | ARK73_HUMAN | 37 kDa | 16 | 15 |
| 207 | ARP8_HUMAN | 70 kDa | 15 | 8 |
| 208 | BAT3_HUMAN | 119 kDa | 14 | 18 |
| 209 | KU70_HUMAN | 70 kDa | 20 | 11 |
| 210 | LARP7_HUMAN | 67 kDa | 14 | 12 |
| 211 | UAP56_HUMAN | 49 kDa | 9 | 17 |
| 212 | XPO1_HUMAN | 123 kDa | 9 | 17 |
| 213 | ALBU_HUMAN | 69 kDa | 15 | 16 |
| 214 | CNOT1_HUMAN | 267 kDa | 15 | 13 |
| 215 | ILF3_HUMAN | 95 kDa | 16 | 12 |
| 216 | NOL6_HUMAN | 128 kDa | 20 | 10 |
| 217 | PUR2_HUMAN | 108 kDa | 14 | 12 |
| 218 | SQSTM_HUMAN | 48 kDa | 15 | 14 |
| 219 | ACL6A_HUMAN | 47 kDa | 16 | 13 |
| 220 | ERP44_HUMAN | 47 kDa | 15 | 12 |
| 221 | K2C78_HUMAN | 57 kDa | 16 | 15 |
| 222 | RL7_HUMAN | 29 kDa | 14 | 10 |
| 223 | SMU1_HUMAN | 58 kDa | 16 | 13 |
| 224 | BCLF1_HUMAN | 106 kDa | 8 | 13 |
| 225 | REPI1_HUMAN | 64 kDa | 8 | 21 |
| 226 | PTBP1_HUMAN | 57 kDa | 22 | 7 |
| 227 | CUL4A_HUMAN | 88 kDa | 2 | 19 |
| 228 | AHNK_HUMAN | 629 kDa | 17 | 8 |
| 229 | CDK9_HUMAN | 43 kDa | 11 | 17 |
| 230 | CLK3_HUMAN | 74 kDa | 14 | 15 |
| 231 | CV028_HUMAN | 55 kDa | 7 | 22 |
| 232 | MYH10_HUMAN | 229 kDa | 21 | 7 |
| 233 | WDR33_HUMAN | 146 kDa | 4 | 10 |
| 234 | COR1A_HUMAN | 51 kDa | 17 | 12 |
| 235 | CPSF5_HUMAN | 26 kDa | 7 | 19 |
| 236 | IQGA1_HUMAN | 189 kDa | 16 | 11 |
| 237 | RS16_HUMAN | 16 kDa | 11 | 17 |
| 238 | UBA1_HUMAN | 118 kDa | 21 | 6 |
| 239 | UBAP2_HUMAN | 117 kDa | 12 | 19 |
| 240 | PSME2_HUMAN | 27 kDa | 16 | 12 |
| 241 | CT027_HUMAN | 19 kDa | 8 | 21 |
| 242 | CDK12_HUMAN | 164 kDa | 8 | 17 |
| 243 | DHX9_HUMAN | 141 kDa | 13 | 14 |
| 244 | FXR1_HUMAN | 70 kDa | 8 | 16 |
| 245 | HNRPC_HUMAN | 34 kDa | 20 | 7 |
| 246 | HSP71_HUMAN | 70 kDa | 7 | 20 |
| 247 | HYOU1_HUMAN | 111 kDa | 19 | 7 |
| 248 | LANC2_HUMAN | 51 kDa | 11 | 14 |
| 249 | RS11_HUMAN | 18 kDa | 6 | 13 |
| 250 | SERA_HUMAN | 57 kDa | 18 | 13 |
| 251 | TAF4_HUMAN | 110 kDa | 14 | 16 |
| 252 | PDCD4_HUMAN | 52 kDa | 20 | 5 |
| 253 | ATPA_HUMAN | 60 kDa | 16 | 14 |
| 254 | DCAF7_HUMAN | 39 kDa | 9 | 14 |
| 255 | DHX30_HUMAN | 134 kDa | 8 | 17 |
| 256 | H2AY_HUMAN | 40 kDa | 9 | 15 |
| 257 | HNRPU_HUMAN | 91 kDa | 15 | 9 |
| 258 | IKZF3_HUMAN | 58 kDa | 10 | 16 |
| 259 | MDHM_HUMAN | 36 kDa | 17 | 13 |
| 260 | PYRG1_HUMAN | 67 kDa | 9 | 18 |
| 261 | PRDX4_HUMAN | 31 kDa | 15 | 5 |
| 262 | DCP1A_HUMAN | 63 kDa | 10 | 14 |
| 263 | DHE3_HUMAN | 61 kDa | 14 | 10 |
| 264 | GSTP1_HUMAN | 23 kDa | 10 | 13 |
| 265 | MCM3_HUMAN | 91 kDa | 12 | 13 |
| 266 | NPM_HUMAN | 33 kDa | 13 | 7 |
| 267 | RS18_HUMAN | 18 kDa | 13 | 14 |
| 268 | SPF45_HUMAN | 45 kDa | 8 | 18 |
| 269 | TXND5_HUMAN | 48 kDa | 13 | 10 |
| 270 | TYDP1_HUMAN | 68 kDa | 8 | 16 |
| 271 | WDR24_HUMAN | 102 kDa | 10 | 14 |
| 272 | YLPM1_HUMAN | 220 kDa | 8 | 13 |
| 273 | ZC3HD_HUMAN | 197 kDa | 5 | 15 |
| 274 | RS27_HUMAN | 9 kDa | 6 | 14 |
| 275 | ZCCHL_HUMAN | 33 kDa | 10 | 12 |
| 276 | LONM_HUMAN | 106 kDa | 18 | 3 |
| 277 | CN166_HUMAN | 28 kDa | 9 | 18 |
| 278 | GPTC8_HUMAN | 164 kDa | 7 | 14 |
| 279 | K6PF_HUMAN | 85 kDa | 7 | 21 |
| 280 | MCM2_HUMAN | 102 kDa | 10 | 14 |
| 281 | PSMD1_HUMAN | 106 kDa | 19 | 5 |
| 282 | SMRC1_HUMAN | 123 kDa | 6 | 11 |
| 283 | XPO2_HUMAN | 110 kDa | 11 | 12 |
| 284 | NUMA1_HUMAN | 238 kDa | 17 | 6 |
| 285 | PABP4_HUMAN | 71 kDa | 10 | 9 |
| 286 | ATAD2_HUMAN | 159 kDa | 2 | 18 |
| 287 | BAZ1B_HUMAN | 171 kDa | 6 | 12 |
| 288 | C1TC_HUMAN | 102 kDa | 9 | 15 |
| 289 | DYHC1_HUMAN | 532 kDa | 16 | 6 |
| 290 | EF2_HUMAN | 95 kDa | 6 | 12 |
| 291 | GOGA3_HUMAN | 167 kDa | 5 | 17 |
| 292 | K2C4_HUMAN | 57 kDa | 13 | 9 |
| 293 | NCKPL_HUMAN | 128 kDa | 11 | 10 |
| 294 | SND1_HUMAN | 102 kDa | 18 | 9 |
| 295 | TAF9_HUMAN | 29 kDa | 11 | 14 |
| 296 | TLE3_HUMAN | 83 kDa | 7 | 15 |
| 297 | SAHH_HUMAN | 48 kDa | 15 | 5 |
| 298 | NUCL_HUMAN | 77 kDa | 18 | 4 |
| 299 | TRA2A_HUMAN | 33 kDa | 5 | 15 |
| 300 | IKZF1_HUMAN | 58 kDa | 12 | 11 |
| 301 | RL19_HUMAN | 23 kDa | 1 | 18 |
| 302 | TBA4A_HUMAN | 50 kDa | 11 | 15 |
| 303 | RS23_HUMAN | 16 kDa | 9 | 15 |
| 304 | DOK6_HUMAN | 38 kDa | 2 | 1 |
| 305 | COPA_HUMAN | 138 kDa | 12 | 10 |
| 306 | GCP2_HUMAN | 103 kDa | 11 | 11 |
| 307 | KINH_HUMAN | 110 kDa | 7 | 13 |
| 308 | PRP6_HUMAN | 107 kDa | 12 | 12 |
| 309 | SMRD2_HUMAN | 52 kDa | 11 | 10 |
| 310 | SYIC_HUMAN | 145 kDa | 14 | 8 |
| 311 | ZN574_HUMAN | 99 kDa | 7 | 14 |
| 312 | ZN768_HUMAN | 60 kDa | 6 | 18 |
| 313 | RL13A_HUMAN | 24 kDa | 4 | 15 |
| 314 | SMAD4_HUMAN | 60 kDa | 4 | 10 |
| 315 | RING1_HUMAN | 42 kDa | 8 | 13 |
| 316 | PIP_HUMAN | 17 kDa | 13 | 12 |
| 317 | RS26_HUMAN | 13 kDa | 3 | 19 |
| 318 | CO1A1_HUMAN | 139 kDa | 14 | 11 |
| 319 | IF2A_HUMAN | 36 kDa | 15 | 6 |
| 320 | NFKB2_HUMAN | 97 kDa | 7 | 11 |
| 321 | OGT1_HUMAN | 117 kDa | 8 | 12 |
| 322 | PSMD2_HUMAN | 100 kDa | 9 | 10 |
| 323 | RSMB_HUMAN | 25 kDa | 8 | 13 |
| 324 | SNUT1_HUMAN | 90 kDa | 7 | 13 |
| 325 | USO1_HUMAN | 108 kDa | 11 | 10 |
| 326 | SKT_HUMAN | 214 kDa | 15 | 3 |
| 327 | DYR1A_HUMAN | 86 kDa | 15 | 4 |
| 328 | OSBL9_HUMAN | 83 kDa | 13 | 6 |
| 329 | ANXA6_HUMAN | 76 kDa | 19 | 5 |
| 330 | IN80E_HUMAN | 26 kDa | 12 | 9 |
| 331 | 2AAA_HUMAN | 65 kDa | 8 | 12 |
| 332 | ARI1A_HUMAN | 242 kDa | 9 | 11 |
| 333 | ATPB_HUMAN | 57 kDa | 13 | 11 |
| 334 | CBX4_HUMAN | 61 kDa | 10 | 10 |
| 335 | DOCK6_HUMAN | 230 kDa | 5 | 16 |
| 336 | RASL3_HUMAN | 112 kDa | 7 | 13 |
| 337 | RS14_HUMAN | 16 kDa | 11 | 11 |
| 338 | SEH1_HUMAN | 40 kDa | 10 | 12 |
| 339 | SYDC_HUMAN | 57 kDa | 11 | 13 |
| 340 | TPP2_HUMAN | 138 kDa | 12 | 7 |
| 341 | VPS11_HUMAN | 108 kDa | 9 | 8 |
| 342 | ZN281_HUMAN | 97 kDa | 7 | 12 |
| 343 | COPB_HUMAN | 107 kDa | 5 | 18 |
| 344 | 1433T_HUMAN | 28 kDa | 8 | 9 |
| 345 | SYLC_HUMAN | 134 kDa | 16 | 4 |
| 346 | TBB2B_HUMAN | 50 kDa | 5 | 20 |
| 347 | RCC2_HUMAN | 56 kDa | 3 | 14 |
| 348 | ANR44_HUMAN | 108 kDa | 9 | 10 |
| 349 | COPB2_HUMAN | 102 kDa | 15 | 6 |
| 350 | DDX3X_HUMAN | 73 kDa | 6 | 15 |
| 351 | DRG1_HUMAN | 41 kDa | 11 | 11 |
| 352 | EPIPL_HUMAN | 553 kDa | 5 | 16 |
| 353 | GRP75_HUMAN | 74 kDa | 11 | 9 |
| 354 | ILF2_HUMAN | 43 kDa | 12 | 10 |
| 355 | PABP1_HUMAN | 71 kDa | 13 | 7 |
| 356 | SF17A_HUMAN | 81 kDa | 4 | 11 |
| 357 | SYEP_HUMAN | 171 kDa | 13 | 5 |
| 358 | SYVC_HUMAN | 140 kDa | 14 | 6 |
| 359 | VP13C_HUMAN | 422 kDa | 10 | 10 |
| 360 | GANAB_HUMAN | 107 kDa | 10 | 5 |
| 361 | RS15A_HUMAN | 15 kDa | 4 | 13 |
| 362 | NU188_HUMAN | 196 kDa | 5 | 16 |
| 363 | 1433G_HUMAN | 28 kDa | 15 | 6 |
| 364 | PDIA6_HUMAN | 48 kDa | 15 | 5 |
| 365 | PBX1_HUMAN | 47 kDa | 5 | 15 |
| 366 | HNRPR_HUMAN | 71 kDa | 19 | 3 |
| 367 | ARMC8_HUMAN | 76 kDa | 12 | 9 |
| 368 | CLK1_HUMAN | 57 kDa | 4 | 5 |
| 369 | DDX6_HUMAN | 54 kDa | 7 | 12 |
| 370 | ELMO1_HUMAN | 84 kDa | 8 | 10 |
| 371 | RLA0_HUMAN | 34 kDa | 8 | 9 |
| 372 | SET1B_HUMAN | 209 kDa | 6 | 13 |
| 373 | SYWC_HUMAN | 53 kDa | 14 | 5 |
| 374 | DSC1_HUMAN | 100 kDa | 12 | 6 |
| 375 | NOP58_HUMAN | 60 kDa | 3 | 17 |
| 376 | SF3B4_HUMAN | 44 kDa | 7 | 15 |
| 377 | H2A1A_HUMAN (+1) | 14 kDa | 6 | 13 |
| 378 | ZN250_HUMAN | 63 kDa | 1 | 19 |
| 379 | CPSF7_HUMAN | 52 kDa | 11 | 10 |
| 380 | INO80_HUMAN | 177 kDa | 13 | 7 |
| 381 | RL7A_HUMAN | 30 kDa | 12 | 7 |
| 382 | RRP5_HUMAN | 209 kDa | 6 | 11 |
| 383 | SYRC_HUMAN | 75 kDa | 10 | 8 |
| 384 | HELC1_HUMAN | 251 kDa | 5 | 15 |
| 385 | CAP1_HUMAN | 52 kDa | 13 | 4 |
| 386 | K1C13_HUMAN | 50 kDa | 15 | 5 |
| 387 | HBA_HUMAN | 15 kDa | 10 | 8 |
| 388 | FILA2_HUMAN | 248 kDa | 9 | 8 |
| 389 | CPEB4_HUMAN | 80 kDa | 1 | 13 |
| 390 | RAE1L_HUMAN | 41 kDa | 14 | 4 |
| 391 | RL29_HUMAN | 18 kDa | 9 | 11 |
| 392 | ATX2_HUMAN | 140 kDa | 1 | 14 |
| 393 | F120C_HUMAN | 121 kDa | 1 | 17 |
| 394 | COBL_HUMAN | 136 kDa | 11 | 7 |
| 395 | DDX17_HUMAN | 72 kDa | 8 | 10 |
| 396 | KDM1_HUMAN | 93 kDa | 9 | 6 |
| 397 | NUP98_HUMAN | 188 kDa | 8 | 8 |
| 398 | VP33A_HUMAN | 68 kDa | 7 | 5 |
| 399 | ZCHC8_HUMAN | 79 kDa | 6 | 12 |
| 400 | RU2B_HUMAN | 25 kDa | 8 | 9 |
| 401 | KAP0_HUMAN | 43 kDa | 13 | 3 |
| 402 | RNPS1_HUMAN | 34 kDa | 6 | 10 |
| 403 | XRN1_HUMAN | 194 kDa | 2 | 15 |
| 404 | CPSF3_HUMAN | 77 kDa | 7 | 8 |
| 405 | DDX21_HUMAN | 87 kDa | 8 | 11 |
| 406 | IMDH2_HUMAN | 56 kDa | 8 | 10 |
| 407 | NU155_HUMAN | 155 kDa | 8 | 11 |
| 408 | RL23A_HUMAN | 18 kDa | 7 | 6 |
| 409 | SAPS1_HUMAN | 97 kDa | 8 | 10 |
| 410 | SARM1_HUMAN | 79 kDa | 10 | 8 |
| 411 | SC16A_HUMAN | 234 kDa | 10 | 5 |
| 412 | SMCE1_HUMAN | 47 kDa | 7 | 8 |
| 413 | TAF1_HUMAN | 213 kDa | 7 | 8 |
| 414 | VPS8_HUMAN | 162 kDa | 4 | 6 |
| 415 | ZBT38_HUMAN | 134 kDa | 11 | 5 |
| 416 | RHG04_HUMAN | 105 kDa | 8 | 8 |
| 417 | BRE1B_HUMAN | 114 kDa | 4 | 10 |
| 418 | TRI27_HUMAN | 58 kDa | 8 | 11 |
| 419 | EIF3E_HUMAN | 52 kDa | 12 | 3 |
| 420 | PSMD3_HUMAN | 61 kDa | 12 | 3 |
| 421 | RL23_HUMAN | 15 kDa | 7 | 10 |
| 422 | CC063_HUMAN | 189 kDa | 2 | 12 |
| 423 | DCD_HUMAN | 11 kDa | 8 | 9 |
| 424 | ARHG7_HUMAN | 90 kDa | 6 | 11 |
| 425 | BIRC6_HUMAN | 528 kDa | 11 | 6 |
| 426 | CCNK_HUMAN | 64 kDa | 7 | 11 |
| 427 | DDX1_HUMAN | 82 kDa | 8 | 9 |
| 428 | F120A_HUMAN | 122 kDa | 10 | 5 |
| 429 | GCP3_HUMAN | 104 kDa | 7 | 10 |
| 430 | NCOR2_HUMAN | 275 kDa | 6 | 6 |
| 431 | PB1_HUMAN | 193 kDa | 9 | 7 |
| 432 | PHF3_HUMAN | 229 kDa | 7 | 11 |
| 433 | K1967_HUMAN | 103 kDa | 4 | 10 |
| 434 | NDKB_HUMAN | 17 kDa | 6 | 8 |
| 435 | UBP2L_HUMAN | 115 kDa | 7 | 8 |
| 436 | RL8_HUMAN | 28 kDa | 10 | 8 |
| 437 | PDC6I_HUMAN | 96 kDa | 11 | 4 |
| 438 | RBBP7_HUMAN | 48 kDa | 4 | 10 |
| 439 | MDN1_HUMAN | 633 kDa | 13 | 4 |
| 440 | PDIA1_HUMAN | 57 kDa | 11 | 3 |
| 441 | PRS4_HUMAN | 49 kDa | 4 | 12 |
| 442 | DOCK7_HUMAN | 243 kDa | 2 | 11 |
| 443 | GLYR1_HUMAN | 61 kDa | 7 | 10 |
| 444 | YBOX1_HUMAN | 36 kDa | 12 | 3 |
| 445 | RL14_HUMAN | 23 kDa | 7 | 10 |
| 446 | MY18A_HUMAN | 233 kDa | 8 | 5 |
| 447 | PSME3_HUMAN | 30 kDa | 6 | 9 |
| 448 | RS13_HUMAN | 17 kDa | 5 | 9 |
| 449 | TBL1R_HUMAN | 56 kDa | 7 | 10 |
| 450 | TFP11_HUMAN | 97 kDa | 5 | 8 |
| 451 | P5CR2_HUMAN | 34 kDa | 10 | 4 |
| 452 | ZN646_HUMAN | 201 kDa | 6 | 10 |
| 453 | PAF1_HUMAN | 60 kDa | 7 | 8 |
| 454 | PPIB_HUMAN | 24 kDa | 8 | 6 |
| 455 | RL1D1_HUMAN | 55 kDa | 5 | 6 |
| 456 | CATA_HUMAN | 60 kDa | 9 | 7 |
| 457 | EIF3A_HUMAN | 167 kDa | 8 | 6 |
| 458 | SYQ_HUMAN | 88 kDa | 10 | 5 |
| 459 | POP1_HUMAN | 115 kDa | 6 | 8 |
| 460 | PDS5B_HUMAN | 165 kDa | 3 | 7 |
| 461 | ELAV1_HUMAN | 36 kDa | 3 | 10 |
| 462 | RRP7A_HUMAN | 32 kDa | 9 | 7 |
| 463 | FUS_HUMAN | 53 kDa | 9 | 4 |
| 464 | ADT3_HUMAN | 33 kDa | 6 | 6 |
| 465 | ROA3_HUMAN | 40 kDa | 11 | 4 |
| 466 | IF4G1_HUMAN | 176 kDa | 9 | 3 |
| 467 | RBM7_HUMAN | 31 kDa | 3 | 11 |
| 468 | CARM1_HUMAN | 63 kDa | 1 | 12 |
| 469 | RL24_HUMAN | 18 kDa | 5 | 9 |
| 470 | PRDX2_HUMAN | 22 kDa | 8 | 1 |
| 471 | BTBDB_HUMAN | 121 kDa | 0 | 15 |
| 472 | AAKG1_HUMAN | 38 kDa | 8 | 6 |
| 473 | EIF3L_HUMAN | 67 kDa | 8 | 6 |
| 474 | PANK4_HUMAN | 86 kDa | 8 | 4 |
| 475 | SC23B_HUMAN | 86 kDa | 8 | 6 |
| 476 | UGPA_HUMAN | 57 kDa | 8 | 7 |
| 477 | NUP93_HUMAN | 93 kDa | 5 | 8 |
| 478 | UBE2N_HUMAN | 17 kDa | 6 | 7 |
| 479 | RBM39_HUMAN | 59 kDa | 7 | 8 |
| 480 | UBQL1_HUMAN | 63 kDa | 8 | 5 |
| 481 | 1433Z_HUMAN | 28 kDa | 9 | 8 |
| 482 | OZF_HUMAN | 33 kDa | 6 | 7 |
| 483 | PPIA_HUMAN | 18 kDa | 6 | 10 |
| 484 | RL27_HUMAN | 16 kDa | 7 | 5 |
| 485 | EDC3_HUMAN | 56 kDa | 4 | 5 |
| 486 | SF3A2_HUMAN | 49 kDa | 3 | 5 |
| 487 | MCM6_HUMAN | 93 kDa | 9 | 6 |
| 488 | RL6_HUMAN | 33 kDa | 9 | 4 |
| 489 | ZNF34_HUMAN | 62 kDa | 4 | 13 |
| 490 | PM14_HUMAN | 15 kDa | 4 | 11 |
| 491 | TF2AA_HUMAN | 42 kDa | 4 | 11 |
| 492 | HAKAI_HUMAN | 55 kDa | 2 | 14 |
| 493 | SP16H_HUMAN | 120 kDa | 11 | 1 |
| 494 | MAZ_HUMAN | 49 kDa | 2 | 8 |
| 495 | PSD11_HUMAN | 47 kDa | 15 | 0 |
| 496 | VPS18_HUMAN | 110 kDa | 6 | 6 |
| 497 | DOCK2_HUMAN | 212 kDa | 7 | 5 |
| 498 | K0802_HUMAN | 209 kDa | 5 | 8 |
| 499 | DAPLE_HUMAN | 228 kDa | 10 | 6 |
| 500 | NRBP_HUMAN | 60 kDa | 4 | 6 |
| 501 | ASML_HUMAN | 69 kDa | 5 | 10 |
| 502 | RNZ2_HUMAN | 92 kDa | 7 | 5 |
| 503 | KU86_HUMAN | 83 kDa | 9 | 4 |
| 504 | LIPA1_HUMAN | 136 kDa | 7 | 5 |
| 505 | BAP31_HUMAN | 28 kDa | 8 | 5 |
| 506 | CT011_HUMAN | 27 kDa | 7 | 9 |
| 507 | DSRAD_HUMAN | 136 kDa | 6 | 7 |
| 508 | EIF3C_HUMAN | 105 kDa | 10 | 4 |
| 509 | PKP2_HUMAN | 97 kDa | 5 | 8 |
| 510 | TRRAP_HUMAN | 438 kDa | 10 | 4 |
| 511 | MK01_HUMAN | 41 kDa | 7 | 5 |
| 512 | RS20_HUMAN | 13 kDa | 4 | 7 |
| 513 | PDIA3_HUMAN | 57 kDa | 8 | 4 |
| 514 | NAMPT_HUMAN | 56 kDa | 2 | 10 |
| 515 | NOG1_HUMAN | 74 kDa | 4 | 10 |
| 516 | ARP5_HUMAN | 68 kDa | 8 | 6 |
| 517 | NKRF_HUMAN | 78 kDa | 2 | 11 |
| 518 | DNJB6_HUMAN | 36 kDa | 7 | 4 |
| 519 | FUBP2_HUMAN | 73 kDa | 9 | 3 |
| 520 | PBIP1_HUMAN | 81 kDa | 3 | 10 |
| 521 | PRP19_HUMAN | 55 kDa | 3 | 7 |
| 522 | FA98A_HUMAN | 55 kDa | 3 | 10 |
| 523 | TNPO3_HUMAN | 104 kDa | 1 | 7 |
| 524 | 1433B_HUMAN | 28 kDa | 8 | 7 |
| 525 | ZFYV1_HUMAN | 87 kDa | 9 | 3 |
| 526 | FRAP_HUMAN | 289 kDa | 5 | 7 |
| 527 | MBB1A_HUMAN | 149 kDa | 5 | 5 |
| 528 | HUWE1_HUMAN | 482 kDa | 4 | 5 |
| 529 | MYL6_HUMAN | 17 kDa | 7 | 6 |
| 530 | RALY_HUMAN | 32 kDa | 7 | 4 |
| 531 | RRP44_HUMAN | 109 kDa | 7 | 5 |
| 532 | WDR5_HUMAN | 37 kDa | 8 | 7 |
| 533 | HTR7A_HUMAN | 181 kDa | 7 | 5 |
| 534 | MLL5_HUMAN | 205 kDa | 4 | 8 |
| 535 | PP2AA_HUMAN | 36 kDa | 5 | 6 |
| 536 | HMGB1_HUMAN | 25 kDa | 4 | 5 |
| 537 | GIT1_HUMAN | 84 kDa | 4 | 6 |
| 538 | HCFC1_HUMAN | 209 kDa | 8 | 5 |
| 539 | ZN771_HUMAN | 36 kDa | 5 | 6 |
| 540 | RB12B_HUMAN | 118 kDa | 3 | 6 |
| 541 | RS24_HUMAN | 15 kDa | 5 | 8 |
| 542 | LSG1_HUMAN | 75 kDa | 2 | 9 |
| 543 | PRS6A_HUMAN | 49 kDa | 8 | 3 |
| 544 | CTBP1_HUMAN | 48 kDa | 3 | 11 |
| 545 | ERG7_HUMAN | 83 kDa | 4 | 9 |
| 546 | RAB1B_HUMAN | 22 kDa | 9 | 5 |
| 547 | RL18_HUMAN | 22 kDa | 5 | 9 |
| 548 | ABHEB_HUMAN | 22 kDa | 5 | 5 |
| 549 | RL10A_HUMAN | 25 kDa | 9 | 3 |
| 550 | RSBN1_HUMAN | 90 kDa | 2 | 9 |
| 551 | SNAA_HUMAN | 33 kDa | 8 | 3 |
| 552 | CYTA_HUMAN | 11 kDa | 8 | 5 |
| 553 | NUDC1_HUMAN | 67 kDa | 2 | 11 |
| 554 | RHG01_HUMAN | 50 kDa | 1 | 10 |
| 555 | CCD55_HUMAN | 66 kDa | 0 | 11 |
| 556 | DHAK_HUMAN | 59 kDa | 13 | 0 |
| 557 | BACH_HUMAN | 42 kDa | 7 | 7 |
| 558 | STRUM_HUMAN | 134 kDa | 6 | 6 |
| 559 | SYFB_HUMAN | 66 kDa | 6 | 7 |
| 560 | CNDD3_HUMAN | 169 kDa | 6 | 5 |
| 561 | NIPBL_HUMAN | 316 kDa | 4 | 3 |
| 562 | COPE_HUMAN | 34 kDa | 5 | 5 |
| 563 | MCRS1_HUMAN | 52 kDa | 6 | 5 |
| 564 | PHB2_HUMAN | 33 kDa | 8 | 5 |
| 565 | PUR8_HUMAN | 55 kDa | 5 | 5 |
| 566 | PRS7_HUMAN | 49 kDa | 6 | 8 |
| 567 | MCM5_HUMAN | 82 kDa | 4 | 5 |
| 568 | COPG_HUMAN | 98 kDa | 6 | 6 |
| 569 | DNPEP_HUMAN | 52 kDa | 6 | 5 |
| 570 | RTTN_HUMAN | 249 kDa | 4 | 7 |
| 571 | 2ABA_HUMAN | 52 kDa | 5 | 5 |
| 572 | WDR6_HUMAN | 122 kDa | 4 | 5 |
| 573 | SF3A3_HUMAN | 59 kDa | 4 | 8 |
| 574 | DDX5_HUMAN | 69 kDa | 4 | 7 |
| 575 | KLC1_HUMAN | 65 kDa | 6 | 4 |
| 576 | ROA2_HUMAN | 37 kDa | 4 | 6 |
| 577 | DDX31_HUMAN | 94 kDa | 4 | 9 |
| 578 | RS10_HUMAN | 19 kDa | 6 | 5 |
| 579 | SMD2_HUMAN | 14 kDa | 7 | 6 |
| 580 | TNPO1_HUMAN | 102 kDa | 4 | 6 |
| 581 | HDAC1_HUMAN | 55 kDa | 5 | 8 |
| 582 | API5_HUMAN | 58 kDa | 9 | 2 |
| 583 | DDX18_HUMAN | 75 kDa | 3 | 11 |
| 584 | PDXK_HUMAN | 35 kDa | 9 | 3 |
| 585 | TRAP1_HUMAN | 80 kDa | 5 | 7 |
| 586 | WDR62_HUMAN | 166 kDa | 7 | 5 |
| 587 | SSBP_HUMAN | 17 kDa | 6 | 6 |
| 588 | TFPT_HUMAN | 28 kDa | 6 | 4 |
| 589 | LRCH3_HUMAN | 86 kDa | 5 | 4 |
| 590 | PPHLN_HUMAN | 53 kDa | 3 | 7 |
| 591 | PSD13_HUMAN | 43 kDa | 8 | 2 |
| 592 | HORN_HUMAN | 282 kDa | 4 | 5 |
| 593 | EF1D_HUMAN | 31 kDa | 7 | 3 |
| 594 | RS29_HUMAN | 7 kDa | 3 | 10 |
| 595 | PKP1_HUMAN | 83 kDa | 2 | 8 |
| 596 | FEN1_HUMAN | 43 kDa | 8 | 3 |
| 597 | ORC5_HUMAN | 50 kDa | 2 | 10 |
| 598 | PSMD6_HUMAN | 46 kDa | 10 | 2 |
| 599 | RRP12_HUMAN | 144 kDa | 3 | 6 |
| 600 | 1B15_HUMAN (+2) | 40 kDa | 7 | 7 |
| 601 | WDR59_HUMAN | 110 kDa | 10 | 1 |
| 602 | CD2A2_HUMAN | 18 kDa | 3 | 8 |
| 603 | MAP7_HUMAN | 84 kDa | 3 | 6 |
| 604 | PUR4_HUMAN | 145 kDa | 13 | 0 |
| 605 | CND1_HUMAN | 157 kDa | 6 | 6 |
| 606 | EHD1_HUMAN | 61 kDa | 7 | 6 |
| 607 | MCM7_HUMAN | 81 kDa | 6 | 4 |
| 608 | TBG1_HUMAN | 51 kDa | 4 | 5 |
| 609 | CUL1_HUMAN | 90 kDa | 6 | 4 |
| 610 | ZN638_HUMAN | 221 kDa | 4 | 7 |
| 611 | TAF8_HUMAN | 34 kDa | 6 | 5 |
| 612 | SNF5_HUMAN | 44 kDa | 4 | 6 |
| 613 | MMS19_HUMAN | 113 kDa | 4 | 5 |
| 614 | DC1I2_HUMAN | 71 kDa | 7 | 5 |
| 615 | VPS16_HUMAN | 95 kDa | 4 | 3 |
| 616 | WAHS7_HUMAN | 136 kDa | 4 | 7 |
| 617 | BRE1A_HUMAN | 114 kDa | 6 | 4 |
| 618 | GCFC_HUMAN | 105 kDa | 4 | 9 |
| 619 | MYO1G_HUMAN | 116 kDa | 4 | 9 |
| 620 | RED_HUMAN | 66 kDa | 5 | 5 |
| 621 | FBSH_HUMAN | 39 kDa | 6 | 6 |
| 622 | RANB9_HUMAN | 78 kDa | 7 | 5 |
| 623 | CSTF2_HUMAN | 61 kDa | 3 | 6 |
| 624 | ZN696_HUMAN | 41 kDa | 4 | 6 |
| 625 | FA49B_HUMAN | 37 kDa | 6 | 4 |
| 626 | LARP1_HUMAN | 124 kDa | 2 | 3 |
| 627 | BUB3_HUMAN | 37 kDa | 5 | 3 |
| 628 | IMA3_HUMAN | 58 kDa | 3 | 8 |
| 629 | RRP1B_HUMAN | 84 kDa | 2 | 4 |
| 630 | VCIP1_HUMAN | 134 kDa | 3 | 7 |
| 631 | CAPZB_HUMAN | 31 kDa | 4 | 8 |
| 632 | KCD10_HUMAN | 35 kDa | 4 | 6 |
| 633 | COPD_HUMAN | 57 kDa | 6 | 5 |
| 634 | PHC3_HUMAN | 106 kDa | 7 | 5 |
| 635 | KLC4_HUMAN | 69 kDa | 2 | 7 |
| 636 | MACF1_HUMAN | 620 kDa | 2 | 6 |
| 637 | RAC2_HUMAN | 21 kDa | 4 | 4 |
| 638 | IF4A3_HUMAN | 47 kDa | 9 | 3 |
| 639 | TYK2_HUMAN | 134 kDa | 5 | 4 |
| 640 | DEPD6_HUMAN | 46 kDa | 8 | 3 |
| 641 | PGAM1_HUMAN | 29 kDa | 4 | 3 |
| 642 | GNL3_HUMAN | 62 kDa | 2 | 9 |
| 643 | PRS10_HUMAN | 44 kDa | 6 | 5 |
| 644 | IDHP_HUMAN | 51 kDa | 9 | 2 |
| 645 | VDAC1_HUMAN | 31 kDa | 8 | 3 |
| 646 | G3BP1_HUMAN | 52 kDa | 10 | 2 |
| 647 | SIK3_HUMAN | 140 kDa | 2 | 10 |
| 648 | S10A9_HUMAN | 13 kDa | 3 | 8 |
| 649 | STIP1_HUMAN | 63 kDa | 7 | 3 |
| 650 | ZN384_HUMAN | 63 kDa | 1 | 7 |
| 651 | OPA1_HUMAN | 112 kDa | 1 | 7 |
| 652 | TGM3_HUMAN | 77 kDa | 4 | 1 |
| 653 | ANR50_HUMAN | 156 kDa | 1 | 7 |
| 654 | FOXJ3_HUMAN | 69 kDa | 2 | 10 |
| 655 | H15_HUMAN | 23 kDa | 12 | 1 |
| 656 | FAM5C_HUMAN-R | ? | 0 | 2 |
| 657 | SNIP1_HUMAN | 46 kDa | 0 | 11 |
| 658 | A16A1_HUMAN | 85 kDa | 6 | 5 |
| 659 | PSD12_HUMAN | 53 kDa | 7 | 5 |
| 660 | SMCA4_HUMAN | 185 kDa | 5 | 5 |
| 661 | PK3C3_HUMAN | 102 kDa | 3 | 4 |
| 662 | MED23_HUMAN | 156 kDa | 4 | 3 |
| 663 | STRAP_HUMAN | 38 kDa | 6 | 5 |
| 664 | ELP3_HUMAN | 62 kDa | 4 | 5 |
| 665 | SNR40_HUMAN | 39 kDa | 4 | 4 |
| 666 | RPN1_HUMAN | 69 kDa | 5 | 5 |
| 667 | UBR5_HUMAN | 309 kDa | 6 | 4 |
| 668 | CALR_HUMAN | 48 kDa | 6 | 5 |
| 669 | URP2_HUMAN | 76 kDa | 4 | 4 |
| 670 | LAP2A_HUMAN | 75 kDa | 6 | 3 |
| 671 | SYK_HUMAN | 68 kDa | 7 | 3 |
| 672 | CALM_HUMAN | 17 kDa | 6 | 4 |
| 673 | GCP4_HUMAN | 76 kDa | 4 | 6 |
| 674 | KIF11_HUMAN | 119 kDa | 5 | 3 |
| 675 | TRM2A_HUMAN | 69 kDa | 3 | 4 |
| 676 | SRPK1_HUMAN | 74 kDa | 4 | 5 |
| 677 | GDIR2_HUMAN | 23 kDa | 7 | 2 |
| 678 | ANR28_HUMAN | 117 kDa | 2 | 6 |
| 679 | CC85C_HUMAN | 45 kDa | 3 | 6 |
| 680 | PDS5A_HUMAN | 151 kDa | 1 | 5 |
| 681 | RLA2_HUMAN | 12 kDa | 3 | 7 |
| 682 | RAGP1_HUMAN | 64 kDa | 4 | 6 |
| 683 | RPP30_HUMAN | 29 kDa | 4 | 7 |
| 684 | PI42A_HUMAN | 46 kDa | 7 | 5 |
| 685 | RBM27_HUMAN | 119 kDa | 2 | 6 |
| 686 | K2C80_HUMAN | 51 kDa | 7 | 2 |
| 687 | FA50A_HUMAN | 40 kDa | 6 | 2 |
| 688 | MINA_HUMAN | 53 kDa | 3 | 4 |
| 689 | RPB2_HUMAN | 134 kDa | 8 | 1 |
| 690 | ROA1_HUMAN | 39 kDa | 7 | 3 |
| 691 | RSBNL_HUMAN | 94 kDa | 3 | 8 |
| 692 | ZN187_HUMAN | 55 kDa | 2 | 8 |
| 693 | ANM1_HUMAN | 42 kDa | 6 | 3 |
| 694 | FNBP2_HUMAN | 121 kDa | 7 | 1 |
| 695 | ML12A_HUMAN (+1) | 20 kDa | 10 | 1 |
| 696 | EIF3D_HUMAN | 64 kDa | 6 | 1 |
| 697 | IF2G_HUMAN | 51 kDa | 6 | 1 |
| 698 | 5NTC_HUMAN | 65 kDa | 1 | 9 |
| 699 | MSH6_HUMAN | 153 kDa | 1 | 11 |
| 700 | RL26_HUMAN | 17 kDa | 5 | 1 |
| 701 | SMHD1_HUMAN | 226 kDa | 9 | 1 |
| 702 | OLIG2_HUMAN | 32 kDa | 1 | 6 |
| 703 | DEOC_HUMAN | 35 kDa | 6 | 5 |
| 704 | ARF1_HUMAN (+1) | 21 kDa | 5 | 6 |
| 705 | RU2A_HUMAN | 28 kDa | 5 | 6 |
| 706 | TGFA1_HUMAN | 97 kDa | 4 | 6 |
| 707 | ACACA_HUMAN | 266 kDa | 3 | 3 |
| 708 | GLYM_HUMAN | 56 kDa | 4 | 6 |
| 709 | DNJA2_HUMAN | 46 kDa | 5 | 4 |
| 710 | RO60_HUMAN | 61 kDa | 5 | 5 |
| 711 | MALT1_HUMAN | 92 kDa | 3 | 6 |
| 712 | CDC73_HUMAN | 61 kDa | 4 | 4 |
| 713 | ACLY_HUMAN | 121 kDa | 5 | 3 |
| 714 | ARGI1_HUMAN | 35 kDa | 6 | 2 |
| 715 | CP135_HUMAN | 134 kDa | 3 | 5 |
| 716 | ESYT1_HUMAN | 123 kDa | 3 | 6 |
| 717 | HELLS_HUMAN | 97 kDa | 4 | 5 |
| 718 | KAD2_HUMAN | 26 kDa | 5 | 4 |
| 719 | CS029_HUMAN | 80 kDa | 5 | 3 |
| 720 | UBR4_HUMAN | 574 kDa | 1 | 5 |
| 721 | TBPL1_HUMAN | 21 kDa | 3 | 8 |
| 722 | RERE_HUMAN | 172 kDa | 3 | 8 |
| 723 | ABCF1_HUMAN | 96 kDa | 4 | 5 |
| 724 | PRDX5_HUMAN | 22 kDa | 5 | 5 |
| 725 | PGK1_HUMAN | 45 kDa | 4 | 4 |
| 726 | TAF9B_HUMAN | 28 kDa | 5 | 5 |
| 727 | PP1B_HUMAN | 37 kDa | 6 | 5 |
| 728 | RL36_HUMAN | 12 kDa | 4 | 4 |
| 729 | THIO_HUMAN | 12 kDa | 2 | 2 |
| 730 | RAN_HUMAN | 24 kDa | 5 | 5 |
| 731 | VPS41_HUMAN | 99 kDa | 5 | 2 |
| 732 | CBX3_HUMAN | 21 kDa | 4 | 5 |
| 733 | BMP2K_HUMAN | 129 kDa | 2 | 8 |
| 734 | PDLI5_HUMAN | 64 kDa | 3 | 7 |
| 735 | TTC1_HUMAN | 34 kDa | 5 | 5 |
| 736 | CSK21_HUMAN | 45 kDa | 3 | 4 |
| 737 | IGHA1_HUMAN | 38 kDa | 3 | 5 |
| 738 | ATM_HUMAN | 351 kDa | 6 | 2 |
| 739 | CPSF6_HUMAN | 59 kDa | 2 | 7 |
| 740 | DDX23_HUMAN | 96 kDa | 7 | 3 |
| 741 | MSH2_HUMAN | 105 kDa | 8 | 2 |
| 742 | SYMC_HUMAN | 101 kDa | 6 | 3 |
| 743 | KAPCB_HUMAN | 41 kDa | 7 | 2 |
| 744 | TAGL2_HUMAN | 22 kDa | 8 | 2 |
| 745 | EIF3M_HUMAN | 43 kDa | 7 | 2 |
| 746 | SPS2L_HUMAN | 62 kDa | 3 | 7 |
| 747 | XRN2_HUMAN | 109 kDa | 2 | 2 |
| 748 | ZN623_HUMAN | 61 kDa | 2 | 8 |
| 749 | ERP29_HUMAN | 29 kDa | 6 | 5 |
| 750 | SP130_HUMAN | 110 kDa | 4 | 5 |
| 751 | CIP2A_HUMAN | 102 kDa | 1 | 4 |
| 752 | RS17_HUMAN | 16 kDa | 1 | 8 |
| 753 | RL28_HUMAN | 16 kDa | 1 | 3 |
| 754 | DJC10_HUMAN | 91 kDa | 1 | 7 |
| 755 | PUF60_HUMAN | 60 kDa | 7 | 1 |
| 756 | K2C6C_HUMAN | 60 kDa | 4 | 5 |
| 757 | GOPC_HUMAN | 51 kDa | 1 | 9 |
| 758 | ARMC6_HUMAN | 54 kDa | 1 | 7 |
| 759 | BMS1_HUMAN | 146 kDa | 0 | 10 |
| 760 | PLIN3_HUMAN | 47 kDa | 11 | 0 |
| 761 | RRBP1_HUMAN | 152 kDa | 8 | 0 |
| 762 | NOC4L_HUMAN | 58 kDa | 0 | 2 |
| 763 | IPO4_HUMAN | 119 kDa | 5 | 5 |
| 764 | NDE1_HUMAN | 39 kDa | 4 | 6 |
| 765 | OTUD4_HUMAN | 124 kDa | 4 | 4 |
| 766 | SC31A_HUMAN | 133 kDa | 6 | 3 |
| 767 | MCM4_HUMAN | 97 kDa | 3 | 4 |
| 768 | SMC1A_HUMAN | 143 kDa | 5 | 3 |
| 769 | ARC1B_HUMAN | 41 kDa | 4 | 4 |
| 770 | P5CR1_HUMAN | 33 kDa | 5 | 4 |
| 771 | IQGA2_HUMAN | 181 kDa | 3 | 6 |
| 772 | PR285_HUMAN | 295 kDa | 5 | 2 |
| 773 | CUL2_HUMAN | 87 kDa | 2 | 5 |
| 774 | IF2B1_HUMAN | 63 kDa | 5 | 3 |
| 775 | CLK4_HUMAN | 57 kDa | 5 | 3 |
| 776 | IN80C_HUMAN | 21 kDa | 7 | 3 |
| 777 | ABCE1_HUMAN | 67 kDa | 5 | 4 |
| 778 | AURKB_HUMAN | 39 kDa | 4 | 5 |
| 779 | TOP2A_HUMAN | 174 kDa | 4 | 4 |
| 780 | DJB11_HUMAN | 41 kDa | 5 | 4 |
| 781 | MK03_HUMAN | 43 kDa | 4 | 5 |
| 782 | SCC4_HUMAN | 69 kDa | 2 | 6 |
| 783 | RPGF6_HUMAN | 179 kDa | 3 | 3 |
| 784 | SAR1A_HUMAN | 22 kDa | 2 | 4 |
| 785 | P5CS_HUMAN | 87 kDa | 2 | 4 |
| 786 | SRP68_HUMAN | 71 kDa | 7 | 2 |
| 787 | ACINU_HUMAN | 152 kDa | 5 | 2 |
| 788 | PPP6_HUMAN | 35 kDa | 2 | 6 |
| 789 | AIP_HUMAN | 38 kDa | 4 | 4 |
| 790 | PDC10_HUMAN | 25 kDa | 4 | 1 |
| 791 | RS7_HUMAN | 22 kDa | 2 | 5 |
| 792 | OSGEP_HUMAN | 36 kDa | 7 | 2 |
| 793 | BMI1_HUMAN | 37 kDa | 6 | 2 |
| 794 | RL31_HUMAN | 14 kDa | 7 | 2 |
| 795 | TRM1L_HUMAN | 82 kDa | 2 | 6 |
| 796 | CSK_HUMAN | 51 kDa | 5 | 3 |
| 797 | SDF2L_HUMAN | 24 kDa | 5 | 3 |
| 798 | SPB12_HUMAN | 46 kDa | 5 | 2 |
| 799 | CDC37_HUMAN | 44 kDa | 6 | 2 |
| 800 | CND2_HUMAN | 83 kDa | 6 | 2 |
| 801 | MTM1_HUMAN | 70 kDa | 2 | 7 |
| 802 | PUR6_HUMAN | 47 kDa | 4 | 3 |
| 803 | NHRF1_HUMAN | 39 kDa | 3 | 4 |
| 804 | ZN652_HUMAN | 70 kDa | 3 | 6 |
| 805 | MTA1_HUMAN | 81 kDa | 4 | 2 |
| 806 | OTUB1_HUMAN | 31 kDa | 5 | 4 |
| 807 | NFX1_HUMAN | 124 kDa | 4 | 5 |
| 808 | PRPS2_HUMAN | 35 kDa | 2 | 6 |
| 809 | H2B1B_HUMAN (+3) | 14 kDa | 4 | 5 |
| 810 | RBMX2_HUMAN | 37 kDa | 1 | 7 |
| 811 | LKAP_HUMAN | 193 kDa | 1 | 8 |
| 812 | PSD7_HUMAN | 37 kDa | 6 | 1 |
| 813 | ATN1_HUMAN | 125 kDa | 1 | 6 |
| 814 | RMD5A_HUMAN | 44 kDa | 6 | 1 |
| 815 | IF2P_HUMAN | 139 kDa | 4 | 1 |
| 816 | FMR1_HUMAN | 71 kDa | 1 | 4 |
| 817 | CASPE_HUMAN | 28 kDa | 7 | 1 |
| 818 | GMDS_HUMAN | 42 kDa | 6 | 2 |
| 819 | AP1B1_HUMAN | 105 kDa | 6 | 1 |
| 820 | RSRC2_HUMAN | 51 kDa | 3 | 6 |
| 821 | DNLI3_HUMAN | 113 kDa | 6 | 0 |
| 822 | SNX2_HUMAN | 58 kDa | 8 | 0 |
| 823 | ZNF8_HUMAN | 65 kDa | 0 | 6 |
| 824 | ZFX_HUMAN (+1) | 91 kDa | 0 | 8 |
| 825 | CUL3_HUMAN | 89 kDa | 3 | 4 |
| 826 | IPO7_HUMAN | 120 kDa | 3 | 3 |
| 827 | ASNS_HUMAN | 64 kDa | 4 | 5 |
| 828 | G6PD_HUMAN | 59 kDa | 4 | 3 |
| 829 | AAPK1_HUMAN | 64 kDa | 4 | 3 |
| 830 | RPR1A_HUMAN | 36 kDa | 4 | 3 |
| 831 | BLM_HUMAN | 159 kDa | 4 | 3 |
| 832 | ANM5_HUMAN | 73 kDa | 4 | 3 |
| 833 | PYR5_HUMAN | 52 kDa | 3 | 4 |
| 834 | UBP14_HUMAN | 56 kDa | 2 | 3 |
| 835 | SMRD1_HUMAN | 58 kDa | 3 | 6 |
| 836 | CALX_HUMAN | 68 kDa | 5 | 4 |
| 837 | BAX_HUMAN | 21 kDa | 5 | 3 |
| 838 | SRRM2_HUMAN | 300 kDa | 3 | 4 |
| 839 | ISG20_HUMAN | 20 kDa | 4 | 3 |
| 840 | ARF6_HUMAN | 20 kDa | 3 | 5 |
| 841 | MOES_HUMAN | 68 kDa | 3 | 2 |
| 842 | EED_HUMAN | 50 kDa | 4 | 2 |
| 843 | RUXE_HUMAN | 11 kDa | 2 | 5 |
| 844 | TAF7_HUMAN | 40 kDa | 3 | 3 |
| 845 | DHX33_HUMAN | 79 kDa | 4 | 3 |
| 846 | PCBP2_HUMAN | 39 kDa | 5 | 2 |
| 847 | INF2_HUMAN | 136 kDa | 2 | 6 |
| 848 | RAD50_HUMAN | 154 kDa | 5 | 2 |
| 849 | KS6A5_HUMAN | 90 kDa | 3 | 3 |
| 850 | SMD1_HUMAN | 13 kDa | 3 | 3 |
| 851 | AIMP1_HUMAN | 34 kDa | 5 | 2 |
| 852 | NU133_HUMAN | 129 kDa | 2 | 4 |
| 853 | SCAI_HUMAN | 70 kDa | 2 | 6 |
| 854 | SEPT2_HUMAN | 41 kDa | 7 | 2 |
| 855 | SFR14_HUMAN | 120 kDa | 4 | 2 |
| 856 | WDR91_HUMAN | 83 kDa | 5 | 3 |
| 857 | KIF23_HUMAN | 110 kDa | 6 | 3 |
| 858 | CENPC_HUMAN | 107 kDa | 4 | 2 |
| 859 | CTR9_HUMAN | 134 kDa | 3 | 3 |
| 860 | CDC5L_HUMAN | 92 kDa | 4 | 3 |
| 861 | ZNF24_HUMAN | 42 kDa | 4 | 4 |
| 862 | TPIS_HUMAN | 27 kDa | 6 | 3 |
| 863 | HCD2_HUMAN | 27 kDa | 3 | 4 |
| 864 | LSM12_HUMAN | 22 kDa | 3 | 5 |
| 865 | ZN189_HUMAN | 73 kDa | 3 | 5 |
| 866 | LC7L3_HUMAN | 51 kDa | 4 | 2 |
| 867 | ELOC_HUMAN | 12 kDa | 2 | 5 |
| 868 | DYN2_HUMAN | 98 kDa | 1 | 2 |
| 869 | RL15_HUMAN | 24 kDa | 0 | 6 |
| 870 | ASH2L_HUMAN | 69 kDa | 2 | 5 |
| 871 | STUB1_HUMAN | 35 kDa | 2 | 4 |
| 872 | KCC2G_HUMAN | 63 kDa | 1 | 4 |
| 873 | RFC3_HUMAN | 41 kDa | 4 | 2 |
| 874 | TIAR_HUMAN | 42 kDa | 7 | 1 |
| 875 | TNIP1_HUMAN | 72 kDa | 3 | 2 |
| 876 | NCRP1_HUMAN | 31 kDa | 4 | 4 |
| 877 | ZN267_HUMAN | 87 kDa | 2 | 4 |
| 878 | 4F2_HUMAN | 68 kDa | 3 | 4 |
| 879 | HCLS1_HUMAN | 54 kDa | 6 | 1 |
| 880 | HNRL2_HUMAN | 85 kDa | 4 | 2 |
| 881 | TBB4_HUMAN | 50 kDa | 1 | 6 |
| 882 | UBP5_HUMAN | 96 kDa | 6 | 2 |
| 883 | HBB_HUMAN | 16 kDa | 7 | 1 |
| 884 | HNRH1_HUMAN | 49 kDa | 1 | 6 |
| 885 | SMD3_HUMAN | 14 kDa | 1 | 4 |
| 886 | PRS8_HUMAN | 46 kDa | 7 | 0 |
| 887 | CUL4B_HUMAN | 102 kDa | 0 | 6 |
| 888 | VIGLN_HUMAN | 141 kDa | 5 | 0 |
| 889 | PROF1_HUMAN | 15 kDa | 4 | 4 |
| 890 | TBL3_HUMAN | 89 kDa | 3 | 3 |
| 891 | RBM15_HUMAN | 107 kDa | 4 | 3 |
| 892 | CXXC1_HUMAN | 76 kDa | 5 | 3 |
| 893 | MFAP1_HUMAN | 52 kDa | 5 | 3 |
| 894 | WDR61_HUMAN | 34 kDa | 3 | 5 |
| 895 | MED24_HUMAN | 110 kDa | 4 | 3 |
| 896 | EXOC4_HUMAN | 111 kDa | 4 | 3 |
| 897 | GDPD3_HUMAN | 37 kDa | 5 | 3 |
| 898 | IF2B2_HUMAN | 66 kDa | 4 | 3 |
| 899 | ERF1_HUMAN | 49 kDa | 3 | 4 |
| 900 | CAZA1_HUMAN | 33 kDa | 4 | 3 |
| 901 | NGDN_HUMAN | 36 kDa | 4 | 3 |
| 902 | METK2_HUMAN | 44 kDa | 3 | 3 |
| 903 | RBP10_HUMAN | 67 kDa | 4 | 3 |
| 904 | IF6_HUMAN | 27 kDa | 4 | 3 |
| 905 | AMPD2_HUMAN | 101 kDa | 2 | 2 |
| 906 | KPRB_HUMAN | 41 kDa | 3 | 2 |
| 907 | GBLP_HUMAN | 35 kDa | 4 | 4 |
| 908 | IRAK1_HUMAN | 77 kDa | 4 | 4 |
| 909 | K1267_HUMAN | 121 kDa | 2 | 2 |
| 910 | CAND1_HUMAN | 136 kDa | 3 | 2 |
| 911 | CYTB_HUMAN | 11 kDa | 3 | 2 |
| 912 | HPRT_HUMAN | 25 kDa | 2 | 4 |
| 913 | WDR18_HUMAN | 47 kDa | 2 | 3 |
| 914 | IMA4_HUMAN | 58 kDa | 3 | 4 |
| 915 | TPPC3_HUMAN | 20 kDa | 3 | 3 |
| 916 | PGRC2_HUMAN | 24 kDa | 4 | 3 |
| 917 | BID_HUMAN | 22 kDa | 2 | 3 |
| 918 | SEC13_HUMAN | 36 kDa | 3 | 2 |
| 919 | CORO7_HUMAN | 101 kDa | 2 | 4 |
| 920 | KPBB_HUMAN | 125 kDa | 3 | 3 |
| 921 | PI3R4_HUMAN | 153 kDa | 2 | 4 |
| 922 | ACTZ_HUMAN | 43 kDa | 4 | 1 |
| 923 | TF3C2_HUMAN | 101 kDa | 1 | 3 |
| 924 | IGBP1_HUMAN | 39 kDa | 2 | 6 |
| 925 | NPA1P_HUMAN | 254 kDa | 2 | 5 |
| 926 | CND3_HUMAN | 114 kDa | 5 | 3 |
| 927 | LR16C_HUMAN | 155 kDa | 4 | 2 |
| 928 | RGPA1_HUMAN | 230 kDa | 2 | 6 |
| 929 | YKT6_HUMAN | 22 kDa | 2 | 5 |
| 930 | M89BB_HUMAN | 80 kDa | 2 | 4 |
| 931 | HDAC6_HUMAN | 131 kDa | 2 | 6 |
| 932 | PPIL4_HUMAN | 57 kDa | 2 | 6 |
| 933 | ASAP1_HUMAN | 125 kDa | 2 | 6 |
| 934 | FPPS_HUMAN | 48 kDa | 2 | 4 |
| 935 | TRI72_HUMAN | 53 kDa | 5 | 1 |
| 936 | DDX54_HUMAN | 99 kDa | 5 | 1 |
| 937 | FA76B_HUMAN | 39 kDa | 3 | 4 |
| 938 | MYO6_HUMAN | 150 kDa | 3 | 1 |
| 939 | ZN444_HUMAN | 35 kDa | 4 | 3 |
| 940 | QCR2_HUMAN | 48 kDa | 3 | 1 |
| 941 | TRIPC_HUMAN | 220 kDa | 1 | 4 |
| 942 | BUD13_HUMAN | 71 kDa | 1 | 4 |
| 943 | SEP11_HUMAN | 49 kDa | 4 | 2 |
| 944 | ZA2G_HUMAN | 34 kDa | 5 | 2 |
| 945 | RBBP5_HUMAN | 59 kDa | 6 | 1 |
| 946 | THIM_HUMAN | 42 kDa | 3 | 1 |
| 947 | SON_HUMAN | 264 kDa | 3 | 0 |
| 948 | MED4_HUMAN | 30 kDa | 1 | 7 |
| 949 | TGM1_HUMAN | 90 kDa | 5 | 1 |
| 950 | TIPRL_HUMAN | 31 kDa | 1 | 6 |
| 951 | NUDC_HUMAN | 38 kDa | 5 | 1 |
| 952 | AHSA1_HUMAN | 38 kDa | 6 | 1 |
| 953 | SCLY_HUMAN | 48 kDa | 1 | 4 |
| 954 | NDKA_HUMAN | 17 kDa | 1 | 5 |
| 955 | K2C6B_HUMAN | 60 kDa | 4 | 4 |
| 956 | CPSF4_HUMAN | 30 kDa | 3 | 5 |
| 957 | ANX11_HUMAN | 54 kDa | 6 | 0 |
| 958 | GAS2_HUMAN | 35 kDa | 7 | 0 |
| 959 | PAIRB_HUMAN | 45 kDa | 0 | 4 |
| 960 | IDH3A_HUMAN | 40 kDa | 5 | 0 |
| 961 | PAPD5_HUMAN | 63 kDa | 0 | 4 |
| 962 | RL5_HUMAN | 34 kDa | 7 | 0 |
| 963 | MOV10_HUMAN | 114 kDa | 4 | 3 |
| 964 | SMC3_HUMAN | 142 kDa | 4 | 3 |
| 965 | KHDR1_HUMAN | 48 kDa | 3 | 3 |
| 966 | ORC4_HUMAN | 50 kDa | 3 | 2 |
| 967 | SKP1_HUMAN | 19 kDa | 3 | 3 |
| 968 | NADE1_HUMAN | 79 kDa | 3 | 3 |
| 969 | PRS6B_HUMAN | 47 kDa | 2 | 3 |
| 970 | BIG2_HUMAN | 202 kDa | 2 | 4 |
| 971 | GLCNE_HUMAN | 79 kDa | 4 | 3 |
| 972 | MAEA_HUMAN | 45 kDa | 3 | 4 |
| 973 | RS19_HUMAN | 16 kDa | 3 | 3 |
| 974 | PSA_HUMAN | 103 kDa | 2 | 3 |
| 975 | PSDE_HUMAN | 35 kDa | 3 | 3 |
| 976 | ASCC1_HUMAN | 46 kDa | 2 | 3 |
| 977 | INT1_HUMAN | 244 kDa | 4 | 1 |
| 978 | FACD2_HUMAN | 166 kDa | 2 | 3 |
| 979 | MARE1_HUMAN | 30 kDa | 4 | 1 |
| 980 | RAB5A_HUMAN | 24 kDa | 2 | 3 |
| 981 | EI2BB_HUMAN | 39 kDa | 4 | 2 |
| 982 | TPPC1_HUMAN | 17 kDa | 2 | 1 |
| 983 | CN043_HUMAN | 115 kDa | 3 | 2 |
| 984 | CNDG2_HUMAN | 131 kDa | 3 | 2 |
| 985 | HELZ_HUMAN | 219 kDa | 2 | 4 |
| 986 | THOC6_HUMAN | 38 kDa | 2 | 4 |
| 987 | BP16L_HUMAN (+1) | 42 kDa | 3 | 2 |
| 988 | ELMO2_HUMAN | 83 kDa | 4 | 2 |
| 989 | MVP_HUMAN | 99 kDa | 5 | 2 |
| 990 | KS6A1_HUMAN | 83 kDa | 2 | 4 |
| 991 | GEMI5_HUMAN | 169 kDa | 2 | 4 |
| 992 | COF1_HUMAN | 19 kDa | 2 | 5 |
| 993 | NUP85_HUMAN | 75 kDa | 2 | 5 |
| 994 | UBL4A_HUMAN | 18 kDa | 4 | 2 |
| 995 | S23IP_HUMAN | 111 kDa | 4 | 2 |
| 996 | UGGG1_HUMAN | 177 kDa | 3 | 2 |
| 997 | DDX50_HUMAN | 83 kDa | 2 | 4 |
| 998 | RCD1_HUMAN | 34 kDa | 2 | 4 |
| 999 | WRIP1_HUMAN | 72 kDa | 3 | 3 |
| 1000 | CGL_HUMAN | 45 kDa | 2 | 2 |
| 1001 | D3D2_HUMAN | 33 kDa | 4 | 2 |
| 1002 | RAB5C_HUMAN | 23 kDa | 4 | 3 |
| 1003 | RL12_HUMAN | 18 kDa | 5 | 2 |
| 1004 | STAT1_HUMAN | 87 kDa | 4 | 3 |
| 1005 | ASCC2_HUMAN | 86 kDa | 2 | 4 |
| 1006 | CH10_HUMAN | 11 kDa | 4 | 2 |
| 1007 | HMGB2_HUMAN | 24 kDa | 1 | 5 |
| 1008 | TADBP_HUMAN | 45 kDa | 3 | 2 |
| 1009 | NUSAP_HUMAN | 49 kDa | 1 | 4 |
| 1010 | DUS11_HUMAN | 39 kDa | 4 | 1 |
| 1011 | MNX1_HUMAN | 41 kDa | 2 | 4 |
| 1012 | SNG2_HUMAN | 25 kDa | 4 | 3 |
| 1013 | GUAA_HUMAN | 77 kDa | 1 | 5 |
| 1014 | K2C7_HUMAN | 51 kDa | 2 | 3 |
| 1015 | AKP13_HUMAN | 308 kDa | 3 | 1 |
| 1016 | SESN2_HUMAN | 54 kDa | 1 | 5 |
| 1017 | SFRS1_HUMAN | 28 kDa | 1 | 3 |
| 1018 | PDCD6_HUMAN | 22 kDa | 3 | 1 |
| 1019 | CTCF_HUMAN | 83 kDa | 1 | 3 |
| 1020 | ARAF_HUMAN | 68 kDa | 2 | 3 |
| 1021 | IDH3G_HUMAN | 43 kDa | 2 | 1 |
| 1022 | EIF2A_HUMAN | 65 kDa | 5 | 1 |
| 1023 | SNX18_HUMAN | 69 kDa | 1 | 4 |
| 1024 | ATG2B_HUMAN | 233 kDa | 1 | 3 |
| 1025 | LRWD1_HUMAN | 71 kDa | 1 | 6 |
| 1026 | PLCG2_HUMAN | 148 kDa | 5 | 1 |
| 1027 | K0020_HUMAN | 74 kDa | 6 | 1 |
| 1028 | PIMT_HUMAN | 25 kDa | 1 | 5 |
| 1029 | SC22B_HUMAN | 25 kDa | 5 | 1 |
| 1030 | HMMR_HUMAN | 84 kDa | 1 | 4 |
| 1031 | CT004_HUMAN | 43 kDa | 6 | 1 |
| 1032 | SRP72_HUMAN | 75 kDa | 6 | 1 |
| 1033 | ZBT24_HUMAN | 78 kDa | 1 | 5 |
| 1034 | ZN691_HUMAN | 36 kDa | 1 | 5 |
| 1035 | SENP1_HUMAN | 73 kDa | 1 | 5 |
| 1036 | TAF3_HUMAN | 104 kDa | 2 | 3 |
| 1037 | INT3_HUMAN | 118 kDa | 2 | 3 |
| 1038 | PDIA4_HUMAN | 73 kDa | 5 | 1 |
| 1039 | BIG1_HUMAN | 209 kDa | 2 | 4 |
| 1040 | CTIF_HUMAN | 68 kDa | 1 | 5 |
| 1041 | FUMH_HUMAN | 55 kDa | 5 | 1 |
| 1042 | ZN174_HUMAN | 46 kDa | 1 | 6 |
| 1043 | ARID2_HUMAN | 197 kDa | 3 | 2 |
| 1044 | CBX5_HUMAN | 22 kDa | 2 | 4 |
| 1045 | MAF_HUMAN | 38 kDa | 4 | 1 |
| 1046 | AIMP2_HUMAN | 35 kDa | 3 | 2 |
| 1047 | NACA_HUMAN | 23 kDa | 5 | 2 |
| 1048 | CKAP5_HUMAN | 226 kDa | 0 | 4 |
| 1049 | AGO2_HUMAN | 97 kDa | 0 | 6 |
| 1050 | CAN1_HUMAN | 82 kDa | 5 | 0 |
| 1051 | SYCC_HUMAN | 85 kDa | 4 | 0 |
| 1052 | BZW2_HUMAN | 48 kDa | 6 | 0 |
| 1053 | PELO_HUMAN | 43 kDa | 6 | 0 |
| 1054 | K22O_HUMAN | 66 kDa | 6 | 0 |
| 1055 | FTSJ2_HUMAN | 95 kDa | 0 | 4 |
| 1056 | KLF12_HUMAN | 44 kDa | 0 | 3 |
| 1057 | BLMH_HUMAN | 53 kDa | 5 | 0 |
| 1058 | EIF3I_HUMAN | 37 kDa | 3 | 3 |
| 1059 | NAGK_HUMAN | 37 kDa | 3 | 3 |
| 1060 | PAK1_HUMAN | 61 kDa | 3 | 3 |
| 1061 | RL32_HUMAN | 16 kDa | 3 | 3 |
| 1062 | TF3C5_HUMAN | 60 kDa | 3 | 3 |
| 1063 | LRCH4_HUMAN | 73 kDa | 2 | 3 |
| 1064 | CPNS1_HUMAN | 28 kDa | 3 | 2 |
| 1065 | SMG8_HUMAN | 110 kDa | 2 | 2 |
| 1066 | NP1L1_HUMAN | 45 kDa | 2 | 2 |
| 1067 | HBS1L_HUMAN | 75 kDa | 2 | 3 |
| 1068 | ORC2_HUMAN | 66 kDa | 2 | 2 |
| 1069 | PELP1_HUMAN | 120 kDa | 2 | 2 |
| 1070 | LGMN_HUMAN | 49 kDa | 1 | 2 |
| 1071 | KIF15_HUMAN | 160 kDa | 1 | 3 |
| 1072 | GTPB1_HUMAN | 72 kDa | 3 | 1 |
| 1073 | ADPPT_HUMAN | 36 kDa | 2 | 4 |
| 1074 | CSDE1_HUMAN | 89 kDa | 4 | 2 |
| 1075 | TFR1_HUMAN | 85 kDa | 4 | 2 |
| 1076 | CDC2_HUMAN | 34 kDa | 2 | 3 |
| 1077 | IF16_HUMAN | 88 kDa | 3 | 2 |
| 1078 | PTN6_HUMAN | 68 kDa | 2 | 3 |
| 1079 | MRE11_HUMAN | 81 kDa | 3 | 2 |
| 1080 | CNN2_HUMAN | 34 kDa | 3 | 2 |
| 1081 | HS74L_HUMAN | 94 kDa | 3 | 2 |
| 1082 | RCOR1_HUMAN | 53 kDa | 4 | 2 |
| 1083 | JAK1_HUMAN | 133 kDa | 2 | 2 |
| 1084 | NU205_HUMAN | 228 kDa | 2 | 2 |
| 1085 | CARF_HUMAN | 61 kDa | 3 | 2 |
| 1086 | TF3C1_HUMAN | 239 kDa | 3 | 1 |
| 1087 | ELOB_HUMAN | 13 kDa | 1 | 2 |
| 1088 | ROA0_HUMAN | 31 kDa | 2 | 2 |
| 1089 | TRP13_HUMAN | 49 kDa | 2 | 2 |
| 1090 | TDIF1_HUMAN | 37 kDa | 4 | 1 |
| 1091 | K1632_HUMAN | 292 kDa | 2 | 2 |
| 1092 | ZFP37_HUMAN | 71 kDa | 1 | 2 |
| 1093 | ZN609_HUMAN | 151 kDa | 2 | 3 |
| 1094 | APT_HUMAN | 20 kDa | 1 | 4 |
| 1095 | TXNL1_HUMAN | 32 kDa | 1 | 2 |
| 1096 | RBBP6_HUMAN | 202 kDa | 1 | 2 |
| 1097 | CX056_HUMAN | 26 kDa | 4 | 2 |
| 1098 | RL11_HUMAN | 20 kDa | 2 | 4 |
| 1099 | SMN_HUMAN | 32 kDa | 2 | 2 |
| 1100 | SPB3_HUMAN | 45 kDa | 3 | 2 |
| 1101 | F117A_HUMAN | 48 kDa | 4 | 1 |
| 1102 | NFYC_HUMAN | 50 kDa | 3 | 2 |
| 1103 | PMVK_HUMAN | 22 kDa | 3 | 1 |
| 1104 | ARP3_HUMAN | 47 kDa | 2 | 1 |
| 1105 | INT4_HUMAN | 108 kDa | 3 | 1 |
| 1106 | PRPK_HUMAN | 28 kDa | 3 | 1 |
| 1107 | RL35A_HUMAN | 13 kDa | 2 | 1 |
| 1108 | CHD7_HUMAN | 336 kDa | 1 | 3 |
| 1109 | ZN184_HUMAN | 86 kDa | 1 | 3 |
| 1110 | ANFY1_HUMAN | 128 kDa | 2 | 0 |
| 1111 | TNAP3_HUMAN | 90 kDa | 1 | 2 |
| 1112 | RRAGC_HUMAN | 44 kDa | 1 | 2 |
| 1113 | DEN4C_HUMAN | 187 kDa | 1 | 3 |
| 1114 | ARHGC_HUMAN | 173 kDa | 2 | 2 |
| 1115 | RS30_HUMAN | 7 kDa | 4 | 1 |
| 1116 | MINT_HUMAN | 402 kDa | 1 | 3 |
| 1117 | DDX20_HUMAN | 92 kDa | 2 | 1 |
| 1118 | SHIP1_HUMAN | 133 kDa | 3 | 1 |
| 1119 | PACAP_HUMAN | 21 kDa | 2 | 3 |
| 1120 | CD2B2_HUMAN | 38 kDa | 4 | 1 |
| 1121 | ZCHC3_HUMAN | 44 kDa | 3 | 2 |
| 1122 | ZN131_HUMAN | 71 kDa | 1 | 3 |
| 1123 | RHG21_HUMAN | 217 kDa | 3 | 1 |
| 1124 | UBP36_HUMAN | 123 kDa | 2 | 0 |
| 1125 | RENT1_HUMAN | 124 kDa | 4 | 0 |
| 1126 | SDS3_HUMAN | 38 kDa | 2 | 0 |
| 1127 | ASNA_HUMAN | 39 kDa | 4 | 0 |
| 1128 | AKAP2_HUMAN | 95 kDa | 5 | 1 |
| 1129 | ANXA7_HUMAN | 53 kDa | 3 | 1 |
| 1130 | UBP42_HUMAN | 146 kDa | 1 | 4 |
| 1131 | EI2BG_HUMAN | 50 kDa | 4 | 1 |
| 1132 | WDR1_HUMAN | 66 kDa | 4 | 1 |
| 1133 | PA1B3_HUMAN | 26 kDa | 1 | 4 |
| 1134 | SUGT1_HUMAN | 41 kDa | 3 | 1 |
| 1135 | LYPA1_HUMAN | 25 kDa | 1 | 5 |
| 1136 | OST48_HUMAN | 51 kDa | 5 | 1 |
| 1137 | PPIG_HUMAN | 89 kDa | 1 | 4 |
| 1138 | RAB10_HUMAN | 23 kDa | 4 | 1 |
| 1139 | TNKS1_HUMAN | 142 kDa | 1 | 5 |
| 1140 | AMPD3_HUMAN | 89 kDa | 4 | 1 |
| 1141 | PLRG1_HUMAN | 57 kDa | 1 | 3 |
| 1142 | EIF3H_HUMAN | 40 kDa | 4 | 1 |
| 1143 | PHB_HUMAN | 30 kDa | 3 | 1 |
| 1144 | EXOC5_HUMAN | 82 kDa | 3 | 1 |
| 1145 | DCPS_HUMAN | 39 kDa | 2 | 1 |
| 1146 | MYST1_HUMAN | 52 kDa | 1 | 2 |
| 1147 | NEDD1_HUMAN | 72 kDa | 1 | 5 |
| 1148 | TBP_HUMAN | 38 kDa | 3 | 1 |
| 1149 | PRD15_HUMAN | 169 kDa | 1 | 2 |
| 1150 | OGFR_HUMAN | 73 kDa | 2 | 2 |
| 1151 | UBP22_HUMAN | 60 kDa | 2 | 3 |
| 1152 | CDSN_HUMAN | 51 kDa | 3 | 2 |
| 1153 | RUXF_HUMAN | 10 kDa | 1 | 5 |
| 1154 | TAF10_HUMAN | 22 kDa | 2 | 4 |
| 1155 | IN80B_HUMAN | 39 kDa | 3 | 0 |
| 1156 | TEX10_HUMAN | 106 kDa | 2 | 0 |
| 1157 | SHIP2_HUMAN | 139 kDa | 4 | 0 |
| 1158 | 1433S_HUMAN | 28 kDa | 5 | 0 |
| 1159 | HP1B3_HUMAN | 61 kDa | 6 | 0 |
| 1160 | MYO5A_HUMAN | 215 kDa | 5 | 0 |
| 1161 | FKBP5_HUMAN | 51 kDa | 5 | 0 |
| 1162 | K1310_HUMAN | 96 kDa | 6 | 0 |
| 1163 | GLRX3_HUMAN | 37 kDa | 4 | 0 |
| 1164 | MEIS1_HUMAN | 43 kDa | 0 | 4 |
| 1165 | ZN192_HUMAN | 66 kDa | 0 | 3 |
| 1166 | ARI5B_HUMAN | 132 kDa | 0 | 6 |
| 1167 | ARPC2_HUMAN | 34 kDa | 2 | 3 |
| 1168 | COPZ1_HUMAN | 20 kDa | 2 | 3 |
| 1169 | CSTF3_HUMAN | 83 kDa | 3 | 2 |
| 1170 | DC1L1_HUMAN | 57 kDa | 3 | 2 |
| 1171 | EIF3F_HUMAN | 38 kDa | 3 | 2 |
| 1172 | EXOS4_HUMAN | 26 kDa | 3 | 2 |
| 1173 | FBX21_HUMAN | 72 kDa | 2 | 3 |
| 1174 | GTPB2_HUMAN | 66 kDa | 2 | 3 |
| 1175 | IF5A1_HUMAN | 17 kDa | 3 | 2 |
| 1176 | IMA2_HUMAN | 58 kDa | 2 | 3 |
| 1177 | INT6_HUMAN | 100 kDa | 3 | 2 |
| 1178 | INT8_HUMAN | 113 kDa | 2 | 3 |
| 1179 | MTMRC_HUMAN | 86 kDa | 2 | 3 |
| 1180 | NXF1_HUMAN | 70 kDa | 2 | 3 |
| 1181 | PPM1B_HUMAN | 53 kDa | 3 | 2 |
| 1182 | PRDX3_HUMAN | 28 kDa | 3 | 2 |
| 1183 | SKA3_HUMAN | 46 kDa | 3 | 2 |
| 1184 | THOC5_HUMAN | 79 kDa | 3 | 2 |
| 1185 | UTP15_HUMAN | 58 kDa | 3 | 2 |
| 1186 | NDRG1_HUMAN | 43 kDa | 2 | 2 |
| 1187 | T22D4_HUMAN | 41 kDa | 2 | 1 |
| 1188 | NEK9_HUMAN | 107 kDa | 2 | 2 |
| 1189 | MED16_HUMAN | 97 kDa | 2 | 1 |
| 1190 | CNO6L_HUMAN | 63 kDa | 2 | 2 |
| 1191 | TAF11_HUMAN | 23 kDa | 2 | 2 |
| 1192 | MLL1_HUMAN | 432 kDa | 2 | 2 |
| 1193 | 6PGD_HUMAN | 53 kDa | 2 | 2 |
| 1194 | KPB2_HUMAN | 138 kDa | 2 | 2 |
| 1195 | ZBT43_HUMAN | 53 kDa | 2 | 2 |
| 1196 | BOD1L_HUMAN | 330 kDa | 3 | 2 |
| 1197 | CLIC1_HUMAN | 27 kDa | 2 | 3 |
| 1198 | DKC1_HUMAN | 58 kDa | 2 | 2 |
| 1199 | DYL2_HUMAN | 10 kDa | 2 | 3 |
| 1200 | F10A1_HUMAN (+1) | 41 kDa | 2 | 2 |
| 1201 | KAP2_HUMAN | 46 kDa | 3 | 2 |
| 1202 | RB11B_HUMAN | 24 kDa | 2 | 3 |
| 1203 | RPR1B_HUMAN | 37 kDa | 3 | 2 |
| 1204 | SCRIB_HUMAN | 175 kDa | 2 | 2 |
| 1205 | ZKSC1_HUMAN | 64 kDa | 2 | 3 |
| 1206 | NSUN5_HUMAN | 47 kDa | 2 | 1 |
| 1207 | TPM3_HUMAN | 33 kDa | 2 | 1 |
| 1208 | MEP50_HUMAN | 37 kDa | 2 | 1 |
| 1209 | ZN316_HUMAN | 108 kDa | 1 | 3 |
| 1210 | DEFI6_HUMAN | 74 kDa | 1 | 2 |
| 1211 | ARP2_HUMAN | 45 kDa | 1 | 2 |
| 1212 | PEBP1_HUMAN | 21 kDa | 1 | 2 |
| 1213 | CSN3_HUMAN | 48 kDa | 2 | 2 |
| 1214 | CSN6_HUMAN | 36 kDa | 3 | 1 |
| 1215 | CALL5_HUMAN | 16 kDa | 1 | 3 |
| 1216 | RICTR_HUMAN | 192 kDa | 2 | 1 |
| 1217 | DOC10_HUMAN | 249 kDa | 1 | 2 |
| 1218 | F120B_HUMAN | 104 kDa | 1 | 2 |
| 1219 | TPPC4_HUMAN | 24 kDa | 2 | 1 |
| 1220 | MPP8_HUMAN | 97 kDa | 3 | 1 |
| 1221 | DDX41_HUMAN | 70 kDa | 1 | 2 |
| 1222 | PFD5_HUMAN | 17 kDa | 3 | 1 |
| 1223 | MRT4_HUMAN | 28 kDa | 2 | 1 |
| 1224 | PHF6_HUMAN | 41 kDa | 1 | 2 |
| 1225 | DGCR8_HUMAN | 86 kDa | 2 | 1 |
| 1226 | GEMI4_HUMAN | 120 kDa | 2 | 1 |
| 1227 | TF3C3_HUMAN | 101 kDa | 1 | 3 |
| 1228 | GCF_HUMAN | 89 kDa | 3 | 1 |
| 1229 | H1X_HUMAN | 22 kDa | 1 | 2 |
| 1230 | ENY2_HUMAN | 12 kDa | 0 | 2 |
| 1231 | UB2V2_HUMAN | 16 kDa | 2 | 0 |
| 1232 | CTBL1_HUMAN | 65 kDa | 4 | 1 |
| 1233 | CO044_HUMAN | 57 kDa | 1 | 3 |
| 1234 | ERF3A_HUMAN | 56 kDa | 3 | 1 |
| 1235 | SPT5H_HUMAN | 121 kDa | 3 | 1 |
| 1236 | GSHR_HUMAN | 56 kDa | 1 | 3 |
| 1237 | RL9_HUMAN | 22 kDa | 3 | 1 |
| 1238 | CD043_HUMAN | 24 kDa | 3 | 1 |
| 1239 | ERH_HUMAN | 12 kDa | 1 | 3 |
| 1240 | GCP6_HUMAN | 200 kDa | 3 | 1 |
| 1241 | HPBP1_HUMAN | 39 kDa | 4 | 1 |
| 1242 | ICLN_HUMAN | 26 kDa | 4 | 1 |
| 1243 | LMAN2_HUMAN | 40 kDa | 3 | 1 |
| 1244 | NAT11_HUMAN | 27 kDa | 1 | 4 |
| 1245 | PLD3_HUMAN | 55 kDa | 4 | 1 |
| 1246 | RAB21_HUMAN | 24 kDa | 4 | 1 |
| 1247 | SCAM3_HUMAN | 38 kDa | 4 | 1 |
| 1248 | SNX6_HUMAN | 47 kDa | 4 | 1 |
| 1249 | TPX2_HUMAN | 86 kDa | 4 | 1 |
| 1250 | IP6K1_HUMAN | 50 kDa | 1 | 3 |
| 1251 | MUS81_HUMAN | 61 kDa | 1 | 3 |
| 1252 | AAKB1_HUMAN | 30 kDa | 1 | 2 |
| 1253 | LA_HUMAN | 47 kDa | 2 | 1 |
| 1254 | RING2_HUMAN | 38 kDa | 3 | 1 |
| 1255 | NU160_HUMAN | 162 kDa | 1 | 4 |
| 1256 | ARD1A_HUMAN | 26 kDa | 3 | 1 |
| 1257 | RS27L_HUMAN | 9 kDa | 1 | 2 |
| 1258 | RIOK3_HUMAN | 59 kDa | 1 | 2 |
| 1259 | AMER1_HUMAN | 35 kDa | 1 | 2 |
| 1260 | CE170_HUMAN | 175 kDa | 2 | 2 |
| 1261 | ABLM1_HUMAN | 88 kDa | 1 | 3 |
| 1262 | FNTA_HUMAN | 44 kDa | 3 | 1 |
| 1263 | ALDOC_HUMAN | 39 kDa | 2 | 2 |
| 1264 | HEM2_HUMAN | 36 kDa | 2 | 3 |
| 1265 | IMA1_HUMAN | 60 kDa | 2 | 3 |
| 1266 | PSB3_HUMAN | 23 kDa | 1 | 3 |
| 1267 | CLIC2_HUMAN | 28 kDa | 4 | 0 |
| 1268 | XPP1_HUMAN | 70 kDa | 3 | 0 |
| 1269 | T22D1_HUMAN | 110 kDa | 0 | 2 |
| 1270 | KLF16_HUMAN | 25 kDa | 0 | 3 |
| 1271 | EI2BD_HUMAN | 58 kDa | 4 | 0 |
| 1272 | RFA1_HUMAN | 68 kDa | 2 | 0 |
| 1273 | TPD52_HUMAN | 24 kDa | 4 | 0 |
| 1274 | SCFD1_HUMAN | 72 kDa | 4 | 0 |
| 1275 | UBR1_HUMAN-R | ? | 2 | 0 |
| 1276 | BPTF_HUMAN | 338 kDa | 4 | 0 |
| 1277 | INT7_HUMAN | 107 kDa | 4 | 0 |
| 1278 | KIF1A_HUMAN | 191 kDa | 2 | 0 |
| 1279 | HNRPG_HUMAN | 42 kDa | 5 | 0 |
| 1280 | MTHR_HUMAN | 75 kDa | 5 | 0 |
| 1281 | SYAC_HUMAN | 107 kDa | 5 | 0 |
| 1282 | TAF4B_HUMAN | 91 kDa | 5 | 0 |
| 1283 | DNJC7_HUMAN | 56 kDa | 4 | 0 |
| 1284 | PP1R7_HUMAN | 42 kDa | 4 | 0 |
| 1285 | ERCC3_HUMAN | 89 kDa | 0 | 3 |
| 1286 | 1433F_HUMAN | 28 kDa | 4 | 0 |
| 1287 | EDC4_HUMAN | 152 kDa | 4 | 0 |
| 1288 | TJAP1_HUMAN | 62 kDa | 4 | 0 |
| 1289 | BRX1_HUMAN | 41 kDa | 3 | 0 |
| 1290 | ORC3_HUMAN | 82 kDa | 0 | 3 |
| 1291 | IN80D_HUMAN | 98 kDa | 4 | 0 |
| 1292 | LEO1_HUMAN | 75 kDa | 0 | 3 |
| 1293 | CHRD1_HUMAN | 37 kDa | 5 | 0 |
| 1294 | CI082_HUMAN | 38 kDa | 0 | 5 |
| 1295 | HNRPQ_HUMAN | 70 kDa | 5 | 0 |
| 1296 | K1C27_HUMAN | 50 kDa | 5 | 0 |
| 1297 | PR38B_HUMAN | 64 kDa | 0 | 3 |
| 1298 | ZC3HE_HUMAN | 83 kDa | 4 | 0 |
| 1299 | ZN490_HUMAN | 61 kDa | 0 | 3 |
| 1300 | LMNB1_HUMAN | 66 kDa | 4 | 0 |
| 1301 | AATF_HUMAN | 63 kDa | 2 | 2 |
| 1302 | AP2A1_HUMAN | 108 kDa | 2 | 2 |
| 1303 | BANP_HUMAN | 56 kDa | 2 | 2 |
| 1304 | BECN1_HUMAN | 52 kDa | 2 | 2 |
| 1305 | BRWD2_HUMAN | 137 kDa | 2 | 2 |
| 1306 | DMAP1_HUMAN | 53 kDa | 2 | 2 |
| 1307 | ES1_HUMAN | 28 kDa | 2 | 2 |
| 1308 | FNTB_HUMAN | 49 kDa | 2 | 2 |
| 1309 | GALK1_HUMAN | 42 kDa | 2 | 2 |
| 1310 | GGCT_HUMAN | 21 kDa | 2 | 2 |
| 1311 | HAUS2_HUMAN | 27 kDa | 2 | 2 |
| 1312 | HINT2_HUMAN | 17 kDa | 2 | 2 |
| 1313 | INT5_HUMAN | 108 kDa | 2 | 2 |
| 1314 | LST8_HUMAN | 36 kDa | 2 | 2 |
| 1315 | MAGD2_HUMAN | 65 kDa | 2 | 2 |
| 1316 | MDHC_HUMAN | 36 kDa | 2 | 2 |
| 1317 | MPCP_HUMAN | 40 kDa | 2 | 2 |
| 1318 | RAB2A_HUMAN | 24 kDa | 2 | 2 |
| 1319 | RL35_HUMAN | 15 kDa | 2 | 2 |
| 1320 | RPN2_HUMAN | 69 kDa | 2 | 2 |
| 1321 | RS25_HUMAN | 14 kDa | 2 | 2 |
| 1322 | SMG9_HUMAN | 58 kDa | 2 | 2 |
| 1323 | SPCS_HUMAN | 56 kDa | 2 | 2 |
| 1324 | TBCD7_HUMAN | 34 kDa | 2 | 2 |
| 1325 | TSN_HUMAN | 26 kDa | 2 | 2 |
| 1326 | CPNE1_HUMAN | 59 kDa | 2 | 2 |
| 1327 | AN32E_HUMAN | 31 kDa | 2 | 1 |
| 1328 | NUP37_HUMAN | 37 kDa | 2 | 1 |
| 1329 | AXN1_HUMAN | 96 kDa | 2 | 1 |
| 1330 | NSUN2_HUMAN | 86 kDa | 1 | 2 |
| 1331 | RFC4_HUMAN | 40 kDa | 1 | 2 |
| 1332 | CSTF1_HUMAN | 48 kDa | 2 | 1 |
| 1333 | SAMH1_HUMAN | 72 kDa | 2 | 1 |
| 1334 | INT11_HUMAN | 68 kDa | 1 | 2 |
| 1335 | PKHA7_HUMAN | 127 kDa | 2 | 1 |
| 1336 | MTMR6_HUMAN | 72 kDa | 1 | 2 |
| 1337 | CN080_HUMAN | 54 kDa | 1 | 2 |
| 1338 | FKBP4_HUMAN | 52 kDa | 2 | 1 |
| 1339 | MYST4_HUMAN | 231 kDa | 1 | 2 |
| 1340 | MED12_HUMAN | 243 kDa | 1 | 2 |
| 1341 | ZN828_HUMAN | 89 kDa | 2 | 0 |
| 1342 | RPRD2_HUMAN | 156 kDa | 2 | 0 |
| 1343 | BRD7_HUMAN | 74 kDa | 3 | 1 |
| 1344 | CEP97_HUMAN | 97 kDa | 3 | 1 |
| 1345 | GCP5_HUMAN | 118 kDa | 3 | 1 |
| 1346 | IF4E_HUMAN | 25 kDa | 3 | 1 |
| 1347 | KBTB2_HUMAN | 71 kDa | 1 | 3 |
| 1348 | LAS1L_HUMAN | 83 kDa | 1 | 3 |
| 1349 | MED17_HUMAN | 73 kDa | 3 | 1 |
| 1350 | MTA2_HUMAN | 75 kDa | 3 | 1 |
| 1351 | RPB1_HUMAN | 217 kDa | 3 | 1 |
| 1352 | TKT_HUMAN | 68 kDa | 1 | 3 |
| 1353 | TMED9_HUMAN | 27 kDa | 3 | 1 |
| 1354 | TRI25_HUMAN | 71 kDa | 3 | 1 |
| 1355 | URB2_HUMAN | 171 kDa | 3 | 1 |
| 1356 | ZBT40_HUMAN | 138 kDa | 1 | 3 |
| 1357 | HEM3_HUMAN | 39 kDa | 2 | 1 |
| 1358 | TMEDA_HUMAN | 25 kDa | 2 | 1 |
| 1359 | CG020_HUMAN | 37 kDa | 2 | 1 |
| 1360 | ZN552_HUMAN | 46 kDa | 1 | 2 |
| 1361 | DCTN2_HUMAN | 44 kDa | 2 | 1 |
| 1362 | ARI1B_HUMAN | 236 kDa | 2 | 1 |
| 1363 | AT1A1_HUMAN | 113 kDa | 2 | 1 |
| 1364 | RASF4_HUMAN | 37 kDa | 2 | 1 |
| 1365 | DDX51_HUMAN | 72 kDa | 2 | 1 |
| 1366 | ZPR1_HUMAN | 51 kDa | 1 | 2 |
| 1367 | MPI_HUMAN | 47 kDa | 2 | 1 |
| 1368 | VPS39_HUMAN | 102 kDa | 2 | 1 |
| 1369 | WDR48_HUMAN | 76 kDa | 1 | 2 |
| 1370 | SMG1_HUMAN | 410 kDa | 2 | 1 |
| 1371 | CN021_HUMAN | 69 kDa | 1 | 2 |
| 1372 | CYTSA_HUMAN | 125 kDa | 1 | 2 |
| 1373 | UBE2K_HUMAN | 22 kDa | 2 | 1 |
| 1374 | CBS_HUMAN | 61 kDa | 2 | 1 |
| 1375 | PWP1_HUMAN | 56 kDa | 2 | 1 |
| 1376 | ARHG6_HUMAN | 88 kDa | 2 | 2 |
| 1377 | ATG3_HUMAN | 36 kDa | 1 | 2 |
| 1378 | BRCC3_HUMAN | 36 kDa | 3 | 1 |
| 1379 | CAZA2_HUMAN | 33 kDa | 2 | 2 |
| 1380 | COR1C_HUMAN | 53 kDa | 3 | 1 |
| 1381 | COX2_HUMAN | 26 kDa | 2 | 2 |
| 1382 | IQEC1_HUMAN | 108 kDa | 2 | 2 |
| 1383 | LUC7L_HUMAN | 44 kDa | 1 | 3 |
| 1384 | MI4GD_HUMAN | 25 kDa | 1 | 3 |
| 1385 | NU107_HUMAN | 106 kDa | 2 | 2 |
| 1386 | RINI_HUMAN | 50 kDa | 3 | 1 |
| 1387 | S10AB_HUMAN | 12 kDa | 1 | 3 |
| 1388 | SET_HUMAN | 33 kDa | 2 | 1 |
| 1389 | TBC17_HUMAN | 73 kDa | 1 | 3 |
| 1390 | UBC12_HUMAN | 21 kDa | 2 | 1 |
| 1391 | XPO5_HUMAN | 136 kDa | 1 | 3 |
| 1392 | ZF64B_HUMAN | 72 kDa | 1 | 3 |
| 1393 | RFA2_HUMAN | 29 kDa | 3 | 0 |
| 1394 | IDH3B_HUMAN | 42 kDa | 3 | 0 |
| 1395 | UIF_HUMAN | 36 kDa | 0 | 3 |
| 1396 | VTNC_HUMAN | 54 kDa | 0 | 3 |
| 1397 | DTNB_HUMAN | 71 kDa | 3 | 0 |
| 1398 | HNRH3_HUMAN | 37 kDa | 2 | 0 |
| 1399 | HNRPD_HUMAN | 38 kDa | 3 | 0 |
| 1400 | MAOM_HUMAN | 65 kDa | 0 | 2 |
| 1401 | POGZ_HUMAN | 155 kDa | 0 | 2 |
| 1402 | NOC2L_HUMAN | 85 kDa | 0 | 3 |
| 1403 | MP2K1_HUMAN | 43 kDa | 2 | 0 |
| 1404 | F128A_HUMAN (+1) | 16 kDa | 0 | 3 |
| 1405 | ZN777_HUMAN | 85 kDa | 2 | 0 |
| 1406 | CKAP4_HUMAN | 66 kDa | 4 | 0 |
| 1407 | CYBP_HUMAN | 26 kDa | 4 | 0 |
| 1408 | EBP2_HUMAN | 35 kDa | 4 | 0 |
| 1409 | EIF3G_HUMAN | 36 kDa | 4 | 0 |
| 1410 | HAUS6_HUMAN | 109 kDa | 4 | 0 |
| 1411 | OSBL3_HUMAN | 101 kDa | 4 | 0 |
| 1412 | RNC_HUMAN | 159 kDa | 0 | 4 |
| 1413 | SET1A_HUMAN | 186 kDa | 4 | 0 |
| 1414 | UBA5_HUMAN | 45 kDa | 0 | 4 |
| 1415 | SEPT1_HUMAN | 42 kDa | 3 | 0 |
| 1416 | WIZ_HUMAN | 179 kDa | 3 | 0 |
| 1417 | NOP2_HUMAN | 89 kDa | 3 | 0 |
| 1418 | VINC_HUMAN | 124 kDa | 3 | 0 |
| 1419 | CH041_HUMAN | 57 kDa | 0 | 3 |
| 1420 | WBP11_HUMAN | 70 kDa | 3 | 0 |
| 1421 | RHG30_HUMAN | 119 kDa | 2 | 0 |
| 1422 | DPY30_HUMAN | 11 kDa | 0 | 4 |
| 1423 | FCL_HUMAN | 36 kDa | 3 | 0 |
| 1424 | IF2BL_HUMAN (+1) | 38 kDa | 3 | 0 |
| 1425 | K2C3_HUMAN | 65 kDa | 4 | 0 |
| 1426 | SF3B5_HUMAN | 10 kDa | 0 | 4 |
| 1427 | SRPRB_HUMAN | 30 kDa | 4 | 0 |
| 1428 | TOM34_HUMAN | 35 kDa | 3 | 0 |
| 1429 | NT5D2_HUMAN | 61 kDa | 0 | 2 |
| 1430 | RSSA_HUMAN | 33 kDa | 0 | 3 |
| 1431 | NARG1_HUMAN | 101 kDa | 2 | 0 |
| 1432 | CAPR1_HUMAN | 78 kDa | 2 | 0 |
| 1433 | FUBP1_HUMAN | 68 kDa | 2 | 0 |
| 1434 | SYYC_HUMAN | 59 kDa | 2 | 0 |
| 1435 | YTDC2_HUMAN | 160 kDa | 0 | 2 |
| 1436 | IPYR_HUMAN | 33 kDa | 2 | 0 |
| 1437 | SART3_HUMAN | 110 kDa | 2 | 0 |
| 1438 | VPS4B_HUMAN | 49 kDa | 2 | 0 |
| 1439 | PCID2_HUMAN | 46 kDa | 0 | 3 |
| 1440 | SPA12_HUMAN | 47 kDa | 2 | 0 |
| 1441 | MED14_HUMAN | 161 kDa | 0 | 2 |
| 1442 | EAF6_HUMAN | 22 kDa | 0 | 3 |
| 1443 | CA057_HUMAN | 21 kDa | 0 | 3 |
| 1444 | GCP60_HUMAN | 61 kDa | 4 | 0 |
| 1445 | REQU_HUMAN | 44 kDa | 2 | 0 |
| 1446 | ZN575_HUMAN | 27 kDa | 0 | 2 |
| 1447 | ABC3D_HUMAN | 47 kDa | 2 | 1 |
| 1448 | ANR12_HUMAN | 236 kDa | 2 | 1 |
| 1449 | ARPC4_HUMAN | 20 kDa | 1 | 2 |
| 1450 | ATPK_HUMAN | 11 kDa | 1 | 2 |
| 1451 | CCD50_HUMAN | 36 kDa | 1 | 2 |
| 1452 | CD20_HUMAN | 33 kDa | 1 | 2 |
| 1453 | CSN4_HUMAN | 46 kDa | 1 | 2 |
| 1454 | DCTN1_HUMAN | 142 kDa | 2 | 1 |
| 1455 | DDX47_HUMAN | 51 kDa | 2 | 1 |
| 1456 | EF1B_HUMAN | 25 kDa | 2 | 1 |
| 1457 | ELP2_HUMAN | 92 kDa | 1 | 2 |
| 1458 | ETFA_HUMAN | 35 kDa | 2 | 1 |
| 1459 | FA76A_HUMAN | 35 kDa | 1 | 2 |
| 1460 | GSTK1_HUMAN | 25 kDa | 1 | 2 |
| 1461 | ING2_HUMAN | 33 kDa | 1 | 2 |
| 1462 | INP4A_HUMAN | 110 kDa | 2 | 1 |
| 1463 | IVD_HUMAN | 46 kDa | 1 | 2 |
| 1464 | KIF5A_HUMAN | 117 kDa | 1 | 2 |
| 1465 | LRC47_HUMAN | 63 kDa | 2 | 1 |
| 1466 | M3K4_HUMAN | 182 kDa | 1 | 2 |
| 1467 | MED20_HUMAN | 23 kDa | 1 | 2 |
| 1468 | NCOR1_HUMAN | 270 kDa | 1 | 2 |
| 1469 | OLA1_HUMAN | 45 kDa | 1 | 2 |
| 1470 | P3C2A_HUMAN | 191 kDa | 1 | 2 |
| 1471 | PI42C_HUMAN | 47 kDa | 1 | 2 |
| 1472 | PICAL_HUMAN | 71 kDa | 2 | 1 |
| 1473 | PML_HUMAN | 98 kDa | 1 | 2 |
| 1474 | PSA6_HUMAN | 27 kDa | 2 | 1 |
| 1475 | PSMD9_HUMAN | 25 kDa | 1 | 2 |
| 1476 | RAB6A_HUMAN (+1) | 24 kDa | 2 | 1 |
| 1477 | RBM22_HUMAN | 47 kDa | 2 | 1 |
| 1478 | RCL1_HUMAN | 41 kDa | 1 | 2 |
| 1479 | RCOR3_HUMAN | 56 kDa | 2 | 1 |
| 1480 | RFC2_HUMAN | 39 kDa | 2 | 1 |
| 1481 | RL22_HUMAN | 15 kDa | 2 | 1 |
| 1482 | RL30_HUMAN | 13 kDa | 2 | 1 |
| 1483 | RS12_HUMAN | 15 kDa | 2 | 1 |
| 1484 | RS28_HUMAN | 8 kDa | 2 | 1 |
| 1485 | SAPS2_HUMAN | 105 kDa | 1 | 2 |
| 1486 | SAPS3_HUMAN | 98 kDa | 2 | 1 |
| 1487 | SFRS9_HUMAN | 26 kDa | 1 | 2 |
| 1488 | SNRPA_HUMAN | 31 kDa | 2 | 1 |
| 1489 | SPF27_HUMAN | 26 kDa | 2 | 1 |
| 1490 | SSA27_HUMAN | 21 kDa | 2 | 1 |
| 1491 | TBCD1_HUMAN | 133 kDa | 2 | 1 |
| 1492 | THOC2_HUMAN | 183 kDa | 1 | 2 |
| 1493 | TPD54_HUMAN | 22 kDa | 2 | 1 |
| 1494 | UBP24_HUMAN | 294 kDa | 1 | 2 |
| 1495 | UBQL2_HUMAN | 66 kDa | 2 | 1 |
| 1496 | YPEL5_HUMAN | 14 kDa | 2 | 1 |
| 1497 | ZBT11_HUMAN | 119 kDa | 2 | 1 |
| 1498 | ZFR_HUMAN | 117 kDa | 1 | 2 |
| 1499 | ZN451_HUMAN | 121 kDa | 2 | 1 |
| 1500 | ZWILC_HUMAN | 67 kDa | 1 | 2 |
| 1501 | CHRC1_HUMAN | 15 kDa | 2 | 1 |
| 1502 | PKCB1_HUMAN | 132 kDa | 2 | 1 |
| 1503 | NH2L1_HUMAN | 14 kDa | 2 | 0 |
| 1504 | NUDC3_HUMAN | 41 kDa | 2 | 0 |
| 1505 | PARD3_HUMAN | 151 kDa | 0 | 2 |
| 1506 | SRP14_HUMAN | 15 kDa | 2 | 0 |
| 1507 | CB043_HUMAN | 37 kDa | 0 | 2 |
| 1508 | SPEE_HUMAN | 34 kDa | 0 | 2 |
| 1509 | TPPC9_HUMAN | 129 kDa | 0 | 2 |
| 1510 | ARFP1_HUMAN | 42 kDa | 2 | 0 |
| 1511 | PINX1_HUMAN | 37 kDa | 2 | 0 |
| 1512 | OASL_HUMAN | 59 kDa | 0 | 2 |
| 1513 | CDK13_HUMAN | 165 kDa | 0 | 2 |
| 1514 | PURB_HUMAN | 33 kDa | 2 | 0 |
| 1515 | ITPA_HUMAN | 21 kDa | 0 | 2 |
| 1516 | COTL1_HUMAN | 16 kDa | 2 | 0 |
| 1517 | PSB1_HUMAN | 26 kDa | 0 | 2 |
| 1518 | RBBP8_HUMAN | 102 kDa | 2 | 0 |
| 1519 | SPT6H_HUMAN | 199 kDa | 2 | 0 |
| 1520 | AIFM1_HUMAN | 67 kDa | 0 | 3 |
| 1521 | ECHA_HUMAN | 83 kDa | 0 | 3 |
| 1522 | HIRA_HUMAN | 112 kDa | 3 | 0 |
| 1523 | LPXN_HUMAN | 43 kDa | 3 | 0 |
| 1524 | NU153_HUMAN | 154 kDa | 3 | 0 |
| 1525 | NUP88_HUMAN | 84 kDa | 3 | 0 |
| 1526 | ODPB_HUMAN | 39 kDa | 0 | 3 |
| 1527 | PRKRA_HUMAN | 34 kDa | 0 | 3 |
| 1528 | PSD10_HUMAN | 24 kDa | 0 | 3 |
| 1529 | RBM14_HUMAN | 69 kDa | 3 | 0 |
| 1530 | RT22_HUMAN | 41 kDa | 0 | 3 |
| 1531 | TB182_HUMAN | 182 kDa | 3 | 0 |
| 1532 | TBB3_HUMAN | 50 kDa | 0 | 3 |
| 1533 | TTL12_HUMAN | 74 kDa | 0 | 3 |
| 1534 | WDR26_HUMAN | 72 kDa | 0 | 3 |
| 1535 | YK023_HUMAN | 56 kDa | 0 | 3 |
| 1536 | ZGPAT_HUMAN | 57 kDa | 0 | 3 |
| 1537 | ZN263_HUMAN | 77 kDa | 3 | 0 |
| 1538 | CP088_HUMAN | 52 kDa | 0 | 3 |
| 1539 | DNJC9_HUMAN | 30 kDa | 2 | 0 |
| 1540 | KI18B_HUMAN | 92 kDa | 2 | 0 |
| 1541 | NCBP1_HUMAN | 92 kDa | 2 | 0 |
| 1542 | EHMT1_HUMAN | 138 kDa | 2 | 0 |
| 1543 | JIP4_HUMAN | 146 kDa | 2 | 0 |
| 1544 | CX023_HUMAN | 84 kDa | 0 | 2 |
| 1545 | MCA3_HUMAN | 20 kDa | 2 | 0 |
| 1546 | MYO1C_HUMAN | 122 kDa | 2 | 0 |
| 1547 | MIB1_HUMAN | 110 kDa | 2 | 0 |
| 1548 | TPC10_HUMAN | 142 kDa | 2 | 0 |
| 1549 | NUP54_HUMAN | 55 kDa | 2 | 0 |
| 1550 | MCM3A_HUMAN | 218 kDa | 0 | 2 |
| 1551 | NOP16_HUMAN | 21 kDa | 0 | 2 |
| 1552 | CCDC9_HUMAN | 60 kDa | 2 | 0 |
| 1553 | CETN2_HUMAN | 20 kDa | 0 | 2 |
| 1554 | SNX1_HUMAN | 59 kDa | 2 | 0 |
| 1555 | PCY1A_HUMAN | 42 kDa | 2 | 0 |
| 1556 | PRDM2_HUMAN | 189 kDa | 0 | 2 |
| 1557 | ARP10_HUMAN | 46 kDa | 2 | 0 |
| 1558 | PR38A_HUMAN | 37 kDa | 0 | 2 |
| 1559 | ATP5L_HUMAN | 11 kDa | 2 | 0 |
| 1560 | PLD1_HUMAN | 124 kDa | 0 | 2 |
| 1561 | NELFE_HUMAN | 43 kDa | 2 | 0 |
| 1562 | CTDP1_HUMAN | 104 kDa | 2 | 0 |
| 1563 | UBE4A_HUMAN | 123 kDa | 2 | 0 |
| 1564 | UN45A_HUMAN | 103 kDa | 2 | 0 |
| 1565 | EP400_HUMAN | 344 kDa | 2 | 0 |
| 1566 | ZKSC4_HUMAN | 62 kDa | 0 | 2 |
| 1567 | PIPNB_HUMAN | 32 kDa | 2 | 0 |
| 1568 | DACH1_HUMAN | 79 kDa | 2 | 0 |
| 1569 | MUDEN_HUMAN | 55 kDa | 0 | 2 |
| 1570 | ABI1_HUMAN | 55 kDa | 0 | 2 |
| 1571 | XPOT_HUMAN | 110 kDa | 2 | 0 |
| 1572 | RFC5_HUMAN | 38 kDa | 0 | 2 |
| 1573 | K1712_HUMAN | 44 kDa | 2 | 0 |
| 1574 | CSN8_HUMAN | 23 kDa | 2 | 0 |
| 1575 | PDE5A_HUMAN | 100 kDa | 2 | 0 |
| 1576 | IMDH1_HUMAN | 55 kDa | 2 | 0 |
| 1577 | WAC_HUMAN | 71 kDa | 0 | 2 |
| 1578 | ROAA_HUMAN | 36 kDa | 2 | 0 |
| 1579 | FABP5_HUMAN | 15 kDa | 0 | 3 |
| 1580 | FKBP3_HUMAN | 25 kDa | 3 | 0 |
| 1581 | MYCBP_HUMAN | 12 kDa | 0 | 3 |
| 1582 | PHRF1_HUMAN | 179 kDa | 0 | 2 |
| 1583 | PSA7_HUMAN | 28 kDa | 2 | 0 |
| 1584 | RWD2A_HUMAN | 34 kDa | 0 | 2 |
| 1585 | TCF20_HUMAN | 212 kDa | 0 | 2 |
| 1586 | TFAP4_HUMAN | 39 kDa | 3 | 0 |
| 1587 | AKAP8_HUMAN | 76 kDa | 2 | 0 |
| 1588 | AMPM1_HUMAN | 43 kDa | 2 | 0 |
| 1589 | AMY1_HUMAN | 58 kDa | 0 | 2 |
| 1590 | AP1M1_HUMAN | 49 kDa | 0 | 2 |
| 1591 | ARI2_HUMAN | 58 kDa | 2 | 0 |
| 1592 | ATLA3_HUMAN | 61 kDa | 2 | 0 |
| 1593 | BACH1_HUMAN | 82 kDa | 2 | 0 |
| 1594 | BAF_HUMAN | 10 kDa | 0 | 2 |
| 1595 | BAG5_HUMAN | 51 kDa | 2 | 0 |
| 1596 | BARD1_HUMAN | 87 kDa | 2 | 0 |
| 1597 | BUB1_HUMAN | 122 kDa | 2 | 0 |
| 1598 | BZW1_HUMAN | 48 kDa | 2 | 0 |
| 1599 | CATD_HUMAN | 45 kDa | 2 | 0 |
| 1600 | CD041_HUMAN | 129 kDa | 2 | 0 |
| 1601 | CDK5_HUMAN | 33 kDa | 0 | 2 |
| 1602 | COASY_HUMAN | 62 kDa | 0 | 2 |
| 1603 | COG2_HUMAN | 83 kDa | 2 | 0 |
| 1604 | CPIN1_HUMAN | 34 kDa | 2 | 0 |
| 1605 | CS010_HUMAN | 19 kDa | 2 | 0 |
| 1606 | DD19A_HUMAN (+1) | 54 kDa | 0 | 2 |
| 1607 | DDX56_HUMAN | 62 kDa | 0 | 2 |
| 1608 | DLX1_HUMAN (+3) | 27 kDa | 0 | 2 |
| 1609 | DNJB1_HUMAN | 38 kDa | 2 | 0 |
| 1610 | DVL2_HUMAN (+1) | 79 kDa | 0 | 2 |
| 1611 | EAP1_HUMAN | 83 kDa | 2 | 0 |
| 1612 | ECH1_HUMAN | 36 kDa | 2 | 0 |
| 1613 | ECHD1_HUMAN | 34 kDa | 0 | 2 |
| 1614 | ECM29_HUMAN | 204 kDa | 0 | 2 |
| 1615 | EXOC2_HUMAN | 104 kDa | 2 | 0 |
| 1616 | EXOC8_HUMAN | 82 kDa | 2 | 0 |
| 1617 | FLII_HUMAN | 145 kDa | 2 | 0 |
| 1618 | GGPPS_HUMAN | 35 kDa | 2 | 0 |
| 1619 | GLD2_HUMAN | 56 kDa | 0 | 2 |
| 1620 | GMFG_HUMAN | 17 kDa | 2 | 0 |
| 1621 | GORS2_HUMAN | 47 kDa | 2 | 0 |
| 1622 | GRWD1_HUMAN | 49 kDa | 2 | 0 |
| 1623 | HS105_HUMAN | 97 kDa | 2 | 0 |
| 1624 | HSF1_HUMAN | 57 kDa | 2 | 0 |
| 1625 | KC1A_HUMAN (+1) | 39 kDa | 0 | 2 |
| 1626 | KDM2A_HUMAN | 133 kDa | 2 | 0 |
| 1627 | KRIT1_HUMAN | 84 kDa | 2 | 0 |
| 1628 | LAT1_HUMAN | 55 kDa | 2 | 0 |
| 1629 | LMAN1_HUMAN | 58 kDa | 2 | 0 |
| 1630 | MD2L2_HUMAN | 24 kDa | 0 | 2 |
| 1631 | MDC1_HUMAN | 227 kDa | 2 | 0 |
| 1632 | MED11_HUMAN | 13 kDa | 0 | 2 |
| 1633 | MIS12_HUMAN | 24 kDa | 0 | 2 |
| 1634 | MPRD_HUMAN | 31 kDa | 2 | 0 |
| 1635 | MTMR5_HUMAN | 208 kDa | 0 | 2 |
| 1636 | MYST2_HUMAN | 71 kDa | 2 | 0 |
| 1637 | NOL8_HUMAN | 132 kDa | 2 | 0 |
| 1638 | NPL4_HUMAN | 68 kDa | 2 | 0 |
| 1639 | NSF1C_HUMAN | 41 kDa | 2 | 0 |
| 1640 | NSF_HUMAN | 83 kDa | 2 | 0 |
| 1641 | PACN3_HUMAN | 48 kDa | 2 | 0 |
| 1642 | PBX2_HUMAN | 46 kDa | 0 | 2 |
| 1643 | PDCL3_HUMAN | 28 kDa | 0 | 2 |
| 1644 | PDIP3_HUMAN | 46 kDa | 2 | 0 |
| 1645 | PI4KA_HUMAN | 231 kDa | 0 | 2 |
| 1646 | PLUNC_HUMAN | 27 kDa | 0 | 2 |
| 1647 | POF1B_HUMAN | 69 kDa | 0 | 2 |
| 1648 | PRP39_HUMAN | 78 kDa | 2 | 0 |
| 1649 | PSB5_HUMAN | 28 kDa | 2 | 0 |
| 1650 | PSMF1_HUMAN | 30 kDa | 0 | 2 |
| 1651 | PUR1_HUMAN | 57 kDa | 2 | 0 |
| 1652 | RAB8A_HUMAN | 24 kDa | 2 | 0 |
| 1653 | RAB8B_HUMAN | 24 kDa | 2 | 0 |
| 1654 | RB33A_HUMAN | 27 kDa | 2 | 0 |
| 1655 | RN213_HUMAN | 374 kDa | 2 | 0 |
| 1656 | RPAP3_HUMAN | 76 kDa | 2 | 0 |
| 1657 | RWDD4_HUMAN | 21 kDa | 2 | 0 |
| 1658 | SETD2_HUMAN | 288 kDa | 2 | 0 |
| 1659 | SFXN1_HUMAN | 36 kDa | 2 | 0 |
| 1660 | SPAS2_HUMAN | 60 kDa | 0 | 2 |
| 1661 | SPRY3_HUMAN | 50 kDa | 2 | 0 |
| 1662 | SSRD_HUMAN | 19 kDa | 0 | 2 |
| 1663 | STMN1_HUMAN | 17 kDa | 0 | 2 |
| 1664 | STRBP_HUMAN | 74 kDa | 2 | 0 |
| 1665 | SYFA_HUMAN | 58 kDa | 2 | 0 |
| 1666 | TBC15_HUMAN | 79 kDa | 0 | 2 |
| 1667 | TF2B_HUMAN | 35 kDa | 2 | 0 |
| 1668 | THOC1_HUMAN | 76 kDa | 2 | 0 |
| 1669 | TMM33_HUMAN | 28 kDa | 2 | 0 |
| 1670 | TMOD3_HUMAN | 40 kDa | 2 | 0 |
| 1671 | TNPO2_HUMAN | 101 kDa | 0 | 2 |
| 1672 | TRM6_HUMAN | 56 kDa | 2 | 0 |
| 1673 | TRPT1_HUMAN | 28 kDa | 0 | 2 |
| 1674 | TSNAX_HUMAN | 33 kDa | 2 | 0 |
| 1675 | UBA3_HUMAN | 52 kDa | 2 | 0 |
| 1676 | VTA1_HUMAN | 34 kDa | 2 | 0 |
| 1677 | XPO7_HUMAN | 124 kDa | 2 | 0 |
| 1678 | ZN148_HUMAN | 89 kDa | 0 | 2 |
| 1679 | ZN217_HUMAN | 115 kDa | 2 | 0 |
| 1680 | ZN398_HUMAN | 71 kDa | 2 | 0 |
| 1681 | ZN550_HUMAN | 48 kDa | 2 | 0 |

**Table S3. The miRNA up- or down-regulated by AGO2 shRNA**

| **miRNA**  Comparison | My5/LV VS. My5/LV/sh72 | My5/LV/EIF2C2 VS. My5/LV/sh72 | My5/CRBN VS. My5/CRBN/sh72 | My5/CRBN VS. My5/LV/sh72 | My5/LV VS.  My5/CRBN/sh72 | My5/CRBN/EIF2C2 VS. My5/CRBN/sh72 | My5/LV/EIF2C2  VS. My5/CRBN/sh72 |
| --- | --- | --- | --- | --- | --- | --- | --- |
| up-regulated |  | | | | | | |
| has-miR-410-3p | 10.4* | 6.4 | 14.3 | 9.6 | 15.5 | 24.5 | 9.5 |
| hsa-miR-802 |  |  | 5.7 |  | 8.9 |  | 4.5 |
| down-regulated |  | | | | | | |
| hsa-miR-1-3p | -4.1 | -4.2 |  | -8.2 |  |  |  |
| hsa-miR-103a-3p | -5.9 | -6 |  | -5.5 |  | -4.1 | -4.1 |
| hsa-miR-107 | -4.6 | -9.4 | -4.5 | -5.6 |  | -7.9 | -7.5 |
| hsa-miR-10a-5p | -5.5 | -4.3 |  | -7.6 |  |  |  |
| hsa-miR-128-3p | -4.4 | -5.4 |  | -4.3 |  |  |  |
| hsa-miR-1324 | -8.4 |  |  |  | -15.6 | -4.4 |  |
| hsa-miR-153-3p | -5 | -10.7 |  | -5.3 |  |  | -4.4 |
| hsa-miR-155-5p |  |  | -9.6 |  | -6.6 | -7.5 |  |
| hsa-miR-15a-5p | -4 | -4.1 |  |  | -4.6 |  | -4.6 |
| hsa-miR-15b-3p |  | -5 |  | -4 |  | -4.2 |  |
| hsa-miR-186-5p |  |  | -5.7 |  | -5.8 | -8.9 | -5.4 |
| hsa-miR-186-3p | -5.2 | -5.5 |  | -9.3 |  |  |  |
| hsa-miR-18a-3p |  | -5.2 |  |  |  | -5.8 | -4.4 |
| hsa-miR-199a-5p | -5.5 | -7.2 |  | -5.4 |  |  |  |
| hsa-miR-199b-5p | -5.3 | -5.1 |  | -6.4 |  | -4.5 |  |
| hsa-miR-223-3p |  | -5.8 |  | -14.3 |  | -4.9 |  |
| hsa-miR-301a-3p | -15.9 | -24.2 | -9.5 | -17.3 | -8.7 | -20.2 | -13.4 |
| hsa-miR-32-5p | -8.7 | -5.4 |  | -6 | -5.1 |  |  |
| hsa-miR-335-5p |  | -5.3 |  |  |  | -4.4 | -4.5 |
| hsa-miR-335-3p |  | -5.8 |  |  |  | -5.3 | -6.7 |
| hsa-miR-33b-5p | -6.5 | -6.1 |  | -4.6 | -5.4 |  | -5.1 |
| hsa-miR-361-5p | -6.8 | -5.5 | -4.2 | -8.5 |  |  |  |
| hsa-miR-505-3p | -6.1 | -9 |  |  | -4 | -5.5 | -5.9 |
| hsa-miR-513a-5p | -4.7 |  |  |  | -12.7 |  | -7.5 |
| hsa-miR-532-5p | -4.7 | -5.1 | -4.2 | -6 |  | -4.6 |  |
| hsa-miR-542-3p | -8.7 | -4.1 |  | -4 | -4.7 |  |  |
| hsa-miR-542-5p |  | -4.3 |  |  |  |  |  |
| hsa-miR-551b-3p |  | -7.3 |  | -14.1 |  | -5.6 |  |
| hsa-miR-652-3p | -5.4 | -5.3 |  | -5 |  |  |  |
| hsa-miR-7-5p |  | -8.8 |  |  | -4.6 | -6 | -12.4 |
| hsa-miR-744-5p | -6.5 | -9 | -5.7 | -11.9 |  | -10.8 | -4.4 |
| hsa-miR-98-5p | -7.1 | -19.9 | -4.2 | -7.8 |  | -8.1 | -10.7 |

*The number in each column represents the fold change of miRNAs expression.

**Table S4. The miRNA up- or down-regulated by lenalidomide treatment for 5 days.**

| Comparison  **miRNA** | My5/LV VS.   My5/LV+Len 5 Days | My5/CRBN VS.  My5/LV+Len 5 days | My5/LV/EIF2C2 VS.  My5/LV+Len 5 days | My5/CRBN/EIF2C2 VS.  My5/LV+ 5 days | My5/LV+Len72h VS.  My5/LV+Len 5 days | My5/LV/EIF2C2+ Len 3 days VS. My5/LV+Len 5 days | My5/CRBN+ Len 3 days VS.  My5/LV+Len 5days | My5/CRBN/EIF2C2+ Len 3days VS. My5/LV+Len 5days | My5/LV VS.  My5/CRBN+Len 5days | My5/CRBN VS.  My5/CRBN+Len 5days | My5/LV /EIF2C2 VS.  My5/CRBN+Len 5days | My5/CRBN /EIF2C2 VS.  My5/CRBN+Len 5days | My5/LV +Len 3days VS.  My5/CRBN+Len 5days | My5/LV/EIF2C2 +Len 3days VS. My5/CRBN+Len 5days | My5/CRBN +Len 3days VS.  My5/CRBN+Len 5days | My5/CRBN/EIF2C2 +Len 3days VS. My5/CRBN+Len 5days |
| --- | --- | --- | --- | --- | --- | --- | --- | --- | --- | --- | --- | --- | --- | --- | --- | --- |
| up-regulated | | | | | | | | | | | | | | | | |
| hsa-miR-10a-5p |  |  | 4.6* | 7.6 |  |  |  |  | 5.3 |  | 6.7 | 11.2 |  | 5.5 |  |  |
| has-miR-10b-5p | 4 |  | 7.1 | 15.9 |  | 6.9 |  |  |  |  | 5.3 | 11.8 |  | 5.1 |  |  |
| hsa-miR-135a-5p |  | 8 |  | 19.1 |  |  |  |  | 6.9 | 15.8 | 6 | 37.6 |  | 7.6 |  | 4.8 |
| has-miR-135b-5p |  |  | 6.2 | 8.5 |  | 5.2 |  |  | 4.3 | 4.6 | 11.5 | 15.9 |  | 9.6 |  | 4.9 |
| has-miR-139-5p |  |  | 6 |  |  | 5.4 |  | 6.4 |  |  | 5.3 |  |  | 4.7 |  | 5.6 |
| has-miR-142-3p |  |  |  |  |  |  |  |  | 6.7 | 5.1 | 5 | 4.9 | 5.6 | 4.9 | 7.8 | 5.5 |
| hsa-miR-143-3p | 4.8 |  |  |  |  |  |  |  | 27.2 | 4.5 | 17.4 | 4.1 | 17.8 | 7.4 |  |  |
| hsa-miR-144-5p |  |  |  |  |  | 12.3 |  |  | 4.7 | 6.4 | 5 |  | 8.6 | 26.9 |  |  |
| hsa-miR-145-3p |  |  |  |  |  |  |  |  | 14.2 | 6 | 8.7 | 5.3 | 14.9 | 9.8 |  |  |
| has-miR-205-5p |  |  |  |  |  |  |  |  | 31.3 | 15.6 | 40.6 | 76.2 | 21.9 | 37.9 |  |  |
| has-miR-22-3p |  |  |  |  |  |  |  |  | 4.6 | 4.3 | 8.1 |  | 4.2 | 5.8 |  |  |
| hsa-miR-223-3p |  |  | 4.7 |  |  |  |  |  | 14 |  | 18.9 | 4.6 | 8.5 | 9.1 |  |  |
| has-miR-455-5p |  |  |  |  |  |  |  |  | 30.9 | 14.5 | 16 | 7.5 | 16.8 | 14 |  |  |
| hsa-miR-488-3p |  |  |  |  |  | 31.6 |  | 10.4 |  |  |  | 4.7 | 5.1 | 41.6 |  | 13.7 |
| down-regulated | | | | | | | | | | | | | | | | |
| hsa-miR-124-3p | -8.6 | -8.2 | -13.3 |  | -8.3 | -5.5 | -14 | -8.4 | -7.4 | -7.1 | -11.5 |  | -7.2 | -4.7 | -12 | -7.2 |
| hsa-miR-1265 |  |  |  |  |  |  | -17.9 | -4.1 | -4 |  | -5.5 | -4.4 | -4.6 |  | -26.2 | -6 |
| hsa-miR-1290 | -4.3 | -4.7 | -7.6 |  |  |  | -4.6 | -4.2 | -4 | -4.4 | -7.2 |  |  |  | -4.4 |  |
| hsa-miR-137 | -4.5 |  |  | -4.8 |  |  |  |  | -8.4 | -4.3 | -6.8 | -9 |  | -6.6 |  |  |
| has-miR-147a | -6.2 | -5.9 | -8.5 |  | -106 | -9 | -6.7 | -5.2 |  |  |  |  |  |  |  |  |
| hsa-miR-155-5p | -20.4 | -29.8 | -5.1 | -23.1 | -7.9 |  | -46.4 | -26.7 |  |  |  |  |  |  |  |  |
| hsa-miR-155-3p | -8.7 | -5.1 | -4 |  | -4.4 | -7.3 | -4.8 |  | -7.6 | -4.5 |  |  |  | -6.4 | -4.2 |  |
| hsa-miR-186-3p | -8.4 | -14.9 | -8.9 | -14.6 | -21.5 | -16.5 | -22.9 | -9.5 | -5.4 | -46.6 | -5.6 | -9.2 | -13.7 | -10.5 | -14.5 | -6 |
| has-miR-191-3p | -7.8 | -4.9 | -16.8 | -7.9 | -5.5 |  | -7.8 | -7.2 | -74.9 |  | - 160 | -75.2 | -52.3 | -32.5 | -74.7 | -68.8 |
| has-miR-196b-5p | -6.6 |  | -5 |  | -4.2 |  | -6.3 |  | -6.8 |  | -5.2 |  | -4.3 |  | -6.5 |  |
| hsa-miR-211-5p | -4.4 |  | -6.6 |  |  |  | -5.1 |  | -4.3 |  | -6.3 |  |  |  | -4.9 |  |
| has-miR-212-3p | -10.1 | -8.9 | -5.1 |  | -8 | -6.8 | -9.4 | -5.4 | -10.1 | -8.9 | -5.1 |  | -8 | -6.8 | -9.4 | -5.4 |
| has-miR-217 |  |  |  | -6.5 | -4.5 | -4.7 |  |  | -7.3 | -4.3 | -8 | -14.1 | -9.7 | -10.2 | -4.8 | -4.9 |
| has-miR-218-1-3p | -5 |  | -7.5 | -4.4 |  |  | -7.6 |  |  |  | -5.8 |  |  |  | -5.8 |  |
| has-miR-222-5p |  |  | -6.7 | -4.5 |  | -4.1 | -9.4 | -5.6 |  |  | -4.3 |  |  |  | -6 |  |
| has-miR-224-5p |  |  |  |  |  |  | -7.7 |  |  | -8.3 | -8.8 | -9.3 | -4.3 | -5.8 | -19.7 | -5.7 |
| has-miR-300 | -19.4 | -19.8 | -36.7 | -16.3 | -19.7 | -11.1 | -38.3 | -22.2 | -24 | -24.5 | -45.5 | -20.2 | -24.4 | -13.7 | -47.5 | -27.6 |
| has-miR-302c-3p | -45.7 | -54 | -44.1 | -15.6 | -33.7 | -31.4 | -61.6 | -43.6 | -17.7 | -21 | -17.1 | -6.1 | -13.1 | -12.2 | -23.9 | -16.9 |
| has-miR-31-5p | -5.6 | -4.6 | -5.6 |  | -6.1 | -6.4 | -7.7 | -6.2 | -5.6 | -4.6 | -5.5 |  | -6 | -6.4 | -7.7 | -6.2 |
| has-miR-323b-5p | -6.5 | -4.5 | -6 | -12.5 | -5.4 | -8 | -4.9 | -6.8 | -5.5 |  | -5.1 | -10.5 | -4.6 | -6.7 | -4.2 | -5.7 |
| has-miR-342-3p | -  2512 | -1832 | -1951 | -4596 | -2474 | -2349 | -2491 | -2049 |  |  |  |  |  |  |  |  |
| has-miR-370-3p | -5.4 | -8.4 |  |  | -4.8 | -6 | -6.9 | -5 | -4.3 | -6.8 |  |  |  | -4.9 | -5.6 | -4 |
| has-miR-372-3p | -4.1 |  | -6 |  | -5.4 |  |  | -5.3 | -4 |  | -5.9 |  | -5.3 |  |  | -5.2 |
| has-miR-376a-3p |  |  |  |  |  |  |  |  | -7.9 | -9.5 | -9.5 |  | -7.2 | -7.3 | -4.6 | -7.7 |
| has-miR-377-5p | -4.5 |  |  | -4.8 |  |  | -7.2 |  | -8.4 | -4.3 | -5.4 | -9 |  |  | -13.5 |  |
| has-miR-379-5p | -12.8 | -11.8 | -10.9 | -5.8 | -10.7 | -7.4 | -27.1 | -13.3 | -15.6 | -14.4 | -13.3 | -7 | -13.1 | -9.1 | -33.1 | -16.3 |
| has-miR-411-5p | -5.9 |  | -8 |  |  | -6.3 | -8.2 |  |  |  | -6.3 |  |  | -4.9 | -6.4 |  |
| has-miR-425-3p |  |  | -5.4 |  |  |  |  |  | -4.7 | -4.3 |  | -5.1 | -4.5 |  | -5.9 | -4.7 |
| has-miR-431-5p | -16.3 |  | -15 | -4.5 | -13.5 | -37.6 | -12.5 | -15 | -10.3 | -10.7 | -9.5 |  | -8.6 | -23.9 | -7.9 | -9.5 |
| has-miR-432-5p |  | -16.9 |  |  |  |  |  |  | -5.8 | -4.7 | -5.1 |  | -4 |  | -5.2 |  |
| has-miR-486-3p | -5.4 |  | -5.9 |  | -6.6 | -4.7 |  |  | -12.9 | -6.3 | -14.1 | -9 | -15.7 | -11.2 | -8.5 | -5.1 |
| has-miR-499a-3p | -4.8 |  | -12.3 | -4.3 | -6 | -4.5 | -11 | -7.2 |  |  | -9.3 |  | -4.5 |  | -8.4 | -5.5 |
| has-miR-509-3p |  |  |  |  | -4.4 |  | -6.9 |  |  | -4.1 |  |  | -5.3 |  | -8.2 |  |
| has-miR-514a-3p |  |  |  |  |  |  |  |  | -19.3 | -7.6 | -23.6 | -9 | -20.7 | -6.2 | -7.8 | -5.1 |
| hsa-miR-517b-3p |  |  | -7.3 | -7.9 | -6.8 |  | -5.1 | -5.1 |  |  | -5 | -5.4 | -4.6 |  |  |  |
| has-miR-549a | -5.2 | -8.7 | -25.4 | -5.5 | -9.5 | -6 | -12.7 | -7.6 |  |  | -4.4 |  |  |  |  |  |
| has-miR-581 | -8.2 | -14.8 | -8.2 |  | -8.1 | -9.1 | -33.3 | -11.6 | -4.4 | -8 | -4.4 |  | -4.4 | -4.9 | -18.1 | -6.3 |
| has-miR-588 | -5.1 |  | -4.6 |  |  | -6 | -4.9 |  |  |  |  |  |  | -4.4 |  |  |
| has-miR-626 |  |  | -9.9 | -4.6 |  | -4.1 | -10.7 | -8.1 |  |  | -6.8 |  |  |  | -7.3 | -5.6 |
| has-miR-639 | -12.3 | -13.9 | -16 | -12.7 | -12.1 | -12.3 | -11.2 | -12.3 | -11.1 | -12.5 | -14.4 | -11.4 | -10.8 | -11.1 | -10.1 | -11 |
| has-miR-643 | -14 | -14.3 | -10.8 | -4.2 | -11.5 | -12.6 | -16.4 | -13.2 | -7.3 | -7.5 | -5.7 |  | -6 | -6.6 | -8.6 | -6.9 |
| has-miR-708-5p | -9.3 | -15.5 | -6 |  | -7.1 | -9.7 | -8.2 | -7.3 | -17.9 | -29.7 | -11.5 | -4.2 | -13.6 | -18.6 | -15.6 | -13.9 |
| has-miR-720 | -13.7 | -31.1 | -15.4 | -9.1 | -15.5 | -17.4 | -23 | -15.9 | -24.8 | -56.2 | -27.9 | -16.4 | -27.9 | -31.5 | -41.6 | -28.8 |

*The number in each column represents the fold change of miRNAs expression.
